# ﻿Vascular plants of east-central Baffin Island, Nunavut, Canada: an annotated checklist of a mid-Arctic flora

**DOI:** 10.3897/phytokeys.264.162520

**Published:** 2025-10-13

**Authors:** Lynn J. Gillespie, Paul C. Sokoloff, Geoffrey A. Levin

**Affiliations:** 1 Botany Section and Centre for Arctic Knowledge and Exploration, Research & Collections, Canadian Museum of Nature, PO Box 3443, Station D, Ottawa, Ontario, K1P 6P4, Canada Research & Collections, Canadian Museum of Nature Ottawa Canada

**Keywords:** Arctic, Baffin Island, biodiversity, Canadian Arctic Archipelago, checklist, floristics, Nunavut, range extension, vascular plants

## Abstract

Baffin Island in the eastern Canadian Arctic is the largest island in Canada and the fifth largest in the world. East-central Baffin Island, a spectacular mountainous area covering over 57,000 km^2^, with long fiords, deep U-shaped valleys, glaciers, and icecaps, is one of the least botanically collected and documented areas of the island. Here, we report the results of a floristic study of vascular plant diversity in east-central Baffin Island based on historical and recent collections and field studies. Previous published accounts covering the flora area cited no or few voucher collections. We compiled a dataset of 3,206 unique collections made between the 1860s and 2022, including 850 collected by us. The vascular plant flora comprises 26 families, 71 genera, 163 species (4 with two subspecies each), and 2 nothospecies. The six most species-rich families are Cyperaceae (26 species), Poaceae (24), Brassicaceae (16), Caryophyllaceae (15), Saxifragaceae (11), and Asteraceae (11). The largest genera are *Carex* (19 species), *Draba* (9), *Potentilla* (8), and *Saxifraga* (8). More than half of the taxa are circumpolar in distribution, few are restricted to North America, and none are endemic to Baffin Island. All taxa found in east-central Baffin Island are native. Many taxa appear to be rare in the flora area; 33 are known from only 1–3 collections and 45 from only one or two localities. Remarkably high diversity was recorded in valleys at the heads of fiords, accounting for 94% of the total flora, which may be attributed to a warmer climate along with high habitat diversity. We document 21 taxa new to the flora area. Particularly noteworthy records include *Puccinellia bruggemannii*, newly reported from Baffin Island; *Ranunculus sabinei* and *Crucihimalaya bursifolia*, not known elsewhere on Baffin Island and disjunct by over 1,000 and 500 km, respectively; *Carex holostoma*, *Diapensia lapponica*, *Harrimanella hypnoides*, and *Vaccinium vitis-idaea*, northernmost records for Canada; and *Arctous alpina* and *Ranunculus trichophyllus*, northernmost records for eastern Canada. Our data show that species new to the flora area continue to be reported and that vast areas within it are botanically under-explored, so we expect that future work will further expand our understanding of the flora of east-central Baffin Island. This study provides essential baseline data for monitoring the impacts of climate change on the flora of this Arctic region.

## ﻿﻿Introduction

Climate change is affecting plants worldwide, resulting in shifts in distribution and, in some cases, extinctions. The Arctic is undergoing rapid climate change, warming at twice the average global rate (Lenssen et al. 2019; GISTEMP Team 2020). Warming temperatures, permafrost melting, and changing snow cover have an impact on vegetation and plant diversity. In Canada, the Arctic ecozone covers about 40% of the landmass and accounts for 25% of the global Arctic. Nunavut, the Inuit homeland, covers over 2 million km^2^ and encompasses much of the Canadian Arctic. Documenting current plant diversity and distributions in the Arctic provides necessary baseline data for monitoring and understanding the impacts of climate change on plant diversity and vegetation.

Unfortunately, much of the Arctic, including in Canada, has been poorly explored botanically. For example, Panchen et al. (2019), in a study of Nunavut herbarium specimens at the National Herbarium of Canada, found that specimens had been collected from only 0.63% of the land area in Nunavut, with the highest density from near communities, research stations, and other facilities. Not surprisingly, recent botanical surveys combined with re-examination of historical herbarium specimens continue to document many new distribution records in Arctic Canada (Saarela et al. 2012; Saarela et al. 2013; Gillespie et al. 2015; Sokoloff 2015; Saarela et al. 2017; Saarela et al. 2020a, 2020b; Saarela et al. 2023; Gillespie et al. 2024). Clearly, more field- and collections-based studies are needed to understand Arctic plant distributions and diversity patterns.

Baffin Island, in east-central Nunavut, is the largest island in Canada and the fifth largest island in the world (507,451 km^2^). Plant diversity of Baffin Island has been documented with collections for almost 170 years; however, as in the rest of Nunavut, coverage has been uneven. Comprehensive collection-based floristic inventories have mostly focused on southern Baffin Island: Iqaluit (formerly Frobisher Bay) (Calder 1951), Ogac Lake on Frobisher Bay’s south side (McLaren 1964), Penny Highlands on the Cumberland Peninsula (Schwarzenbach 2011), Dorset and Mallik islands off the southern Foxe Peninsula (Saarela et al. 2020a), and Katannilik Territorial Park, Kimmirut, and vicinity (Saarela et al. 2023). Only two collections-based floristic studies have been published for central Baffin Island: Bray Island off the west coast (Dansereau 1954) and Agguttinni Territorial Park on the east side (Gillespie et al. 2024). Plant lists and checklists have often been included in studies focused on vegetation, but these typically did not cite collections, e.g., a study of Auyuittuq National Park on southeast Baffin Island (Hines and Moore 1988).

East-central Baffin Island is one of the least botanically collected and documented areas of Baffin Island. Taylor, a surgeon on a whaling vessel, made the first collections at two coastal sites during the period 1856–61 and published a list of species for the area bordering Baffin Bay and Davis Strait (Taylor 1863). While other collections have been made in the subsequent nearly 170 years, many early collectors were not botanists and made only incidental collections, and collections made by botanists were mostly in the vicinity of the only community along the coast, Clyde River, where the annual supply boat stopped. The first extensive plant collections in the area were made as part of the 1950 Baffin Island Expedition (Baird et al. 1950; Baird 1951, 1952); the majority of these collections were made at the head of Clyde Inlet, where the expedition botanists were based. Unfortunately, comprehensive results from the expedition were never published; only two small papers on vascular plants were published (Dansereau 1954; Dansereau and Steiner 1956), and neither dealt with floristics, species diversity, or vegetation of east-central Baffin Island. In 1965–1967, several botanists made numerous collections at Inugsuin Fiord and vicinity; only Hainault (1966) wrote a report, which included a list of plant species but cited no collections.

Four regional floras that include Baffin Island have been published. Polunin (1940), in “Botany of the Canadian Eastern Arctic”, cited many of the early collections from east-central Baffin Island, including those of Taylor (1863), plus his own collections and observations made mostly at Clyde River in 1934 and 1936. Later Canadian Arctic floras mapped collections from the flora area (Porsild 1957; Porsild and Cody 1980; Aiken et al. 2007), but no specimens were cited, and it is often difficult to track down the collections that the map dots are based on. Citing collections as vouchers is essential to enable verification and repeatability of results. This is especially important given the many taxonomic, nomenclatural, and species circumscription changes that have taken place since the publication of these floras.

In 2021, we conducted a botanical inventory to support resource assessment of the newly established Agguttinni Territorial Park (Agguttinni TP) in partnership with Nunavut Parks and Special Places. Located north of Clyde River, the park is the largest of Nunavut’s territorial parks, covering an area of 16,465 km^2^. Previous collecting in this large remote area had been very limited. In our resulting publication, we recorded 141 species of vascular plants and extremely variable levels of species diversity across localities, with the heads of fiords having by far the highest diversity (Gillespie et al. 2024).

Here, we report the results of our research on the vascular plants of east-central Baffin Island, building on our recent study of the flora of Agguttinni TP (Gillespie et al. 2024) but encompassing a much larger area and including many more historical collections.

### ﻿﻿Study area

#### ﻿Geography

**Figure 1.**
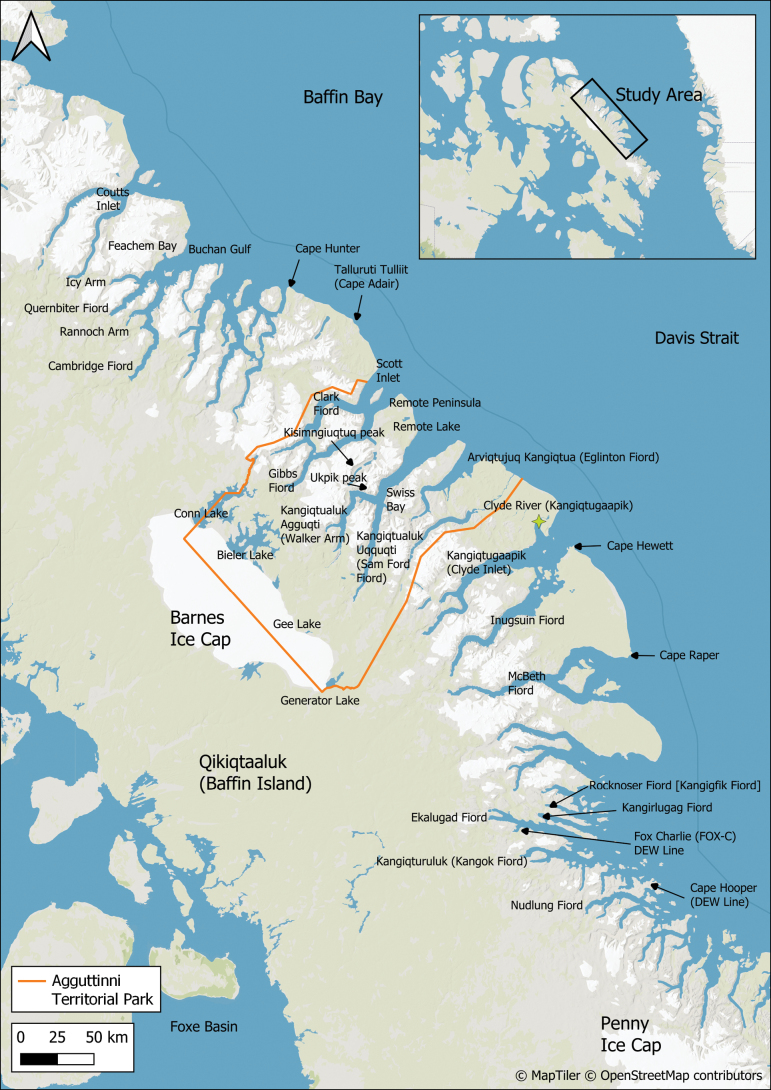
Map of central Baffin Island showing the locations of places and features in east-central Baffin Island mentioned in this study. The inset map shows the location of the study area in the eastern Canadian Arctic.

Baffin Island is located in the eastern Canadian Arctic Islands in the Qikiqtaaluk region of Nunavut, Canada (Fig. 1). Qikiqtaaluk means “very big island” in Inuktitut, a fitting reference to Baffin Island as the largest island in the region and in Canada, with a land area of about 507,450 km^2^. Despite Baffin Island being the most populated area of Nunavut, with about 50% of Nunavut’s population, density is very low, estimated at 0.03 inhabitants per km^2^. There are eight communities on the island, ranging in size from the smallest hamlet, Qikiqtarjuaq, with a population of fewer than 600, to Iqaluit, the capital of Nunavut, with a growing population of about 7,500. No roads connect the communities, and travel is by scheduled or chartered plane, snowmobile in winter, or boat in summer.

Baffin Island lies within two of Environment Canada’s 15 Canadian ecozones, Arctic Cordillera and Northern Arctic, and includes 13 of Canada’s 217 ecoregions (Ecological Stratification Working Group 1995; http://www.ecozones.ca/english/zone/index.html). The eastern part of Baffin Island is part of the Arctic Cordillera ecozone and encompasses the Baffin Mountains ecoregion. This part is dominated by an extensive mountain chain, the Baffin Mountains, part of the Arctic Cordillera extending from Labrador north to Ellesmere Island. The highest peaks are over 1,900 m in elevation, with Mount Odin in Auyuittuq National Park the highest at 2,147 m. The remainder of the island is included in the Northern Arctic ecozone. The western part is mostly flat or gently sloping, and in the middle is a central plateau, 400–700 m in elevation. Baffin Island was entirely glaciated during the last Ice Age. The Penny and Barnes ice caps, 6,000 km^2^ and 6,300 km^2^ in area, respectively, and hundreds of meters thick, are remnants of the Laurentide Ice Sheet. Both ice caps are receding, the Penny Ice Cap by as much as 4 m/year at lower elevations (Schaffer et al. 2020).

**Figure 2.**
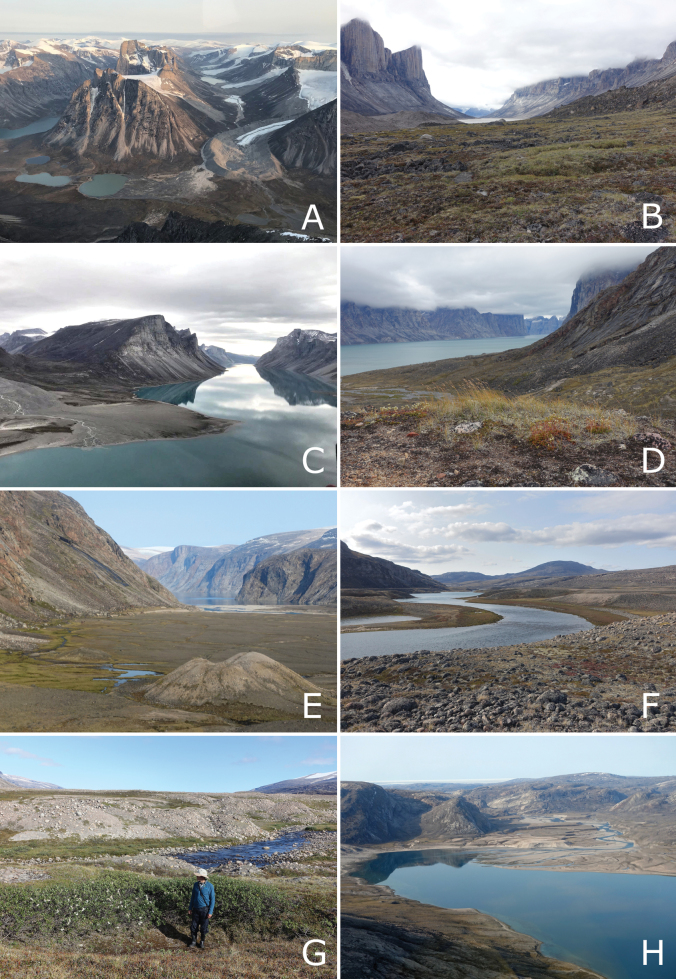
Landforms and habitats in east-central Baffin Island—Baffin Mountains ecoregion. A. Atagulisaktalik (valley), with head of Arviqtujuq Kangiqtua [Eglinton Fiord] on left; B. Stewart Valley, rocky *Cassiope–Carex* tundra in foreground; C. Tasialuk, a landlocked fiord; D. Tingijattut, side valley along Kangiqtualuk Agguqti, Anthoxanthum monticola subsp. alpinum and *Saxifraga tricuspidata* on the hilltop in the foreground; E. Gibbs Fiord, view of head of fiord, polygon-patterned ground on flat valley bottom, kame in foreground; F. Kangiqtualuk Agguqti, river valley at the head of the fiord; G. Kangiqtualuk Agguqti, willow thicket (*Salix richardsonii*) in a small side valley; H. Kangiqtualuk Uqquqti, view of the head of the fiord and river valley. Photos by P.C. Sokoloff (A, C) and L.J. Gillespie (B, D–H).

East-central Baffin Island is dominated by a broad band of rugged mountains intersected by long fiords and U-shaped glacial valleys (Fig. 2). This area, part of the Baffin Mountains ecoregion (Ecological Stratification Working Group 1995), includes some of the most spectacular scenery in Canada, with deep fiords bordered by near-vertical cliffs and hanging glaciers. The two highest peaks are Kisimngiuqtuq (1,905 m) and Ukpik (1,809 m). The largest fiords—Inugsuin Fiord, Kangiqtugaapik [Clyde Inlet], Kangiqtualuk Uqquqti [Sam Ford Fiord] (Fig. 2G), and McBeth Fiord—extend 100–120 km inland, with valleys at their heads extending further inland.

**Figure 3.**
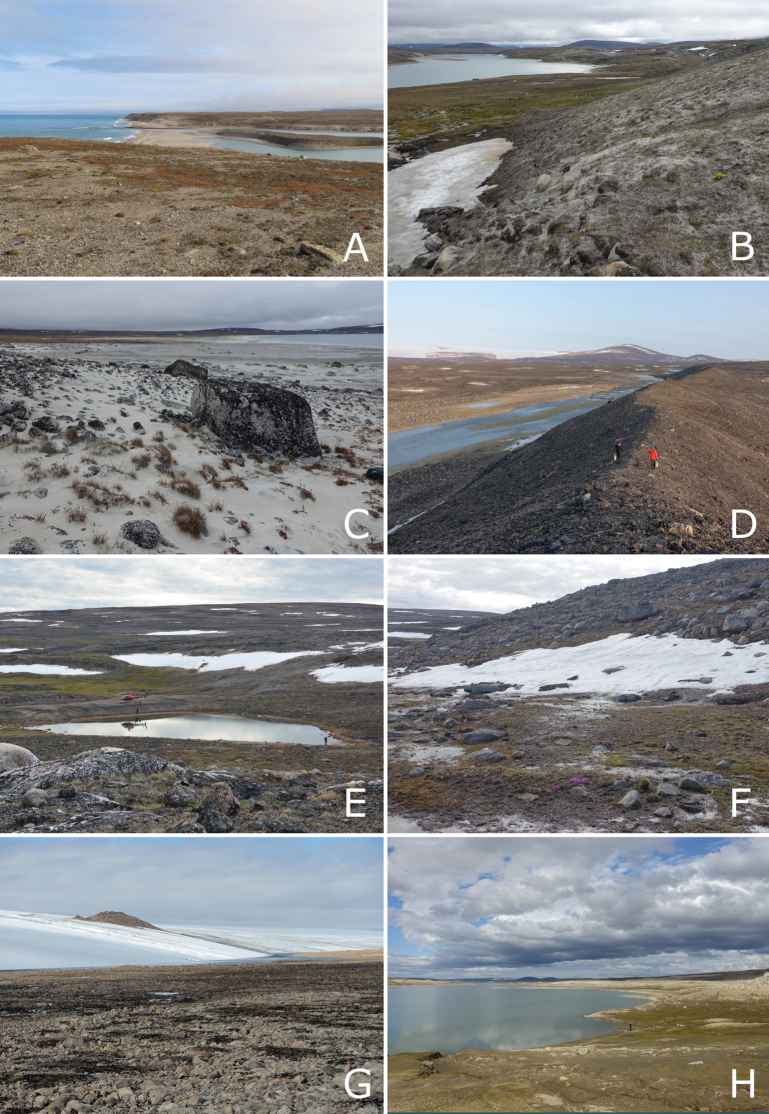
Landforms and habitats in east-central Baffin Island. A–C. Baffin Island Coastal Lowlands ecoregion. A. Kuugaaluk river mouth, coastal tundra in foreground; B. Kuugaaluk, sand hills along the river, with snowbed and *Ranunculus nivalis* in the foreground; C. Niaqurnaaluk-Qassialuit, a long sand beach between two capes, *Luzula confusa* hummocks with Anthoxanthum monticola subsp. alpinum, *Oxyria digyna*, and *Poa arctica* in foreground. D–H. Baffin Island Uplands ecoregion; D. “Gibbs Esker,” esker on plateau above head of Gibbs Fiord, rocky barrens and glacier in background; E. “Marble Lake,” vegetated meadows around pond and adjacent ice-covered lake, Anthoxanthum monticola subsp. alpinum, Cassiope tetragona subsp. tetragona, and *Salix arctica* between marble outcrops in the foreground; F. “Marble Lake,” snowbed and associated plant community, *Salix arctica* and *Saxifraga oppositifolia* (pink flowers) in foreground; G. Barnes Ice Cap, showing edge of ice cap, recently deglaciated zone devoid of lichens, and rocky barrens with scattered patches of lichen, moss, and *Luzula* spp; H. Generator Lake, recently exposed shoreline, with green moss meadow and a few scattered vascular plants. Photos by G.A. Levin (A), L.J. Gillespie (B–D, F–H), and P.C. Sokoloff (E).

To the east, the mountains either descend directly to Baffin Bay or into a flat to gently sloping coastal plain. The Baffin Island Coastal Lowlands ecoregion occupies the central coastal area from the north side of Home Bay north to Cape Hunter (Fig. 3A–C). The coastal lowlands are particularly prominent, extending up to 40 km inland, from Home Bay to the mouth of Arviqtujuq Kangiqtua [Eglinton Fiord]. Here, the flat coastal plain gently rises to hilly terrain inland before transitioning to mountainous terrain. To the west of the mountains is an upland area, 400–700 m in elevation, of flat to rolling terrain and many small lakes (Fig. 3D–H). This area is part of the Baffin Island Uplands ecoregion of the Northern Arctic ecozone. The Barnes Ice Cap is located in the middle of central Baffin Island and rises to over 1,100 m elevation. Around its perimeter are several very large glacial meltwater lakes, Conn, Bieler, and Generator lakes.

For the purposes of our study, we define east-central Baffin Island as the area between and including Cape Hooper at the south edge of Home Bay northwest to Coutts Inlet, a distance of 560 km. The western boundary, running southeast–northwest, lies approximately at the divide between Baffin Bay to the east and Foxe Basin to the west. The encompassing rectangle is approximately 560 km long and 180 km wide. The land area is about 57,500 km^2^ and ranges in width from 100–180 km.

Clyde River, centrally located in the flora area near the mouth of Clyde Inlet, is the only community on east-central Baffin Island. The hamlet, also known by its Inuktitut name Kangiqtugaapik, meaning “nice little inlet,” has a population of about 1,200 inhabitants. The closest communities are Pond Inlet, located about 415 km northwest of Clyde River on northern Baffin Island, and Qikiqtarjuaq, 375 km southeast.

Agguttinni TP is the only officially protected area in the flora area. However, due to its remote location and lack of infrastructure, most of the study area has been little impacted by human activity. Immediately south of the study area is Auyuittuq National Park, a scenic mountainous area including the Penny Ice Cap, the two highest peaks on Baffin Island, and numerous fiords. Sirmilik National Park, including Bylot Island, lies northwest of the study area.

Place names, landforms, and water features in Nunavut often have both Inuktitut and English names, and many are now officially known by their Inuktitut name. Official names as recognized in the Government of Canada Canadian Geographical Names Database (https://geonames.nrcan.gc.ca/), whether Inuktitut or English, are used throughout. Formerly used English names and non-official Inuktitut names (at least at the time of publication) are provided in square brackets.

#### ﻿Geology

Bedrock is Precambrian Archean and Proterozoic igneous and metamorphic rock, primarily made up of gneiss, monzogranite–granodiorites, and migmatites (Geological Survey of Canada, Maps 1458A, 1582A). There are scattered outcrops of Flint Lake Formation marble and dolomite and Mary River Formation amphibolites and gneiss on the Barnes Plateau. Quaternary glacial drift with associated marine, lacustrine, and fluvial deposits covers much of the coastal lowlands and interior plateau and uplands (e.g., Utting and Kerr 2011; Smith et al. 2012). Glacial deposits include till, moraines, eskers, and kames. The large flat-bottomed U-shaped valleys at the heads of fiords have extensive glaciofluvial terraces and more recent alluvial plains and terraces. Glacial surface features, with a focus on moraines and glacial retreat in the Home Bay area, are described in Andrews et al. (1970). Substrates are predominantly acidic (pH < 5.5) in the study area (CAVM Team 2003).

#### ﻿Climate

Weather data for Clyde River recorded by Environment Canada meteorological station Clyde River include long-term data for the 1991–2020 climate normals period (i.e., three-decade averages of climate variables) (Environment Canada 2025). During the 30-year period, Clyde River had a mean annual air temperature of –11.9 °C ± 2.6 SD, a mean July temperature of 5.3 °C ± 1.2, and a mean February temperature of –29.0 °C ± 3.2. Temperatures vary considerably from year to year, with a maximum mean July temperature of 14.8 °C and a minimum mean July temperature of –1.2 °C recorded for that period. Average annual precipitation for the 30-year period was 231.9 mm, with over half falling as snow and the highest monthly averages from July to October.

According to the worldwide Köppen–Geiger climate classification, the study area’s climate is class ET (Polar climate—Tundra), characterized by an average temperature of the warmest month between 0 °C and 10 °C (Beck et al. 2018). Their models predict that the study area will remain in class ET during the period 2071–2100, but that neighboring areas on west-central Baffin Island are predicted to transition to class D (Cold climate) with a warmest month average temperature > 10 °C and a coldest month average temperature ≤ 0 °C.

The study area lies entirely within Bioclimatic subzone C, also called the Middle Arctic zone, one of five Arctic Bioclimatic subzones (Elvebakk 1999; CAVM Team 2003; Walker et al. 2005; Elven et al. 2011). Subzone C is characterized by a mean July temperature of 5–7 °C and a summer warmth index of 9–12 °C (sum of mean monthly temperatures greater than 0 °C) (CAVM Team 2003; Young 1971). The climate at higher elevations in the mountains resembles the colder subzones A and B. At elevations of about 650–1,000 m, the climate is similar to subzone B, characterized by a mean July temperature of 3–5 °C and a summer warmth index of 6–9 °C. At elevations above 1,000 m, the climate is similar to subzone A, characterized by a mean July temperature of 0–3 °C and a summer warmth index less than 6 °C.

#### ﻿Vegetation

According to the Bioclimatic subzone classification, the zonal lowland vegetation of Bioclimatic subzone C consists of two layers, a moss layer 3–5 cm thick and an herbaceous layer 5–10 cm thick, with mostly prostrate dwarf shrubs less than 15 cm tall (CAVM Team 2003). Vegetation is open and patchy, with a vascular plant cover of 5–50%, and local vascular plant floras include 75–150 species. In the mountains, vegetation changes with elevation; thus, vegetation at higher elevations is more similar to that of the colder subzones B (about 650–1,000 m) and A (above about 1,000 m). Subzone B is characterized by prostrate dwarf shrubs less than 5 cm tall, a 5–25% cover of vascular plants, up to 60% cover of mosses and lichens, and local vascular floras with 50–100 species. Subzone A is characterized by vegetation mostly less than 2 cm tall without shrubs, less than 5% cover of vascular plants, up to 40% cover of mosses and lichens, and local vascular floras with fewer than 50 species.

The only published vegetation map that includes the study area is the Circumpolar Arctic Vegetation Map (CAVM Team 2003; Walker et al. 2005). This 1:7,500,000 map was created based on satellite photos along with ground-truthing fieldwork in some parts of the Arctic (fieldwork was apparently not conducted on east-central Baffin Island) and provides a coarse estimate of the vegetation. Five major vegetation types were recognized within the study area. Prostrate dwarf-shrub herb tundra (P1) is dry tundra with patchy vegetation, dominated by prostrate shrubs (less than 5 cm high) such as *Dryas* L. spp. and *Salix arctica* Pall. Prostrate/hemiprostrate dwarf-shrub tundra (P2) is moist to dry tundra dominated by prostrate and hemiprostrate shrubs less than 15 cm tall, with *Cassiope* D.Don spp. often dominant. Rush/grass–forb–cryptogam tundra (G1) is moist tundra with moderate to complete cover of very low-growing plants and lichens. Cryptogam barren complex (bedrock) (B2) includes areas with extensive exposed bedrock covered in lichens, with some more vegetated areas between. Noncarbonate mountain complex (B3) includes mountain vegetation on noncarbonate (i.e., acidic) bedrock, from tundra to mountain barrens depending on the elevation. Vegetation types P2 and B2 dominate in the coastal lowlands and interior uplands of the study area, whereas B3 dominates the mountain zone. G1 is recorded as the main vegetation type in much of the area on the west and south sides of Home Bay, an area of lower rounded mountains, lakes, and broad valleys. Areas of vegetation type P1 are indicated adjacent to the Barnes Ice Cap and the mouth of Kangiqtualuk Uqquqti.

Few studies include descriptions of the vegetation in the study area. Polunin (1948) provided an overview of plant communities in the vicinity of Clyde River. Hainault (1966) gave a limited description of vegetation in the Inugsuin Fiord area. Gillespie et al. (2024) provided descriptions and images of the vegetation of Agguttinni TP.

Several areas on Baffin Island outside the flora area with similar topography and vegetation have been more thoroughly studied, and their flora and vegetation have been better documented than those of east-central Baffin Island. Auyuittuq National Park has been the focus of several vegetation and floristic studies (Lafarge-England 1975; Hines and Moore 1988; Schwarzenbach 2011) and a popular guide (Wilson 1976). Bylot Island in Sirmilik National Park has also been the focus of vegetation and floristic studies (Drury 1962; Zoltai et al. 1983).

The complex topography of the study area results in considerable local climatic and substrate variation. Precipitation, temperature, and insolation vary considerably with elevation and proximity to the coast, resulting in a complex pattern of vegetation. On a smaller scale, differences in moisture and nutrient availability, slope aspect, and exposure result in high microhabitat diversity and large differences in local species diversity.

### ﻿﻿History of botanical collecting on east-central Baffin Island

William E. Parry (1821: 273), during his 1819–20 voyages in the Canadian Arctic, appears to have recorded the first botanical observation along the east coast of Baffin Island near Scott Inlet, but he apparently made no collections (see Suppl. material 1). The first plant collections made in the flora area were those of James Taylor, an Aberdeen-based surgeon who travelled aboard whaling ships during the period 1856–61 (Taylor 1863; Simmons 1913). He made collections on both the Canadian and Greenlandic sides of Baffin Bay, visiting at least five localities on Baffin Island from Cumberland Sound north to Cape Adair. Two sites are located within the flora area: Cape Adair (71°29'44"N, 71°33'55"W), now officially named Talluruti Tulliit, and Scott’s Bay/Scott’s Inlet (71°6'30"N, 71°6'30"W, official name Scott Inlet). Scott Inlet encompasses a large area, 25 × 20 km, at the mouth of Clark and Gibbs fiords where they meet. We located three specimens at MEL from Scott’s Inlet that can be attributed to Taylor, although they do not bear Taylor’s name (see Suppl. material 1) (herbarium acronyms according to Thiers 2025 [continuously updated]).

James Goldie (J.G.) McMillan, a geologist with the Geological Survey of Canada, collected a single specimen at Clyde River (housed at CAN) on 6 September 1909, while on the return voyage of the DGS *Arctic* on Captain J.E. Bernier’s 1908–1910 expedition through the Northwest Passage, which had overwintered at Melville Island (Bernier et al. 1910).

Several botanists visited Clyde River in the 1920s and 1930s, where a Hudson Bay trading post had been established in 1924. Malte Oskar Malte, a botanist with the Biology Division, Geological Survey of Canada, made collections at Clyde River on 18 August 1927, while accompanying the Canadian Arctic Expedition of 1927 aboard the SS *Beothic*, led by Captain George P. Mackenzie (Collins 1929). The 20 specimens seen by us are housed at CAN, MT, and QFA. Nicholas V. Polunin visited Clyde River twice, on 9 September 1934 and 15 September 1936—both times aboard the SS *Nascopie* (Polunin 1940; Cockburn 1983). The 43 specimens examined here are housed at CAN, F, GH, MIN, and US. Rev. Father (Père) Arthème Dutilly also traveled to Clyde River twice—like Polunin, both times aboard the SS *Nascopie* (Smith and Lackenbauer 2014; Eastern Arctic Patrol 1941). Dutilly collected at Clyde on 15 September 1936 (one specimen at QFA was collected on 9 September) and on 21 September 1941; the 60 collections examined by us are housed at CAN, CM, DAO, MIN, MT, QFA, S, and US. Norman Bethune Sanson, then curator of the Banff Park Museum, joined the 1938 voyage of the SS *Nascopie* (Hallworth and Jackson 1985), which called at Clyde River on 5 and 6 September (Shortt and Peters 1942). Two of his specimens (at TRT) have been examined, but there likely are others in undigitized collections (e.g., citations in Ball 1950). John George Oughton, a zoologist with the Royal Ontario Museum, visited Clyde River on 7 September 1939, aboard the SS *Nascopie* on the Eastern Arctic Patrol (Oughton 1939; Chitty 1940), and collected four specimens that we found at TRT.

Three non-botanists collected in the flora area during the 1930s and 1940s. Rupert W. Bartlett accompanied the Greenland Expedition of 1938 with Captain Robert A. Bartlett, collecting plants along the ship’s route (Smallwood 1981). Their only Canadian landing was at Hoffman Cove, “a small sheltered cove inside” Cape Raper (Nutt 1938) on the north side of Isabella Bay, on 16 August 1938. We located 33 of these collections deposited at CAN, MT, and US. Rutherford Platt, photographer and nature writer, collected at Cape Hewitt on 19 August 1947 (or 1948), while part of the MacMillan Arctic Expedition (Platt 1948, 1956) (see Suppl. material 1 for details regarding the year collected). We located 18 specimens, all at NY. D.B. Coombs, working as a surveyor for the Geodetic Survey of Canada, made collections of vascular plants and mosses at Coutts Inlet in August 1948 (Anonymous 1948). Some of these collections include the locality “Manning Fjord,” which is not a current or historic place name in Canada (Canadian Geographic Names Database, https://geonames.nrcan.gc.ca/search-place-names/search, accessed 20 January 2025). Daniels (1956) contains the only published reference to a “Manning Fjord”: “Manning Fjord on the N.E. coast of Baffin Island, lying some 75 miles E.S.E. of Erik Harbour, penetrates approximately 40 miles inland in a south-westerly direction.” Imprecise coordinates given on these specimens suggest this refers to the upper part of Coutts Inlet, which is located about 75 km SSE of Kangiqługaapik [Erik Harbour]. We found 50 of his collections at DAO, MO, and US.

**Figure 4.**
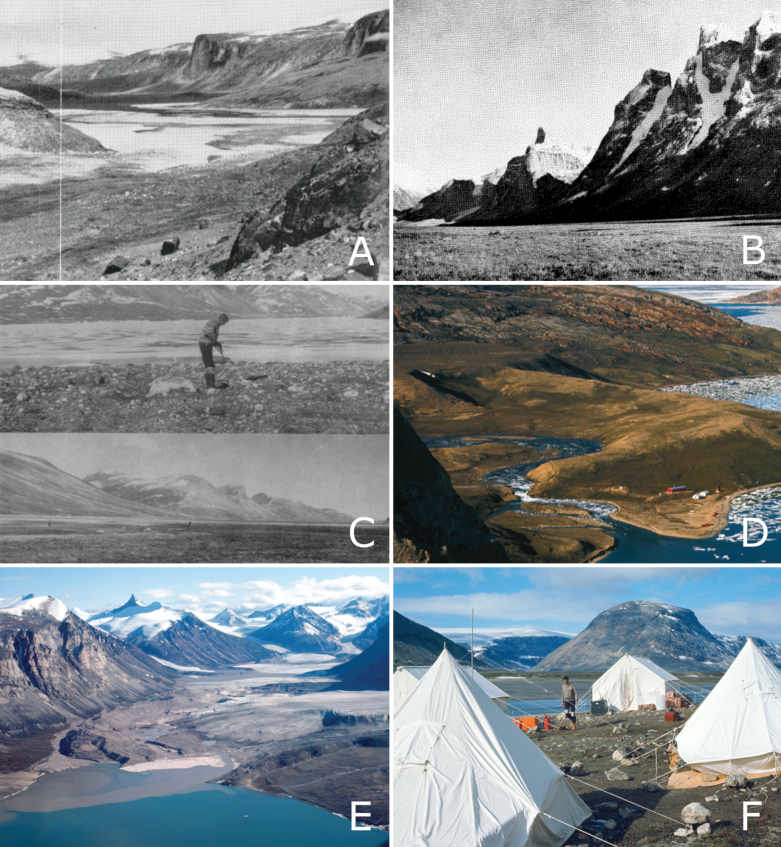
Locations sampled by historical collectors in east-central Baffin Island. A. Kangiqtugaapik Head (head of Clyde Inlet), vicinity of Camp B, 1950, captured by F. Dansereau (adapted from Baird 1951, courtesy of Canadian Geographic); B. Atagulisaktalik (“Revoir Pass”) with Eglinton Tower in background, 1950. Photo by F. Elmiger (adapted from Baird et al. 1950, courtesy of the Arctic Institute of North America); C. Rocknoser Fiord in 1961, head of fiord, N.G. Smith in foreground (top) (adapted from Snell 1991: Fig. 1A, courtesy of *Colonial Waterbirds*); D. Geographical Branch of Canada base camp at the head of Inugsuin Fiord, 1966 (adapted from Ives 2016: Fig. 22, CC BY-NC-ND 4.0); E. Remote Lake, August 1966 (adapted from Ives 2016: Fig. 48, CC BY-NC-ND 4.0); F. Geographical Branch of Canada camp at the head of Ekalugad Fiord, with Mike Church in the background (adapted from fig. 54, Ives 2016: Fig. 54, CC BY-NC-ND 4.0).

During the 1950 Baffin Island Expedition led by P.D. Baird (Baird et al. 1950; Baird 1951, 1952), botanist Pierre Dansereau and ornithologist Vero Copner Wynne-Edwards both made extensive collections from various locations on east-central Baffin Island (Fig. 4A, B). Dansereau collected extensively from numerous sites within walking distance of the expedition’s base camp (“B2”) at the head of Clyde Inlet (Kangiqtugaapik) (Fig. 4A), as well as at Clyde River, the head of Arviqtujuq Kangiqtua [Eglinton Fiord] (Fig. 4B), Umiujaq [Agnes Monument], and Gee Lake (Baird et al. 1950). We located 594 of Dansereau’s collections, made between 30 May and 30 August 1950, at DAO, MIN, and MT (the primary set). Wynne-Edwards also collected primarily at the head of Clyde Inlet, as well as once at the head of Arviqtujuq Kangiqtua, from 1 June to 24 August 1950. There are 300 of his collections at CAN (primary set), with a few at E and LSU. Hans Röthlisberger, a mountaineer with the expedition, made scattered collections at Arviqtujuq Kangiqtua and Swiss Bay (where the expedition had two mountaineering camps) from 12 July to 11 August 1950, 10 of which were located at CAN and ZT (Baird 1952).

John E.H. Martin, an entomologist with the Department of Agriculture, Ottawa (now Agriculture and Agri-Food Canada), collected plants in Clyde River, at the head of Clyde Inlet, and on unspecified islands in Clyde Inlet, from 29 June to 18 August 1958, during the department’s insect survey fieldwork that year (Lotz 1963: 2). We located 63 of Martin’s collections at DAO, MT, QFA, RBCM, TRT, and UTC.

Neal G. Smith, an ornithologist conducting his PhD research on *Larus* gulls on Baffin Island in 1961, collected vascular plant specimens from the Home Bay area between 30 June and 6 August. We located 63 of these, all at CAN. While there is some controversy as to where Smith spent most of that summer’s field season, work by Snell (1991) indicated that Smith was almost certainly based at the head of Rocknoser Fiord (Kangigfik Fiord) (Fig. 4C) for the majority of the season.

Between 1965 and 1967, the Geographical Branch, Department of Energy, Mines and Resources, Canada, established a long-term base camp at the head of Inugsuin Fiord that hosted several botanists over three years of operations (Løken et al. 1966) (Fig. 4D). In 1965, Robert Hainault, based out of the Fowler Herbarium at Queen’s University, traveled to Inugsuin Fiord, collecting extensively around the Geographical Branch base camp, at a field camp near the mouth of Inugsuin Fiord, and in a valley inland at the head of McBeth Fiord, from 6 June to 28 August (Hainault 1966). We found 332 of his collections at ACAD, CAN, DAO, H, MT, QFA, and QK. From 21–22 July 1967, J.A. Parmelee and J.R. Seaborn collected near the Cape Hooper DEW Line site (FOX-4), and from 25 July to 2 August, they collected within walking distance of the Inugsuin Fiord base camp. The main set of their collections is at DAO (160 collections), with at least one collection (Parmelee & Seaborn 3933) distributed more widely to ALTA, BC, BISH, F, L, LD, MO, S, TRH, TROM, TRT, UAM, UTC, and W as a part of a DAO exsiccate set.

During the same period, several researchers associated with the Geographical Branch also made collections on east-central Baffin Island (Ives 2016). From 17 June to 18 August 1966, Jane T. Philpot, Penny A. Crompton, and June Ryder collected from several locations accessed via the Inugsuin Fiord base camp (Ives 2016), including Ekalugad Fiord, Kangok Fiord, Inugsuin Fiord, and Remote Lake on Remote Peninsula (Fig. 4E). We located 82 of these collections at COLO. In 1967, June Ryder collected at Ekalugad Fiord between 17 June and 23 July and Nudlung Fiord on 30 July; we located 24 collections at COLO and UBC. From 13–18 July 1966, Mike Church, studying glacial outwash plains for his PhD research at the University of British Columbia and the Geological Survey of Canada (Church 1972; Ives 2016), collected at the head of Ekalugad Fiord (Fig. 4F); we located eight of his specimens at COLO. He also collected plants in 1967 (dates unknown) at the head of Ekalugad Fiord, three of which are at UBC.

Other collectors were also active in the Home Bay area of east-central Baffin Island in 1967. From 7 July to 8 August 1967, botanist Patrick Webber collected at Ekalugad, Rocknoser, and Kangirlugag fiords; 108 collections are vouchered at CAN, COLO, QFA, and QK. Between 7 and 17 July, J. Richardson and P. Webber made collections from the Fox Charlie (FOX-C) DEW Line station on the southern shore of Ekalugad Fiord, 72 of which are also at CAN, COLO, QFA, and QK. We also located five collections at COLO and UBC made by R. Stock from the head of Ekalugad Fiord on 22 August 1967.

In 1983, Phil Sadler made one collection of *Eriophorum scheuchzeri* while working with a runway maintenance team at the Cape Hooper DEW Line site (FOX-4) (Sadler, personal communication, 2024). This specimen is at ASC.

Over the course of three years, Bruce C. Forbes collected specimens in Clyde River during the periods 19 June–14 August 1988, 27 June–5 July 1989, and 22 June–12 July 1990, while studying the ecology of archaeological sites and tundra disturbance (Forbes 1992, 1993, 1996). We located 116 of his specimens at DAO.

David Oswald of Royal Roads Military College (now Royal Roads University) collected vascular plants in the vicinity of the FOX-C DEW Line station; we located 31 of these, all deposited at QK. Oswald was affiliated with the college’s Environmental Sciences Group (as indicated on the specimen labels), and these plants likely were collected during this group’s study of the environmental impact of the DEW Line sites (Dushenko et al. 1996).

During the Tundra Northwest 1999 expedition (Eriksen et al. 2006), three researchers made collections at Cape Hooper on 30–31 August 1999: botanist Reidar Elven (80 collections found at ALA, CAN, and O), zoologist Johan Hammar (three collections found at CAN and O), and plant ecologist Ulf Molau (one collection found at O).

Several Canadian Museum of Nature(CMN) scientists visited the study area and collected in the early 2000s. In 2008, botanist Julian Starr collected at Swiss Bay on 11 August 2008, during an expedition with Students on Ice; to date only two of these specimens have been processed and are available at CAN. From 8–10 August 2017, Oluwayemisi Dare, on the Canada C3 expedition, made collections at Tingijattut on the western arm of Kangiqtualuk Uqquqti [Sam Ford Fiord], at Ravenscraig Harbour on the eastern shore of Arviqtujuq Kangiqtua [Eglinton Fiord], and in Clyde River; these 49 specimens are at CAN. On 16 August 2018, Roger Bull made two vascular plant collections (housed at CAN) from Coutts Inlet during an expedition with Students on Ice.

Between 22 July and 22 August 2021, we, accompanied by Clyde River wildlife monitors Jaypiti Inutiq and Leeno Apak, collected vascular plant specimens from 165 sites at 23 main localities across Agguttinni Territorial Park, northwest of Clyde River. Details regarding the fieldwork within the park are summarized in Gillespie et al. (2024). We collected vascular plants at an additional seven sites in Clyde River on 10 and 22 August, within the community and near the airport. In total, we made 850 vascular plant collections from Agguttinni TP and Clyde River; the primary set of these collections is vouchered at CAN, with duplicates at Nunavut Parks and Special Places (“npsp”), ALTA, ALA, CHARS, CORD, MIN, MO, MT, NFLD, NY, QFA, UBC, US, and WIN.

From 4–16 August 2022, botanists Martha Raynolds and Helga Bültmann made 44 collections of vascular plants at a camp near Generator Lake at the southern edge of the Barnes Ice Cap. These specimens are at CAN.

Despite a long history of exploration, collecting, and documenting the vascular flora of east-central Baffin Island, only a small subset of the collection data has been published. Only Taylor (1863), Polunin (1940), and Hainault (1966) provided lists or accounts of species in the flora area. Of these authors, only Polunin (1940) provided vouchers for some species; however, most voucher information was skeletal, giving only a collector’s name, general locality, and sometimes the year, and he rarely cited specific collections. Subsequent authors of regional floras that include this area simply provided dot maps of species distributions, and no specimen details were given (Porsild 1957; Porsild and Cody 1980; Aiken et al. 2007). Substantial effort is needed to match dots with voucher specimens, and the size of the dots makes it difficult—sometimes impossible—to align a dot with a specific voucher collection. It is also unclear whether all of these dots were based on voucher collections; some may have been based on the above literature sources. Saarela et al. (2020b) also noted in their flora of Victoria Island that at least some of Porsild’s dots were based on unvouchered observations.

**Table 1.** Collection localities and sites in east central Baffin Island. Collection sites are ordered alphabetically by main area, main locality, and then site number. For each site, the site number, date(s), collector(s), location (including elevation if recorded), and coordinates are provided. Secondary georeferenced coordinates with estimated uncertainty in m are enclosed in square brackets.

Site	Date	Collectors	Location	Coordinates
Agguttinni Territorial Park				
Arviqtujuq Kangiqtua NW				
18.3	18 Aug 2021	Gillespie et al.	East coast of peninsula on NW side of Arviqtujuq Kangiqtua, opposite Ravenscraig Harbour. Elev. 30 m	70°46'21"N, 69°54'6"W
18.4	18 Aug 2021	Gillespie et al.	East coast of peninsula on NW side of Arviqtujuq Kangiqtua, opposite Ijjuq. Elev. 10 m	70°42'28"N, 70°2'27"W
Atagulisaktalik				
22.1	22 Jul 2021	Gillespie et al.	Atagulisaktalik, 2.2 km W of river mouth on Arviqtujuq Kangiqtua, N side of valley. Elev. 100 m	70°29'15"N, 70°39'0"W
22.2	22 Jul 2021	Gillespie et al.	Atagulisaktalik, 2.2 km W of river mouth on Arviqtujuq Kangiqtua, N side of valley. Elev. 100 m	70°29'16"N, 70°38'54"W
22.3	22 Jul 2021	Gillespie et al.	Atagulisaktalik, 2.3 km W of river mouth on Arviqtujuq Kangiqtua, N side of valley. Elev. 105 m	70°29'20"N, 70°39'18"W
23.1	23 Jul 2021	Gillespie et al.	Atagulisaktalik, 2.0 km WNW of river mouth on Arviqtujuq Kangiqtua, N side of valley. Elev. 125 m	70°29'27"N, 70°38'31"W
23.2	23 Jul 2021	Gillespie et al.	Atagulisaktalik, 2.1 km WNW of river mouth on Arviqtujuq Kangiqtua, N side of valley. Elev. 150 m	70°29'32"N, 70°38'18"W
23.3	23 Jul 2021	Gillespie et al.	Atagulisaktalik, 2.0 km WNW of river mouth on Arviqtujuq Kangiqtua, N side of valley. Elev. 185 m	70°29'35"N, 70°38'4"W
23.4	23 Jul 2021	Gillespie et al.	Atagulisaktalik, 1.8 km WNW of river mouth on Arviqtujuq Kangiqtua, N side of valley. Elev. 130 m	70°29'31"N, 70°37'52"W
23.5	23 Jul 2021	Gillespie et al.	Atagulisaktalik, 0.9 km WNW of river mouth on Arviqtujuq Kangiqtua, N side of valley. Elev. 80 m	70°29'6"N, 70°36'56"W
23.6	23 Jul 2021	Gillespie et al.	Atagulisaktalik, 0.6 km W of river mouth on Arviqtujuq Kangiqtua, N side of river. Elev. 70 m	70°29'1"N, 70°36'32"W
23.7	23 Jul 2021	Gillespie et al.	Atagulisaktalik, 0.4 km W of river mouth on Arviqtujuq Kangiqtua, N side of river. Elev. 40 m	70°28'55"N, 70°36'23"W
23.8	23 Jul 2021	Gillespie et al.	Atagulisaktalik, 0.3 km W of river mouth on Arviqtujuq Kangiqtua, N side of river. Elev. 25 m	70°28'51"N, 70°36'13"W
23.9	23 Jul 2021	Gillespie et al.	Atagulisaktalik, near river mouth at Arviqtujuq Kangiqtua. Elev. 2–5 m	70°28'47"N, 70°35'58"W
23.10	23 Jul 2021	Gillespie et al.	Atagulisaktalik, 0.4 km W of river mouth at Arviqtujuq Kangiqtua, on N side of river. Elev. 40 m	70°28'52"N, 70°36'34"W
23.11	23 Jul 2021	Gillespie et al.	Atagulisaktalik, 0.8 km W of river mouth at Arviqtujuq Kangiqtua, on N side of river. Elev. 55 m	70°28'56"N, 70°37'4"W
23.12	23 Jul 2021	Gillespie et al.	Atagulisaktalik, 1.1 km W of river mouth at Arviqtujuq Kangiqtua, on N side of river. Elev. 60 m	70°29'2"N, 70°37'29"W
23.13	23 Jul 2021	Gillespie et al.	Atagulisaktalik, 2.1 km WNW of river mouth on Arviqtujuq Kangiqtua, N side of valley. Elev. 150 m	70°29'31"N, 70°38'18"W
24.1	24 Jul 2021	Gillespie et al.	Atagulisaktalik, N side of valley opposite East Pioneer Glacier. Elev. 110 m	70°29'21"N, 70°41'56"W
24.2	24 Jul 2021	Gillespie et al.	Atagulisaktalik, N side of valley opposite East Pioneer Glacier. Elev. 110 m	70°29'21"N, 70°42'11"W
24.3	24 Jul 2021	Gillespie et al.	Atagulisaktalik, N side of valley opposite East Pioneer Glacier. Elev. 110 m	70°29'19"N, 70°42'24"W
24.4	24 Jul 2021	Gillespie et al.	Atagulisaktalik, N side of valley opposite East Pioneer Glacier. Elev. 110 m	70°29'18"N, 70°42'39"W
24.5	24 Jul 2021	Gillespie et al.	Atagulisaktalik, N side of valley opposite E end of Revoir Mountain and East Pioneer Glacier. Elev. 140 m	70°29'33"N, 70°44'15"W
24.6	24 Jul 2021	Gillespie et al.	Atagulisaktalik, N side of valley opposite E end of Revoir Mountain and East Pioneer Glacier. Elev. 185 m	70°29'38"N, 70°43'33"W
AT-1	22–26 Jul, Aug 21, 24 1950	Dansereau, Röthlisberger, Wynne-Edwards	Head of Eglinton Fiord	[70.457°N, 70.616°W; ± 1000 m]
AT-2	25 Jul 1950	Dansereau	Head of Eglinton Fiord. Talus à la base de Cockscomb Mt.	[70.487°N, 70.565°W; ± 2500 m]
AT-3	4 Aug 1950	Röthlisberger	Eglinton Fiord, N bank	[70.483°N, 70.600°W; ± 1000 m]
AT-4	21 Aug 1950	Röthlisberger	Head of Eglinton Inlet. Gendarme of the Cockscomb	[70.501°N, 70.536°W; ± 500 m]
AT-5	22 Aug 1950	Röthlisberger	Eglinton Fiord, S bank	[70.457°N, 70.613°W; ± 1000 m]
Clark Fiord				
20.1	20 Aug 2021	Gillespie et al.	Valley on W side of Clark Fiord, opposite Ullainnagaq on Qikiqtaaluk, 2 km NW of river mouth. Elev. 70 m	71°6'0"N, 72°7'7"W
20.2	20 Aug 2021	Gillespie et al.	Valley on W side of Clark Fiord, opposite Ullainnagaq on Qikiqtaaluk, 1.9 km NW of river mouth. Elev. 80 m	71°6'0"N, 72°6'55"W
20.3	20 Aug 2021	Gillespie et al.	Valley on W side of Clark Fiord, opposite Ullainnagaq on Qikiqtaaluk, 1.8 km NW of river mouth,. Elev. 140 m	71°6'2"N, 72°6'33"W
20.4	20 Aug 2021	Gillespie et al.	Valley on W side of Clark Fiord, opposite Ullainnagaq on Qikiqtaaluk, 1.8 km NW of river mouth. Elev. 125 m	71°5'57"N, 72°6'22"W
20.5	20 Aug 2021	Gillespie et al.	Valley on W side of Clark Fiord, opposite Ullainnagaq on Qikiqtaaluk. Elev. 110 m	71°5'54"N, 72°6'22"W
20.6	20 Aug 2021	Gillespie et al.	Valley on W side of Clark Fiord, opposite Ullainnagaq on Qikiqtaaluk, 1.4 km NW of river mouth. Elev. 75 m	71°5'45"N, 72°6'15"W
20.7	20 Aug 2021	Gillespie et al.	Valley on W side of Clark Fiord, opposite Ullainnagaq on Qikiqtaaluk, 1.4 km NW of river mouth. Elev. 90 m	71°5'46"N, 72°6'10"W
20.8	20 Aug 2021	Gillespie et al.	Valley on W side of Clark Fiord, opposite Ullainnagaq on Qikiqtaaluk, 1.1 km NW of river mouth. Elev. 75 m	71°5'37"N, 72°6'1"W
Gee Lake				
31.4	31 Jul 2021	Gillespie et al.	Barnes Plateau, E edge of Barnes Ice Cap, ca. 6 km WNW of Gee Lake. Elev. 530 m	69°52'18"N, 72°27'22"W
GE-1	11 Aug 1950	Dansereau	Gee Lake	[69.870°N, 72.252°W; ± 1000 m]
Generator Lake				
31.5	31 Jul 2021	Gillespie et al.	Barnes Plateau, between Barnes Ice Cap and Generator Lake, ca. 4 km W of N end of Generator Lake. Elev. 470 m	69°39'3"N, 71°49'41"W
31.6	31 Jul 2021	Gillespie et al.	Barnes Plateau, between Barnes Ice Cap and Generator Lake, ca. 4 km W of N end of Generator Lake. Elev. 480 m	69°39'11"N, 71°49'50"W
31.7	31 Jul 2021	Gillespie et al.	Barnes Plateau, between Barnes Ice Cap and Generator Lake, ca. 4 km W of N end of Generator Lake. Elev. 470 m	69°39'5"N, 71°49'32"W
31.8	31 Jul 2021	Gillespie et al.	Barnes Plateau, E shore of Generator Lake, at narrows midway along lake. Elev. 400 m	69°35'7"N, 71°48'49"W
31.9	31 Jul 2021	Gillespie et al.	Barnes Plateau, E shore of Generator Lake, at narrows midway along lake. Elev. 400 m	69°35'8"N, 71°48'36"W
GL-10	5 Aug 2022	Raynolds & Bültmann	Generator Strip, GEN-02. Elev. 490 m	69.6056°N, 71.6972°W
GL-11	5 Aug 2022	Raynolds & Bültmann	Generator Strip, GEN-04. Elev. 490 m	69.6061°N, 71.7066°W
GL-12	6 Aug 2022	Raynolds & Bültmann	Generator Strip, GEN-05. Elev. 480 m	69.6083°N, 71.7114°W
GL-12	6 Aug 2022	Raynolds & Bültmann	Generator Strip, GEN-05. Elev. 480 m	69.6083°N, 71.7114°W
GL-13	6 Aug 2022	Raynolds & Bültmann	Generator Strip, GEN-06. Elev. 470 m	69.6084°N, 71.7119°W
GL-14	7 Aug 2022	Raynolds & Bültmann	Generator Strip, GEN-09. Elev. 400 m	69.6252°N, 71.7374°W
GL-15	16 Aug 2022	Raynolds & Bültmann	Generator Strip, GEN-10. Elev. 390 m	69.6251°N, 71.7364°W
GL-16	8 Aug 2022	Bültmann & Raynolds	Generator Strip, GEN-11. Elev. 410 m	69.6219°N, 71.7198°W
GL-17	16 Aug 2022	Raynolds & Bültmann	Generator Strip, GEN-12. Elev. 400 m	69.6193°N, 71.7231W
GL-18	10 Aug 2022	Raynolds & Bültmann	Generator Strip, GEN-17. Elev. 460 m	69.6126°N, 71.7165°W
GL-19	16 Aug 2022	Raynolds & Bültmann	Generator Strip, GEN-19. Elev. 440 m	69.6173°N, 71.7095°W
GL-20	4 Aug 2022	Raynolds & Bültmann	Generator Strip, GEN-23. Elev. 465 m	69.6164°N, 71.6966°W
GL-21	16 Aug 2022	Raynolds & Bültmann	Generator Strip, GEN-25. Elev. 475 m	69.6105°N, 71.6988°W
GL-22	16 Aug 2022	Raynolds & Bültmann	Generator Strip, GEN-26. Elev. 465 m	69.6112°N, 71.7039°W
Gibbs esker				
20.12	20 Aug 2021	Gillespie et al.	Barnes Plateau, esker E of Erik River canyon, 7.3 km S of mouth of Eric River at head of Gibbs Fiord. Elev. 550 m	70°32'58"N, 72°33'2"W
Gibbs Fiord				
20.9	20 Aug 2021	Gillespie et al.	Tay River valley at head of Gibbs Fiord, kame 3.3 km W of head of fiord. Elev. 150–170 m	70°37'51"N, 72°42'55"W
20.1	20 Aug 2021	Gillespie et al.	Tay River valley at head of Gibbs Fiord, 3.2 km W of head of fiord, NE of kame. Elev. 150 m	70°37'53"N, 72°42'46"W
20.11	20 Aug 2021	Gillespie et al.	Tay River delta at head of Gibbs Fiord. Elev. 5 m	70°38'0"N, 72°37'48"W
GF-1	24 Jul 1950	Dansereau	Gibbs Fiord	[70.615°N, 72.568°W; ± 1500 m]
Kangiqtualuk Agguqti				
3.1	3 Aug 2021	Gillespie et al.	3.2 km W of head of Kangiqtualuk Agguqti, N shore of lake, WNW of Akuliqutaaluk. Elev. 180 m	70°17'31"N, 71°57'8"W
3.2	3 Aug 2021	Gillespie et al.	3.3 km W of head of Kangiqtualuk Agguqti, N side of lake, WNW of Akuliqutaaluk. Elev. 215 m	70°17'43"N, 71°57'16"W
3.3	3 Aug 2021	Gillespie et al.	3.6 km W of head of Kangiqtualuk Agguqti, N side of lake, WNW of Akuliqutaaluk. Elev. 225 m	70°17'43"N, 71°57'52"W
3.4	3 Aug 2021	Gillespie et al.	4.0 km W of head of Kangiqtualuk Agguqti, N of lake, WNW of Akuliqutaaluk. Elev. 240 m	70°17'46"N, 71°58'24"W
3.5	3 Aug 2021	Gillespie et al.	4.0 km W of head of Kangiqtualuk Agguqti, N of lake, WNW of Akuliqutaaluk. Elev. 255 m	70°17'50"N, 71°59'4"W
3.6	3 Aug 2021	Gillespie et al.	3.8 km W of head of Kangiqtualuk Agguqti, N of lake, WNW of Akuliqutaaluk. Elev. 245 m	70°17'53"N, 71°58'11"W
5.1	5 Aug 2021	Gillespie et al.	2.7 km W of head of Kangiqtualuk Agguqti, NE end of lake at outlet, WNW of Akuliqutaaluk. Elev. 185 m	70°17'37"N, 71°56'23"W
5.3	5 Aug 2021	Gillespie et al.	1.9 km W of head of Kangiqtualuk Agguqti, N side of river valley, WNW of Akuliqutaaluk. Elev. 160 m	70°17'32"N, 71°55'4"W
5.4	5 Aug 2021	Gillespie et al.	1.7 km W of head of Kangiqtualuk Agguqti, N side of river valley, WNW of Akuliqutaaluk. Elev. 150 m	70°17'29"N, 71°54'46"W
5.5	5 Aug 2021	Gillespie et al.	3 km W of head of Kangiqtualuk Agguqti, NE end of lake near outlet, WNW of Akuliqutaaluk. Elev. 180 m	70°17'34"N, 71°56'48"W
6.1	6 Aug 2021	Gillespie et al.	0.7 km W of head of Kangiqtualuk Agguqti, N side of river valley, WNW of Akuliqutaaluk. Elev. 140 m	70°17'42"N, 71°53'10"W
6.2	6 Aug 2021	Gillespie et al.	0.5 km W of head of Kangiqtualuk Agguqti, N side of river valley, WNW of Akuliqutaaluk. Elev. 120 m	70°17'45"N, 71°52'49"W
6.3	6 Aug 2021	Gillespie et al.	Head of Kangiqtualuk Agguqti, coast near river mouth, NW of Akuliqutaaluk. Elev. 0–30 m	70°17'42"N, 71°52'3"W
6.4	6 Aug 2021	Gillespie et al.	0.4 km W of head of Kangiqtualuk Agguqti, N side of river, WNW of Akuliqutaaluk. Elev. 55 m	70°17'32"N, 71°52'24"W
6.5	6 Aug 2021	Gillespie et al.	N side of river, 1.1 km W of head of Kangiqtualuk Agguqti, WNW of Akuliqutaaluk. Elev. 100 m	70°17'22"N, 71°53'33"W
6.6	6 Aug 2021	Gillespie et al.	1.2 km W of head of Kangiqtualuk Agguqti, N side of river valley, WNW of Akuliqutaaluk. Elev. 105 m	70°17'33"N, 71°54'60"W
7.1	7 Aug 2021	Gillespie et al.	3.9 km W of head of Kangiqtualuk Agguqti, NW side of lake, W of Akuliqutaaluk. Elev. 245 m	70°17'26"N, 71°58'13"W
7.2	7 Aug 2021	Gillespie et al.	4.9 km W of head of Kangiqtualuk Agguqti, W side of lake, W of Akuliqutaaluk. Elev. 215 m	70°17'3"N, 71°59'28"W
8.1	8 Aug 2021	Gillespie et al.	Delta of large river near head of Kangiqtualuk Agguqti, NE of Akuliqutaaluk. Elev. 4 m	70°18'41"N, 71°45'5"W
8.2	8 Aug 2021	Gillespie et al.	Side valley on E side of large river valley, 5 km S of river mouth near head of Kangiqtualuk Agguqti, E of Akuliqutaaluk. Elev. 30–35 m	70°16'17"N, 71°44'46"W
8.3	8 Aug 2021	Gillespie et al.	East side of large river valley, 5.2 km S of river mouth near head of Kangiqtualuk Agguqti, E of Akuliqutaaluk. Elev. 60 m	70°16'11"N, 71°44'58"W
8.4	8 Aug 2021	Gillespie et al.	East side of large river valley, 5.3 km S of river mouth near head of Kangiqtualuk Agguqti, E of Akuliqutaaluk. Elev. 70 m	70°16'8"N, 71°45'7"W
8.5	8 Aug 2021	Gillespie et al.	East side of large river valley, 5.4 km S of river mouth near head of Kangiqtualuk Agguqti, E of Akuliqutaaluk. Elev. 90 m	70°16'3"N, 71°45'20"W
8.6	8 Aug 2021	Gillespie et al.	West side of large river, 12 km SW of river mouth near head of Kangiqtualuk Agguqti, SW of Akuliqutaaluk. Elev. 80 m	70°13'23"N, 71°55'1"W
8.7	8 Aug 2021	Gillespie et al.	West side of large river, 12.3 km SW of river mouth near head of Kangiqtualuk Agguqti, SW of Akuliqutaaluk. Elev. 110 m	70°13'21"N, 71°55'25"W
8.8	8 Aug 2021	Gillespie et al.	West side of large river, 12.5 km SW of river mouth near head of Kangiqtualuk Agguqti, SW of Akuliqutaaluk. Elev. 100 m	70°13'14"N, 71°55'39"W
8.9	8 Aug 2021	Gillespie et al.	West side of large river, 12.8 km SW of river mouth near head of Kangiqtualuk Agguqti, SW of Akuliqutaaluk. Elev. 90 m	70°13'10"N, 71°55'45"W
8.10	8 Aug 2021	Gillespie et al.	West side of large river, 13.5 km SW of river mouth near head of Kangiqtualuk Agguqti, SW of Akuliqutaaluk. Elev. 90 m	70°12'52"N, 71°56'25"W
21.1	21 Aug 2021	Gillespie et al.	Large river valley at head of Kangiqtualuk Agguqti, on N side of river, 15.5 km SW of river mouth. Elev. 55–60 m	70°12'38"N, 72°1'24"W
21.2	21 Aug 2021	Gillespie et al.	Large river valley, 15.5 km SW of river mouth near head of Kangiqtualuk Agguqti, on N side of river. Elev. 55 m	70°12'39"N, 72°1'29"W
21.3	21 Aug 2021	Gillespie et al.	Large river valley, 15.5 km SW of river mouth near head of Kangiqtualuk Agguqti, on N side of river. Elev. 60 m	70°12'37"N, 72°1'44"W
21.4	21 Aug 2021	Gillespie et al.	Large river valley, 15.5 km SW of river mouth near head of Kangiqtualuk Agguqti, on N side of river. Elev. 75 m	70°12'43"N, 72°1'59"W
21.5	21 Aug 2021	Gillespie et al.	Large river valley, 16.1 km SW of river mouth near head of Kangiqtualuk Agguqti, vicinity of cabin on N side of river. Elev. 60 m	70°12'35"N, 72°2'20"W
21.6	21 Aug 2021	Gillespie et al.	Large river valley, 16.3 km SW of river mouth near head of Kangiqtualuk Agguqti, on N side of river. Elev. 60 m	70°12'30"N, 72°2'52"W
21.7	21 Aug 2021	Gillespie et al.	Large river valley, 16.3 km SW of river mouth near head of Kangiqtualuk Agguqti, on N side of river. Elev. 60 m	70°12'27"N, 72°3'6"W
21.8	21 Aug 2021	Gillespie et al.	Large river valley, 16.3 km SW of river mouth near head of Kangiqtualuk Agguqti, on N side of river. Elev. 60 m	70°12'27"N, 72°3'45"W
21.9	21 Aug 2021	Gillespie et al.	Large river valley, 16.3 km SW of river mouth near head of Kangiqtualuk Agguqti, on N side of river. Elev. 65 m	70°12'28"N, 72°4'38"W
21.10	21 Aug 2021	Gillespie et al.	Large river valley, 16.3 km SW of river mouth near head of Kangiqtualuk Agguqti, on N side of river. Elev. 65 m	70°12'26"N, 72°4'43"W
Kangiqtualuk Uqquqti				
1.1	1 Aug 2021	Gillespie et al.	Sam Ford River valley, 2.3 km W of head of Kangiqtualuk Uqquqti, small valley on NW side of river. Elev. 90 m	70°1'50"N, 71°36'19"W
1.2	1 Aug 2021	Gillespie et al.	Sam Ford River valley, 2.3 km W of head of Kangiqtualuk Uqquqti, small valley on NW side of river. Elev. 55 m	70°1'45"N, 71°36'3"W
27.1	27 Jul 2021	Gillespie et al.	Sam Ford River valley, 3 km W of river mouth at head of Kangiqtualuk Uqquqti, near small lake on NW side of valley. Elev. 125 m	70°1'54"N, 71°37'5"W
28.1	28 Jul 2021	Gillespie et al.	Sam Ford River valley, 3 km W of river mouth at head of Kangiqtualuk Uqquqti, near small lake on NW side of valley. Elev. 125 m	70°1'54"N, 71°37'5"W
28.2	28 Jul 2021	Gillespie et al.	Sam Ford River valley, 3.3 km W of river mouth at head of Kangiqtualuk Uqquqti, NW side of valley. Elev. 135 m	70°1'56"N, 71°37'40"W
28.3	28 Jul 2021	Gillespie et al.	Sam Ford River valley, 3.3 km W of river mouth at head of Kangiqtualuk Uqquqti, NW side of valley. Elev. 140 m	70°1'58"N, 71°37'44"W
28.4	28 Jul 2021	Gillespie et al.	Sam Ford River valley, 3.5 km W of river mouth at head of Kangiqtualuk Uqquqti, NW side of valley. Elev. 150 m	70°1'56"N, 71°38'2"W
28.5	28 Jul 2021	Gillespie et al.	Sam Ford River valley, 3.5 km W of river mouth at head of Kangiqtualuk Uqquqti, NW side of valley. Elev. 135 m	70°1'52"N, 71°38'7"W
28.6	28 Jul 2021	Gillespie et al.	Sam Ford River valley, 3.5 km W of river mouth at head of Kangiqtualuk Uqquqti, NW side of valley. Elev. 140 m	70°1'50"N, 71°38'10"W
28.7	28 Jul 2021	Gillespie et al.	Sam Ford River valley, 3.4 km W of river mouth at head of Kangiqtualuk Uqquqti, NW side of valley. Elev. 110 m	70°1'45"N, 71°37'51"W
29.1	29 Jul 2021	Gillespie et al.	Sam Ford River valley, 2.8 km W of river mouth at head of Kangiqtualuk Uqquqti, NW side of river. Elev. 20 m	70°1'31"N, 71°36'44"W
29.2	29 Jul 2021	Gillespie et al.	Sam Ford River valley, 2.8 km W of river mouth at head of Kangiqtualuk Uqquqti, NW side of river. Elev. 10 m	70°1'28"N, 71°36'39"W
29.3	29 Jul 2021	Gillespie et al.	Sam Ford River valley, 3.0 km W of river mouth at head of Kangiqtualuk Uqquqti, NW side of river. Elev. 20 m	70°1'21"N, 71°36'52"W
29.4	29 Jul 2021	Gillespie et al.	Sam Ford River valley, 3.3 km W of river mouth at head of Kangiqtualuk Uqquqti, NW side of river. Elev. 15 m	70°1'12"N, 71°37'12"W
29.5	29 Jul 2021	Gillespie et al.	Sam Ford River valley, 3.7 km W of river mouth at head of Kangiqtualuk Uqquqti, NW side of river. Elev. 25 m	70°1'3"N, 71°37'38"W
30.1	30 Jul 2021	Gillespie et al.	Sam Ford River valley, 1.5 km W of river mouth at head of Kangiqtualuk Uqquqti, N side of river. Elev. 5 m	70°1'52"N, 71°34'53"W
30.2	30 Jul 2021	Gillespie et al.	Sam Ford River valley, 1.5 km W of river mouth at head of Kangiqtualuk Uqquqti, N side of river. Elev. 5 m	70°1'55"N, 71°34'46"W
30.3	30 Jul 2021	Gillespie et al.	Sam Ford River valley, 1.1 km W of river mouth at head of Kangiqtualuk Uqquqti, N side of river. Elev. 5 m	70°2'8"N, 71°34'7"W
30.4	30 Jul 2021	Gillespie et al.	Sam Ford River valley, 0.9 km W of river mouth at head of Kangiqtualuk Uqquqti, N side of river. Elev. 2 m	70°2'10"N, 71°33'36"W
30.5	30 Jul 2021	Gillespie et al.	Sam Ford River delta at head of Kangiqtualuk Uqquqti. Elev. 0–1 m	70°1'56"N, 71°32'26"W
30.6	30 Jul 2021	Gillespie et al.	Sam Ford River delta, 0.5 km W of head of Kangiqtualuk Uqquqti. Elev. 0–1 m	70°1'60"N, 71°33'2"W
30.7	30 Jul 2021	Gillespie et al.	Sam Ford River delta, 0.6 km W of head of Kangiqtualuk Uqquqti. Elev. 1 m	70°2'0"N, 71°33'10"W
30.8	30 Jul 2021	Gillespie et al.	Sam Ford River delta, 0.9 km W of head of Kangiqtualuk Uqquqti, N side of river. Elev. 1 m	70°2'4"N, 71°33'36"W
30.9	30 Jul 2021	Gillespie et al.	Sam Ford River valley, 1.6 km W of river mouth at head of Kangiqtualuk Uqquqti, N side of river. Elev. 10 m	70°1'55"N, 71°34'55"W
31.10	31 Jul 2021	Gillespie et al.	Large waterfall on Sam Ford River, 9.2 km SW of head of Kangiqtualuk Uqquqti. Elev. 230 m	69°57'50"N, 71°42'17"W
Kuugaaluk				
16.1	16 Aug 2021	Gillespie et al.	Mouth of Kuugaaluk, on W side of river. Elev. 20 m	70°42'20"N, 69°0'41"W
16.2	16 Aug 2021	Gillespie et al.	Mouth of Kuugaaluk, on W side of river. Elev. 20 m	70°42'21"N, 69°0'48"W
16.3	16 Aug 2021	Gillespie et al.	Mouth of Kuugaaluk, on W side of river. Elev. 25 m	70°42'20"N, 69°0'53"W
16.4	16 Aug 2021	Gillespie et al.	Mouth of Kuugaaluk, on W side of river. Elev. 15 m	70°42'23"N, 69°0'49"W
16.5	16 Aug 2021	Gillespie et al.	Mouth of Kuugaaluk, on W side of river. Elev. 1 m	70°42'18"N, 69°0'27"W
16.6	16 Aug 2021	Gillespie et al.	Mouth of Kuugaaluk, on W side of river. Elev. 5 m	70°42'18"N, 69°0'30.0"W
16.7	16 Aug 2021	Gillespie et al.	Mouth of Kuugaaluk, on W side of river. Elev. 4 m	70°42'16"N, 69°0'43"W
16.8	16 Aug 2021	Gillespie et al.	Kame and kettle complex, 4 km SE of mouth of Kuugaaluk. Elev. 70 m	70°40'30"N, 68°56'58"W
19.1	19 Aug 2021	Gillespie et al.	Waterfall on Kuugaaluk, 13.5 km SW of river mouth, W side of river. Elev. 45 m	70°35'59"N, 69°11'14"W
19.2	19 Aug 2021	Gillespie et al.	Waterfall on Kuugaaluk, 13.5 km SW of river mouth, W side of river. Elev. 65 m	70°35'57"N, 69°11'37"W
19.3	19 Aug 2021	Gillespie et al.	Waterfall on Kuugaaluk, 13.5 km SW of river mouth, W side of river. Elev. 60 m	70°36'4"N, 69°11'24"W
19.4	19 Aug 2021	Gillespie et al.	Sand hills on W bank of Kuugaaluk, 7 km SW of river mouth. Elev. 30–40 m	70°39'0"N, 69°6'30"W
19.5	19 Aug 2021	Gillespie et al.	Kame and kettle complex, 4 km SE of mouth of Kuugaaluk. Elev. 70 m	70°40'30"N, 68°56'58"W
A22.2	22 Aug 2021	Gillespie et al.	South shore of lake, 5 km SW of mouth of Kuugaaluk. Elev. 40 m	70°40'47"N, 69°7'30"W
A22.3	22 Aug 2021	Gillespie et al.	Mouth of Kuugaaluk, on W side of river. Elev. 0–2 m	70°42'18"N, 69°0'30"W
Marble Lake				
31.1	31 Jul 2021	Gillespie et al.	South shore of unnamed lake, 14.5 km WNW of head of Kangiqtualuk Uqquqti. Elev. 460 m	70°4'29"N, 71°54'16"W
31.2	31 Jul 2021	Gillespie et al.	South shore of unnamed lake, 14.5 km WNW of head of Kangiqtualuk Uqquqti. Elev. 455 m	70°4'35"N, 71°54'24"W
31.3	31 Jul 2021	Gillespie et al.	South shore of unnamed lake, 14.5 km WNW of head of Kangiqtualuk Uqquqti. Elev. 455 m	70°4'29"N, 71°53'51"W
Niaqurnaaluk-Qassialuit				
18.1	18 Aug 2021	Gillespie et al.	Sand beach between Niaqurnaaluk and Qassialuit, at E end of beach. Elev. 0–1 m	70°46'22"N, 69°21'50"W
18.2	18 Aug 2021	Gillespie et al.	Rocky coast E of long sand beach between Niaqurnaaluk and Qassialuit. Elev. 2–3 m	70°46'29"N, 69°21'32"W
Ravenscraig Harbour				
18.6	18 Aug 2021	Gillespie et al.	Bay on E side of Arviqtujuq Kangiqtua, SW of Ravenscraig Harbour. Elev. 4 m	70°41'21"N, 69°46'44"W
RH-1	8 Aug 2017	Dare	Ravenscraig Harbour on E shore of Arviqtujuq Kangiqtua. Elev. 0-100 m	70°42'10"N, 69°41'52"W
Refuge Harbour				
11.1	11 Aug 2021	Gillespie et al.	Remote Peninsula, large valley NE of Refuge Harbour, 2.5 km NE of head of bay. Elev. 180 m	70°52'59"N, 71°7'36"W
11.2	11 Aug 2021	Gillespie et al.	Remote Peninsula, large valley NE of Refuge Harbour, 2.3 km NE of head of bay. Elev. 180 m	70°52'55"N, 71°7'44"W
11.3	11 Aug 2021	Gillespie et al.	Remote Peninsula, large valley NE of Refuge Harbour, 2.0 km NE of head of bay. Elev. 170 m	70°52'48"N, 71°7'50"W
11.4	11 Aug 2021	Gillespie et al.	Remote Peninsula, large valley NE of Refuge Harbour, 2.2 km NE of head of bay. Elev. 175 m	70°52'51"N, 71°7'37"W
12.1	12 Aug 2021	Gillespie et al.	Remote Peninsula, large valley NE of Refuge Harbour, 1.9 km NE of head of bay. Elev. 195 m	70°52'38"N, 71°7'54"W
12.2	12 Aug 2021	Gillespie et al.	Remote Peninsula, large valley NE of Refuge Harbour, 1.0 km NE of head of bay. Elev. 125 m	70°52'14"N, 71°8'53"W
12.3	12 Aug 2021	Gillespie et al.	Remote Peninsula, large valley NE of Refuge Harbour, 0.3 km NE of head of bay. Elev. 40 m	70°52'4"N, 71°10'3"W
12.4	12 Aug 2021	Gillespie et al.	Remote Peninsula, Refuge Harbour, NE shore at head of bay. Elev. 3 m	70°51'57"N, 71°10'24"W
12.5	12 Aug 2021	Gillespie et al.	Remote Peninsula, large valley NE of Refuge Harbour, 0.3 km E of head of bay. Elev. 10–15 m	70°51'46"N, 71°10'13"W
12.6	12 Aug 2021	Gillespie et al.	Remote Peninsula, large valley NE of Refuge Harbour, 0.3 km E of head of bay. Elev. 10–15 m	70°51'49"N, 71°10'9"W
12.7	12 Aug 2021	Gillespie et al.	Remote Peninsula, large valley NE of Refuge Harbour, 0.3 km NE of head of bay. Elev. 45 m	70°52'0"N, 71°9'55"W
12.8	12 Aug 2021	Gillespie et al.	Remote Peninsula, large valley NE of Refuge Harbour, 0.8 km NE of head of bay. Elev. 90 m	70°52'14"N, 71°9'29"W
14.1	14 Aug 2021	Gillespie et al.	Remote Peninsula, large valley NE of Refuge Harbour, 2.2 km NE of head of bay. Elev. 185 m	70°52'43"N, 71°7'27"W
14.2	14 Aug 2021	Gillespie et al.	Remote Peninsula, large valley NE of Refuge Harbour, 2.2 km NE of head of bay. Elev. 195 m	70°52'40"N, 71°7'17"W
14.4	14 Aug 2021	Gillespie et al.	Remote Peninsula, large valley NE of Refuge Harbour, 2.6 km NE of head of bay. Elev. 220 m	70°52'37"N, 71°6'30"W
Remote Lake				
RL-1	10 Aug 1966	Philpot et al.	Remote Lake, Remote Peninsula, Elev. 0–75 m	[71.0008°N, 70.6433°W; ± 1500 m]
Scott Inlet				
SI-1	1856–1861	Taylor	Scott Inlet, Davis Strait	[71.1166°N, 71.1°W; ± 10000 m]
Stewart Valley				
15.1	15 Aug 2021	Gillespie et al.	Stewart Valley, midway along valley, on W side opposite glacier and moraine below Tiiturvik and Pana peaks. Elev. 100–130 m	70°45'13"N, 71°23'46"W
Swiss Bay				
SB-1	19 Jul 1950	Röthlisberger	Ritchie Fiord: M.1	[70.5583°N, 71.0417°W; ± 1000 m]
SB-2	11 Aug 2008	Starr	Sam Ford Fiord. Glacial valley on E side of Kigut Peak. Elev. 180 m	70°33'41"N, 70°59'57"W
Tasialuk N				
26.1	26 Jul 2021	Gillespie et al.	Tasialuk, NE end of lake, S of Kuugaaluk. Elev. 70 m	70°30'20"N, 69°34'12"W
26.2	26 Jul 2021	Gillespie et al.	Tasialuk, NE end of lake, S of Kuugaaluk. Elev. 70 m	70°30'24"N, 69°34'16"W
26.3	26 Jul 2021	Gillespie et al.	Tasialuk, NE end of lake, S of Kuugaaluk. Elev. 80 m	70°30'28"N, 69°34'31"W
26.4	26 Jul 2021	Gillespie et al.	Kuugaaluk valley, 7 km E of NE end of Tasialuk, S of river. Elev. 170 m	70°29'42"N, 69°22'37"W
Tasialuk S				
21.12	21 Aug 2021	Gillespie et al.	Sand hills on E side of large valley at S end of Tasialuk, 13.6 km S of unnamed lake. Elev. 220–230 m	70°4'46"N, 70°46'49"W
26.5	26 Jul 2021	Gillespie et al.	Large valley at S end of Tasialuk, 13.9 km S of unnamed lake, E side of valley. Elev. 120 m	70°4'50"N, 70°48'50"W
26.6	26 Jul 2021	Gillespie et al.	Large valley at S end of Tasialuk, 6.1 km S of unnamed lake, W side of valley. Elev. 90 m	70°8'45"N, 70°44'2"W
Tingijattut				
15.2	15 Aug 2021	Gillespie et al.	Valley at S end of Tingijattut on NW corner of Kangiqtualuk Agguqti, 1.8 km from coast, at N end of lake. Elev. 130 m	70°35'6"N, 71°38'9"W
15.3	15 Aug 2021	Gillespie et al.	Valley at S end of Tingijattut on NW corner of Kangiqtualuk Agguqti, 1.7 km from coast, near N end of lake. Elev. 140 m	70°35'8"N, 71°38'7"W
15.4	15 Aug 2021	Gillespie et al.	Valley at S end of Tingijattut on NW corner of Kangiqtualuk Agguqti, 1.7 km from coast, near N end of lake. Elev. 140 m	70°35'9"N, 71°38'5"W
15.5	15 Aug 2021	Gillespie et al.	Valley at S end of Tingijattut on NW corner of Kangiqtualuk Agguqti, 1.3 km from coast. Elev. 100 m	70°35'21"N, 71°37'41"W
15.6	15 Aug 2021	Gillespie et al.	Valley at S end of Tingijattut on NW corner of Kangiqtualuk Agguqti, 1.2 km from coast. Elev. 70 m	70°35'23"N, 71°37'29"W
15.7	15 Aug 2021	Gillespie et al.	Valley at S end of Tingijattut on NW corner of Kangiqtualuk Agguqti, 1.0 km from coast. Elev. 60 m	70°35'26"N, 71°37'17"W
15.8	15 Aug 2021	Gillespie et al.	Valley at S end of Tingijattut on NW corner of Kangiqtualuk Agguqti, 0.3 km from coast. Elev. 15 m	70°35'40"N, 71°36'15"W
15.9	15 Aug 2021	Gillespie et al.	Coast on N side of river mouth at S end of Tingijattut on NW corner of Kangiqtualuk Agguqti. Elev. 2 m	70°35'40"N, 71°35'59"W
15.10	15 Aug 2021	Gillespie et al.	Coast N of river mouth at S end of Tingijattut on NW corner of Kangiqtualuk Agguqti. Elev. 5 m	70°35'53"N, 71°36'2"W
TG-2	10 Aug 2017	Dare et al.	Remote Peninsula, Tingijattut, at W arm of Kangiqtualuk Uqquqti. Elev. 0–50 m	70°35'41"N, 71°35'59"W
Clyde Area				
Cape Hewett				
CH-1	18 Aug 1948	Platt	Cape Hewett	[70.2544°N, 67.7893°W; ± 200 m]
Clyde River				
10.1	10 Aug 2021	Gillespie et al.	Kanngiqtugaapik, S edge of hamlet near coast by Qammaq Hotel	70°28'15.0"N, 68°35'17.3"W
10.2	10 Aug 2021	Gillespie et al.	Kanngiqtugaapik, S edge of hamlet along coast, near community fuel tanks and docks	70°28'8.0"N, 68°35'39.3"W
10.3	10 Aug 2021	Gillespie et al.	Kanngiqtugaapik, E edge of hamlet, between community fuel tanks near coast and road to community dump	70°28'14.2"N, 68°36'21.8"W
10.4	10 Aug 2021	Gillespie et al.	Kanngiqtugaapik, E edge of hamlet along road to community dump	70°28'22.6"N, 68°36'23.9"W
A22.4	22 Aug 2021	Gillespie et al.	Kanngiqtugaapik, vicinity of airport	70°29'9.3"N, 68°30'45.8"W
A22.5	22 Aug 2021	Gillespie et al.	Kanngiqtugaapik, on SE side of hamlet, tundra near gas station and community docks	70°28'10.8"N, 68°36'0.1"W
A22.6	22 Aug 2021	Gillespie et al.	Kanngiqtugaapik, behind Qammaq Hotel, between hotel and beach	70°28'16.3"N, 68°35'17.8"W
CR-1	6 Sep 1909; 18 Aug 1927; 9 Sep 1934; 9–15 Sep 1936; 5 Sep 1938; 7 Aug 1939; 21 Sep 1941; 30 Jul 1950; 29 Jun–13 Aug 1958	McMillan, Malte, Polunin, Dutilly, Sanson, Oughton, Dansereau, Martin	Clyde River	[70.4651°N, 68.5535°W; ± 5000 m]
CR-2	19 Jun–25 Aug 1988; 27 Jun–13 Aug 1989; 22 Jun–12 Jul 1990	Forbes	Old Clyde River Settlement	[70.4651°N, 68.5535°W; ± 500 m]
CR-3	9 Aug 2017	Dare	Clyde River	70.4768°N, 68.5689°W
CR-4	30 Jul 1950	Dansereau	Clyde, Agnes Monument [Umiujaq]	[70.5105°N, 68.2012°W; ± 200 m]
CR-5	6 Aug 1958	Martin	Islands in Clyde Inlet	[70.295°N, 68.523°W; ± 18000 m]
Hewett Cache				
HC-1		Hainault	Hewett Cache, 25 km SW of Cape Hewett	[70.0729°N, 67.4009°W; ± 1000 m]
Hoffman Cove				
HF-1	16 Aug 1938	Bartlett	“Hoffman Cove”	69°35'N, 67°25'W
Inugsuin Head				
IG-1	25 Jun–24 Aug 1965; 6 Aug 1966	Hainault, Crompton et al.	Head of Inugsuin Fiord	[69.6244°N, 70.0194°W; ± 5000 m]
IG-2	25 Jun–26 Jul 1965	Hainault	Inugsuin Fiord, base camp	[69.6244°N, 70.0194°W; ± 500 m]
IG-3	1–2 Aug 1967	Parmelee & Seaborn	Inugsuin Fiord, Geological Survey Site	[69.6244°N, 70.0194°W; ± 500 m]
IG-4	1 Aug 1967	Parmelee & Seaborn	Geological Survey Site, Inugsuin Fiord, 1 mile N of Inugsuin site	[69.6391°N, 70.0199°W; ± 1000 m]
IG-5	30–31 Jul 1967	Parmelee & Seaborn	Geological Survey Site, Inugsuin Fiord, 1.5 miles N of Inugsuin site	[69.6461°N, 70.0199°W; ± 1000 m]
IG-6	25 Jul 1967	Parmelee & Seaborn	Geological Survey Site, Inugsuin Fiord, 4 miles NE of Inugsuin site	[69.6671°N, 69.9066°W; ± 2500 m]
IG-7	28 Jul 1967	Parmelee & Seaborn	Geological Survey Site, Inugsuin Fiord, 2 miles E of Inugsuin site	[69.6212°N, 69.9355°W; ± 1300 m]
IG-8	29 Jul 1967	Parmelee & Seaborn	Geological Survey Site, Inugsuin Fiord, 0.5 miles S of Inugsuin site	[69.6178°N, 70.0195°W; ± 1200 m]
IG-9	29–31 July 1967	Parmelee & Seaborn	Geological Survey Site, Inugsuin Fiord, 1 mile S of Inugsuin site	[69.6102°N, 70.0202°W; ± 800 m]
IG-10	30 Jul 1967	Parmelee & Seaborn	Geological Survey Site, Inugsuin Fiord, 1.5 miles S of Inugsuin site	[69.6027°N, 70.0197°W; ± 1000 m]
IG-11	31 Jul 1967	Parmelee & Seaborn	Geological Survey Site, Inugsuin Fiord, 1.5 miles SW of Inugsuin site	[69.609°N, 70.0633°W; ± 1000 m]
IG-12	30 Jul 1967	Parmelee & Seaborn	Geological Survey Site, Inugsuin Fiord, 2 miles SW of Inugsuin site	[69.6028°N, 70.0748°W; ± 1600 m]
IG-13	27 Jul 1967	Parmelee & Seaborn	Geological Survey Site, Inugsuin Fiord, 1 mile W of Inugsuin site	[69.6255°N, 70.0603°W; ± 800 m]
IG-14	27 Jul 1967	Parmelee & Seaborn	Geological Survey Site, Inugsuin Fiord, 1.5 miles W of Inugsuin site	[69.62°N, 70.096°W; ± 1000 m]
IG-15	25–26 Jul 1967	Parmelee & Seaborn	Geological Survey Site, Inugsuin Fiord, Koodloo Lake, 2.5 miles W of Inugsuin site	[69.6248°N, 70.1222°W; ± 1600 m]
IG-16	2–5 Jul 1965	Hainault	Inugsuin Fiord, 1 km N of base camp	[69.6333°N, 70.0201°W; ± 400 m]
IG-17	6 Jul 1965	Hainault	Inugsuin Fiord, 1.5 km N of base camp	[69.6381°N, 70.0201°W; ± 600 m]
IG-18	17 Aug 1965	Hainault	Inugsuin Fiord, 2 km N of base camp	[69.6425°N, 70.0181°W; ± 800 m]
IG-19	24 Aug 1965	Hainault	Inugsuin Fiord, 1 km S of base camp	[69.6155°N, 70.0197°W; ± 400 m]
IG-20	19 Jun–18 Aug 1965	Hainault	Inugsuin Fiord, 1 km W of base camp	[69.6241°N, 70.0457°W; ± 400 m]
IG-21	7 Jul–21 Aug 1965	Hainault	Inugsuin Fiord, 2 km W of base camp	[69.6248°N, 70.0709°W; ± 800 m]
IG-22	21 Aug 1965	Hainault	Inugsuin Fiord, 2.5 km W of base camp	[69.6228°N, 70.084°W; ± 1000 m]
IG-23	21 Aug 1965	Hainault	Inugsuin Fiord, 3.5 km W of base camp	[69.6215°N, 70.1084°W; ± 1200 m]
IG-24	26 Jun–5 Jul 1965	Hainault	Inugsuin Fiord, 4 km W of base camp	[69.6252°N, 70.1216°W; ± 1600 m]
IG-25	18–19 Aug 1965	Hainault	Inugsuin Fiord, 6 km W of base camp	[69.6243°N, 70.1732°W; ± 2400 m]
IG-26	30 Jun–19 Aug 1965	Hainault	Inugsuin Fiord, 2 km NW of base camp	[69.6384°N, 70.0517°W; ± 800 m]
IG-27	19 Aug 1965	Hainault	Inugsuin Fiord, 2.5 km NW of base camp	[69.6416°N, 70.061°W; ± 1000 m]
IG-28	29 Jun 1965	Hainault	Inugsuin Fiord, 3 km W of head of the fiord (N side of Inugsuin valley)	[69.6203°N, 70.0953°W; ± 1200 m]
IG-29	19 Aug 1965	Hainault	Inugsuin Fiord, 3 km W of base camp	[69.6203°N, 70.0953°W; ± 1200 m]
IG-30	26 Jun–21 Aug 1965	Hainault	Inugsuin Fiord, 5 km W of head of the fiord	[69.6247°N, 70.1478°W; ± 2000 m]
Inugsuin Mouth				
IM-1	2 Jul-8 Aug 1965	Hainault	S side of Inugsuin Fjord, at 70° Lat.	[70.1406°N, 68.1965°W; ± 3000 m]
Kangiqtugaapik Head				
KP-1	31 May–30 Aug 1950	Dansereau, Wynne-Edwards	Head of Clyde Inlet	[69.8513°N, 70.4906°W; ± 4000 m]
KP-2	7 Aug 1958	Martin	Head of Clyde Inlet	[69.8513°N, 70.4906°W; ± 2000 m]
KP-3	7 Jun–14 Jul 1950	Dansereau, Wynne-Edwards	Head of Clyde Inlet, Camp B	[69.8513°N, 70.4906°W; ± 500 m]
KP-4	22 Jun–11 Jul 1950	Dansereau, Wynne-Edwards	Head of Clyde Inlet, Camp B2	[69.8518°N, 70.5146°W; ± 500 m]
KP-5	14 Jun–28 Aug1950	Dansereau, Wynne-Edwards	Head of Clyde Inlet, “Falcon Hollow”	[69.8524°N, 70.5183°W; ± 500 m]
KP-6	30 Jun–4 Aug1950	Dansereau, Wynne-Edwards	Head of Clyde Inlet, “Falcon Creek” and “upper Falcon Valley”	[69.8482°N, 70.5476°W; ± 2000 m]
KP-7	9 Jul–1 Aug 1950	Dansereau	Head of Clyde Inlet, “Falcon Ridge E”	[69.8536°N, 70.508°W; ± 1000 m]
KP-8	29 Jun–23 Aug 1950	Dansereau, Wynne-Edwards	Head of Clyde Inlet, “Falcon Mountain”, “Falcon’s Cliff”, and “Gyrfalcon’s Nest”	[69.8477°N, 70.5175°W; ± 2000 m]
KP-9	8 Jul–5 Aug 1950	Dansereau, Wynne-Edwards	Head of Clyde Inlet, “Falcon Pond”	[69.8458°N, 70.5345°W; ± 250 m]
KP-10	18 Jul–27 Aug 1950	Dansereau, Wynne-Edwards	Head of Clyde Inlet, W end of “Falcon Ridge”	[69.8349°N, 70.5393°W; ± 1000 m]
KP-11	26 Jul–3 Aug 1950	Dansereau, Wynne-Edwards	Head of Clyde Inlet, “Falcon River” and “Falcon River outwash”	[69.8532°N, 70.4873°W; ± 1000 m]
KP-12	12 Jul 1950	Wynne-Edwards	Head of Clyde Inlet, mouth of Clyde River	[69.8535°N, 70.455°W; ± 1000 m]
KP-13	3 Jul–8 Aug 1950	Dansereau, Wynne-Edwards	Head of Clyde Inlet, margin of Clyde River	[69.8456°N, 70.4849°W; ± 1000 m]
KP-14	12 Jul 1950	Röthlisberger	Valley of Clyde River, about 2 km above mouth in Clyde Fiord	[69.8419°N, 70.4952°W; ± 500 m]
KP-15	13–18 Jul 1950	Dansereau	Head of Clyde Inlet, terrasse en face de “Graveyard”	[69.8373°N, 70.5083°W; ± 600 m]
KP-16	2 Jul–2 Aug 1950	Dansereau, Wynne-Edwards	Head of Clyde Inlet, “Kranck Lake”	[69.828°N, 70.5449°W; ± 700 m]
KP-17	23 Jun–29 Jul 1950	Dansereau, Wynne-Edwards	Head of Clyde Inlet, below “Marble Canyon”	[69.8103°N, 70.5771°W; ± 3000 m]
KP-18	1 Jun–28 Jul 1950	Dansereau, Wynne-Edwards	Head of Clyde Inlet, “Marble Canyon” and Clyde River gorge	[69.8162°N, 70.6304°W; ± 3000 m]
KP-19	31 Jul 1950	Dansereau	Head of Clyde Inlet, à l’extrémité E du “col Falcon, Caribou”	[69.8655°N, 70.4904°W; ± 1000 m]
KP-20	30 Jun–31 Jul 1950	Dansereau	Head of Clyde Inlet, “Dyke Mountain” and “Dyke Cliff”	[69.8663°N, 70.523°W; ± 1500 m]
KP-21	11 Jun–30 Jul 1950	Dansereau	Head of Clyde Inlet, “Hale Hills” and “Hale Cliffs”	[69.863°N, 70.4621°W; ± 800 m]
KP-22	3–31 Jul 1950	Dansereau, Wynne-Edwards	Head of Clyde Inlet, mouth of “Caribou River”	[69.861°N, 70.4348°W; ± 500 m]
KP-23	31 Jul 1950	Wynne-Edwards	Head of Clyde Inlet, beyond mouth of “Caribou River”	[69.8672°N, 70.4184°W; ± 500 m]
KP-24	14 Jun–21 Jul 1950	Wynne-Edwards	Head of Clyde Inlet, “Caribou Valley”	[69.8658°N, 70.4497°W; ± 1000 m]
KP-25	30–31 Jul 1950	Dansereau, Wynne-Edwards	Head of Clyde Inlet, “Caribou Falls”	[69.8746°N, 70.4731°W; ± 1000 m]
KP-26	13 Jun–31 Jul 1950	Dansereau, Wynne-Edwards	Head of Clyde Inlet, “Dryopteris Mountain” and “Dryopteris Garden”	[69.8787°N, 70.4355°W; ± 1000 m]
KP-27	7–16 Jul 1950	Dansereau, Wynne-Edwards	Head of Clyde Inlet, “Pipit Lakes” and “Pipit Hills”	[69.8782°N, 70.3928°W; ± 1000 m]
KP-28	6 Jun–31 Jul 1950	Dansereau, Wynne-Edwards	Head of Clyde Inlet, “Pipit Point”	[69.8651°N, 70.3772°W; ± 1000 m]
McBeth Valley				
MB-1	15–27 Jul 1965	Hainault	Inugsuin Fiord. McBeth Valley, 30 km W of the head of the fiord	[69.4384°N, 70.7027°W; ± 5000 m]
Coutts Inlet				
Cootts Mouth				
CO-1	14 Aug 1948	Coombs	Coutts Inlet [mouth/lower Coutts Inlet]	[72.175°N, 74.933°W; ± 10000 m]
CO-2	16 Aug 2017	Bull	Coutts Inlet, tundra above zodiac landing area	72.1682°N, 75.1087°W
Cootts Fiord				
CO-3	8–12 Aug 1948	Coombs	“Manning Fiord” [upper Coutts Inlet]	71.85°N, 75.5°W
Home Bay				
Cape Hooper				
HO-1	30–31 Aug 1999	Elven, Hammar, Molau	Cape Hooper, Tanner Bay	68.4333°N, 66.8167°W
HO-2	5 Aug 1983	Sadler	Cape Hooper	68.4719°N, 66.8166°W
HO-3	21–22 Jul 1967	Parmelee & Seaborn	DEW Line Site: Fox 4 (Cape Hooper)	[68.4614°N, 66.8389°W; ± 1500 m]
Ekalugad FOX-C				
FC-1	29 Jun–4 Jul 1966; 7, 16–17 Jul 1967	Crompton et al., Philpot et al., Richardson & Webber	Ekalugad Fiord, Fox Charlie Camp	[68.7307°N, 68.589°W; ± 3000 m]
FC-2	21–23 Jul 1993	Oswald	Ekalugad Fiord, FOX-C DEW line station and beach below station	68.7416°N, 68.6041°W
FC-3	21 Jul 1993	Oswald	Ekalugad Fiord, ~8.7 nautical miles W of FOX-C DEW line at edge of riverbed	68.7307°N, 69.000°W
FC-4	23 Jul 1993	Oswald	Ekalugad Fiord, ~7.2 nautical miles E of FOX-C DEW line station, on coast near fiord	68.7166°N, 68.2166°W
FC-5	23 Jul 1993	Oswald	Ekalugad Fiord, ~7.2 nautical miles E of FOX-C DEW line station, on plateau below rocky slopes and above beach	68.7000°N, 68.2166°W
FC-6	23 Jul 1993	Oswald	Ekalugad Fiord, ~3.9 nautical miles E of FOX-C DEW line station, ~1/3 of the distance to Florence Point	68.7166°N, 68.383°W
Ekalugad Head				
EK-1	13 Jul–2 Aug 1966; 17 Jun–23 Jul 1967	Church, Crompton et al., Philpot et al., Ryder, Stock	Ekalugad Fiord, Venturi Bay and locations T1, T2, and T3	[68.8667°N, 69.4497°W; ± 3000 m]
EK-2	22 Aug 1967	Ryder, Stock	Ekalugad Fiord, “Middle Valley”	[68.8703°N, 69.5971°W; ± 2000 m]
EK-3	6–11 Jul 1966	Crompton et al., Philpot et al.	Ekalugad Fiord, “South Ekalugad Valley”	[68.846°N, 69.5613°W; ± 2000 m]
EK-4	23–27 Jul 1966	Crompton et al., Philpot et al.	Ekalugad Fiord, “Bonny Bay”	[68.8798°N, 69.0369°W; ± 4000 m]
Kangirlugag Fiord				
KG-1	10 Jul–8 Aug 1967	Webber	“Kangerdluak” Fiord, “Botany Bay”	[68.8508°N, 68.6209°W; ± 2000 m]
Kangok Fiord				
KO-1	17–28 Jun 1966	Crompton et al., Philpot et al.	Kangok Fiord	[68.614°N, 68.9379°W; ± 1000 m]
Nudlung Fiord				
NF-1	30 Jul 1967	Ryder	Nudlung Valley	[68.25°N, 67.75°W; ± 10000 m]
Rocknoser Fiord				
RF-1	30 Jun–4 Aug 1961; 20 Jul 1967	Smith, Webber	Rocknoser Fiord, “Home Bay” and “Kangigfik Fiord”	[68.9018°N, 68.524°W; ± 5000 m]

## ﻿﻿Methods

### ﻿﻿Herbarium research and data curation

Complementing our fieldwork in Agguttinni Territorial Park and Clyde River described above, we searched for existing specimens from the study area online and examined Arctic specimens in various herbaria. We manually searched collections at the National Herbarium of Canada at the Canadian Museum of Nature(CAN), the National Collection of Vascular Plants at Agriculture and Agri-Food Canada(DAO), the Marie-Victorin Herbarium at the Université de Montréal(MT), the Bell Museum Herbarium at the University of Minnesota (MIN), the Green Plant Herbarium at the Royal Ontario Museum(TRT), the Fowler Herbarium at Queen’s University(QK), and the monocot collections at the Finnish Museum of Natural History (H). We also manually searched a complete set of images of University of Oslo (O) Canadian Arctic specimens received from O in 2008. The recently completed full digitization of Arctic vascular plants at CAN greatly assisted our search of CAN specimens. Our study also benefited from the recent processing at CAN of several backlog collections, as described in Gillespie et al. (2024).

Through the Global Biodiversity Information Facility (GBIF), we conducted various queries to locate additional specimens from the study area in other herbaria. These included two bounding-box searches for georeferenced plant specimens from the area (GBIF.org [30 Nov 2021] GBIF Occurrence Download https://doi.org/10.15468/dl.5rx9mr; GBIF.org [18 Mar 2024] GBIF Occurrence Download https://doi.org/10.15468/dl.ppa2at), two searches of non-georeferenced plant specimens using locality text (GBIF.org [2 Dec 2021] GBIF Occurrence Download https://doi.org/10.15468/dl.bkyhur; GBIF.org [29 Jan 2023a] GBIF Occurrence Download https://doi.org/10.15468/dl.j888bp), targeted searches for plants collected by Hans Röthlisberger (GBIF.org [29 Jan 2023b] GBIF Occurrence Download https://doi.org/10.15468/dl.k6ccd5) and V.C. Wynne-Edwards (GBIF.org [29 Jan 2023c] GBIF Occurrence Download https://doi.org/10.15468/dl.av2hwm), all non-georeferenced plant specimens from Canada collected in July 1950 (GBIF.org [29 Jan 2023d] GBIF Occurrence Download https://doi.org/10.15468/dl.92czcp), and all Canadian specimens at the University of Colorado Herbarium (GBIF.org [30 Jan 2023] GBIF Occurrence Download https://doi.org/10.15468/dl.49avyj). All records from the latter two searches were manually examined to locate digital specimen records from east-central Baffin Island.

This digital search uncovered specimens at an additional 32 herbaria: the E.C. Smith Herbarium at Acadia University(ACAD), the Herbarium at the University of Alaska Museum of the North(ALA), the Herbarium at the University of Alberta(ALTA), the Deaver Herbarium at Northern Arizona University(ASC), the B.A. Bennett Herbarium in the Yukon (BABY), Institut Botànic de Barcelona(BC), Herbarium Pacificum at the Bishop Museum(BISH), Meise Botanic Garden(BR), Carnegie Museum of Natural History(CM), University of Colorado Museum of Natural History(COLO), Royal Botanic Garden Edinburgh (E), Field Museum of Natural History (F), the Gray Herbarium at Harvard University(GH), Naturalis Biodiversity Center (L), the Shirley C. Tucker Herbarium at Louisiana State University(LSU), the National Herbarium of Victoria at the Royal Botanic Gardens Melbourne(MEL), the University of Michigan(MICH), Missouri Botanical Garden(MO), the William and Lynda Steere Herbarium at the New York Botanical Garden(NY), O, the Chinese National Herbarium at the Institute of Botany, Chinese Academy of Sciences(PE), the Herbier Louis-Marie at the Université Laval(QFA), the Swedish Museum of Natural History (S), the Norwegian University of Science and Technology(TRH), The Arctic University Museum of Norway(TROM), the University of Arkansas at Monticello(UAM), the University of British Columbia Herbarium at the Beaty Biodiversity Museum(UBC), the United States National Herbarium at the Smithsonian Institution(US), the Intermountain Herbarium at Utah State University(UTC), the Royal British Columbia Museum (V), Naturhistorisches Museum Wien (W), and the Herbarium of the University of Zürich (Z).

Most specimens collected prior to 2012 either lack coordinates for their collection locations or have general, often imprecise, coordinates. Where possible, we georeferenced these collection sites using the point-radius method or assigned coordinates based on literature sources; the rationales for each site are provided in Suppl. material 2. We assigned uncertainties for all secondary georeferenced coordinates based on the precision of locality information and the terrain accessible for collecting.

The three largest historical collections required extensive georeferencing: Dansereau and Wynne-Edwards’s 1950 specimens from the head of Kangiqtugaapik [Clyde Inlet], Hainault’s 1965 collections, and Parmelee and Seaborn’s 1967 collections from the head of Inugsuin Fiord.

Dansereau’s 1950 specimens bear pre-printed labels with a general collection location circled (i.e., “Clyde” or “Head of Clyde Inlet”) and usually a short, typed location description, often with an unofficial place name. These locations were also used on Wynne-Edwards’s labels, which were typed in full. A hand-drawn map included in Dansereau’s field notes stored at MT indicates the location of many of these features. Comparing this map to ESRI satellite imagery in QGIS 3.28 (https://qgis.org/) allowed us to georeference most of these specimens (Suppl. material 3).

The historical collections from the head of Inugsuin Fiord either bear no coordinates (Hainault) or general coordinates for the fiord location (Parmelee and Seaborn), but most also include a locality description indicating a collection location with a bearing and distance (in either kilometers or miles) from the head of Inugsuin Fiord. These locality descriptions formed the basis for our georeferencing of these collections (see Suppl. material 2).

The first townsite of Clyde River developed around the Hudson Bay Post established in 1924 on the east side of Patricia Bay. The hamlet was relocated to the northwest shore of Patricia Bay at the mouth of Clyde River in 1970–73 (Forbes 1992, 1993). Pre-1970 Clyde River collection localities (often labeled simply “Clyde” on many historic collections) have been georeferenced to the historic townsite on the east side of Patricia Bay, whereas later collections are georeferenced to the current hamlet location. For vague historical collection localities, such as Clyde River (pre-1950), we applied a standard 5 km radius uncertainty based on the likely distance collectors may have traveled in a day by foot from a known base camp or specific site (minimum 10 km return).

Several collection localities appeared to have inaccurate or very imprecise coordinates. The coordinates (68°15'N, 68°45'W) on Ryder’s Nudlung Valley collections indicate a barren upland area at ca. 500 m elevation about 15 km ENE of the upper reaches of the river valley extending from the head of Nudlung Fiord. In addition to being an unlikely collecting locality, this set of plants is not consistent with this elevation and habitat. We assume that the coordinates are incorrect. Coordinates of 68°15'N, 67°45'W would place the locality between the heads of the two arms of Nudlung Fiord, closest to the southeast arm. We tentatively georeferenced the locality here with a 10 km radius uncertainty that encompasses the entire valley of the southeast arm and the lower reaches of the larger valley at the head of the northwest arm. Coombs’s Coutts Inlet locality has primary coordinates that lie in Baffin Bay about 7 km from the nearest land, which is an unnamed island in the mouth of Coutts Inlet. We re-georeferenced this locality to the southeast tip of that island, with an uncertainty of 10 km encompassing the island, mouth, and adjacent land. Coordinates for Coombs’s Manning Fiord locality appear to be equally imprecise; lacking any additional information, we retained the primary coordinates (which lie in a barren mountainous area surrounded by glaciers, about 4 km from an arm of Coutts Inlet) and recognized that the uncertainty would be at least equally large. Primary coordinates for Bartlett’s “Hoffman Cove” locality (an unofficial name) place the locality on a large island in the mouth of Isabella Bay, whereas expedition notes indicate a location near Cape Raper on the north side of the mouth (Nutt 1938); we assigned coordinates based on the latter location.

Georeferencing of several vague localities is described below. Collections by Smith from “Home Bay” are considered here to be collected at or in the vicinity of the head of Rocknoser Fiord, based on where Snell (1991) argued Smith likely spent most of his time. This locality is likely correct for most, but perhaps not all, specimens, and it should be noted there remains considerable uncertainty as to exactly where these specimens were collected. Martin’s “islands in Clyde Inlet” collection locality most likely refers to a group of islands in the mouth and lowermost reaches of Kangiqtugaapik [Clyde Inlet], scattered over a distance of 36 km (rather than two islands near the head of Kangiqtugaapik). We georeferenced this locality on the largest island in the center of this area with an uncertainty of 18 km encompassing all the islands in the mouth of Clyde Inlet, plus many of the islands at the mouth of Inugsuin Fiord.

With few exceptions, we were not able to study the only known 19^th^-century collection, that of Taylor. The majority of specimens are not yet digitized, and the location of his main collection is unknown.

All collection data were amalgamated into a spreadsheet and manually cleaned. Duplicate specimens of a collection were combined into a single field, and additional columns were added in order to maintain the herbaria where individual specimens are deposited.

We also searched iNaturalist (inaturalist.org) for all vascular plant photograph-based observation records within the flora area.

### ﻿﻿Specimen identification

Vascular plant taxonomy and species identification were based on the relevant global taxonomic literature, including Elven et al. (2011), numerous treatments in the “Flora of North America north of Mexico” (Flora of North America Editorial Committee 1993+), “Flore nordique du Québec et du Labrador” (Payette 2013–2024), and recent taxonomic revisions and nomenclatural updates, including Banfi et al. (2022), Barberá et al. (2019), Elvebakk and Bjerke (2024), Gilman and Testo (2015), Jørgensen et al. (2006), Morin (2020), Schönswetter et al. (2001), Solstad (2009), and Wiegleb et al. (2017).

We reidentified or confirmed almost all collections cited, including most duplicates. A small subset of material was confirmed based on review of specimen images available online. Determinations of a few specimens not seen were accepted based on the authority of previous determiners, especially for taxa that are well known and not taxonomically problematic and/or the determiner is an expert in that particular genus or family. We also reidentified or confirmed records based on photographs on iNaturalist when accurate identifications were possible.

### ﻿﻿Literature review

We reviewed relevant Canadian Arctic floristic literature (Polunin 1940; 1957, 1964; Porsild and Cody 1980; Aiken et al. 2007). We searched the literature for publications on specific localities or by specific collectors (e.g., Taylor 1863; Hainault 1966). We also consulted an unpublished database of specimen records that was developed and used in the production of maps for the “Flora of the Canadian Arctic Archipelago” (Aiken et al. 2007).

We determined the number of taxa recorded in the flora area in Taylor (1863), Polunin (1940), and Hainault (1966). We also counted the number of taxa that were mapped within the flora area in Porsild (1957), Porsild and Cody (1980), and Aiken et al. (2007). We then determined the number of taxa in these floras based on current accepted taxonomy.

### ﻿﻿Patterns of floristic diversity, species richness, and collecting intensity

**Figure 5.**
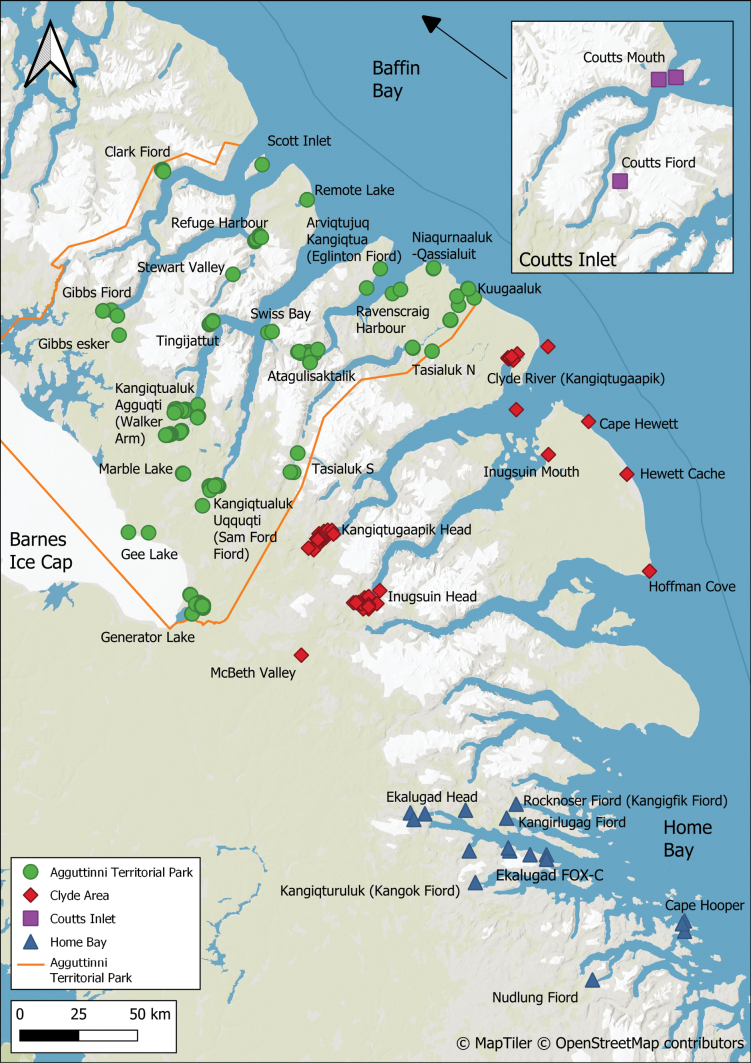
Map of collection sites on east-central Baffin Island grouped by the four main areas (north to south: Coutts Inlet [purple squares], Agguttinni Territorial Park [green dots, boundary shown by orange line], Clyde area [red diamonds], and Home Bay area [blue triangles]) with the 21 main localities labeled.

To facilitate floristic analyses, we divided the study area into four main areas. From south to north, these are Home Bay, the Clyde area, Agguttinni TP, and Coutts Inlet (Fig. 5). The Home Bay region includes all fiords draining into Home Bay and adjacent inland areas. The Clyde area includes Kangiqtugaapik [Clyde Inlet], Inugsuin Fiord, Isabella Fiord, and the hamlet of Clyde River. Agguttinni TP includes all areas within the outer boundary of the park, including Inuit Owned Land that is not part of the park (for more details, see Gillespie et al. 2024). The Coutts Inlet region encompasses the area surrounding the Coutts Inlet fiord complex. No collections from the large area between Agguttinni TP and Coutts Inlet were found. We further grouped collection sites into 38 main localities: 21 in Agguttinni TP, eight in Home Bay, seven in the Clyde area, and three in Coutts Inlet (Table 1, Fig. 5). The Agguttinni TP main localities are identical to those in Gillespie et al. (2024), except for Gee Lake and Generator Lake, which were treated there as sublocalities under Barnes Plateau S. Sites visited by us in Agguttinni TP in 2021 are numbered in the order visited, with the day of the month followed by a site number for that day, following Gillespie et al. (2024). All other collection sites are numbered by locality with an acronym and site number. Locality data for all sites are provided in Table 1.

To characterize floristic diversity patterns, we scored the presence of species/taxa in the four main areas and the 38 main localities. For the 10 most well-collected localities, we estimated the area (km^2^) and determined the number of days spent collecting (i.e., the number of days on which collections were made). The locality area was calculated using QGIS 3.28 to draw convex hulls around the sites at each locality, exporting into Google Earth, and using the polygon measuring tool to determine the area of each locality. For Kangiqtualuk Uqquqti, we excluded site 31.1, which was distant from the other sites and where we spent very little time; no species unique to this locality were found at site 31.1. For Inugsuin Mouth, a locality with a single collecting site, we calculated the area based on the estimated coordinate uncertainty, then subtracted the area occupied by large bodies of water such as fiords. For Clyde River we excluded two distant and/or very imprecise sites (CR-4 and CR-5, representing four collections) and calculated the area based on the estimated coordinate uncertainty of the site with the largest uncertainty (CR-1), minus the area occupied by large bodies of water; this area encompassed all other sites. Two species found at these sites, *Silene sorensenis* and *Taraxacum ceratophorum*, were not collected elsewhere in the Clyde River and were excluded from there in our diversity analyses.

We also scored the presence of taxa in the three ecoregions in the flora area. Based on the map in Ecological Stratification Working Group (1995), we included 13 localities in the Baffin Coastal Lowlands ecoregion (ordered north to south): Scott Inlet, Remote Peninsula, Refuge Harbour, Arviqtujuq Kangiqtua NW, Ravenscraig Harbour, Niaqurnaaluk–Qassialuit, Kuugaaluk, Tasialuk N, Clyde River, Cape Hewett, Inugsuin Mouth, Hewett Cache, and Hoffman Cove. We included Gibbs Esker, Marble Lake, Gee Lake, Generator Lake, and McBeth Valley in the Baffin Uplands ecoregion. The remaining 15 localities were included in the Baffin Mountains ecoregion, including two fiord heads, Kangiqtualuk Agguqti and Kangiqtualuk Uqquqti, that are not included in this ecoregion on the above map but should be based on their climate, low elevation, and mountainous surroundings.

We generated maps showing the locations of all collecting sites using QGIS 3.28. Based on our dataset, we calculated and plotted the number of collections by decade, the cumulative number of collections by year, and the cumulative number of taxa by year. We generated heat maps using QGIS 3.28 to show the density of collections and species richness at each locality.

We explored the relationship between how widespread a taxon is and how many times it was collected by plotting the number of localities where a taxon was found against the number of collections for that taxon. We also performed linear regression between these variables using Excel.

To characterize floristic affinities of the flora, we recorded the global distribution of each taxon following Elven et al. (2011). Distributions were modified based on new information and for simplicity. The pattern Amphi-Atlantic (W) is restricted here to occurrences in Svalbard and/or Iceland in Europe and cited as Amphi-Atlantic (Svalbard) or Amphi-Atlantic (Iceland). Distribution patterns were grouped into major distribution types for analyses.

## ﻿﻿Results

We compiled a dataset of 3,206 unique collections of vascular plants comprising 5,337 specimens from east-central Baffin Island (the flora area as defined above) (Suppl. material 3). We gathered 850 collections in Agguttinni Territorial Park and Clyde River in 2021. Other collectors gathered the following number of collections: Dansereau (571), Hainault (326), Wynne-Edwards (300), Parmelee (159), Forbes (116), Webber (107), Elven (80), Richardson (72), Martin (63), Smith (63), Dutilly (60), and Coombs (50). All other collectors made fewer than 50 collections (see Suppl. material 4).

**Figure 6.**
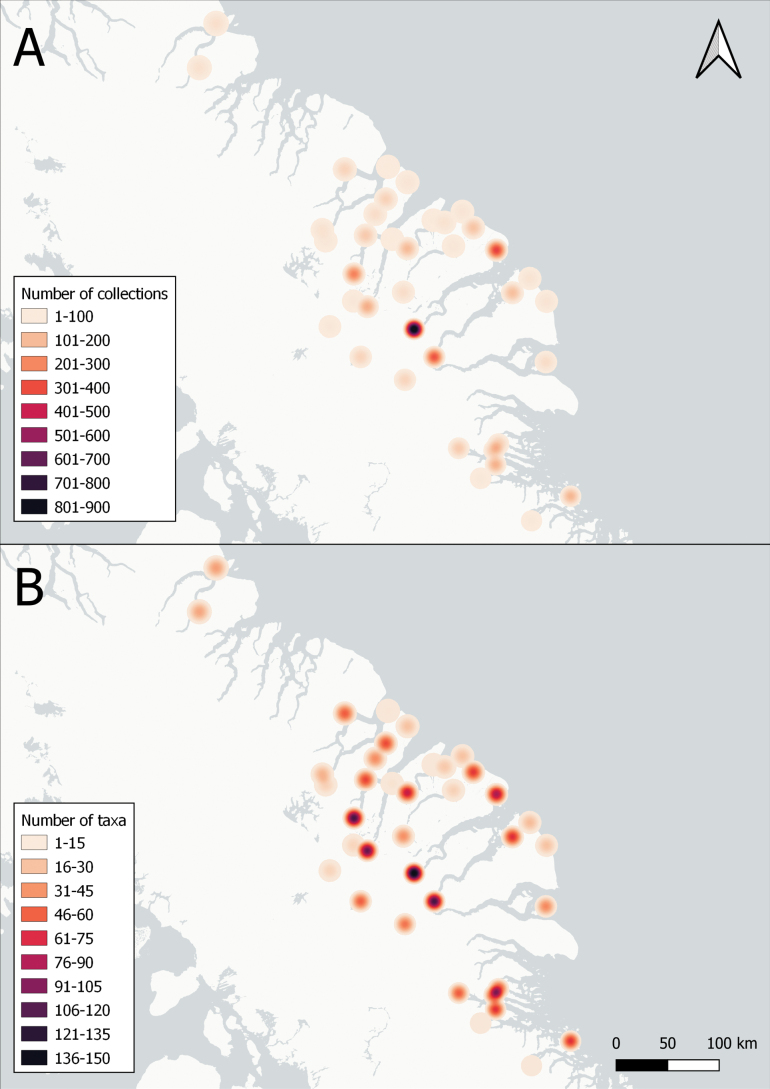
Heat map of vascular plant collections and taxonomic richness in east-central Baffin Island; darker areas indicate higher collection intensity or number of taxa. A. Number of collections; B. Number of taxa.

There is a considerable range in intensity of collections made within the four main collection areas and among specific localities (Figs 5, 6A, Suppl. material 5). The most intensively collected main area is the Clyde area (1,708 collections), followed by Agguttinni TP (952), Home Bay (494), and Coutts Inlet (52). We did not find any collections from the large area between Coutts Inlet and Agguttinni TP, although we know that Taylor (1863) collected at Cape Adair just north of Agguttinni TP between 1856 and 1861.

The ten most intensively collected localities (Figs 5, 6A, Suppl. material 5) are Kangiqtugaapik Head (870 unique collections), Clyde River (330), Inugsuin Head (294), Kangiqtualuk Agguqti (215), Kangiqtualuk Uqquqti (131), Ekalugad FOX-C (124), Cape Hooper (114), Atagulisaktalik (105), Kangirlugag Fiord (98), and Inugsuin Mouth (96). These localities varied considerably in area, as did the number of days on which collections were made: Atagulisaktalik (17.5 km^2^, 12 days), Cape Hooper (5.2 km^2^, 5 days), Clyde River (63.6 km^2^, 74 days), Ekalugad FOX-C (60.9 km^2^, 11 days), Inugsuin Head (36.2 km^2^, 33 days), Inugsuin Mouth (21.7 km^2^, 14 days), Kangiqtualuk Agguqti (83.8 km^2^, 6 days), Kangiqtualuk Uqquqti (3.95 km^2^, 5 days), Kangiqtugaapik Head (30 km^2^, 66 days), and Kangirlugag Fiord (8.66 km^2^, 12 days). Fewer than 10 collections were made at six localities (Arviqtujuq Kangiqtua NW, Gee Lake, Kangok Fiord, Marble Lake, Nudlung Fiord, Swiss Bay).

**Figure 7.**
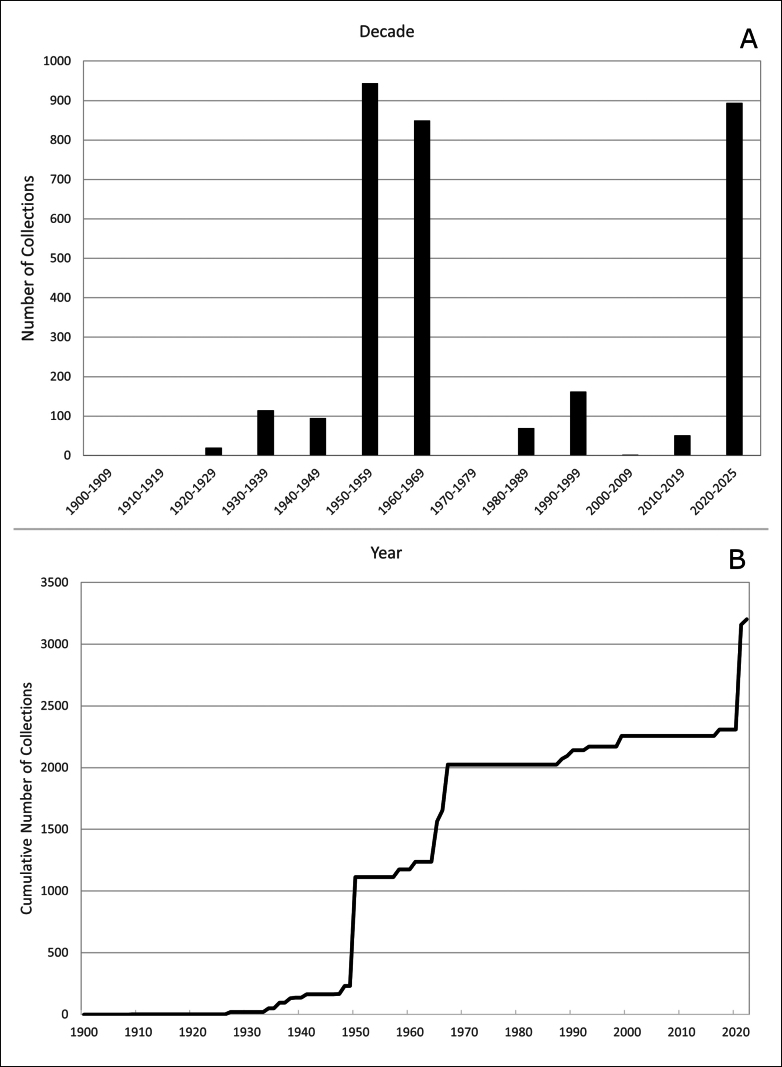
Vascular plant collections from east-central Baffin Island. A. Number of collections from the study area per decade, 1909–present; B. Cumulative number of collections from the study area, 1909–present. Taylor’s collections from 1856–1861 are omitted from these graphs.

The greatest number of collections were made in the 1950s (944), 1960s (849), and 2020s (894) (Fig. 7), mostly reflecting those made by the 1950 Baird Arctic Expedition (881), at the Geographical Branch base camps and field sites in 1965–1967 (651), and by us in Agguttinni TP and Clyde River in 2021 (850). No collections were made between Taylor’s collections in 1856–1861 and 1909, nor in the 1910s or 1970s. The number of collections made in other decades ranged from 1 (1909) to 162 (1990s).

The vascular flora of east-central Baffin Island comprises 26 families, 71 genera, 163 species (four with two subspecies each; hereafter, given only as the number of additional subspecies), and two nothospecies (Tables 2, 3). We recorded 153 taxa in the Clyde area (148 species, three subspecies, and two nothospecies), 143 taxa in Agguttinni TP (140 species and three subspecies), 96 taxa in the Home Bay area (93 species and three subspecies), and 35 taxa in Coutts Inlet (35 species). These numbers do not include three unnamed hybrids (*Potentilla arenosa* × *P. vahliana*, *Salix arctica* × *S. arctophila*, *Tofieldia coccinea* × *T. pusilla*) that are discussed in the annotated checklist. Lycophytes (Lycopodiopsida) comprise one family, one genus, and one species. Monilophytes (Polypodiopsida) comprise four families, four genera, and five species. Flowering plants (angiosperms) comprise 21 families, 66 genera, 156 species, four subspecies, and two nothospecies. Of the angiosperms, four families, 18 genera, 58 species, two subspecies, and one nothospecies are monocots, and 17 families, 48 genera, 98 species, two subspecies, and one nothospecies are eudicots. A complete list of taxa is presented in Table 3 and the annotated checklist.

**Figure 8.**
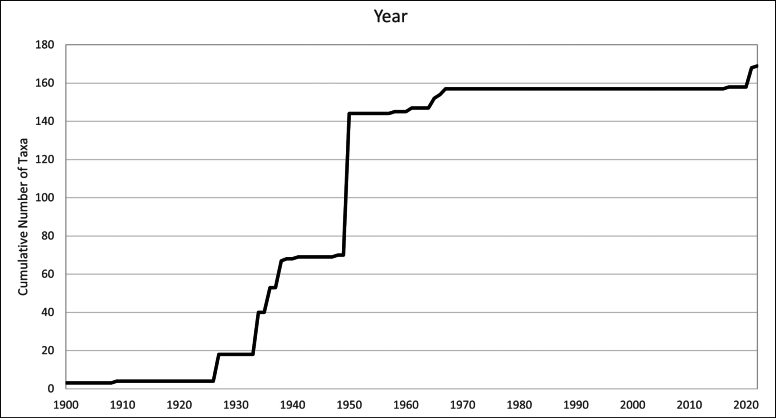
Cumulative number of vascular plant taxa known from east-central Baffin Island, 1900–present. The year 1900 includes three species based on Taylor’s 1856–1861 collections examined here.

The cumulative number of taxa known from east-central Baffin Island based on collections examined is presented in Fig. 8. Note the stepped rise in known taxa in the 1930s based on collections made by various collectors mostly from Clyde River (68 taxa by 1939), followed by a sharp rise in 1950 as a result of collections made during the 1950 Baird Arctic Expedition (144 taxa) and a moderate rise in the mid-1960s (157 taxa by 1967). No new taxa were added between 1968 and 2016. The number of taxa continued to rise subsequently, particularly in 2021 as a result of our fieldwork in Agguttinni TP (168 taxa).

Table 2 provides a list of families and genera in east-central Baffin Island and the number of taxa in each. Only one family is represented by more than 10 genera: Poaceae with 13 genera. The next five largest families are Brassicaceae (8 genera), Caryophyllaceae (8), Asteraceae (7), Ericaceae (7), and Polygonaceae (3). Of the remaining 20 families, five are represented by two genera, and 15 by a single genus. The largest families in terms of number of species and taxa are Cyperaceae (26 species [28 taxa]), Poaceae (24 [25]), Brassicaceae (16), Caryophyllaceae (15), Saxifragaceae (11 [12]), and Asteraceae (11). Four families are represented by two species and eight by only a single species. The largest genera are *Carex* (19 species), *Draba* (9), *Potentilla* (8 [10 taxa]), *Saxifraga* (8 [9]), *Ranunculus* (7), and *Eriophorum* (7). The remaining genera have five or fewer species. Two nothotaxa were recorded, one in *Eriophorum* and one in *Potentilla*. Four species had two subspecies: *Eriophorum scheuchzeri*, *Poa arctica*, *Potentilla arenosa*, and *Saxifraga rivularis*.

**Table 2.** Families and genera of vascular plants in east central Baffin Island. Families are arranged alphabetically within major clades (lycophytes, monilophytes, angiosperm monocots and eudicots), and the number of genera and species and taxa are given for each. Genera are arranged alphabetically within families and the number of species and taxa are given for each. Number of taxa is provided only if nothospecies or species with multiple subspecies are present.

	Family	Genera	Species/Taxa	Genus	Species/Taxa
Lycophytes	Lycopodiaceae	1	1	* Huperzia *	1
Monilophytes	Equisetaceae	1	2	* Equisetum *	2
	Cystopteridaceae	1	1	* Cystopteris *	1
	Dryopteridaceae	1	1	* Dryopteris *	1
	Woodsiaceae	1	2	* Woodsia *	2
Angiosperms					
Monocots	Cyperaceae	2	26/28	* Carex *	19
				* Eriophorum *	7/9
	Juncaceae	2	6	* Juncus *	4
				* Luzula *	2
	Poaceae	13	24/25	* Alopecurus *	1
				* Anthoxanthum *	1
				* Arctagrostis *	1
				* Calamagrostis *	1
				* Deschampsia *	2
				* Dupontia *	1
				* Festuca *	3
				* Koeleria *	1
				* Leymus *	1
				* Phippsia *	1
				* Pleuropogon *	1
				* Poa *	5/6
				* Puccinellia *	5
	Tofieldiaceae	1	2	* Tofieldia *	2
Eudicots	Asteraceae	7	11	* Antennaria *	2
				* Arnica *	1
				* Askellia *	1
				* Erigeron *	2
				* Hulteniella *	1
				* Taraxacum *	3
				* Tripleurospermum *	1
	Boraginaceae	1	1	* Mertensia *	1
	Brassicaceae	8	16	* Arabidopsis *	1
				* Braya *	1
				* Cardamine *	1
				* Cochlearia *	1
				* Crucihimalaya *	1
				* Draba *	9
				* Eutrema *	1
				* Physaria *	1
	Campanulaceae	1	1	* Melanocalyx *	1
	Caryophyllaceae	8	15	* Arenaria *	1
				* Cerastium *	2
				* Cherleria *	1
				* Honckenya *	1
				* Sabulina *	2
				* Sagina *	2
				* Silene *	4
				* Stellaria *	2
	Diapensiaceae	1	1	* Diapensia *	1
	Ericaceae	7	9	* Arctous *	1
				* Cassiope *	1
				* Empetrum *	1
				* Harrimanella *	1
				* Pyrola *	1
				* Rhododendron *	2
				* Vaccinium *	2
	Onagraceae	2	2	* Chamaenerion *	1
				* Epilobium *	1
	Orobanchaceae	1	3	* Pedicularis *	3
	Papaveraceae	1	2	* Oreomecon *	2
	Plantaginaceae	1	1	* Hippuris *	1
	Plumbaginaceae	1	1	* Armeria *	1
	Polygonaceae	3	3	* Bistorta *	1
				* Koenigia *	1
				* Oxyria *	1
	Ranunculaceae	1	7	* Ranunculus *	7
	Rosaceae	2	9/11	* Dryas *	1
				* Potentilla *	8/10
	Salicaceae	1	5	* Salix *	5
	Saxifragaceae	2	11/12	* Micranthes *	3
				* Saxifraga *	8/9
Totals		71	163/169		163/169

**Table 3.** Vascular plant taxa recorded from east-central Baffin Island. Species, infraspecific taxa, and nothotaxa are listed by major clade and then alphabetically by family. The dataset records presence of each taxon in the four main areas (Coutts Inlet, Agguttinni Territorial Park, Clyde area, and Home Bay area, ordered north to south), and in the 10 most collected main localities (Atagulisaktalik, Kangiqtualuk Agguqti, and Kangiqtualuk Uqquqti in Agguttinni TP; Clyde River, Inugsuin Head, Inugsuin Mouth, and Kangiqtugaapik Head in the Clyde area; Cape Hooper, Ekalugad FOX-C, and Kangirlugag Fiord in the Home Bay area). Also recorded are the number of taxa and the number of collections in each of the four main areas and 10 most collected localities.

Family	Taxon	Coutts Inlet	Atagulisaktalik	Kangiqtualuk Agguqti	Kangiqtualuk Uqquqti	Aggutinni TP	Clyde River	Inugsuin Head	Inugsuin Mouth	Kangiqtugaapik Head	Clyde Area	Cape Hooper	Ekalugad FOX-C	Kangirlugag Fiord	Home Bay	Total Localities
Lycophytes																
Lycopodiaceae	*Huperzia arctica*		●	●	●	♦	●	●		●	♦	●	●	●	♦	17
Monilophytes																
Cystopteridaceae	*Cystopteris fragilis*			●	●	♦		●		●	♦					6
Dryopteridaceae	*Dryopteris fragrans*			●	●	♦		●		●	♦					4
Equisetaceae	Equisetum arvense subsp. alpestre		●			♦	●	●	●	●	♦	●	●	●	♦	10
	Equisetum variegatum subsp. variegatum				●	♦				●	♦					2
Woodsiaceae	*Woodsia alpina*			●	●	♦				●	♦					3
	*Woodsia glabella*							●		●	♦					2
Angiosperms																
Monocots																
Cyperaceae	Carex aquatilis subsp. stans		●	●	●	♦	●	●	●	●	♦		●	●	♦	14
	*Carex atrofusca*			●	●	♦		●		●	♦					6
	Carex bigelowii subsp. bigelowii	♦	●	●	●	♦	●	●	●	●	♦	●	●	●	♦	22
	Carex capillaris subsp. fuscidula		●	●	●	♦		●		●	♦					9
	Carex fuliginosa subsp. misandra	♦	●	●	●	♦	●	●	●	●	♦	●	●	●	♦	22
	Carex glareosa subsp. glareosa							●	●	●	♦				♦	4
	*Carex holostoma*			●	●	♦										2
	*Carex marina*			●		♦										1
	*Carex maritima*			●	●	♦	●			●	♦					5
	*Carex membranacea*		●	●	●	♦	●	●		●	♦			●	♦	14
	*Carex myosuroides*		●	●	●	♦		●	●	●	♦					10
	*Carex nardina*		●	●	●	♦	●	●	●	●	♦	●	●		♦	17
	*Carex rupestris*			●	●	♦		●		●	♦					7
	*Carex saxatilis*						●			●	♦					2
	Carex scirpoidea subsp. scirpoidea			●	●	♦		●		●	♦					4
	Carex simpliciuscula subsp. subholarctica			●	●	♦				●	♦					3
	*Carex subspathacea*									●	♦			●	♦	2
	Carex supina subsp. spaniocarpa			●	●	♦				●	♦					5
	*Carex ursina*					♦	●		●	●	♦		●	●	♦	7
	*Eriophorum angustifolium*	♦	●	●		♦	●	●	●	●	♦	●	●	●	♦	16
	*Eriophorum brachyantherum*			●		♦				●	♦					2
	*Eriophorum callitrix*			●	●	♦				●	♦					3
	Eriophorum × medium subsp. album						●				♦					1
	Eriophorum russeolum subsp. albidum					♦			●	●	♦					3
	Eriophorum scheuchzeri subsp. arcticum	♦	●	●		♦	●	●		●	♦	●	●	●	♦	15
	Eriophorum scheuchzeri subsp. scheuchzeri							●	●	●	♦					3
	*Eriophorum triste*			●	●	♦	●	●	●	●	♦	●	●		♦	14
Cyperaceae	Eriophorum vaginatum subsp. spissum													●	♦	2
Juncaceae	*Juncus arcticus*							●		●	♦					2
	*Juncus biglumis*		●	●	●	♦	●	●	●	●	♦	●	●	●	♦	15
	*Juncus leuchoclamys*			●	●	♦	●	●		●	♦		●		♦	7
	Juncus triglumis subsp. albescens			●	●	♦		●		●	♦				♦	5
	*Luzula confusa*	♦	●	●	●	♦	●	●	●	●	♦	●	●	●	♦	27
	*Luzula nivalis*		●	●	●	♦	●	●	●	●	♦	●	●	●	♦	20
Poaceae	*Alopecurus borealis*	♦				♦	●		●	●	♦	●	●	●	♦	15
	Anthoxanthum monticola subsp. alpinum	♦	●	●	●	♦	●	●	●	●	♦	●	●	●	♦	24
	Arctagrostis latifolia subsp. latifolia	♦	●	●	●	♦	●	●	●	●	♦	●	●	●	♦	22
	*Calamagrostis purpurascens*			●		♦				●	♦					2
	*Deschampsia brevifolia*					♦	●				♦		●	●	♦	6
	*Deschampsia sukatschewii*					♦	●	●		●	♦					4
	*Dupontia fisheri*		●	●	●	♦	●	●	●	●	♦		●	●	♦	13
	*Festuca baffinensis*				●	♦										1
	Festuca brachyphylla subsp. brachyphylla	♦	●	●		♦	●	●	●	●	♦	●	●	●	♦	22
	*Festuca hyperborea*			●	●	♦			●		♦				♦	10
	*Koeleria spicata*		●			♦			●	●	♦	●	●	●	♦	9
	Leymus mollis subsp. villosissimus			●		♦				●	♦					2
	*Phippsia algida*			●		♦	●	●	●		♦	●	●	●	♦	13
	*Pleuropogon sabinei*		●			♦	●	●	●	●	♦				♦	8
	Poa abbreviata subsp. abbreviata			●		♦	●				♦					2
	Poa alpigena var. alpigena						●	●		●	♦					3
	Poa arctica subsp. arctica	♦	●	●	●	♦	●	●	●	●	♦	●	●	●	♦	24
	Poa arctica subsp. caespitans			●		♦		●		●	♦	●			♦	5
	Poa glauca subsp. glauca			●	●	♦	●	●		●	♦				♦	11
	Poa hartzii subsp. hartzii				●	♦				●	♦					2
	*Puccinellia angustata*			●		♦		●		●	♦		●	●	♦	7
	*Puccinellia bruggemannii*					♦										1
	Puccinellia phryganodes subsp. neoarctica			●		♦	●	●	●	●	♦		●	●	♦	10
	Puccinellia tenella subsp. langeana		●	●	●	♦		●	●	●	♦		●	●	♦	10
	*Puccinellia vahliana*									●	♦					1
Tofieldiaceae	*Tofieldia coccinea*			●	●	♦		●		●	♦					8
	*Tofieldia pusilla*			●	●	♦				●	♦					3
Dicots																
Asteraceae	Antennaria friesiana subsp. friesiana	♦	●	●	●	♦		●	●	●	♦	●	●		♦	19
	Antennaria media subsp. compacta							●		●	♦					2
	Arnica angustifolia subsp. angustifolia							●	●	●	♦			●	♦	4
	*Askellia pygmaea*		●		●	♦									♦	5
	*Erigeron compositus*									●	♦					1
	*Erigeron eriocephalus*		●			♦				●	♦				♦	4
	*Hulteniella integrifolia*			●	●	♦										2
	*Taraxacum ceratophorum*		●	●	●	♦	●	●	●	●	♦				♦	14
	*Taraxacum holmenianum*				●	♦										2
	*Taraxacum phymatocarpum*		●	●	●	♦		●		●	♦					6
Asteraceae	Tripleurospermum maritimum subsp. phaeocephalum					♦										1
Boraginaceae	Mertensia maritima subsp. tenella					♦	●	●		●	♦					5
Brassicaceae	*Arabidopsis arenicola*					♦										1
	Braya glabella subsp. purpurescens			●	●	♦				●	♦		●		♦	4
	*Cardamine bellidifolia*		●	●	●	♦	●	●	●	●	♦	●		●	♦	15
	*Cochlearia groenlandica*		●	●	●	♦	●	●	●	●	♦		●		♦	10
	*Crucihimalaya bursifolia*									●	♦					1
	*Draba arctica*			●	●	♦				●	♦					3
	*Draba cinerea*			●	●	♦		●		●	♦					7
	*Draba corymbosa*				●	♦				●	♦		●		♦	5
	*Draba fladnizensis*	♦	●		●	♦		●		●	♦	●			♦	7
	*Draba glabella*							●		●	♦					2
	*Draba lactea*		●	●	●	♦	●	●	●	●	♦	●	●	●	♦	23
	*Draba nivalis*	♦	●	●	●	♦	●	●	●	●	♦			●	♦	18
	*Draba oblongata*									●	♦					1
	*Draba subcapitata*					♦										1
	*Eutrema edwardsii*			●	●	♦	●			●	♦					6
	*Physaria arctica*			●	●	♦		●		●	♦					4
Campanulaceae	*Melanocalyx uniflora*		●	●	●	♦	●	●	●	●	♦	●		●	♦	14
Caryophyllaceae	*Arenaria humifusa*			●	●	♦										2
	Cerastium alpinum subsp. alpinum				●	♦	●			●	♦					5
	*Cerastium arcticum*	♦	●	●	●	♦	●	●	●	●	♦	●	●	●	♦	22
	*Cherleria biflora*					♦	●				♦	●	●	●	♦	5
	Honckenya peploides subsp. diffusa		●	●	●	♦	●	●		●	♦				♦	11
	*Sabulina rubella*			●	●	♦		●		●	♦					8
	*Sabulina stricta*			●	●	♦				●	♦					3
	*Sagina caespitosa*					♦			●		♦	●			♦	4
	*Sagina nivalis*		●	●	●	♦	●	●	●		♦	●	●	●	♦	13
	*Silene acaulis*	♦	●	●	●	♦	●	●		●	♦	●	●	●	♦	20
	Silene involucrata subsp. involucrata		●	●	●	♦	●	●	●	●	♦				♦	15
	*Silene sorensenis*					♦	●	●	●	●	♦					7
	Silene uralensis subsp. arctica		●	●	●	♦	●	●	●	●	♦	●		●	♦	14
	*Stellaria humifusa*				●	♦	●	●	●	●	♦		●		♦	11
	Stellaria longipes subsp. longipes	♦	●	●	●	♦	●	●	●	●	♦	●	●	●	♦	23
Diapensiaceae	*Diapensia lapponica*			●		♦		●	●	●	♦			●	♦	8
Ericaceae	*Arctous alpina*										♦					1
	Cassiope tetragona subsp. tetragona	♦	●	●	●	♦	●	●		●	♦	●	●	●	♦	20
	*Empetrum nigrum*		●	●	●	♦	●	●		●	♦	●		●	♦	15
	*Harrimanella hypnoides*											●		●	♦	2
	*Pyrola grandiflora*	♦	●	●	●	♦	●	●	●	●	♦	●		●	♦	21
	*Rhododendron lapponicum*			●	●	♦		●		●	♦				♦	10
	Rhododendron tomentosum subsp. decumbens		●			♦	●	●		●	♦			●	♦	10
	*Vaccinium uliginosum*		●	●	●	♦	●	●		●	♦	●	●	●	♦	18
	*Vaccinium vitis-idaea*					♦										1
Onagraceae	*Chamaenerion latifolium*	♦	●	●	●	♦		●	●	●	♦	●	●		♦	18
	*Epilobium arcticum*					♦								●	♦	3
Orobanchaceae	*Pedicularis flammea*			●	●	♦		●		●	♦	●			♦	7
	*Pedicularis hirsuta*	♦	●	●	●	♦	●	●	●	●	♦	●	●	●	♦	25
	*Pedicularis lanata*			●	●	♦				●	♦					4
Papaveraceae	*Oreomecon labradorica*	♦	●		●	♦	●	●	●	●	♦	●	●	●	♦	17
	*Oreomecon lapponica*	♦	●	●		♦	●	●		●	♦		●		♦	15
Plantaginaceae	*Hippuris lanceolata*							●		●	♦					2
Plumbaginaceae	Armeria maritima subsp. sibirica	♦	●	●	●	♦		●	●	●	♦			●	♦	15
Polygonaceae	*Bistorta vivipara*	♦	●	●	●	♦	●	●		●	♦	●	●	●	♦	21
	*Koenigia islandica*		●	●		♦	●	●	●	●	♦	●			♦	10
	*Oxyria digyna*	♦	●	●	●	♦	●	●		●	♦	●	●	●	♦	20
Ranunculaceae	*Ranunculus arcticus*			●		♦				●	♦					2
	Ranunculus hyperboreus subsp. hyperboreus		●	●		♦		●		●	♦				♦	8
	*Ranunculus nivalis*		●	●	●	♦	●	●	●	●	♦	●	●	●	♦	18
	*Ranunculus pygmaeus*		●			♦	●		●	●	♦	●			♦	7
	*Ranunculus sabinei*												●	●	♦	2
	*Ranunculus sulphureus*						●				♦					1
	*Ranunculus trichophyllus*									●	♦					1
Rosaceae	Dryas integrifolia subsp. integrifolia	♦	●	●	●	♦	●	●		●	♦	●	●	●	♦	18
	Potentilla arenosa subsp. arenosa			●	●	♦		●		●	♦					4
	Potentilla a renosa subsp. chamissonis			●		♦				●	♦					2
	Potentilla hyparctica subsp. hyparctica	♦	●	●	●	♦	●	●	●	●	♦	●	●	●	♦	24
	*Potentilla nivea*					♦		●		●	♦					3
	*Potentilla pedersenii*							●		●	♦					2
	*Potentilla × prostrata*							●		●	♦					2
	*Potentilla pulchella*			●		♦		●		●	♦		●		♦	4
	*Potentilla subgorodkovii*			●		♦		●		●	♦					3
	*Potentilla subvahliana*			●	●	♦		●		●	♦					9
	*Potentilla vahliana*	♦	●		●	♦		●	●	●	♦	●	●	●	♦	18
Salicaceae	*Salix arctica*	♦	●	●	●	♦	●	●	●	●	♦	●	●	●	♦	24
	*Salix arctophila*							●			♦	●			♦	2
	*Salix herbacea*		●	●	●	♦	●	●		●	♦	●	●	●	♦	16
	*Salix reticulata*	♦	●	●	●	♦	●	●		●	♦					11
	*Salix richardsonii*			●	●	♦		●		●	♦					7
Saxifragaceae	*Micranthes foliolosa*		●	●		♦	●		●	●	♦	●	●	●	♦	19
	*Micranthes nivalis*		●	●	●	♦	●	●	●	●	♦	●	●	●	♦	19
	*Micranthes tenuis*			●	●	♦		●			♦				♦	5
	*Saxifraga aizoides*			●	●	♦		●		●	♦					4
	*Saxifraga cernua*		●	●	●	♦	●	●	●	●	♦	●	●	●	♦	21
	Saxifraga cespitosa subsp. cespitosa	♦	●	●	●	♦	●	●		●	♦					9
	*Saxifraga hirculus*									●	♦					1
	*Saxifraga hyperborea*		●		●	♦	●			●	♦	●	●	●	♦	10
	Saxifraga oppositifolia subsp. oppositifolia	♦	●	●	●	♦	●	●		●	♦	●	●		♦	18
	Saxifraga rivularis subsp. arctolitoralis			●		♦	●	●	●	●	♦	●			♦	9
	Saxifraga rivularis subsp. rivularis			●		♦	●	●		●	♦	●		●	♦	10
	*Saxifraga tricuspidata*	♦	●	●	●	♦		●		●	♦				♦	13
Total taxa		33	69	109	98	143	78	109	59	141	153	58	58	62	96	

Global distributions of the 169 taxa present in east-central Baffin Island are provided in the annotated checklist and in Suppl. material 5. Common distribution patterns are circumpolar–alpine (47 taxa), circumpolar (34), and circumboreal–polar (14). Including one minor circumpolar pattern (circumpolar–amphi-Pacific (N)), these circumpolar patterns account for 96 taxa, or 57% of the total number. Six taxa are almost circumpolar (4%). North American distribution patterns account for ten taxa (6%), of which four (Eriophorum vaginatum subsp. spissum, *Arabidopsis arenicola*, *Oreomecon labradorica*, *Potentilla vahliana*) are restricted to northeastern North America and six (Eriophorum × medium subsp. album, *Puccinellia bruggemannii*, P. phryganodes subsp. neoarctica, *Salix arctophila*, *Saxifraga tricuspidata*, *Taraxacum holmenianum*) are more widespread across the north. Patterns encompassing North America and Asia include 25 taxa (15%), with those restricted to Beringia in Asia accounting for 11 taxa (6%). An additional four taxa are distributed primarily in Asia/Beringia and North America but barely extend into Europe (only in Svalbard and/or Iceland). Patterns encompassing North America and Europe account for 13 taxa (8%), including four taxa with a strictly amphi-Atlantic distribution and two that extend only into Iceland or Svalbard. Ten taxa (6%) are distributed in Asia, North America, and Europe; nine of these have large gaps in Asia. Two taxa (1%) are widespread globally.

Table 3 provides a list of taxa and their occurrence in the four main areas. Twenty taxa were recorded only from the Clyde area: Antennaria media subsp. compacta, *Arctous alpina*, *Carex saxatilis*, *C. ursina*, *Crucihimalaya bursifolia*, *Draba glabella*, *D. oblongata*, *Erigeron compositus*, Eriophorum × medium subsp. album, E. scheuchzeri subsp. scheuchzeri, *Hippuris lanceolata*, *Juncus arcticus*, Poa alpigena var. alpigena, *Potentilla pedersenii*, *P. × prostrata*, *Puccinellia vahliana*, *Ranunculus sulphureus*, *R. trichophyllus*, *Saxifraga hirculus*, and *Woodsia glabella*. Eleven taxa were recorded only from Agguttinni TP: *Arabidopsis arenicola*, *Arenaria humifusa*, *Carex holostoma*, *C. marina*, *Draba subcapitata*, *Festuca baffinensis*, *Hulteniella integrifolia*, *Puccinellia bruggemannii*, *Taraxacum holmenianum*, Tripleurospermum maritimum subsp. phaeocephalum, and *Vaccinium vitis-idaea*. Three taxa were recorded only from the Home Bay area: Eriophorum vaginatum subsp. spissum, *Harrimanella hypnoides*, and *Ranunculus sabinei*. No species were unique to the Coutts Inlet area.

Species richness per locality varied considerably (Figs 5, 6B). Species occurrences in localities that have been the most botanically explored and/or comprehensively sampled are provided in Table 3, and species occurrences for all localities are provided in Suppl. material 5. Localities with the highest number of taxa were Kangiqtugaapik Head (141), Inugsuin Head (109), Kangiqtualuk Agguqti (109), Kangiqtualuk Uqquqti (98), Clyde River (78), Atagulisaktalik (69), Kangirlugag Fiord (62), Inugsuin Mouth (59), Cape Hooper (58), Ekalugad FOX-C (58), Kuugaaluk (56), Rocknoser Fiord (55), Tingijattut (52), and Refuge Harbour (50). All other localities were recorded as having fewer than 50 taxa.

**Figure 9.**
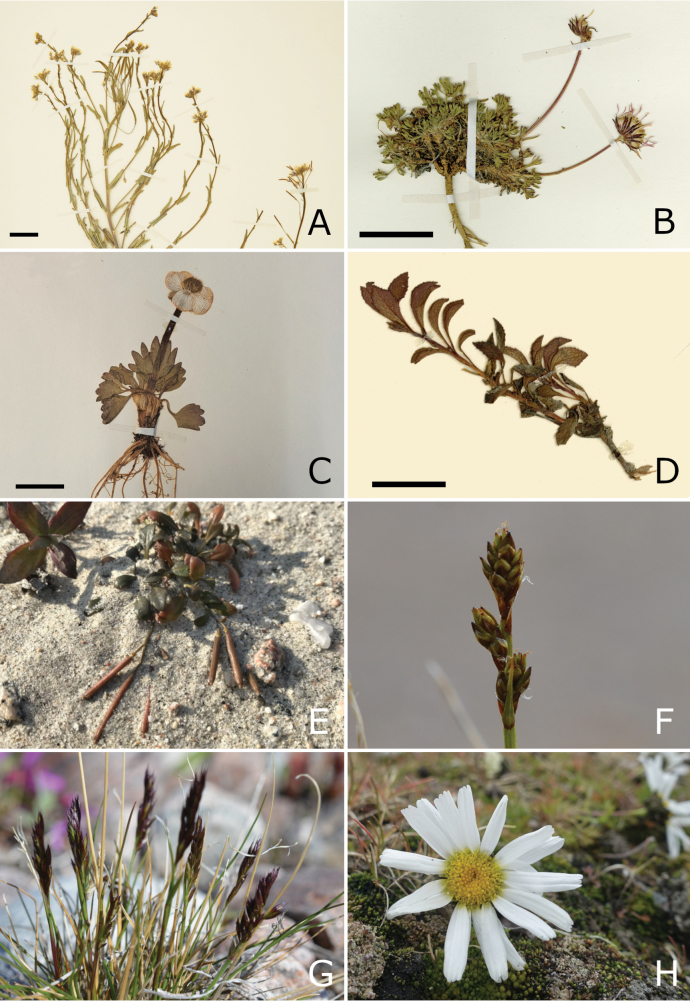
Species known from the east-central Baffin Island study area from a single collection or locality. A. *Crucihimalaya bursifolia* (Wynne-Edwards 8945 [CAN 10057914]); B. *Erigeron compositus* (Wynne-Edwards 9061 [CAN 10086649]); C. *Ranunculus sulphureus* (Martin 2 [DAO 01-01667470]); D. *Arctous alpina* (Hainault 3797 [CAN 10075863]); E. *Arabidopsis arenicola* (Gillespie et al. 12438); F. *Carex marina* (Gillespie et al. 11965); G. *Festuca baffinensis* (Gillespie et al. 11718); H. Tripleurospermum maritimum subsp. phaeocephalum (Gillespie et al. 12235). Photos by W. Wang (A), P.C. Sokoloff (B, D–G), G.A. Levin (C), and L.J. Gillespie (H). Scale bars: 2 cm (A–D).

Fifteen taxa were recorded from only a single locality (Table 3). Nine of these were recorded only from the Clyde area: *Crucihimalaya bursifolia* (Fig. 9A), *Draba oblongata*, *Erigeron compositus* (Fig. 9B), *Puccinellia vahliana*, *Ranunculus trichophyllus*, and *Saxifraga hirculus* from Kangiqtugaapik Head; Eriophorum × medium subsp. album and *Ranunculus sulphureus* (Fig. 9C) from Clyde River; and *Arctous alpina* (Fig. 9D) from McBeth Valley. The remaining six taxa, each known from only a single collection, were recorded from Agguttinni TP: *Arabidopsis arenicola* (Fig. 9E) from Tasialuk S; *Carex marina* (Fig. 9F) from Kangiqtualuk Agguqti; *Puccinellia bruggemannii* from Kuugaaluk; *Draba subcapitata* from Generator Lake; *Festuca baffinensis* (Fig. 9G) from Kangiqtualuk Uqquqti; Tripleurospermum maritimum subsp. phaeocephalum (Fig. 9H) from Niaqurnaaluk–Qassialuit; and *Vaccinium vitis-idaea* from Arviqtujuq Kangiqtua NW. A further 23 taxa were recorded from only two localities: eight from both Agguttinni TP and the Clyde area (*Calamagrostis purpurascens*, Equisetum variegatum subsp. variegatum, *Eriophorum brachyantherum*, Leymus mollis subsp. villosissimus, Poa abbreviata subsp. abbreviata, Poa hartzii subsp. hartzii, Potentilla arenosa subsp. chamissonis, *Ranunculus arcticus*); seven from the Clyde area (Antennaria media subsp. compacta, *Carex saxatilis*, *Draba glabella*, *Hippuris lanceolata*, *Potentilla pedersenii*, *P. prostrata*, *Woodsia glabella*); four from Agguttinni TP (*Arenaria humifusa*, *Carex holostoma*, *Hulteniella integrifolia*, *Taraxacum holmenianum*); two from Home Bay (Eriophorum vaginatum subsp. spissum, *Harrimanella hypnoides*); and two from Home Bay and the Clyde area (*Salix arctophila*, *Carex subspathacea*).

**Figure 10.**
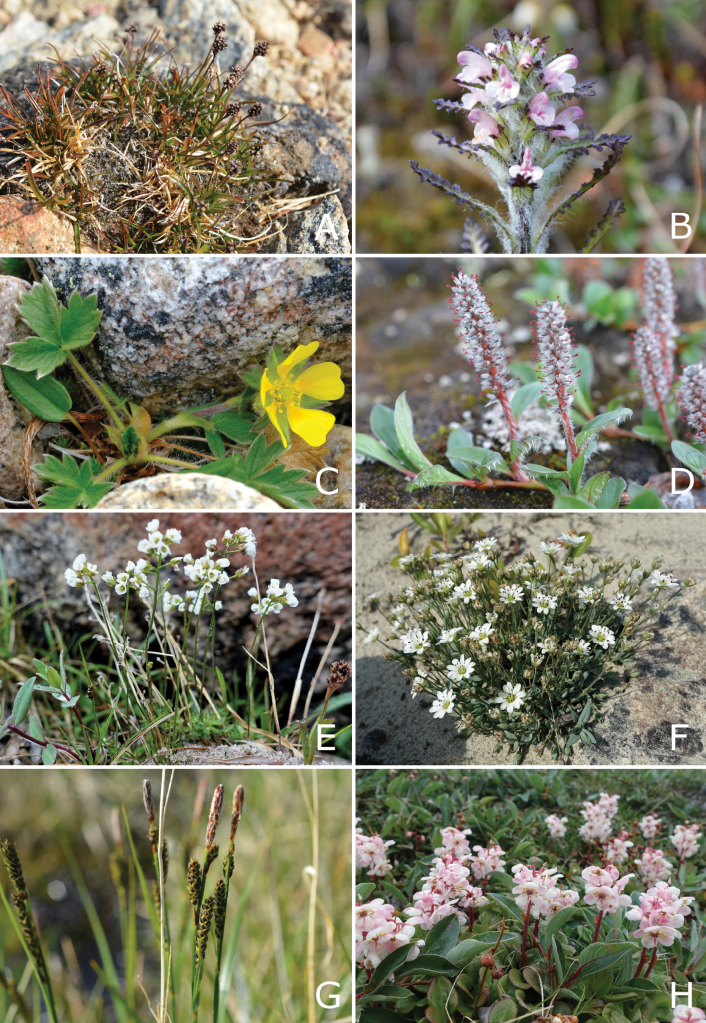
Common widespread species in the east-central Baffin Island study area. A. *Luzula confusa* (Gillespie et al. 11751); B. *Pedicularis hirsuta* (Gillespie et al. 11745); C. Potentilla hyparctica subsp. hyparctica (Gillespie et al. 11792); D. *Salix arctica* (Gillespie et al. 12028); E. *Draba lactea* (Gillespie et al. 11696); F. Stellaria longipes subsp. longipes (Gillespie et al. 12440); G. Carex bigelowii subsp. bigelowii (Gillespie et al. 12352a); H. *Pyrola grandiflora*, head of Kangiqtualuk Uqquqti, August 1, 2021. Photos by P.C. Sokoloff (A, B, D, G) and L.J. Gillespie (C, E, F, H).

The most widespread taxa in terms of number of localities were: *Luzula confusa* (27 localities) (Fig. 10A); *Pedicularis hirsuta* (25) (Fig. 10B); Anthoxanthum monticola subsp. alpinum, Poa arctica subsp. arctica, Potentilla hyparctica subsp. hyparctica (Fig. 10C), *Salix arctica* (Fig. 10D) (24); *Draba lactea* (Fig. 10E), Stellaria longipes subsp. longipes (Fig. 10F) (23); Arctagrostis latifolia subsp. latifolia, Carex bigelowii subsp. bigelowii (Fig. 10G), Carex fuliginosa subsp. misandra, *Cerastium arcticum*, Festuca brachyphylla subsp. brachyphylla (22); *Bistorta vivipara*, *Pyrola grandiflora* (Fig. 10H), *Saxifraga cernua* (21); and Cassiope tetragona subsp. tetragona, *Luzula nivalis*, *Oxyria digyna*, and *Silene acaulis* (20).

The most commonly collected taxa based on number of collections were: *Draba lactea* (77 collections), Carex bigelowii subsp. bigelowii (73), Potentilla hyparctica subsp. hyparctica (73), *Salix arctica* (72), Stellaria longipes subsp. longipes (65), *Luzula confusa* (60), Poa arctica subsp. arctica (58), Dryas integrifolia subsp. integrifolia (56), *Draba nivalis* (54), Anthoxanthum monticola subsp. alpinum (51), and Arctagrostis latifolia subsp. latifolia (51). Other species were rare in the flora area, with 33 taxa known from only three or fewer collections: 14 from one, 11 from two, and eight from three collections.

**Figure 11.**
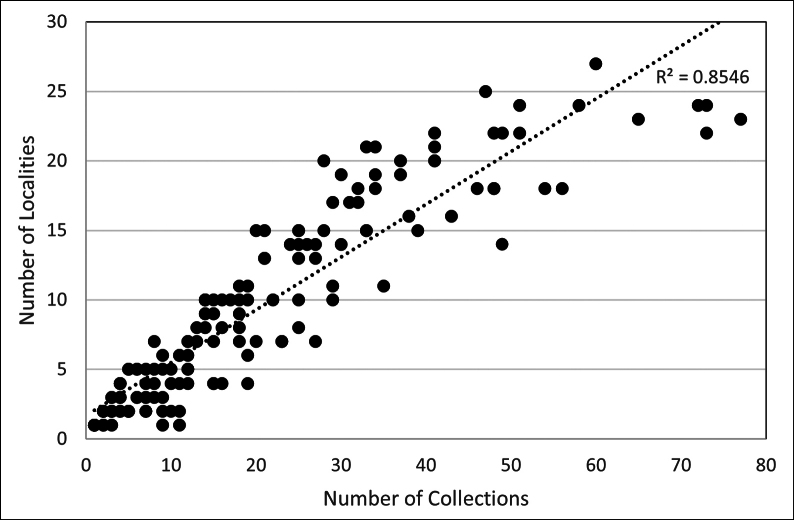
Number of localities in east-central Baffin Island where each taxon is found plotted against the number of collections for that taxon.

There is a strong positive correlation (R^2^ = 0.855) between the number of collections for a taxon and the number of localities where it is found (Fig. 11). Thus, widespread species generally are represented by more collections. Indeed, seven of the eight most widespread taxa in terms of number of localities (*Luzula confusa*, *Pedicularis hirsuta*, Anthoxanthum monticola subsp. alpinum, Poa arctica subsp. arctica, Potentilla hyparctica subsp. hyparctica, *Salix arctica*, *Draba lactea*, and Stellaria longipes subsp. longipes) were among the 10 most commonly collected taxa. However, some species were collected more times than the breadth of their distribution would suggest—for example, some *Carex* and *Draba* species—and some others, for example, Cassiope tetragona subsp. tetragona and *Pedicularis hirsuta*, were represented by fewer collections than expected based on the number of localities where they are found.

**Figure 12.**
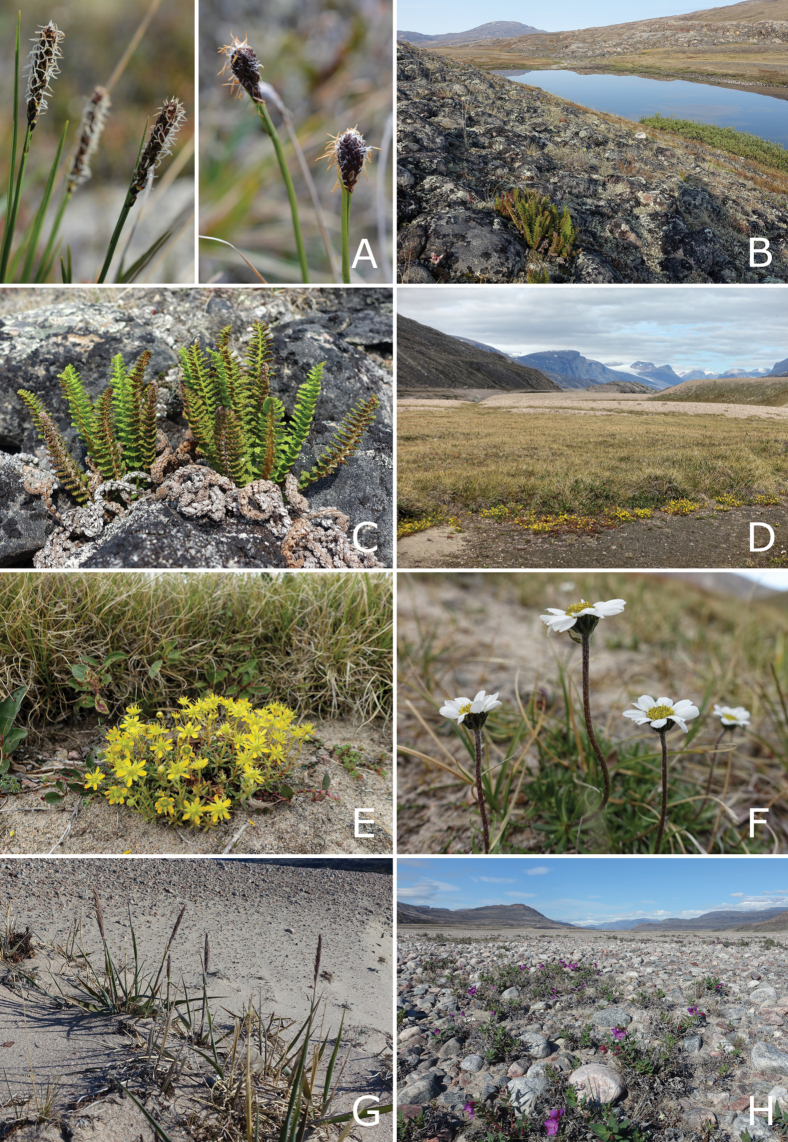
Species found only at the heads of the largest fiords in east-central Baffin Island. A. Carex scirpoidea subsp. scirpoidea, pistillate left (Gillespie et al. 11832), staminate right (Gillespie et al. 11917), Kangiqtualuk Agguqti; B. *Dryopteris fragrans*, habitat (Gillespie et al. 12414), on rocky slope above pond in large valley at head of Kangiqtualuk Agguqti (site 21.1); C. *Dryopteris fragrans*, habit, Kangiqtualuk Uqquqti, July 30, 2021; D. *Saxifraga aizoides*, habitat (Gillespie et al. 11963), along the edge of a dried pond on a terrace in a large valley at the head of Kangiqtualuk Agguqti (site 8.5); E. *Saxifraga aizoides*, habit (Gillespie et al. 11963); F. *Hulteniella integrifolia*, habit (Gillespie et al. 11958), in a small side valley on river terrace at head of Kangiqtualuk Agguqti (site 8.4); G. Leymus mollis subsp. villosissimus, habit (Gillespie et al. 11911), on sand bank of terrace at edge of delta at head of Kangiqtualuk Agguqti (site 8.1); H. Poa hartzii subsp. hartzii, habitat (Gillespie et al. 11729), growing with *Chamaenerion latifolium* on sand-cobble river delta at head of Kangiqtualuk Uqquqti (site 30.6). Photos by P.C. Sokoloff (A) and L.J. Gillespie (B–H).

We recorded 154 taxa from the heads of the four largest fiords with well-inventoried heads (Kangiqtualuk Agguqti and Kangiqtualuk Uqquqti in Agguttinni TP, and Kangiqtugaapik Head and Inugsuin Head in the Clyde area), representing 91% of the total number of taxa in the entire flora area. Of these, 37 taxa were found only here. Five of them were found at all four fiord heads (Carex scirpoidea subsp. scirpoidea [Fig. 12A], *Dryopteris fragrans* [Fig. 12B, C], *Physaria arctica*, Potentilla arenosa subsp. arenosa, *Saxifraga aizoides* [Fig. 12D, E]). Six were found only at Kangiqtugaapik Head (*Crucihimalaya bursifolia* [Fig. 9A], *Draba oblongata*, *Erigeron compositus* [Fig. 9B], *Puccinellia vahliana*, *Ranunculus trichophyllus*, and *Saxifraga hirculus*), and one each only at Kangiqtualuk Agguqti (*Carex marina*) and Kangiqtualuk Uqquqti (*Festuca baffinensis* [Fig. 9G]); no species were recorded only from Inugsuin Head. Three taxa were found only at the two large fiord heads in Agguttinni TP (*Arenaria humifusa*, *Carex holostoma*, *Hulteniella integrifolia* [Fig. 12F]), and six only in the Clyde area (Antennaria media subsp. compacta, *Draba glabella*, *Hippuris lanceolata*, *Juncus arcticus*, *Potentilla × prostrata*, *P. pedersenii*, *Woodsia glabella*). Five taxa were present only at Kangiqtualuk Agguqti and Kangiqtugaapik Head (*Calamagrostis purpurascens*, *Eriophorum brachyantherum*, Leymus mollis subsp. villosissimus [Fig. 12G], Potentilla arenosa subsp. chamissonis, *Ranunculus arcticus*), two at Kangiqtualuk Uqquqti and Kangiqtugaapik Head (Equisetum variegatum subsp. variegatum, Poa hartzii subsp. hartzii [Fig. 12H]), five at Kangiqtugaapik Head and both fiord heads in Agguttinni TP (Carex simpliciuscula subsp. subholarctica, *Draba arctica*, *Eriophorum callitrix*, *Sabulina stricta*, *Tofieldia pusilla*, *Woodsia alpina*), and one at Kangiqtualuk Agguqti and both Clyde area fiord heads (*Potentilla subgorodkovii*).

Including the heads of three smaller fiords in the Home Bay area (Ekalugad Head, Kangirlugag Fiord, Rocknoser Fiord), we recorded 160 taxa from the heads of fiords (94% of the total number of taxa in the flora area), an increase of six species. Three of the additional species were found only in the Home Bay area (Eriophorum vaginatum subsp. spissum, *Harrimanella hypnoides*, *Ranunculus sabinei*), but of these, only Eriophorum vaginatum subsp. spissum was restricted to a Home Bay area fiord head. The other three species were also found farther north outside fiord heads (*Cherleria biflora*, *Deschampsia brevifolia*, *Epilobium arcticum*). The remaining fiord head localities were not well collected and added no additional species.

Seventy-four photograph-documented observations of vascular plants from the flora area were recorded in iNaturalist: 14 from Clyde River, four from Cape Christian (Clyde River vicinity), 31 from Coutts Inlet, and 25 from the Buchan Gulf area (Suppl. material 6). No species new to the flora area were documented. All species based on photograph-documented observations from Clyde River and vicinity, and the majority from Coutts Inlet, were already documented by cited collections. Three species (*Luzula nivalis*, *Saxifraga cernua*, *Vaccinium uliginosum*) are newly recorded from Coutts Inlet and are included in Table 3. No collections are known from the Buchan Gulf area; therefore, all taxa observed here are new records for this area (Suppl. material 6). These include seven species from Feachem Bay, Buchan Gulf (Cassiope tetragona subsp. tetragona, *Pedicularis hirsuta*, Poa arctica subsp. arctica, *Pyrola grandiflora*, *Salix arctica*, *Salix herbacea*, *Salix reticulata*); five from Icy Arm, Quernbiter Fiord (Cassiope tetragona subsp. tetragona, *Oreomecon labradorica*, *Pyrola grandiflora*, *Saxifraga tricuspidata*, Stellaria longipes subsp. longipes); and four from Rannoch Arm, Cambridge Fiord (Dryas integrifolia subsp. integrifolia, *Pyrola grandiflora*, *Saxifraga tricuspidata*, Stellaria longipes subsp. longipes). Six potential new records could not be verified due to poor-quality images or diagnostic features not being visible. All new localities for species based on verified iNaturalist observations are mentioned in the notes under the appropriate species in the annotated checklist with the iNaturalist citation provided.

Six previous floristic treatments included east-central Baffin Island, although five of them encompassed larger areas and one a much smaller area (Hainault 1966, three localities) (see Suppl. materials 7–10). As expected, the number of reported species has increased over time as the region has been more thoroughly explored: 47 species in Taylor (1863), 64 in Polunin (1940), 139 in Porsild (1957), 150 in Porsild and Cody (1980), and 166 in Aiken et al. (2007). However, determining the true number of species in each treatment and whether they validly reported any species we did not find is difficult for two reasons. First, the two earliest treatments, Taylor (1863) and Polunin (1940), did not give specific localities for many of the common species they reported, so it is impossible to tell whether these species were thought to be in our flora area. Second, and more importantly, these treatments almost never cited voucher collections for their records. The only exception is Polunin (1940), who cited specific collections for only nine taxa (for many others he provided a literature reference or gave only the collector’s name and sometimes the year), and we were able to verify vouchers for six of them (*Carex bigelowii*, *Dupontia fisheri*, *Festuca brachyphylla*, *Poa arctica*, Potentilla hyparctica subsp. hyparctica, *Salix arctica*); vouchers for the remaining three taxa were either reidentified or not found. Thus, reconciling changing taxonomic concepts, updating potential misidentifications, and correcting mapping errors (the three most recent treatments gave localities only as dot maps) are challenging. Fortunately, most map dots in Aiken et al. (2007), the most recent of the treatments, can be connected to a specimen via their unpublished mapping spreadsheets. Taking into account 18 taxa that we exclude (see Excluded taxa and Suppl. materials 7, 8), Aiken et al. (2007) reported 148 taxa (145 species, two with two subspecies each, and one nothospecies), 21 fewer taxa (18 species, two additional subspecies, and one nothospecies) than we report here.

In our paper on the flora of Agguttinni TP (Gillespie et al. 2024), we provided voucher collections for 144 vascular plant taxa (141 species, three subspecies); all taxa were vouchered for the first time except for the six species vouchered by Polunin (1940) listed above; 143 taxa and 140 species are accepted here (see discussion under *Draba corymbosa*). Here we document with voucher collections an additional 27 taxa in the flora area, including the 23 taxa unique to the Clyde and Home Bay areas listed previously, plus the following four taxa found only in both Clyde and Home Bay areas: Carex glareosa subsp. glareosa, *Carex subspathacea*, Arnica angustifolia subsp. angustifolia, and *Salix arctophila*.

The 18 species we document as new to the flora area are *Arabidopsis arenicola*, *Arenaria humifusa*, *Carex holostoma*, *Carex marina*, *Deschampsia brevifolia*, *Draba subcapitata*, Eriophorum vaginatum subsp. spissum, *Hulteniella integrifolia*, *Oreomecon labradorica*, *O. lapponica*, Poa abbreviata subsp. abbreviata, *Potentilla pedersenii*, *P. subgorodkovii*, *P. subvahliana*, *Puccinellia bruggemannii*, *Ranunculus trichophyllus*, *Taraxacum holmenianum*, and *Vaccinium vitis-idaea*. We also newly document two subspecies (Potentilla arenosa subsp. arenosa, Saxifraga rivularis subsp. arctolitoralis) and one nothospecies (*Potentilla × prostrata*). Most of these taxa were collected during our 2021 Agguttinni TP fieldwork and first recorded in Gillespie et al. (2024). Three species (Eriophorum vaginatum subsp. spissum, *Potentilla pedersenii*, and *Ranunculus trichophyllus*) are based on previous collections from the Clyde and Home Bay areas and newly reported here for the flora area (note that *P. pedersenii* was mistakenly mentioned in the discussion in Gillespie et al. (2024) based on previous preliminary determinations but is not known from Agguttinni TP). The new records of *Potentilla* and *Oreomecon* species are mostly due to recent taxonomic updates and the recognition of multiple species previously included within a single species.

## ﻿﻿Discussion

**Figure 13.**
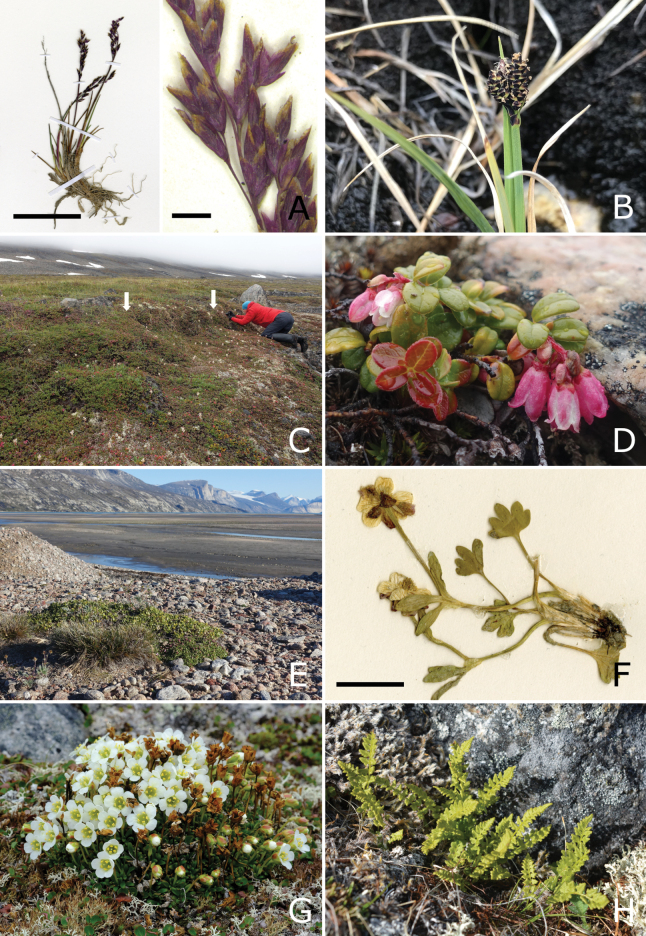
New distribution records. A. *Puccinellia bruggemannii*, habit (left) and inflorescence (right) (Gillespie et al. 12193 [CAN 10114935]), Kuugaaluk; B. *Carex holostoma*, habit (Gillespie et al. 11871), Kangiqtualuk Agguqti; C. *Vaccinium vitis-idaea*, habitat (Gillespie et al. 12275), Arviqtujuq Kangiqtua (white arrow indicates plant in habitat), *Pyrola grandiflora*, and *Vaccinium uliginosum* are common in the foreground; D. *Vaccinium vitis-idaea*, habit (Gillespie et al. 12275), Arviqtujuq Kangiqtua; E. Poa abbreviata subsp. abbreviata, habit/habitat (Gillespie et al. 11899), growing with *Honckenya peploides* on stony terrace above delta at head of Kangiqtualuk Agguqti; F. *Ranunculus sabinei* (Webber 1230 [CAN 10052156]), Ekalugad Fiord; G. *Diapensia lapponica*, habit (Gillespie et al. 11794), Kangiqtualuk Agguqti; H. *Woodsia alpina*, habit (Gillespie et al. 11896), Kangiqtualuk Agguqti. Photos by P.C. Sokoloff (A, B, F, H) and L.J. Gillespie (C–E, G). Scale bars: 2 cm (A left, F), 2 mm (A right).

We report a total of 169 taxa (163 species, four subspecies, and two nothospecies) in east-central Baffin Island. This represents an increase of 13% from the 148 taxa previously recorded and/or mapped in this area in the most recent flora covering the region (Aiken et al. 2007), based on current species taxonomy and excluding erroneous reports. The 21 taxa reported as new include one species new to Baffin Island (*Puccinellia bruggemannii* [Fig. 13A]), two northernmost records for Canada (*Carex holostoma* [Fig. 13B], *Vaccinium vitis-idaea* [Fig. 13C, D]), and one northernmost record for eastern Canada (*Ranunculus trichophyllus*). Other notable newly reported taxa include the high Arctic species Poa abbreviata subsp. abbreviata (Fig. 13E) (easternmost and among the southernmost records for Baffin Island), *Arabidopsis arenicola* (Fig. 9E), and *Arenaria humifusa* (both among the northernmost records in Canada).

The flora area also includes noteworthy species that were previously mapped in Aiken et al. (2007) but not remarked upon there. The three collections of *Ranunculus sabinei* (Fig. 13F), the only records from Baffin Island, have a remarkably disjunct distribution, isolated by over 1000 km from other localities in the species’ range. The two collections of *Crucihimalaya bursifolia* (Fig. 9A) are also the only ones known from Baffin Island and are isolated from the closest populations in western Greenland by over 500 km. East-central Baffin Island collections of *Diapensia lapponica* (Fig. 13G) and *Harrimanella hypnoides* are the northernmost records for Canada, those of *Arctous alpina* (Fig. 9D) the northernmost in eastern Canada, and those of *Salix arctophila* and *Tofieldia pusilla* among the northernmost in eastern Canada. Collections of the southern Arctic species *Woodsia alpina* (Fig. 13H), previously recorded only by Polunin (1940) based on Taylor (1863), are among the northernmost records in Canada. Our Clark Fiord collection of *Sagina caespitosa*, first reported here, is the northernmost record in Canada.

All vascular plant taxa recorded in east-central Baffin Island are native. The circumarctic, as a whole, has a relatively low number of non-native species, estimated at 8% of the total flora, including both stabilized (= naturalized) and casual introductions (Daniëls et al. 2013). Stabilized introduced species are most frequent in the low Arctic, as expected, and none have yet been recorded from the high Arctic subzones A and B. Only a single non-Arctic species (*Barbarea vulgaris* W.T.Aiton) has been recorded as a stabilized introduction in the mid-Arctic subzone C, in which east-central Baffin Island is located, and only on Svalbard in the European Arctic (Daniëls et al. 2013). Few stabilized introduced species have been reported from Arctic Canada, mostly from low Arctic mainland areas (Wasowicz et al. 2019). Although several non-native species have been recorded from the Canadian Arctic Islands, all or most are casual introductions found in the vicinity of communities; it is unknown if any have become stabilized (Aiken et al. 2007; Gillespie et al. 2015; Saarela et al. 2020a, 2020b, 2023).

Many taxa appear to be rare in the flora area; 33 are known from only 1–3 collections and 45 from only one or two localities. However, none are considered to be of conservation concern in Canada, and only two species (*Crucihimalaya bursifolia*, *Woodsia alpina*) are considered vulnerable in Nunavut (Canadian Endangered Species Conservation Council 2022). Although some Canadian Arctic species are indeed rare, very few are under threat since the native vegetation is, for the most part, intact with minimal, if any, impact from human disturbance or invasive species. Only a single Arctic vascular plant species is listed as a species at risk in Canada (*Braya pilosa* Hook. in mainland Arctic Northwest Territories) (https://laws.justice.gc.ca/eng/acts/s-15.3/page-10.html, https://laws.justice.gc.ca/PDF/S-15.3.pdf), with a highly restricted distribution threatened by coastal erosion aggravated by climate change.

Many of the species that are rare in the flora area have widespread distributions in the North American Arctic or circumarctic. In general, Arctic species tend to be fairly widely distributed, with few having highly restricted distributions (Hultén 1962, 1971). Only three taxa are endemic to the Canadian Arctic Islands, and all are subspecies in *Braya* (B. humilis subsp. ellesmerensis J.G. Harris, B. glabella subsp. prostrata J.G. Harris, and B. thorild-wulffii subsp. glabrata J.G. Harris; the latter two are sometimes not considered as distinct taxa), and none occur in the flora area (Harris 2006; Gillespie et al. 2015). No taxon is considered to be endemic to Baffin Island.

The floristic composition of east-central Baffin Island is similar overall to other areas in the Arctic and Arctic Canada, with mostly angiosperms, few lycophytes and ferns, and no conifers (few sometimes present in subzone A). Five of the six largest families in terms of species diversity in the flora area—Cyperaceae, Poaceae, Brassicaceae, Caryophyllaceae, and Asteraceae (ordered by decreasing size)—are among the eight largest families in the circumarctic flora, and four of the five largest genera—*Carex*, *Potentilla*, *Draba*, and *Ranunculus* (ordered by decreasing size)—are among the six largest genera in the circumarctic (Daniëls et al. 2013). Compared with other well-sampled areas in the Canadian Arctic Islands, the flora area has the same six largest families (including Saxifragaceae in addition to those listed above) as Victoria Island in subzones C and D (Saarela et al. 2020b) and Dorset and Mallik Islands, off the southwest coast of Baffin Island, in subzone C (Saarela et al. 2020a), and four of the five largest families as the area encompassing Katannilik Territorial Park and Kimmirut and vicinity on southern Baffin Island in subzone D (Saarela et al. 2023). Compared with areas in mainland Arctic Canada, the flora area has five of the six largest families (excluding Saxifragaceae) in common with Tuktut Nogait Territorial Park and vicinity in subzones D and E (Saarela et al. 2013) and the lower Coppermine River valley in subzone E and the adjacent forest–tundra ecozone (Saarela et al. 2017). *Carex* is consistently the largest genus in the above-mentioned Canadian Arctic areas, and *Draba*, *Potentilla*, and *Saxifraga* are among the largest. *Salix*, the second largest genus in the circumarctic, has low diversity in east-central Baffin Island compared with the above-mentioned Canadian Arctic areas (5 versus 8–16 taxa), consistent with the flora area’s more northerly latitude and/or colder subzone. This finding is in agreement with a dramatic northward decrease in *Salix* species in the circumarctic, from 72 species in subzone E to two species in subzone A (Daniëls et al. 2013).

Of the taxa found in east-central Baffin Island, 58% are broadly circumpolar in distribution, whereas only 6% are restricted to North America. Among the remaining taxa, more are shared with Asia (15%; 17% including those that also occur in Svalbard and/or Iceland) than with Europe (8%). This might seem counterintuitive since Baffin Island is considerably closer to Europe than to Asia; however, North America and Asia were connected by a land bridge multiple times during the Pleistocene (Farmer et al. 2023), facilitating plant dispersal, whereas no land connection existed between North America and Europe during this period. Baffin Island’s flora, as in most of the Canadian Arctic, is recent, since the region was completely covered by ice sheets multiple times during the Pleistocene. Deglaciation along the coast of east-central Baffin Island following the last glacial maximum started about 15,000 years ago (Miller et al. 2005).

The majority of vascular plant taxa both in the global Arctic and in the flora area are not restricted to the Arctic; most taxa also occur in adjacent alpine and/or boreal areas, and some also extend to temperate areas. Daniëls et al. (2013) recorded 106 vascular plant taxa endemic to the Arctic, representing about 5% of the total Arctic flora of 2218 taxa. Of these endemic taxa, 12 are found in east-central Baffin Island, representing 7% of the flora: five are circumpolar in distribution (*Draba oblongata*, *Pleuropogon sabinei*, Potentilla hyparctica subsp. hyparctica, *Puccinellia angustata*, and Silene uralensis subsp. arctica), three are endemic to North America (*Potentilla vahliana*, *Puccinellia bruggemannii*, *Taraxacum holmenianum*), three are found in North America and Europe (*Cerastium arcticum*, *Draba arctica*, *Puccinellia vahliana*), and one is in Beringia and North America (*Silene sorensenis*). However, the list compiled by Daniëls et al. (2013) was based on a preliminary distributional dataset from Elven et al. (2011) and requires updating. For example, Poa hartzii subsp. hartzii is now considered an Arctic endemic; its recorded rare presence in the boreal zone appears to be based on misidentifications. More collections-based studies are needed to more precisely and accurately determine distributions of taxa occurring in the Arctic.

Total species richness recorded in the four main areas of east-central Baffin Island is correlated with collection intensity. The Clyde area is the most species-rich (153 taxa) and the most intensively collected (1708 collections), whereas only 35 taxa were recorded in Coutts Inlet, the least well-collected area (52 collections). Almost as many taxa (143) were recorded in Agguttinni TP despite being based on many fewer collections (952) than the Clyde area. This suggests that species discovery may have levelled out in localities surveyed in the Clyde area, at least at Kangiqtugaapik Head, the most well-collected locality (870 collections). The relatively high number of taxa compared with collection intensity in Agguttinni TP may also be due to the higher number of localities surveyed (21 versus nine), representing a greater diversity of vegetation types and habitats. In Agguttinni TP, with helicopter assistance, we were able to survey more widely and more easily target possible areas of botanical interest, although we had more limited time at each locality (ranging from hours to 6 days compared with 66 days of collecting at Kangiqtugaapik Head by members of the Baird expedition). Compared with four localities visited (but only one reasonably well surveyed) on the interior plateau and uplands in Agguttinni TP, located in the Baffin Island Uplands ecoregion, only one site in this ecoregion has been surveyed in the Clyde area, and none in the other two main areas. Fewer coastal lowland sites were surveyed in the Clyde area than in Agguttinni TP, which may account for several species (*Puccinellia bruggemannii*, Tripleurospermum maritimum subsp. phaeocephalum, *Vaccinium vitis-idaea*) found in Agguttinni TP but not in the Clyde area. Targeting specific habitats in Agguttinni TP led to the discovery of several species not found in other areas, such as *Arabidopsis arenicola* on shifting sand in an interior valley. With more intensive collecting at a greater number of localities representing all ecoregions, we anticipate that more taxa will be discovered in both Agguttinni TP and the Clyde area and many more taxa in the Coutts Inlet and Home Bay areas.

We found that the number of collections for a species and how widespread it is are strongly and positively correlated (R^2^ = 0.855; Fig. 11). This result is not surprising, assuming that all species are collected about the same number of times at each locality where they are found. Still, it is interesting to look at those taxa that appear to be over- or under-collected based on their distribution. Although we have no objective measure of local abundance, our subjective assessments of abundance suggest that abundance alone does not explain over- or under-collection. For example, *Draba lactea* and Potentilla hyparctica subsp. hyparctica, both widespread species, are seldom abundant at a site, yet they were collected more times than expected. In contrast, Cassiope tetragona subsp. tetragona, a dominant species at many sites, was not among the most commonly collected taxa (i.e., those with > 50 collections). Instead, we suggest two other factors are involved. The first is that species that are difficult to identify in the field are collected more frequently at a locality, whereas those that are easy to identify are collected once at most. *Carex*, *Draba*, and *Potentilla* species are notoriously difficult to identify, and many botanists (including our team) tend to collect them whenever they see them, at least until they develop a good feel for the more common species. However, easy-to-identify species, such as Cassiope tetragona subsp. tetragona, were often collected only once per locality, or not at all if time was limited. Another factor is how conspicuous a species is. For example, *Pedicularis hirsuta* was the second most widespread species but was not among the most frequently collected species, nor was it abundant at any site. Since it is a conspicuous, showy species, it would be more likely to be collected, especially by non-botanists, than inconspicuous species such as many graminoids. Indeed, *P. hirsuta* was collected at seven localities visited entirely by non-botanists. *Salix* species present a unique situation because they are dioecious; hence, many botanists collect both sexes as separate collections, inflating the number of collections relative to how widespread they are.

Species diversity in east-central Baffin Island (169 taxa) was higher than expected for a local flora in subzone C (75–150) (Young 1971; CAVM Team 2003). Compared with other well-surveyed Canadian Arctic areas in subzone C, diversity was higher than on Dorset and Mallik Islands (153 taxa), a much smaller but more southern area (Saarela et al. 2020a). Diversity is also higher than in the subzone C portion of Victoria Island (157), but considerably lower than on Victoria Island as a whole, which includes both subzones C and D (280) (Saarela et al. 2020b). The concept of local floras, as applied to bioclimatic subzones by Young (1971), is not well defined, but based on examples provided, it may be more comparable to a main area, or perhaps to a large locality as used here, than to the entire east-central Baffin Island area. Species diversity in Agguttinni TP (143) and Home Bay (96) is within the expected range, while the Clyde area (153) has a slightly higher diversity than expected (Coutts Inlet has not been adequately sampled for comparison). The five most well-collected localities all fall within the expected local flora range for subzone C: Kangiqtugaapik Head (141), Inugsuin Head (109), Kangiqtualuk Agguqti (109), Kangiqtualuk Uqquqti (98), and Clyde River (78).

The Baffin Mountains ecoregion has an overall higher species diversity (162 taxa) than the Baffin Island Coastal Lowlands (110) and Baffin Island Uplands (66) ecoregions in the flora area. Although much of the Baffin Mountains ecoregion consists of barren steep slopes, ridges, peaks, and glaciers with very low species diversity, numerous sheltered valleys have much higher diversity. Much of the diversity in this ecoregion occurs in the four comprehensively sampled large fiord heads (Inugsuin Head, Kangiqtugaapik Head, Kangiqtualuk Agguqti [Fig. 2F], Kangiqtualuk Uqquqti [Fig. 2H]), together accounting for 154 taxa, representing 95% of the flora in the ecoregion and 91% of the total flora. Including the heads of five smaller fiords in the Home Bay area (Ekalugad Head, Kangirlugag Fiord, Kangok Fiord, Nudlung Fiord, and Rocknoser Fiord) and one in Agguttinni TP (Gibbs Fiord [Fig. 2E]), all less well collected, brings the total to 160 taxa (99% in the ecoregion, 94% in the flora area). Forty-seven taxa were found only at the heads of fiords, with 37 only at the heads of the four largest fiords. The only large shrub found in the flora area, *Salix richardsonii* (Fig. 2G), is primarily restricted to this zone, with few small plants observed in the Baffin Island Uplands ecoregion.

The remarkably high species diversity at the heads of these four fiords may be attributed to a warmer climate along with high habitat diversity. Their sheltered inland, low-elevation locations result in sunnier, warmer, and drier summers compared to the surrounding mountains and to the coastal lowlands, which are cooler, wetter, and often shrouded in fog. These localities are large river valleys with large deltas, extensive glacioalluvial terraces, rocky valley slopes, and numerous side valleys (Figs 2F–H, 4A, D). Habitat diversity is high, ranging from coastal sandy habitats, river and terrace banks, lake, pond, and stream margins, and wet sedge meadows to dry stony terraces and rocky outcrops (Figs 12B, D, H, 13E). Among the 37 species found only in these fiord heads in the flora area are species at or near their northern limits, at least in eastern Canada (e.g., *Arenaria humifusa*, *Carex holostoma* [Fig. 13B], *Eriophorum brachyantherum*, *Tofieldia pusilla*); species more characteristic of the high and western Arctic (*Erigeron compositus* [Fig. 9B], Poa hartzii subsp. hartzii [Fig. 12H], both preferring calcareous substrates); and diverse habitat specialists. The latter include *Leymus mollis* (Fig. 12G) on unstable coastal sand, *Hippuris lanceolata* and *Ranunculus trichophyllus* in freshwater ponds, and *Physaria arctica* on dry stony calcareous flats and slopes. *Carex marina* and *Saxifraga aizoides* (Fig. 12D, E), both preferring wet mossy sites, were found growing at the same single site along the margin of a recently dried-out pond in late summer. All four fern species were recorded in the large fiord heads; three were found only here (*Dryopteris fragrans* [Fig. 12B, C], *Woodsia alpina* [Fig. 13H], *W. glabella*).

Other localities in the Baffin Mountains ecoregion were much less diverse than the fiord head localities. These include small side valleys off large fiords, medium-sized valleys connecting large fiords, and valleys in land-locked fiords (Figs 2A, B, D). Many of these localities are considerably closer to the coast and thus influenced by cooler, wetter coastal weather. Only three were reasonably well surveyed: Atagulisaktalik (69 taxa), Cape Hooper (58), and Tingijattut (52), all within subzone C local flora diversity levels. Although Cape Hooper is located on the coast, it is considered part of the Baffin Mountains rather than the Coastal Lowlands ecoregion. Including it here added no additional species but would have added four species to the latter ecoregion. The only species unique to this ecoregion outside of the fiord heads was *Arabidopsis arenicola* (Fig. 9E) from Tasialuk S, a long interior valley at the head of a land-locked fiord. This is a habitat specialist found on unstable sand on the steep side of a valley, similar to those at fiord heads.

The Baffin Island Coastal Lowlands ecoregion contains 65% of the total taxa recorded in the flora area, intermediate in species diversity between the Baffin Mountains and Baffin Uplands ecoregions. It is considerably smaller in size than the other two ecoregions. Five localities were reasonably well collected in this ecozone: Clyde River (78 taxa), Ekalugad FOX-C (58), Inugsuin Mouth (59), Kuugaaluk (56), and Refuge Harbour (50). All have diversity levels within the range expected for ecozone C, mostly at the lower end. Although individual localities were not very species diverse, the ecoregion includes interesting coastal and riparian habitats not found elsewhere in the flora area. Only four species and one nothotaxon were found exclusively in this ecoregion. Tripleurospermum maritimum subsp. phaeocephalum (Fig. 9H) was found only once in a wet depression along a rocky shoreline just above the high tide mark. *Puccinellia bruggemannii* (Fig. 13A) was found in flat tundra near the top of coastal bluffs. Only one small population of *Vaccinium vitis-idaea* was discovered, on a low bank between south-facing sloping benches covered in dense heath tundra (Fig. 13C, D).

The Baffin Uplands ecoregion is the least diverse ecoregion, with only 39% of the total species diversity in the flora area. This large ecoregion was the least sampled, with only five localities surveyed and only two with over 20 collections (Generator Lake, 52; McBeth Valley, 50). The low collection intensity is at least in part due to low diversity. All collections were made by botanical experts, collecting over a period of 9 days at Generator Lake and 7 days at McBeth Valley. The other three localities were visited by us for only one to several hours, and our focus was on collecting species we had not already collected in the uplands. Total species diversity within the ecoregion (66 taxa) and at the two most collected localities (38, 44) was lower than expected for subzone C. Lower diversity would be expected at higher elevations in the Arctic. Most of the collecting sites were between 400 and 550 m elevation (all those in Agguttinni TP). Although not as high as the subzone C mountain equivalent of subzone B (lower limit of 650 m), elevation would certainly have an effect on both vegetation and species diversity. Species diversity of the ecoregion, but not the Generator Lake and McBeth Valley localities, was within the range expected for subzone B. This ecoregion was also deglaciated more recently than the Coastal Lowlands and lower elevation areas in the Baffin Mountains ecoregion, thus allowing less time for substrate and flora development. Two species were recorded only in this ecoregion. *Draba subcapitata*, found only at Generator Lake, is a species more characteristic of the high and western Arctic, while *Arctous alpina* (Fig. 9D), collected only at McBeth Valley, is a more southerly species that reaches its northern limit in eastern Canada here. McBeth Valley in the Clyde area is an atypical locality in this ecoregion; the site is in a long interior valley at an elevation of approximately 80–100 m, much lower than typical for the ecoregion, with mountains rising to over 600 m on either side. Thirteen taxa were recorded only here in the ecoregion, including numerous species common elsewhere in the flora area (e.g., Arctagrostis latifolia subsp. latifolia, Carex bigelowii subsp. bigelowii [Fig. 10G], *Carex nardina*, *Pyrola grandiflora* [Fig. 10H], *Saxifraga tricuspidata*, *Vaccinium uliginosum*) and some more typical of the low Arctic (*Arctous alpina*, *Diapensia lapponica* [Fig. 13G], *Rhododendron lapponicum*, Rhododendron tomentosum subsp. decumbens). Excluding this locality decreased species diversity in the ecoregion to 53 taxa. Obviously more comprehensive collections from throughout this ecoregion are needed; while species diversity levels will likely increase somewhat, we anticipate levels will remain relatively low.

iNaturalist records contributed to our knowledge, especially in areas where no or few collections have been made. Observations were likely made primarily by passengers travelling by ship, allowing access to these very difficult-to-reach areas. However, species recorded are primarily the common conspicuous species. Photos were sometimes of poor quality and did not show the features necessary for accurate identification.

We have shown that reliance on previous floras and other publications can be problematic. Here, we exclude 25 species recorded as present in east-central Baffin Island in previous floras (Taylor 1863; Polunin 1940; Porsild 1957; Porsild and Cody 1980; Aiken et al. 2007). Many of these records were based on specimens that have since been redetermined, others were mapping errors, and at least one is likely a label error. The problem is compounded by the frequent lack of specimen citations; tracking down the specimens on which these records are based is sometimes impossible. Some dubious records in Taylor (1863) seem to have been uncritically cited or mapped in later floras. We regard some of Taylor’s records as erroneous, as was also noted by Porsild and Porsild (1920) for the Greenland flora. However, some of the species we excluded because no specimen records could be found are known from elsewhere on Baffin Island and should be looked for in the flora area (e.g., *Anthoxanthum arcticum*, *Astragalus alpinus*, *Campanula rotundifolia*, *Carex microglochin*).

Two taxa included here based on single older collections from Clyde River should also be confirmed with additional collections. The presence of Eriophorum × medium subsp. album, based on a Polunin collection from 1936, is somewhat dubious given that neither parent taxon has been collected at Clyde River. Polunin had an unusual method of collecting, pressing numerous species on a single sheet with minimal information, leading to possible errors in collection label data. *Ranunculus sulphureus* (Fig. 9C), collected by Martin in 1958, is a relatively conspicuous species, and it is therefore surprising that it has not been recollected at this well-collected locality.

This study has substantially increased our knowledge of the diversity and distribution of the vascular plant flora of east-central Baffin Island and represents a new foundation on which continued botanical exploration of the area can build. This is the first study to completely document the flora with voucher herbarium specimens; previous studies have not, or have only minimally, cited collections, which has led to difficulties verifying the presence of numerous species. Documentation allows future researchers to build easily on our study as new collections and other data sources become available. The data presented here show that additional species continue to be found in the flora area. Furthermore, vast areas of east-central Baffin Island remain botanically unexplored or only minimally so, in particular the interior uplands and the large fiord complexes between Coutts Inlet and Agguttinni TP. Thus, we expect that future work will further expand our understanding of the flora of east-central Baffin Island. Our results also provide important baseline data for monitoring and understanding the impact of climate change on the flora of the Arctic, an area of the world that is disproportionately affected.

## ﻿﻿Annotated checklist

The vascular plant flora of east-central Baffin Island is summarized as an annotated checklist. Classification of lycophytes and ferns follows the Pteridophyte Phylogeny Group (2016). Angiosperm families follow the classification of the Angiosperm Phylogeny Group (2016) and are ordered alphabetically within monocots and eudicots. Genera are listed alphabetically within families, and species are listed alphabetically within genera. We provide synonyms for species and subspecies, focusing on names previously used in critical Canadian Arctic floras (Porsild 1957; Porsild and Cody 1980; Aiken et al. 2007) and panarctic floras (Elven et al. 2011). English common names follow Brouillet et al. (2010) and/or Flora of North America Editorial Committee (1993+). For global distribution we mostly follow Elven et al. (2011); distributions of several taxa were updated based on new information. For genera with multiple taxa, we provide keys to species and subspecies, which also show the characters we used for identification.

We provide notes for many species, such as those that are rare and otherwise of interest, including new records for the flora area and other regions, and distributional information. Species distributions in the Canadian Arctic are based both on the literature and on herbarium specimens that we verified, including specimens at the Canadian Museum of Nature and from Global Biodiversity Information Facility (GBIF)-mediated herbarium collection data. We accepted records discovered on GBIF only when we could confirm a specimen determination or make a new determination based on an image. We also provide taxonomic notes for taxa whose taxonomy has recently changed or is controversial.

Localities recorded in the literature or mapped in Canadian Arctic floras (Porsild 1957; Porsild and Cody 1980; Aiken et al. 2007) for which no collections were seen (and may not exist) are mentioned in the species notes. Several mapping errors in Aiken et al. (2007) are noted; these were detected by examining the unpublished digital mapping database associated with the flora. We are not aware of the locations of any specimen lists or file cards used in the production of maps in Porsild (1957) and Porsild and Cody (1980), and it was therefore not possible to detect mistakes in the placement of dots on maps in these floras, nor was it possible to be certain about the identity of specimens used for mapping. The majority of localities mapped could be matched with specimens; those that could not be matched are discussed. Species listed in Taylor (1863) as occurring at Cape Adair and Scott’s Inlet for which no collections were found are mentioned in the notes when there is little or no doubt as to the identification. We mention iNaturalist records only when no collections exist to document a species at a locality.

All identified and confirmed collections are cited in the *Specimens examined* sections. Collections are organized alphabetically by the four main areas used for the floristic analyses, then by locality and site (Table 1). For all collections, the primary collector, collection number, herbarium acronym (following Thiers, 2025 [continuously updated]), and, where present, herbarium barcode or accession number are cited. Collections by Malte do not include collection numbers but instead have unique six-digit numbers from a previous National Herbarium of Canada series; these numbers are cited in square brackets. The small herbarium at Nunavut Parks and Special Places in Iqaluit, Nunavut, is not an officially recognized herbarium and is cited here as “npsp.” Herbarium barcodes are given for specimens from ASC, BR, CAN, COLO, F, MEL, MIN, O, TRH, US, and ZT, whereas herbarium accession numbers are cited for specimens from ACAD, ALA, BABY, CM, H, MT, QK, RBCM, S, and UBC. For specimens from DAO and QFA, barcodes are cited if available; otherwise, accession numbers are given. Specimens at CHARS and npsp have neither accession numbers nor barcodes. When a specimen comprises multiple taxa, the specimen is cited pro parte, and the relevant plants are indicated by letters or numbers that appear on the specimen.

For further details on species present in Agguttinni TP, including habitat and ecology, Inuktitut names and traditional uses, French common names, brief descriptions, and identification features, see Gillespie et al. (2024). Photographs of plant habit and/or close-ups are also included there for the majority of species.

### ﻿﻿Lycophytes

**Lycopodiaceae**

#### ﻿*Huperzia* Bernh.

##### *Huperzia arctica* (Grossh. ex Tolm.) Sipliv. (≡Lycopodium selago subsp. arcticum Tolm.)—Arctic firmoss—Circumpolar?

We recognize *H. arctica* as separate from boreal *H. selago* (L.) Bernh. ex. Schrank *&* Mart. (≡*Lycopodium selago* L.) following Elven et al. (2011), Dignard (2013), Gilman and Testo (2015), and Saarela et al. (2020b).

**Specimens examined: Agguttinni TP.** Atagulisaktalik: site 22.1, *Gillespie et al. 11467* (CAN 10112557, US). Clark Fiord: site 20.5, *Gillespie et al. 12350* (CAN 10112565). Generator Lake: site GL-21, *Raynolds & Bültmann MKR-2022-55* (CAN 10169288). Kangiqtualuk Agguqti: site 3.1, *Gillespie et al. 11796* (CAN 10112561); site 5.3, *Gillespie et al. 11866* (ALTA, CAN 10112558); site 8.7, *Gillespie et al. 11980* (CAN 10112559). Kangiqtualuk Uqquqti: site 28.1, *Gillespie et al. 11634* (CAN 10112563, MT). Kuugaaluk: site 19.1, *Gillespie et al. 12287* (ALA, CAN 10112567, CHARS); site A22.2, *Gillespie et al. 12451* (CAN 10112566, WIN). Niaqurnaaluk-Qassialuit: site 18.2, *Gillespie et al. 12244* (CAN 10112560, QFA). Refuge Harbour: site 11.2, *Gillespie et al. 12021* (CAN 10112564, npsp). Tingijattut: site 15.2, *Gillespie et al. 12128* (CAN 10112562). **Clyde Area.** Clyde River: site CR-1, *Polunin 634* (CAN 10003917), *Dutilly 1434* (QFA0010709, QFA0013466, QFA0131112); site CR-2, *Forbes 59* (DAO 01-01001123385), *Forbes 121* (DAO 01-01001123377). Hoffman Cove: site HF-1, *Bartlett 217* (US 01380536). Inugsuin Head: site IG-1, *Hainault 3675* (CAN 10003913, O V545027, QK 59692). Kangiqtugaapik Head: site KP-1, *Wynne-Edwards 8871* (CAN 10003896), *Dansereau 500608-0751* (MT00286484), *500628-5992* (MT00286486), *500530-0555* (MT00286485); site KP-6, *Wynne-Edwards 9051* (CAN 10003895). McBeth Valley: site MB-1, *Hainault 3758* (QK 59522). **Home Bay.** Cape Hooper: site HO-1, *Elven 3103/99* (O V545023), *3305/99* (ALA V132322), *3441/99* (CAN 10003862, O V545022). Ekalugad FOX-C: site FC-1, *Richardson & Webber 62* (CAN 10003707). Kangirlugag Fiord: site KG-1, *Webber 1190* (CAN 10003704, QK 71130).

### ﻿﻿Monilophytes—ferns

**Cystopteridaceae**

#### ﻿*Cystopteris* Bernh.

##### *Cystopteris fragilis* (L.) Bernh.—Fragile fern—Cosmopolitan

Also recorded from Scott’s Bay (Taylor 1863), but no collection located.

**Specimens examined: Agguttinni TP.** Clark Fiord: site 20.1, *Gillespie et al. 12341* (CAN10112569). Kangiqtualuk Agguqti: site 21.4, *Gillespie et al. 12422* (CAN 10112572, MT, npsp). Kangiqtualuk Uqquqti: site 28.1, *Gillespie et al. 11642* (CAN 10112571); site 27.1, *Gillespie et al. 11615b* (CAN 10112570, CHARS). Stewart Valley: site 15.1, *Gillespie et al. 12111* (CAN 10112568). **Clyde Area.** Inugsuin Head: site IG-1, *Hainault 4032* (QK 59537); site IG-9, *Parmelee & Seaborn 3934* (DAO 01-01425578); site IG-5, *Parmelee & Seaborn 3972* (DAO 01-01425563). Kangiqtugaapik Head: site KP-2, *Martin 45* (DAO 01-014255574); site KP-16, *Wynne-Edwards 8936* (CAN 10005330); site KP-18, *Wynne-Edwards 9049* (CAN 10005331).

#### ﻿Dryopteridaceae

***Dryopteris* Adans.**

##### *Dryopteris fragrans* (L.) Schott (Fig. 12B, C)—Fragrant wood fern—European (NE)–Asian–amphi-Beringian–North American (N)

**Specimens examined: Agguttinni TP.** Kangiqtualuk Agguqti: site 21.1, *Gillespie et al. 12414* (CAN 10112573); site 6.5, *Gillespie et al. 11894* (CAN 10112574, npsp). Kangiqtualuk Uqquqti: site 27.1, *Gillespie et al. 11622* (CAN 10112575). **Clyde Area.** Inugsuin Head: site IG-20, *Hainault 3626* (CAN 10005883, DAO 01-01401893, O V545110, QK 59595); site IG-5, *Parmelee & Seaborn 3963* (DAO 01-01401890); site IG-26, *Hainault 4026* (DAO 01-01401892, O V545109, QK 59830). Kangiqtugaapik Head: site KP-1, *Dansereau 500530-0399* (DAO 01-01401887), *500531-0399* (MT00194666), *500628-5754* (MT00194664), *500629-2199* (MT00194665, MT00199281), *500710-5099* (MT00194663), *500731-0751* (MT00194661), *500809-0666* (MT00194662), *Wynne-Edwards 8810* (CAN 10005884); site KP-2, *Martin 44* (DAO 01-01401891).

#### ﻿Equisetaceae

***Equisetum* L.**

##### Key to species of *Equisetum* [adapted from Porsild and Cody (1980) and Saarela et al. (2020b)]

**Table:** 

1	Stems annual (persisting one year or less), bearing branches in whorls; cones terminal on brown unbranched reproductive stems or green branched stems	**E. arvense subsp. alpestre**
–	Stems evergreen (persisting more than one year), unbranched or forking, lacking branches in whorls; cones terminal on green stems	**E. variegatum subsp. variegatum**

##### Equisetum arvense L. subsp. alpestre (Wahlenb.) Schönsw. & Elven—Alpine field horsetail—Circumpolar-alpine

We follow Schönswetter et al. (2001), Elven and Murray (2008), and Elven et al. (2011) in recognizing arctic-alpine Equisetum arvense subsp. alpestre as a distinct subspecies (sometimes incorrectly called subsp. boreale (Bong.) Tolm.). Compared to the more southerly distributed subsp. arvense , subsp. alpestre has prostrate to ascending shoots with slender, mostly 4-ribbed branches and strobili mostly less than 15 mm long. Taylor (1863) recorded *E. arvense* from Scott’s Bay (presumably subsp. alpestre), but no collection was located.

**Specimens examined: Agguttinni TP.** Atagulisaktalik: site 24.6, *Gillespie et al. 11555* (CAN 10112555, CHARS, MO, npsp, NY). Generator Lake: site GL-16, *Raynolds & Bültmann MKR-2022-29* (CAN 10169260). Kuugaaluk: site 16.5, *Gillespie et al. 12211* (ALA, CAN 10112554, CHARS, MT, npsp); site 19.4, *Gillespie et al. 12303* (ALTA, CAN 10112551), *Gillespie et al. 12307* (CAN 10112552, MT, npsp). **Clyde Area.** Clyde River: site 10.3, *Gillespie et al. 12002* (CAN 10112553, QFA). Inugsuin Head: site IG-1, *Hainault 3702* (QK 59645); site IG-14, *Parmelee & Seaborn 3901* (DAO 01-01243109, DAO 01-01334896); site IG-26, *Hainault 3677* (O V545046, QK 59704); site IG-27, *Hainault 4033* (CAN 10004602, DAO 01-01334899, O V545053, QK 59758). Inugsuin Mouth: site IM-1, *Hainault 3910* (DAO 01-01334900, O V545045, QK 59812). Kangiqtugaapik Head: site KP-1, *Wynne-Edwards 8813* (CAN 10004294), *Dansereau 500708-0652* (MT00286632), *500803-0152* (MT00286631), *500803-0286* (MT00286630), **Home Bay.** Cape Hooper: site HO-1, *Elven 3477/99* (CAN 10004279, O V545038). Ekalugad FOX-C: site FC-1, *Richardson & Webber 13* (QK 71131), *50* (CAN 10004596). Kangirlugag Fiord: site KG-1, *Webber 1315* (CAN 10004595).

##### Equisetum variegatum Schleich. subsp. variegatum—Variegated scouring-rush—Circumpolar-boreal–cool North Temperate

This widespread species is known from only two localities in the flora area. Porsild (1957) and Porsild and Cody (1980) mapped an additional locality from the Clyde area, possibly from Clyde River, but no specimen was found to verify this locality.

**Specimens examined: Agguttinni TP.** Kangiqtualuk Uqquqti: site 28.1, *Gillespie et al. 11625* (CAN 10112556, npsp). **Clyde Area.** Kangiqtugaapik Head: site KP-1, *Wynne-Edwards 8937* (CAN 10001245); site KP-18, *Wynne-Edwards 9052* (CAN 10001246).

#### ﻿Woodsiaceae

***Woodsia* R.Br.**

##### Key to species of *Woodsia* [adapted from Windham (1993)]

**Table:** 

1	Fronds with scattered hairs or scales on the undersurface (mostly on the rachis), petioles reddish brown near the base, pinnae ovate to triangular, longer than wide	***W. alpina***
–	Fronds completely glabrous, petioles green or pale-colored throughout, pinnae fan-shaped, usually wider than long	***W. glabrata***

##### *Woodsia alpina* (Bolton) Gray (Fig. 13H)—Alpine woodsia—Circumpolar-alpine

This is primarily a low Arctic species. Collections cited here from the flora area are among the northernmost records in Canada, together with one confirmed record from Ellesmere Island (*Murray & Yurtsev 10254*CAN). The only previous record of the species in the flora area was by Polunin (1940), who cited Scott’s Bay and also Cape Searle southeast of the flora area, both based on Taylor (1863). Given that no specimens have been located for verification, our records could be the first for the flora area. Porsild (1957) and Porsild and Cody (1980) mapped a collection from the east coast just southeast of the flora area, perhaps corresponding to the Cape Searle locality, but no collections were located. Elsewhere in the Canadian Arctic Islands, *Woodsia alpina* is known only from southern Baffin, Coates, and Southampton islands (Porsild 1957; Porsild and Cody 1980; Aiken et al. 2007; Saarela et al. 2023). It is currently considered vulnerable in Nunavut (Canadian Endangered Species Conservation Council 2022).

**Specimens examined: Agguttinni TP.** Kangiqtualuk Agguqti: site 7.1, *Gillespie et al. 11896* (CAN 10112577); site 21.3, *Gillespie et al. 12421* (CAN 10112576, npsp). Kangiqtualuk Uqquqti: site 27.1, *Gillespie et al. 11615a* (CAN 10112578). **Clyde Area.** Kangiqtugaapik Head: site KP-1, *Dansereau 500701-1551* (MT00286572).

##### *Woodsia glabella* R.Br.—Smooth woodsia—Circumpolar-alpine

Also recorded from Scott’s Bay (Taylor 1863), but no collection located.

**Specimens examined: Clyde Area.** Inugsuin Head: site IG-1, *Hainault 3740* (CAN 10005063, DAO 01-01384089, O V545128, QK 59616); site IG-5, *Parmelee & Seaborn 3971* (DAO 01-01384094), *3980c* (DAO 01-01384084). Kangiqtugaapik Head: site KP-1, *Dansereau 500628-6188* (MT00286575), *500715-0594* (MT00286573), *500804-0951* (MT00286576); site KP-18, *Wynne-Edwards 8823* [pro parte] (CAN 10005069), *8892* (CAN 10005068), *9048* (CAN 10005059); site KP-20, *Wynne-Edwards 8990* (CAN 10005058).

### ﻿﻿Angiosperms—flowering plants

**Monocots**

﻿**Cyperaceae**

#### *Carex* L.

##### Key to species of Carex [adapted from Porsild and Cody (1980), Ball (2002), Ball and Reznicek (2002), Toivonen (2002), and Saarela et al. (2020b)]

**Table:** 

1	Margins of perigynium open; terminal and distal spikelets usually 1-flowered, staminate; proximal spikelets 1-flowered and pistillate, or 2–4-flowered and bisexual with 1 pistillate flower proximally and 1–3 staminate flowers distally	**2**
–	Margins of perigynium fused; all spikelets 1-flowered	**3**
2	Inflorescences compound, (2–)3–8 mm wide, spike ovoid-oblong; basal leaf sheaths dull, base of blade usually persistent	**C. simpliciuscula subsp. subholarctica**
–	Inflorescences simple, 2–3 mm wide, spike linear; basal leaf sheaths somewhat glossy, bladeless	***C. myosuroides***
3	Spikes solitary	**4**
–	Spikes compound	**7**
4	Stigmas 2; plants densely cespitose	**5**
–	Stigmas 3; plants cespitose or loosely cespitose	**6**
5	Spikes gynecandrous (staminate flowers proximal, pistillate flowers distal); perigynia 1.5–2 mm long; plants of seashores	***C. ursina***
–	Spikes androgynous (pistillate flowers proximal, staminate flowers distal); perigynia 3–5 mm; plants of dry, exposed tundra	***C. nardina***
6	Plants dioecious, rarely monoecious; rhizomes short, sometimes inconspicuous; perigynia hairy	**C. scirpoidea subsp. scirpoidea**
–	Plants monoecious, spike androgynous; rhizomes long; perigynia glabrous	***C. rupestris***
7	Spikes all bisexual and sessile	**8**
–	Spikes mostly unisexual, the terminal one staminate or bisexual, spikes pedunculate or sessile	**10**
8	Some or all spikes androgynous, plants rhizomatous	***C. maritima***
–	Some or all spikes gynecandrous, plants cespitose	**9**
9	Plants loosely cespitose; culms erect, 10–15(–30) cm long; spikes 2–3(–4); lateral spikes gynecandrous, containing 3–8 perigynia, oblong-clavate; perigynia elliptic, green-white proximally, pale brown distally, often brown in age; beak indistinct	***C. marina***
–	Plants densely cespitose; culms often arching, weak, 10–25 cm long; spikes 2–4; lateral spikes pistillate, containing 5–10(–15) perigynia, oblong-linear; perigynia broadly elliptic-obovate to lanceolate, light to pale brown, often gray-brown at maturity; beak short	**C. glareosa subsp. glareosa**
10	Stigmas 2	**11**
–	Stigmas 3	**14**
11	Perigynia somewhat glossy, glabrous, more or less inflated, beak distinct; lateral spikes usually pendant	***C. saxatilis***
–	Perigynia dull, usually papillose, sometimes glabrous, not inflated, beak indistinct; lateral spikes erect	**12**
12	Pistillate scales with a prominent, scabrous awn on at least the proximal scales; leaf blades involute, 1–2 mm wide	***C. subspathacea***
–	Pistillate scales with apex acute, acuminate, or mucronate, lacking a prominent, scabrous awn; leaf blades not involute, the widest > 2 mm wide	**13**
13	Proximal bract longer than inflorescence (usually at least 1.5 × as long)	**C. aquatilis subsp. stans**
–	Proximal bract shorter than or equal to inflorescence	**C. bigelowii subsp. bigelowii**
14	Terminal spike gynecandrous	**C. fuliginosa subsp. misandra**
–	Terminal spike staminate	**15**
15	Inflorescences capitate, the lateral spikes short-peduncled	**16**
–	Inflorescences not capitate	**17**
16	Terminal spike often prominent above lateral, spreading pistillate spikes; pistillate spikes few-flowered; pistillate scales chestnut brown with wide hyaline margins, acuminate; perigynia brown	**C. supina subsp. spaniocarpa**
–	Terminal spike often hidden between lateral, erect pistillate spikes; pistillate spikes many-flowered; pistillate scales dark brown to black with very narrow hyaline margins, mucronate; perigynia green, black on apical half	***C. holostoma***
17	Pistillate spikes sessile or nearly sessile	***C. membranacea***
–	Pistillate spikes on peduncles as long as or longer than the spikes	**18**
18	Pistillate scales black with pale midvein, ovate or oblong-ovate, 3–4.8 × 0.9–1.6 mm; lateral spikes 4–7 mm wide; terminal spike 6–15 × 2–5 mm, usually over-topping lateral spikes, sometimes overlapping some of them	***C. atrofusca***
–	Pistillate scales medium to dark brown with pale midvein, ovate, obovate, or obovate-circular, 2.3–3.5 × 0.8–1.2 mm; lateral spikes 3–4 mm wide; terminal spike 4–10 × 0.7–1.4 mm, level with or over-topped by some of the lateral spikes	**C. capillaris subsp. fuscidula**

##### Carex aquatilis Wahlenb. subsp. stans (Drejer) Hultén (≡*Carex stans* Drejer)—Arctic water sedge—Circumpolar-alpine

Also recorded from Scott’s Bay (Taylor 1863), but no collection located.

**Specimens examined: Agguttinni TP.** Atagulisaktalik: site 24.2, *Gillespie et al. 11546* (CAN 10112601, MT). Clark Fiord: site 20.2, *Gillespie et al. 12352b* (ALA, CAN 10112606, CHARS, NY). Generator Lake: site GL-17, *Raynolds & Bültmann MKR-2022-61* (CAN 10169271). Kangiqtualuk Agguqti: site 3.1, *Gillespie et al. 11811* (CAN 10112603, npsp, QFA, US); site 8.7, *Gillespie et al. 11974* (CAN 10112602, MT). Kangiqtualuk Uqquqti: site 29.1, *Gillespie et al. 11684* (ALA, ALTA, CAN 10112604, MO). Refuge Harbour: site 11.1, *Gillespie et al. 12013* (CAN 10112600, NY, UBC, WIN). Tasialuk S: site 26.6, *Gillespie et al. 11603* (CAN 10112605, npsp). **Clyde Area.** Clyde River: site CR-1, *Dutilly 9385* (MT00176818), *Martin 42* (DAO 01-01667582), *Polunin 644* (CAN 10022109), *2599* (CAN 10022110); site CR-2, *Forbes 2* (DAO 01-01667584), *30* (DAO 01-01667583), *105* (DAO 01-01667581), *118* (DAO 01-01667585). Inugsuin Head: site IG-7, *Parmelee & Seaborn 3926* (DAO 01-01667587, DAO 01-01667633); site IG-30, *Hainault 4058* (CAN 10022111, H 1300179, O V546517, QK 59739). Inugsuin Mouth: site IM-1, *Hainault 3845* (H 1300183, O V546514, QK 59655), *3863* (CAN 10021934, DAO 01-01667586, H 1300181, O V546515, QK 59794). Kangiqtugaapik Head: site KP-1, *Dansereau 500624-0463* (MT00178671), *500625-0259* (MT00178657), *500627-2365* (MT00178664, MT00183488), *500630-2064* (MT00183489), *500630-2164* (MT00178667), *500630-2291* (MT00178666), *500702-0662* (MT00178674), *500702-0865* (MT00183477), *500709-0868* (DAO 01-01667580, MT00178662, MT00183490), *500713-0254* (MT00178673), *500716-0271* (MT00178668), *500716-0453* (MT00178663, MT00183495), *500721-0152* (MT00178659, MT00183484), *500801-0451* (MT00178656, MT00183498), *500810-1865* (MT00178655), *500822-0669* (MT00178672, MT00183492), *500827-0764* (MT00178669), *500827-0855* (MIN 1276412, MT00178665), *500830-3955* (MT00178658), *Wynne-Edwards 8963* (CAN 10022099), *8973* (CAN 10022102), *9021* (CAN 10022103); site KP-5, *Wynne-Edwards 9022* (CAN 10021942); site KP-9, *Dansereau 500805-0584* [pro parte, plants B] (MT00286099); site KP-28, *Dansereau 500604-0394* (MT00178661, MT00183486). McBeth Valley: site MB-1, *Hainault 3750* (DAO 01-01667632, H 1300182, O V546516, QK 59586). **Home Bay.** Ekalugad FOX-C: site FC-1, *Richardson & Webber 45* (COLO 01021559, QK 71080). Kangirlugag Fiord: site KG-1, *Webber 1218* (CAN 10021921, COLO 01018365, QK 71081), *1276* (CAN 10021928 [pro parte, plants a], COLO 01018407 [pro parte, plants a], QK 71082).

##### *Carex atrofusca* Schkuhr—Dark-brown sedge—Circumpolar-alpine

**Specimens examined: Agguttinni TP**. Kangiqtualuk Agguqti: site 6.1, *Gillespie et al. 11875* (CAN 10112610, MO, npsp, NY); site 8.5, *Gillespie et al. 11967* (CAN 10112611, US). Kangiqtualuk Uqquqti: site 28.5, *Gillespie et al. 11666* (CAN 10112607). Tasialuk S: site 26.6, *Gillespie et al. 11602* (ALA, ALTA, CAN 10112609, MT). Tingijattut: site 15.8, *Gillespie et al. 12176* (CAN 10112608, CHARS). **Clyde Area.** Inugsuin Head: site IG-7, *Parmelee & Seaborn 3914* (DAO 01-01667650 [pro parte, plants 1], DAO 01-01667651). Kangiqtugaapik Head: site KP-1, *Dansereau 500702-0660* (MT00286773, MT00286774), *500711-4253A* (MT00286762), *500713-0253* (MT00286771, MT00286772), *500713-0457* (MT00286764), *500713-1062* (MT00286765, MT00286766), *500731-0276A* (MT00286761), *500801-0457A* (MT00286763), *500827-0853* (MT00286767, MT00286768), *500827-1357* (DAO 01-01667652, MT00286769, MT00286770), *Wynne-Edwards 8925* (CAN 10022324), *9036* (CAN 10022325); site KP-5, *Wynne-Edwards 8984* (CAN 10022332).

##### Carex bigelowii Torr. in Schwein. subsp. bigelowii (Fig. 10G)—Bigelow’s sedge—North American (N)–amphi-Atlantic

**Specimens examined: Agguttinni TP.** Atagulisaktalik: site 22.1, *Gillespie et al. 11463* (CAN 10112599, MT); site 22.2, *Gillespie et al. 11485* (CAN 10112592, MT); site 23.8, *Gillespie et al. 11523* (ALTA, CAN 10112593, NY); site 23.10, *Gillespie et al. 11534* (CAN 10112579, MO); site AT-2, *Dansereau 500725-0572* (MT00176801); site AT-5, *Röthlisberger s.n.* (CAN 10020831). Clark Fiord: site 20.1, *Gillespie et al. 12332* (CAN 10112587); site 20.2, *Gillespie et al. 12352a* (CAN 10112594, MT). Kangiqtualuk Agguqti: site 3.1, *Gillespie et al. 11816* (ALA, ALTA, CAN 10112595, CHARS, MO); site 8.2, *Gillespie et al. 11922* (CAN 10112584). Kangiqtualuk Uqquqti: site 1.1, *Gillespie et al. 11779* (CAN 10112598, MT, npsp, NY). Kuugaaluk: site 16.2, *Gillespie et al. 12201* (CAN 10112597, npsp); site 19.2, *Gillespie et al. 12294* (ALA, CAN 10112591, NY, QFA); site A22.3, *Gillespie et al. 12461* (CAN 10112596). Refuge Harbour: site 11.1, *Gillespie et al. 12007* (CAN 10112583, QFA, US, WIN). Stewart Valley: site 15.1, *Gillespie et al. 12095* (CAN 10112582, MO, NY, UBC), *Gillespie et al. 12096* (CAN 10112586, CHARS, QFA, US). Tasialuk N: site 26.1, *Gillespie et al. 11578* (ALA, CAN 10112588, CHARS, MT, NY, QFA); site 26.2, *Gillespie et al. 11580* (CAN 10112580, MO, WIN). Tasialuk S: site 26.5, *Gillespie et al. 11600* (CAN 10112585). Tingijattut: site 15.2, *Gillespie et al. 12136* (CAN 10112581, MT); site 15.6, *Gillespie et al. 12169* (ALTA, CAN 10112589); site TG-2, *Dare et al. 78* (CAN 10092307). **Clyde Area.** Clyde River: site CR-1, *Dutilly 9381* (CM412169, MT00176817, US 02105198), *Malte s.n.* [118524] (CAN 10020825, MICH 1363409), *s.n.* [118525] (US 02105201), *s.n.* [118528] (CAN 10020807), *s.n.* [118535] (CAN 10020808); site CR-2, *Forbes 43* (DAO 01-01667578), *136* (DAO 01-01667576). Hoffman Cove: site HF-1, *Bartlett 225* (US 02259121). Inugsuin Head: site IG-30, *Hainault 4051* (CAN 10020684, DAO 01-01667579, H 1300928, O V546571, QK 59729). Inugsuin Mouth: site IM-1, *Hainault 3835* (DAO 01-01667575, H 1300925, O V546573, QK 59686), *3859* (CAN 10020686, H 1300927, O V546574, QK 59791). Kangiqtugaapik Head: site KP-1, *Dansereau 500702-0458* (MT00176805), *500704-58* (MT00286984), *500707-0255* (MT00176811), *500711-4253* (MT00286982, MT00286983), *500713-0661* (MT00176812, MT00183487), *500714-0251* (MT00185812), *500720-0155* (MT00176807, MT00176815), *500720-0251* (MT00176808, MT00176813), *500720-0660* (MT00176806), *500723-0867* (MT00176810), *500723-1076* (MT00185813), *500726-1375* (MT00176823, MT00183478), *500728-0572* (MT00176824), *500801-0456* (MT00183479), *500803-0864* (MT00176809), *500827-1169* (MT00176802), *500830-3852* (MT00176804), *500830-3954* (MT00176803); site KP-2, *Martin 48* (DAO 259623); site KP-5, *Dansereau 500828-0563* (DAO 01-01667577, MIN 1281338, MT00176800, MT00176814), *Wynne-Edwards 9034* (CAN 10020675); site KP-15, *Dansereau 500713-0870* (MT00176799, MT00183485); site KP-25, *Wynne-Edwards 9073* (CAN 10020816). McBeth Valley: site MB-1, *Hainault 3777* (DAO 834138, H 1300926, O V546572, QK 59670). **Coutts Inlet.** Coutts Fiord: site CF-1, *Coombs 59* (DAO 01-01667631). **Home Bay.** Cape Hooper: site HO-1, *Elven 3466/99* (ALA V132080, CAN 10020651, O V546598), *Hammar 3493/33* (O V546597). Ekalugad FOX-C: site FC-1, *Richardson & Webber 24* (CAN 10020666, COLO 01021518, QK 71079); site FC-2, *Oswald 349* (QK 135640), 367 (QK 135630); site FC-3, *Oswald 347* (QK 135639); site FC-6, *Oswald 357* (QK 135641). Ekalugad Head: site EK-1, *Church 6* (COLO 01021492), *13* (COLO 01021500); site EK-4, *Philpot et al. s.n.* (COLO 01021534). Kangirlugag Fiord: site KG-1, *Webber 1276* (CAN 10021928 [pro parte, plants b], COLO 01018407 [pro parte, plants b]), *1318* (CAN 10020655, COLO 01095892). Rocknoser Fiord: site RF-1, *Smith VP-96-61* (CAN 10020664), *Webber 1191* (CAN 10020819, COLO 01021542, QK 71078).

##### Carex capillaris L. subsp. fuscidula (V.I.Krecz. ex T.V.Egorova) Á.Löve & D.Löve—Dusky-spike sedge—Circumpolar-alpine

**Specimens examined: Agguttinni TP.** Atagulisaktalik: site 23.10, *Gillespie et al. 11532* (ALTA, CAN 10112618, MO, MT, npsp, NY). Gibbs Fiord: site 20.9, *Gillespie et al. 12378* (CAN 10114758). Kangiqtualuk Agguqti: site 3.2, *Gillespie et al. 1182*1 (CAN 10112614), *Gillespie et al. 11833* (ALA, CAN 10112617, NY); site 8.2, *Gillespie et al. 11923* (CAN 10112615, UBC); site 21.1, *Gillespie et al. 12411* (CAN 10114759, MT). Kangiqtualuk Uqquqti: site 27.1, *Gillespie et al. 11619* (ALA, CAN 10112612, CHARS). Stewart Valley: site 15.1, *Gillespie et al. 12101* (CAN 10112616). Tasialuk S: site 26.5, *Gillespie et al. 11601* (CAN 10112613). Tingijattut: site 15.9, *Gillespie et al. 12183* (CAN 10112619, US). **Clyde Area.** Inugsuin Head: site IG-1, *Hainault 3708* (O V546643, QK 59639),

*3722* (CAN 10036514, O V546641, QK 59606); site IG-25, *Hainault 4024* (DAO 01-01667567, O V546642). Kangiqtugaapik Head: site KP-1, *Dansereau 500709-1468* (MT00286559, MT00286560), *500723-0860* (MT00286557, MT00286558), *500801-0366* (MT00286562), *500805-0483* (MT00286563); site KP-18, *Wynne-Edwards 8903* (CAN 10036504).

##### Carex fuliginosa Schkuhr subsp. misandra (R.Br.) Nyman (≡*Carex misandra* R.Br.)—Short-leaved sedge—Circumpolar-alpine

**Specimens examined: Agguttinni TP.** Atagulisaktalik: site 23.6, *Gillespie et al. 11516* (CAN 10112621, npsp); site AT-1, *Dansereau 500723-1079* (MT00286965, MT00286966). Clark Fiord: site 20.5, *Gillespie et al. 12351* (CAN 10112628, NY). Generator Lake: site GL-12, *Raynolds & Bültmann MKR-2022-48* (CAN 10169273). Kangiqtualuk Agguqti: site 3.1, *Gillespie et al. 11812* (ALA, ALTA, CAN 10112623, CHARS, MO); site 8.5, *Gillespie et al. 11968* (CAN 10112624). Kangiqtualuk Uqquqti: site 27.1, *Gillespie et al. 11612* (CAN 10112620, MT, UBC). Niaqurnaaluk-Qassialuit: site 18.2, *Gillespie et al. 12248* (CAN 10112627, npsp). Refuge Harbour: site 11.2, *Gillespie et al. 12025* (ALA, CAN 10112625, QFA). Stewart Valley: site 15.1, *Gillespie et al. 12105* (CAN 10112622, MT). Swiss Bay: site SB-2, *Starr 08-220* (CAN 10036872). Tingijattut: site 15.2, *Gillespie et al. 12135* (ALTA, CAN 10112626, US). **Clyde Area.** Cape Hewett: site CH-1, *Platt 486A* (NY 2291729). Clyde River: site CR-1, *Dutilly 9383* (CM409782, US 02125250), *Malte s.n..* [118482] (CAN 10037047), *Polunin 132* (US 02125148), *231* (F V0378589F), *369* (MIN 1382119), *463* (MIN 1382118), *536* (US 02125127), *737* (F V0378607F), *773* (F V0378601F), *871* (US 02125147); site CR-2, *Forbes 41A* (DAO 01-01667571), *94* (DAO 01-01667570), *137* (DAO 01-01667569). Inugsuin Head: site IG-1, *Hainault 4025* (CAN 10037054, DAO 01-01667568, H 1306157, O V546712, QK 59726); site IG-7, *Parmelee & Seaborn 3914* (DAO 01-01667650 [pro parte, plants 2]). Inugsuin Mouth: site IM-1, *Hainault 386*0 (CAN 10037057, H 1306155, O V546711, QK 59792). Kangiqtugaapik Head: site KP-1, *Dansereau 500731-0276* (MT00286961, MT00286962), *Wynne-Edwards 9035* (CAN 10037051), site KP-5, *Wynne-Edwards 8983* (CAN 10037059), site KP-18, *Wynne-Edwards 8902* (CAN 10037058), site KP-20, *Wynne-Edwards 8989* (CAN 10041087). McBeth Valley: site MB-1, *Hainault 3774* (DAO 01-01667630, H 1306156, O V546710, QK 59668). **Coutts Inlet.** Coutts Fiord: site CF-1, *Coombs 58* (DAO 01-01667629). **Home Bay.** Cape Hooper: site HO-1, *Elven 3437/99* (CAN 10037050, O V546697). Ekalugad FOX-C: site FC-1, *Richardson & Webber 47* (CAN 10036998, COLO 01046937, QK 71074). Kangirlugag Fiord: site KG-1, *Webber 1261* (CAN 10037056, COLO 01046879, QK 71075). Nudlung Fiord: site NF-1, *Ryder s.n.* (COLO 01046853). Rocknoser Fiord: site RF-1, *Smith VP-108-61* (CAN 10037000).

##### Carex glareosa Wahlenb. subsp. glareosa (=Carex glareosa var. amphigena Fernald)—Gravel sedge—Circumpolar

**Specimens examined: Clyde Area.** Inugsuin Head: site IG-1, *Hainault 3995* (CAN 10087320, H 1303818, O V546750, QK 59559). Inugsuin Mouth: site IM-1, *Hainault 3828* (CAN 10087318, DAO 01-01667563, DAO 01-01667564, H 1303819, O V546751, QK 59680). Kangiqtugaapik Head: site KP-1, *Wynne-Edwards 8919* (CAN 10087336). **Home Bay.** Rocknoser Fiord: site RF-1, *Smith VP-109-61* (CAN 10087330).

##### *Carex holostoma* Drejer (Fig. 13B)—Arctic marsh sedge—Circumpolar?

This species is newly reported for the flora area here and in Gillespie et al. (2024). These collections, along with one from the west coast of Baffin Island (*Burt s.n.*CAN 10089113), are the northernmost known Canadian records of this low Arctic species.

**Specimens examined: Agguttinni TP.** Kangiqtualuk Agguqti: site 5.4, *Gillespie et al. 11871* (CAN 10112630, CHARS, MT, npsp); site 8.7, *Gillespie et al. 11976* (CAN 10112629, NY). Kangiqtualuk Uqquqti: site 28.4, *Gillespie et al. 11664* (CAN 10112631).

##### *Carex marina* Dewey (=*Carex amblyorhyncha* V.I.Krecz.) (Fig. 9F)—Sea sedge—Circumpolar-alpine

This species is newly reported for the flora area here and in Gillespie et al. (2024). It has a scattered distribution across the Canadian Arctic, mostly absent from the colder subzones A and B (Porsild 1957; Porsild and Cody 1980; Aiken et al. 2007; Saarela et al. 2020b). The single population observed was growing with *Saxifraga aizoides* along the margin of a dried-out pond (Fig. 12D).

**Specimens examined: Agguttinni TP.** Kangiqtualuk Agguqti: site 8.5, *Gillespie et al. 11965* (CAN 10112632, npsp).

##### *Carex maritima* Gunnerus—Seaside sedge—Circumpolar-alpine

**Specimens examined: Agguttinni TP.** Kangiqtualuk Agguqti: site 8.1, *Gillespie et al. 11902* (CAN 10112633, CHARS, MT). Kangiqtualuk Uqquqti: site 1.2, *Gillespie et al. 11781a* (CAN 10112634); site 30.5, *Gillespie et al. 11724* (ALTA, CAN 10112635). Tasialuk S: site 21.12, *Gillespie et al. 12439* (ALA, CAN 10112636, npsp, NY). **Clyde Area**. Clyde River: site CR-1, *Dutilly 1432* (US 02124300). Kangiqtugaapik Head: site KP-1, *Dansereau 500604-0253* (MT00107903, MT00107905), 500710-3751 (DAO 01-01662630), *Wynne-Edwards 8933* (CAN 10127846); site KP-2, *Martin 50* (DAO 01-01662629, RBCM V138401); site KP-4, *Dansereau 500711-5657* (MT00107908, MT00107909); site KP-10, *Dansereau 500827-0151* (MT00107906, MT00107907); site KP-22, *Dansereau 500703-3751* (MT00107902, MT00107904).

##### *Carex membranacea* Hook.—Fragile sedge—Amphi-Beringian–North American (N)

Also recorded from Scott’s Bay (as *Carexcompacta R.Br.*) (Taylor 1863), but no collection located.

**Specimens examined: Agguttinni TP.** Atagulisaktalik: site 22.2, *Gillespie et al. 11491* (ALA, ALTA, CAN 10112638, CHARS, MT); site 24.2, *Gillespie et al. 11547* (CAN 10112639, US); site AT-1, *Dansereau 500723-1078* (MT00286927). Gibbs Fiord: site 20.10, *Gillespie et al. 12388* (CAN 10114729, MT, NY). Kangiqtualuk Agguqti: site 3.1, *Gillespie et al. 11814* (CAN 10114724, CHARS, MT, npsp, NY, UBC, US); site 8.2, *Gillespie et al. 11924* (ALTA, CAN 10112640, MT). Kangiqtualuk Uqquqti: site 28.1, *Gillespie et al. 11637* (ALA, CAN 10112637, npsp, NY). Kuugaaluk: site 19.1, *Gillespie et al. 12291* (ALTA, CAN 10114727, MO, WIN). Refuge Harbour: site 12.7, *Gillespie et al. 12054* (CAN 10114725, MO, US, WIN). Tasialuk N: site 26.4, *Gillespie et al. 11583* (CAN 10114728, MO, QFA). Tingijattut: site 15.2, *Gillespie et al. 12143* (ALA, CAN 10114726, NY, QFA, UBC). **Clyde Area.** Clyde River: site CR-1, *Dutilly 1456* (CM409626), *9382* (US 02124521), *Malte s.n..* [118532] (CAN 10037941, GH barcode-01774588), *Martin 37* (DAO 01-01667595); site CR-2, *Forbes 138* (DAO 01-01667596). Inugsuin Head: site IG-1, *Hainault 4011* (QK 59538), *4012* (CAN 10037927, DAO 01-01667593, H 1305829, O V546885, QK 59746); site IG-6, *Parmelee & Seaborn 3869* (DAO 01-01667594); site IG-26, *Hainault 4034* (DAO 01-01667592, H 1305830, O V546887, QK 59759), *4035* (CAN 10038022, H 1300180, O V546513, QK 59760); site IG-30, *Hainault 4057* (CAN 10037926, H 1305831, O V546886, QK 59738). Kangiqtugaapik Head: site KP-1, *Dansereau 500701-0453* (MT00286940, MT00286941), *500822-0767* (MT00286928, MT00286939), *Wynne-Edwards 8926* (CAN 10038018), *9037* (CAN 10037964); site KP-18, *Wynne-Edwards 8906* (CAN 10037963). **Home Bay.** Kangirlugag Fiord: site KG-1, *Webber 1283* (CAN 10037924, COLO 01044767). Nudlung Fiord: site NF-1, *Ryder s.n.* (COLO 01044783). Rocknoser Fiord: site RF-1, *Smith VP-98-61* (CAN 10037967).

##### *Carex myosuroides* Vill. (≡ *Kobresia myosuroides* (Vill.) Fiori)—Mouse-tail bog sedge—Circumpolar-alpine

**Specimens examined: Agguttinni TP.** Atagulisaktalik: site 23.10, *Gillespie et al. 11533* (CAN 10114730, WIN); site AT-2, *Dansereau 500725-0464* (MT00202111, MT00202127). Clark Fiord: site 20.1, *Gillespie et al. 12333* (CAN 10114737, UBC). Kangiqtualuk Agguqti: site 3.5, *Gillespie et al. 11850* (CAN 10114732, CHARS, NY); site 8.2, *Gillespie et al. 11928* (CAN 10114733, MO, QFA). Kangiqtualuk Uqquqti: site 29.2, *Gillespie et al. 11686* (ALTA, CAN 10114731, MT, npsp). Kuugaaluk: site 19.4, *Gillespie et al. 12302* (CAN 10114736, MT). Refuge Harbour: site 12.6, *Gillespie et al. 12053* (ALA, CAN 10114734). Stewart Valley: site 15.1, *Gillespie et al. 12099* (CAN 10114735, US). **Clyde Area.** Inugsuin Head: site IG-1, *Hainault 3993* (CAN 10034750, O V547263, QK 59802); site IG-6, *Parmelee & Seaborn 3862a* (DAO 01-01662140); site IG-19, *Hainault 4070* (DAO 01-01662153, O V547261, QK 59846). Inugsuin Mouth: site IM-1, *Hainault 3890* (DAO 01-01662151, O V547262, QK 60102). Kangiqtugaapik Head: site KP-1, *Dansereau 500624-0152* (MT00202112, MT00202125), *500624-0153a* (MT00202162), *500702-0251* (MT00202117, MT00202134), *500702-0354* (MT00202116, MT00202133), *500709-0560* (MT00202118, MT00202126), *500721-0662* (MT00202110, MT00202132), *500723-0851* (DAO 01-01662158, MT00202115, MT00202128), *500726-1376* (MT00202114, MT00202130), *500726-1479* (MT00202129), *500827-1166* (MT00202113, MT00202131), *Wynne-Edwards 8908-A* (CAN 10034741), *8952* (CAN 10034740).

##### *Carex nardina* Fr.—Nard sedge—Amphi-Beringian–North American–amphi-Atlantic

**Specimens examined: Agguttinni TP.** Atagulisaktalik: site 23.6, *Gillespie et al. 11517* (CAN 10114738, QFA); site AT-2, *Dansereau 500725-0466* (MT00106394, MT00106399). Clark Fiord: site 20.1, *Gillespie et al. 12336* (CAN 10114746, UBC). Kangiqtualuk Agguqti: site 3.1, *Gillespie et al. 11802* (ALTA, CAN 10114740, npsp); site 8.4, *Gillespie et al. 12003* (ALA, CAN 10114741); site 21.2, *Gillespie et al. 12417* (ALTA, CAN 10114747). Kangiqtualuk Uqquqti: site 28.1, *Gillespie et al. 11643* (CAN 10114739, MT). Kuugaaluk: site 19.4, *Gillespie et al. 12306* (ALA, CAN 10114745, CHARS, MT). Refuge Harbour: site 12.2, *Gillespie et al. 12040* (CAN 10114742, MO). Stewart Valley: site 15.1, *Gillespie et al. 12103* (CAN 10114743, US). Swiss Bay: site SB-2, *Starr 08-226* (CAN 10038166). Tingijattut: site 15.3, *Gillespie et al. 12147* (CAN 10114744). **Clyde Area.** Clyde River: site CR-1, *Malte s.n..* [118511] (CAN 10038143, GH barcode-02367618). Inugsuin Head: site IG-1, *Hainault 3992* (O V546951, QK 59800), *3719* (CAN 10038177, DAO 01-016610703, O V546953, QK 59603); site IG-4, *Parmelee & Seaborn 3987a* (DAO 01-01697014); site IG-30, *Hainault 4056* (CAN 10038206, O V546949, QK 59737). Inugsuin Mouth: site IM-1, *Hainault 3877* (CAN 10038212, O V546950, QK 59775). Kangiqtugaapik Head: site KP-1, *Dansereau 500703-0263* (MT00106392, MT00106400), *500703-0699* (MT00106395, MT00106401), *500803-0478* (MT00106393, MT00106396), *500811-0899* (MT00106390, MT00106403), *Wynne-Edwards 8908* (CAN 10038326), *8953* (CAN 10038172); site KP-15, *Dansereau 500713-0871* (MT00106397, MT00106391); site KP-18, *Wynne-Edwards 8801* (CAN 10038327); site KP-19, *Dansereau 500731-0277* (MT00106388, MT00106402); site KP-25, *Dansereau 500731-0570* (MT00106398, MT00106389). McBeth Valley: site MB-1, *Hainault 3756* (DAO 01-01661702, O V546952, QK 59593). **Home Bay.** Cape Hooper: site HO-1, *Elven 3474/99* (ALA V132172, CAN 10038201, O V546945). Ekalugad FOX-C: site FC-1, *Richardson & Webber 36* (COLO 01047554). Rocknoser Fiord: site RF-1, *Smith VP-70-61* (CAN 10038145).

##### *Carex rupestris* All.—Rock sedge—Circumpolar-alpine

**Specimens examined: Agguttinni TP.** Gibbs Fiord: site 20.9, *Gillespie et al. 12375* (CAN 10114751, NY). Kangiqtualuk Agguqti: site 3.5, *Gillespie et al. 11849* (ALA, CAN 10114753, NY, QFA); site 8.2, *Gillespie et al. 11927* (CAN 10114749, MT). Kangiqtualuk Uqquqti: site 27.1, *Gillespie et al. 11618* (CAN 10114752, npsp, US). Stewart Valley: site 15.1, *Gillespie et al. 12100* (CAN 10114750, npsp). Tasialuk S: site 26.5, *Gillespie et al. 11595* (ALTA, CAN 10114748, CHARS, MT, npsp). **Clyde Area.** Inugsuin Head: site IG-4, *Parmelee & Seaborn 3985c* (DAO 01-01667653); site IG-19, *Hainault 4071* (CAN 10038932, DAO 01-01667654, H 1229260, O V547033, QK 59847); site IG-25, *Hainault 4018* (QK 59724). Kangiqtugaapik Head: site KP-1, *Dansereau 500604-0561* (MT00107190, MT00107191), *500607-7020253* (?) (MT00107173), *500624-0153* (MT00107194, MT00107195), *500701-1552* (MT00107192, MT00107193), *500702-0253* (MT00107172, MT00107178), *500702-0356* (MT00107182, MT00107183), *500709-0558* (MT00107184, MT00107185), *500714-0158* (MT00107181), *500716-0858* (MT00107188, MT00107189), *500716-0862* (MT00107174, MT00107175), *500721-0661* (MT00107179), *500803-0861A* (MT00107177), *500827-1168* (MT00107186, MT00107187), *Wynne-Edwards 8904-A* (CAN 10038944), *8960* (CAN 10038931), *9016* (CAN 10039486); site KP-21, *Dansereau 500730-0953* (MT00107180), *500730-0953A* (MT00107176).

##### *Carex saxatilis* L.—Russet sedge—Circumboreal-polar

Also recorded from Scott’s Bay (Taylor 1863), but no collection located. This species has a scattered distribution on Baffin Island and the eastern Canadian Arctic, with collections from Ellesmere, Southampton, King William, and Victoria Islands (Aiken et al. 2007; Saarela et al. 2020a, b). While uncommon in the flora area, it has been frequently collected near the Mary River project site south of Pond Inlet by Page Burt (specimens at CAN) and is known from a collection in Pangnirtung (*Blouin 10*CAN 10039145), as well as scattered localities on west-central and southern Baffin Island.

**Specimens examined: Clyde Area.** Clyde River: site CR-1, *Oughton s.n.* (TRT00042273). Kangiqtugaapik Head: site KP-1, *Dansereau 500721-0557* (MT00287029, MT00287040), *500801-0457* (MT00287041, MT00287042), *500803-0771* (MT00287043, MT00287044), *Wynne-Edwards 9015* (CAN 10039150); site KP-16, *Wynne-Edwards 8982* (CAN 10039138).

##### Carex scirpoidea Michx. subsp. scirpoidea (Fig. 12A)—Single-spike sedge—Amphi-Beringian–North American (N)–amphi-Atlantic

Recorded from Scott’s Bay by Taylor (1863) (as “C. serpoides Mich.”, misspelled) and cited by Polunin (1940), but no collections seen.

**Specimens examined: Agguttinni TP.** Kangiqtualuk Agguqti: site 3.2, *Gillespie et al. 11832* (CAN 10114763, CHARS); site 8.2, *Gillespie et al. 11917* (CAN 10114761), *Gillespie et al. 11930* (ALA, CAN 10114762), Kangiqtualuk Uqquqti: site 27.1, *Gillespie et al. 11613* (CAN 10114764, MT, npsp), *11614* (ALTA, CAN 10114760, npsp). **Clyde Area.** Inugsuin Head: site IG-25, *Hainault 4014* (CAN 10039552, DAO 01-01667565, O V547068, QK 59722). Kangiqtugaapik Head: site KP-1, *Dansereau 500627-3260* (MT00106807, MT00106808), *500702-0252* (MT00106809, MT00106810, MT00106811), *500702-0357* (MT00106812, MT00106813), *500703-0455* (MT00106806), *500703-0861* (MT00106814, MT00106815), *500720-0659* (MT00106817), *500721-0659* (MT00106816), *500726-1482* (MT00106797, MT00106798), *500731-0278* (MT00106799, MT00106800), *500803-0861* (MT00106802, MT00106803), *500803-0864bis* (MT00106801), *500827-1167* (MT00106804, MT00106805), *Wynne-Edwards 8904* (CAN 10039551).

##### Carex simpliciuscula Wahlenb. subsp. subholarctica (T.V.Egorova) Saarela (≡Kobresia simpliciuscula subsp. subholarctica T.V.Egorova)—Simple bog sedge—Asian (NE)–amphi-Beringian–North American (N)–amphi-Atlantic (Svalbard)

**Specimens examined: Agguttinni TP.** Kangiqtualuk Agguqti: site 8.2, *Gillespie et al. 11915* (CAN 10114766); site 8.5, *Gillespie et al. 11966* (CAN 10114768, npsp). Kangiqtualuk Uqquqti: site 1.2, *Gillespie et al. 11781b* (CAN 10114767); site 28.1, *Gillespie et al. 11636* (CAN 10114765, CHARS, MT). **Clyde Area.** Kangiqtugaapik Head: site KP-1, *Dansereau 500830-3851* (MT00202161); site KP-5, *Wynne-Edwards 8985* (CAN 10031483).

##### *Carex subspathacea* Wormsk. ex Hornem.—Hoppner’s sedge—Circumpolar

This species is distributed primarily in the southern Canadian Arctic Islands. In the eastern Canadian Arctic, it is found north of Kangiqtugaapik Head only on Bylot Island (Aiken et al. 2007) and at one locality on Devon Island (*Svoboda s.n.*UBC V194288, det. P.W. Ball).

**Specimens examined: Clyde Area.** Kangiqtugaapik Head: site KP-1, *Dansereau 500714-0152* (MT00185811), *Wynne-Edwards 8917* (CAN 10039722), *8928* (CAN 10039710); site KP-9, *Dansereau 500708-085*1 (MT00185785), *500805-0584* [pro parte, plants A] (MT00185784); site KP-23, Wynne-Edwards 9081 (CAN 10039721). **Home Bay.** Kangirlugag Fiord: site KG-1, Webber 1188 (CAN 10039717, COLO 01062454, QK 71007).

##### Carex supina Willd. ex Wahlenb. subsp. spaniocarpa (Steud.) Hultén—Weak Arctic sedge—Asian (NE)–amphi-Beringian–North American (N)

These specimens, along with a collection from Pond Inlet (*Polunin 687*, CAN 10040967) and the Mary River (*Burt s.n.*, CAN 10039810) on northern Baffin Island, are at the northern edge of this species’ Canadian and North American range.

**Specimens examined: Agguttinni TP.** Clark Fiord: site 20.1, *Gillespie et al. 12326* (ALA, CAN 10114773, MO, QFA). Kangiqtualuk Agguqti: site 6.5, *Gillespie et al. 11893* (CAN 10114772); site 8.9, *Gillespie et al. 11986* (CAN 10114770, NY). Kangiqtualuk Uqquqti: site 30.3, *Gillespie et al. 11720* (ALTA, CAN 10114769, CHARS, MT, npsp). Tasialuk S: site 26.5, *Gillespie et al. 11594* (ALA, CAN 10114771). **Clyde Area.** Kangiqtugaapik Head: site KP-1, *Dansereau 500703-1452* (MT00286919, MT00286920), *Wynne-Edwards 8905* (CAN 10039772), *8954* (CAN 10039773), *9017* (CAN 10039771).

##### *Carex ursina* Dewey—Bear sedge—Circumpolar

**Specimens examined: Agguttinni TP.** Niaqurnaaluk-Qassialuit: site 18.1, *Gillespie et al. 12237* (ALA, ALTA, CAN 10114774, CHARS, MO, MT, npsp, NY). **Clyde Area.** Clyde River: site CR-1, *Polunin 606* (CAN 10039902). Inugsuin Mouth: site IM-1, *Hainault 3872* (CAN 10039911, H 1231371, O V547096, QK 59771). Kangiqtugaapik Head: site KP-1, *Dansereau 500819-2489bis* (MT00106491), *Wynne-Edwards 8918* (CAN 10039906). **Home Bay.** Ekalugad FOX-C: site FC-1, *Richardson & Webber 59* (CAN 10039905, COLO 01065440, QK 71076). Kangirlugag Fiord: site KG-1, *Webber 1304* (COLO 01065457). Rocknoser Fiord: site RF-1, *Smith VP-94-61* (CAN 10039901).

#### ﻿*Eriophorum* L.

##### Key to species and subspecies of *Eriophorum* [adapted from Ball and Wujek (2002), Cayouette (2004), and Saarela et al. (2020b)]

**Table:** 

1	Spikelets usually 2 or more per inflorescence, spreading or nodding, subumbellate or capitate, subtended by 1 or more blade-bearing involucral bracts, sometimes reduced to sheaths; distal leaves on culms with blades ≥ 1 cm long	**2**
–	Spikelets solitary, erect, without blade-bearing involucral bracts; distal leaves on culms bladeless or with blades 1 cm long	**3**
2	Peduncles smooth (sometimes scabrous only on angles), drooping, up to 5–10 cm; lowermost involucral bract cylindrical; flowering spikelets oblong-ovoid or oblong-elliptical; fruiting spikelets bell-shaped or narrowly bell-shaped; scales brownish grey, greyish, reddish, or ferrugineous, with white margins; anthers (2.5–)3–4(–5) mm long; achenes oblong-obovoid or oblong-elliptical, (2.5–)2.8–3(–3.5) mm long	***E. angustifolium***
–	Peduncles scabrous throughout, arcuate, up to 2 cm; lowermost involucral bract funnel-shaped; flowering spikelets ovoid to almost spherical; fruiting spikelets obovoid; scales blackish, without whitish margins; anthers (1.8–)2.5–2.8(–3) mm long; achenes widely obovoid, 2–2.5 mm long	***E. triste***
3	Culms usually solitary, plants rhizomatous; empty proximal scales usually 7 or fewer	**4**
–	Culms densely tufted, plants not rhizomatous; empty proximal scales usually 10 or more	**7**
4	Perianth bristles brownish to off white; spikelets globose in fruit; medial scales (0.8–)1.0–2.4 mm wide, acute, 0.25–0.6 mm wide just below apex, widest at middle to distal half of scale, fertile scales with hyaline margins ≥ 1 mm wide; anthers 1–3 mm long	**E. russeolum subsp. albidum**
–	Perianth bristles bright white to whitish; spikelets hemispherical to obovoid or globose in fruit; medial scales 0.3–1.5(–1.7) mm wide, acuminate, 0.05–0.3(–0.4) mm wide just below apex, widest at proximal half of scale, fertile scales with hyaline margins ≤ 1 mm wide; anthers 0.35–1.6 mm long	**5**
5	Anthers 0.9–1.6 mm long; hypogynous bristles (10–)22–32 mm long; stigmatic branches 1.0–2.2 mm long	**E. × medium subsp. album**
–	Anthers 0.35–1.0 mm long; hypogynous bristles 10–25 mm long; stigmatic branches 0.5–1. 3(–1.5) mm long (*E. scheuchzeri*)	**6**
6	Spikelets hemispherical; proximal fertile scales of spikelets dark, with dark margins or reduced hyaline margins sharply differentiated from the darker parts; medial scales narrowly acuminate (usually 0.1 mm wide at 0.2 mm below the apex), 0.3–0.7(–0.9) mm wide near the middle; mature achenes beige-brown to olive-brown, slightly lustrous	**E. scheuchzeri subsp. scheuchzeri**
–	Spikelets globose; proximal fertile scales of spikelets bicolored, with lower and medial parts dark but gradually passing to various tones of gray and conspicuous marginal and apical hyaline areas; medial scales acuminate (usually 0.2 mm wide at 0.2 mm below the apex), (0.5–)0.7–1.4(–1.6) mm wide near the middle; mature achenes orange-brown to dark reddish brown, mostly dull	**E. scheuchzeri subsp. arcticum**
7	Proximal scales spreading or reflexed in fruit, with white-hyaline margins to 1 mm wide; perianth bristles pure white in fruit; distal sheaths on culms inflated	**E. vaginatum subsp. spissum**
–	Proximal scales appressed to ascending, without conspicuous whitish margins; perianth bristles white or brownish; distal sheaths on culms inflated or not	**8**
8	Culms 20–70 cm long, smooth; sheaths evenly distributed along culms, distal not inflated, bladeless; proximal scales with broad ribless margins; anthers 0.5–2 mm	***E. brachyantherum***
–	Culms 5–20(–50) cm long, smooth or rough distally; sheaths mostly confined to proximal 1/2 of culm, often with short blade; proximal scales with ribs ± to margins; anthers 0.6–1.2 mm	***E. callitrix***

##### *Eriophorum angustifolium* Honck.—Narrow-leaved cottongrass—Circumboreal-polar

**Specimens examined: Agguttinni TP.** Atagulisaktalik: site 22.1, *Gillespie et al. 11465* (ALTA, CAN 10114776, MO, MT, npsp, NY, US). Clark Fiord: site 20.2, *Gillespie et al. 12354* (CAN 10114780, MO, npsp, QFA, US). Kangiqtualuk Agguqti: site 3.2, *Gillespie et al. 11817* (ALA, CAN 10114775, CHARS, NY, QFA, UBC, WIN). Stewart Valley: site 15.1, *Gillespie et al. 12106* (ALTA, CAN 10114778, MIN, US). Tingijattut: site 15.2, *Gillespie et al. 12144* (ALA, CAN 10114779, CHARS, MT). **Clyde Area**. Cape Hewett: site CH-1, *Platt 477* (NY 2836826), *478* (NY 2836821). Clyde River: site A22.6, *Gillespie et al. 12471* (CAN 10114777, MT); site CR-1, *Dutilly 1427* (US 02076617), *9377* (CM413443), *Martin 39* (DAO 01-01685977); site CR-2, *Forbes 8* (DAO 01-01685979), *Forbes 41c* (DAO 01-01685980), *104* (DAO 01-01685991). Hoffman Cove: site HF-1, *Bartlett 227* (US 02076831). Inugsuin Head: site IG-4, *Parmelee & Seaborn 3985a* (DAO 01-01685976). Inugsuin Mouth: site IM-1, *Hainault 3844* (CAN 10034168, DAO 01-01685989, O V547149, QK 59654). Kangiqtugaapik Head: site KP-1, *Dansereau 500624-0668* (MT00286392), *500625-0258* (DAO 01-01686001, MT00286389), *500627-2352* (DAO 01-01685986, MIN 1382698, MT00286396), *500630-2063* (MT00286397), *500702-0864* (MT00286388), *500709-086*7 (MT00286395), *500711-3752* (MT00286391), *500726-1378* (MT00286387), *500801-0452* (MT00286394), *500822-0770* (MT00286398), *500827-0761* (MT00286383), *500828-1317* (MT00286390), *500830-3953* (MT00286393), *Wynne-Edwards 8814* (CAN 10033655), *8864* (CAN 10034154). **Coutts Inlet.** Coutts Mouth: site CO-1, *Coombs 76* (DAO 01-01685999). **Home Bay.** Cape Hooper: site HO-1, *Elven 3472/99* (CAN 10034161). Ekalugad FOX-C: site FC-1, *Philpot et al. s.n.* (COLO 01077270). Ekalugad Head: site EK-3, *Philpot et al. s.n*. (COLO 01077288). Kangirlugag Fiord: site KG-1, *Webber 1212* (COLO 01077312, QK 71085), *1277* (CAN 10034157, COLO 01077304, QK 71084).

##### *Eriophorum brachyantherum* Trautv. & C.A.Mey.—Closed-sheathed cottongrass—Circumboreal-polar

These two cited collections are among the only ones known from Baffin Island; the Clyde area collection was first mapped in Porsild (1957) and Porsild and Cody (1980). There are two recent unreported collections from south and west Baffin Island (only *Saarela & Bull 5108*CAN verified here). Elsewhere in the Canadian Arctic, it is known from Southampton Island (Porsild 1957; Porsild and Cody 1980; Aiken et al. 2007) and Victoria Island (Gillespie et al. 2015; Saarela et al. 2020b).

**Specimens examined: Agguttinni TP.** Kangiqtualuk Agguqti: site 21.10, *Gillespie et al. 12436* (CAN 10114781, npsp). **Clyde Area.** Kangiqtugaapik Head: site KP-1, *Wynne-Edwards 9000* (CAN 10033988).

##### *Eriophorum callitrix* Cham.—Beautiful cottongrass—Asian (N)–amphi-Beringian–North American (N)

This species is uncommon on Baffin Island, and these specimens are the only records on the northeastern coast of the island (Aiken et al. 2007).

**Specimens examined: Agguttinni TP**. Kangiqtualuk Agguqti: site 8.7, *Gillespie et al. 11975* (ALA, CAN 10114783, npsp). Kangiqtualuk Uqquqti: site 1.2, *Gillespie et al. 11782* (CAN 10114784, MT); site 28.7, *Gillespie et al. 11679* (CAN 10114782, CHARS). **Clyde Area.** Kangiqtugaapik Head: site KP-1, *Dansereau, 500630-2192* (MT00286384), *500630-2292* (MT00286376), *500803-0779* (MT00286381, MT00286382), *Wynne-Edwards 8845* (CAN 10033920), *8859* (CAN 10033932); site KP-17, *Wynne-Edwards 8907* (CAN 10034180).

##### Eriophorum russeolum Fr. subsp. albidum (Nyl.) Väre (≡Eriophorum russeolum Fr. var. albidum Väre; =Eriophorum russeolum subsp. leiocarpum Novoselova)—Smooth-fruited russet cottongrass—Asian (N/C)–amphi-Beringian–North American (N)

We follow the taxonomy of Cayouette (2004, 2024) rather than Ball and Wujek (2002), where this taxon is treated within a broadly circumscribed *E. chamissonis* C.A.Mey. Porsild and Cody (1980) mapped two sites in the flora area, most likely corresponding to the two Clyde area sites listed below, although the dot for site KP-1 was somewhat misplaced; Aiken et al. (2007) incorrectly mapped the latter site between McBeth and Rocknoser Fiords, where no plant collections are known. This taxon is rare and sparsely distributed in the Canadian Arctic Islands, known outside the flora area from one site on Victoria Island (Saarela et al. 2020b), five sites on Banks Island (Aiken et al. 2007) (based on *Aiken 99-047*CAN, *Edlund s.n.*CAN 10027453, *Edlund s.n.*CAN 10027479, *Edlund s.n.*CAN 10027460, and *Edlund s.n.*CAN 10027449), one site on Melville Island (*Edlund 419*CAN), and at least four sites on southern Baffin Island (e.g., *Aiken 98-078*CAN, plant b, Koukdjuak River) (Aiken et al. 2007; but see Saarela et al. 2023). Cayouette (2004) cited one collection for Bylot Island off the northern coast of Baffin Island, and Porsild and Cody (1980) mapped two collections on northern Baffin Island, but these collections have not been located and verified. Collections from the flora area are among the northernmost records for the eastern Canadian Arctic.

**Specimens examined: Agguttinni TP.** Clark Fiord: site 20.2, *Gillespie et al. 12356* (CAN 10114785, CHARS, MT, npsp). **Clyde Area.** Inugsuin Mouth: site IM-1, *Hainault 3842* (CAN 10034007, DAO 01-01686225, O V547178, QK 59652). Kangiqtugaapik Head: site KP-1, *Dansereau 500711-3751* (MT00186235, MT00186236), *500711-3751A* (MT00186242), *500711-3751B* (MT00186244), *500711-3751C* (MT00186245).

##### Eriophorum scheuchzeri Hoppe subsp. arcticum M.S.Novos.—Scheuchzer’s Arctic cottongrass—Circumpolar

**Specimens examined: Agguttinni TP.** Atagulisaktalik: site 24.1, *Gillespie et al. 11543* (CAN 10114793). Generator Lake: site GL-12, *Raynolds & Bültmann MKR-2022-23* (CAN 10169272). Kangiqtualuk Agguqti: site 8.4, *Gillespie et al. 11961* (CAN 10114792). Kuugaaluk: site 16.8, *Gillespie et al. 12225* (ALTA, CAN 10114788, npsp); site 19.4, *Gillespie et al. 12318* (CAN 10114790, CHARS). Refuge Harbour: site 12.1, *Gillespie et al. 12042* (CAN 10114787, MT). Tingijattut: site 15.2, *Gillespie et al. 12145* (ALA, CAN 10114791). **Clyde Area.** Cape Hewett: site CH-1, *Platt 478* (NY 2836821). Clyde River: site A22.6, *Gillespie et al. 12472* (CAN 10114789); site CR-1, *Dutilly 1426a* (US 02077469), *9376* (CM409410), *Polunin 2603* (CAN 10034331); site CR-2, *Forbes 10* (DAO 01-01688358), *23* (DAO 01-01688360), *41b* (DAO 01-01688321), *101* (DAO 01-01719037); site CR-3, *Dare 43* (CAN 10092246). Inugsuin Head: site IG-15, *Parmelee & Seaborn 3879* (DAO 01-01688339); site KP-1, *Dansereau, 500822-0763* (MT00185895, MT00185913), *500822-0772* (MT00185873, MT00185906), *500828-0393* (MT00185912), *Wynne-Edwards 9033* (CAN 10034494); site KP-8, *Dansereau 500705-0455* (MT00185907, MT00185914), *500804-0782* (MT00185874, MT00185877); site KP-11, *Wynne-Edwards 8844* (CAN 10034263); site KP-27, *Dansereau 500716-0456* (MT00185878, MT00185911). **Coutts Inlet.** Coutts Fiord: site CF-1, *Coombs 52* (DAO 01-01719038). **Home Bay.** Cape Hooper: site HO-2, *Sadler s.n.* (ASC00038795); site HO-3, *Parmelee & Seaborn 3807a* (DAO 01-01688340). Ekalugad Head: site EK-3, *Philpot et al. s.n.* (COLO 01078351). Ekalugad FOX-C: site FC-1, *Richardson & Webber 38* (CAN 10034268, COLO 01078377, QK 71087). Kangirlugag Fiord: site KG-1, *Webber 1211* (CAN 10034381, COLO 01077908, QK 71086). Rocknoser Fiord: site RF-1, *Smith VP-42-61* (CAN 10034493).

##### Eriophorum scheuchzeri Hoppe subsp. scheuchzeri—Scheuchzer’s cottongrass—Circumpolar-alpine

Also recorded from Cape Adair and Scott’s Bay (as *Eriophorum capitatum* Host) (Taylor 1863), but no collections located. Although *E. capitatum* is currently considered a synonym of subsp. scheuchzeri, these records might represent either subspecies found in the flora area.

**Specimens examined: Clyde Area.** Inugsuin Head: site IG-1, *Hainault 4009* (CAN 10034380, DAO 01-01688344, O V547192, QK 59744). Inugsuin Mouth: site IM-1, *Hainault 3976* (CAN 10034382, DAO 01-01000677455, O V547193, QK 59797). Kangiqtugaapik Head: site KP-27, *Dansereau 500716-1456* (DAO 01-01688466).

##### *Eriophorum triste* (Th.Fr.) Hadač & Á.Löve (≡ Eriophorum angustifolium subsp. triste (Th.Fr.) Hultén)—Tall cottongrass—Amphi-Beringian–North American (N)–amphi-Atlantic

**Specimens examined: Agguttinni TP.** Generator Lake: site GL-12, *Raynolds & Bültmann MKR-2022-22* (CAN 10169281). Kangiqtualuk Agguqti: site 5.1, *Gillespie et al. 11860* (ALA, CAN 10114816, MO, QFA); site 8.4, *Gillespie et al. 11960* (CAN 10114798, MT, npsp); site 21.10, *Gillespie et al. 12435* (ALTA, CAN 10114795, NY, WIN). Kangiqtualuk Uqquqti: site 28.6, *Gillespie et al. 11673* (ALTA, CAN 10114796, CHARS, MT). Kuugaaluk: site 16.3, *Gillespie et al. 12205* (CAN 10114797). Refuge Harbour: site 11.1, *Gillespie et al. 12015* (ALA, CAN 10114794, npsp, NY, US). **Clyde Area.** Clyde River: site CR-1, *Polunin 645* (CAN 10034615); site CR-2, *Forbes 58* (DAO 01-01687776). Inugsuin Head: site IG-6, *Parmelee & Seaborn 3833* (DAO 01-01687797); site IG-30, *Hainault 3715* (CAN 10089106, O V547151, QK 59635), *4061* (CAN 10034609, DAO 01-01687747, H 1219851, O V547148, QK 59840). Inugsuin Mouth: site IM-1, *Hainault 3798* (QK 59799). Kangiqtugaapik Head: site KP-1, *Dansereau, 500604-0398* (MT00286399), *500628-5993* (MT00286377, MT00286378), *500716-0272* (MT00286385), *500720-0157* (MT00286379, MT00286380), *500723-1077* (MT00286386). McBeth Valley: site MB-1, *Hainault 3779* (CAN 10034599, DAO 01-01687748, O V547150, QK 59632). **Home Bay.** Cape Hooper: site HO-3, *Parmelee & Seaborn 3808* (DAO 01-01686000). Ekalugad FOX-C: site FC-1, *Philpot et al. s.n.* (COLO 01078872), *Richardson & Webber 15b* (COLO 01078930), *46* (CAN 10034600, COLO 01078922, QK 71083); site FC-5, *Oswald 361* (QK 135635). Rocknoser Fiord: site RF-1, *Smith VP-80-61* (CAN 10034610). Nudlung Fiord: site NF-1, *Ryder s.n.* (COLO 01078914).

##### Eriophorum vaginatum L. subsp. spissum (Fern.) Hultén—Dense cottongrass—North American (NE)

On the Canadian Arctic islands, this taxon is known only from Baffin Island, where it has a scattered distribution on both northern and southern parts (Aiken et al. 2007 [specimens confirmed here]; Saarela et al. 2020a; Saarela et al. 2023). These are the first records for the flora area and the first confirmed records for central Baffin Island. Porsild and Cody (1980) mapped a record from west-central Baffin Island, but no specimen has been located.

**Specimens examined: Home Bay.** Kangirlugag Fiord: site KG-1, *Webber 1257* (CAN 10034362, COLO 01079300). Ekalugad Head: site EK-3, *Philpot et al. s.n.* (COLO 01079276).

##### Eriophorum × medium subsp. album J.Cay.—North American (N)

This hybrid taxon, considered to be a cross between E. russeolum subsp. albidum and E. scheuchzeri subsp. scheuchzeri (Cayouette 2004), is only known from scattered sites on southern and eastern Baffin Island (Cayouette 2004; Aiken et al. 2007) and Nunavik in northern Quebec (Cayouette 2024). The single record from the flora area is at the northern edge of this hybrid’s Canadian (and global) range, about 420 km north of the closest locality at Nettilling Lake. Morphological characters are largely intermediate between the two parent taxa, and E. × medium subsp. album can be keyed out based on its intermediate anther length and by the length of the stigmatic branches (Cayouette 2004). The specimen cited here is a paratype (Cayouette 2004). Polunin (1940) tentatively cited this collection under *E. chamissonis* C.A.Mey., a species sometimes considered synonymous with *E. russeolum*, noting that it approached *E. scheuchzeri*. This record is unusual, and the locality is perhaps slightly suspect since neither parent is known from Clyde River, and both are uncommon in the flora area. The hybrid should be looked for in the vicinity of Clyde River and in locations where the two parents occur.

**Specimens examined: Clyde Area.** Clyde River: site CR-1, *Polunin 2599* (CAN 10034039).

#### ﻿Juncaceae

***Juncus* L.**

##### Key to species of *Juncus* [adapted from Porsild and Cody (1980), Brooks and Clemants (2002), and Saarela et al. (2020b)].

**Table:** 

1	Inflorescences lateral cymes, sympodial; bracts erect, terete, appearing to be a continuation of the culm; bracteoles 2, at base of perianth; basal leaves bladeless, cauline leaves absent; flowers borne singly, not in heads	***J. arcticus***
–	Inflorescences terminal panicles or racemes of several heads or a single terminal head, sympodial or monopodial; bracts ascending, terete or involute, distinct from culm; bracteoles absent at base of perianth; basal leaves (at least some) usually with blade, cauline leaves present or absent; flowers in multiflowered heads	**2**
2	Plants strongly rhizomatous, culms solitary; inflorescences of 1–3(–5) heads, each 2–10-flowered; tepals lanceolate, 4.5–6.6 mm long	***J. leucochlamys***
–	Plants cespitose, culms clustered; inflorescences single heads, each 1–2(–4)-flowered; tepals oblong or oblong-lanceolate, 2.5–5 mm long	**3**
3	Primary bract much longer than inflorescence; capsule apex retuse; filaments 1–1.5 mm long	***J. biglumis***
–	Primary bract nearly equal to or shorter than inflorescence; capsule apex obtuse, mucronate; filaments 2.5–4 mm long	**J. triglumis subsp. albescens**

##### *Juncus arcticus* Willd.—Arctic rush—Circumboreal-polar

Also recorded from Scott’s Bay (Taylor 1863), but no collection located.

**Specimens examined: Clyde Area.** Inugsuin Head: site IG-7, *Parmelee & Seaborn 3915* (DAO 01-01667600). Kangiqtugaapik Head: site KP-1, *Dansereau 500801-2489* (MT00253376, MT00253377), *Wynne-Edwards 8962* (CAN 10040290); site KP-2, *Martin 49* (DAO 01-01667601); site KP-9, *Wynne-Edwards 9084* (CAN 10040293).

##### *Juncus biglumis* L.—Two-glumed rush—Circumpolar-alpine

**Specimens examined: Agguttinni TP.** Atagulisaktalik: site 22.3, *Gillespie et al. 11495* (CAN 10114819, npsp). Generator Lake: site GL-15, *Raynolds & Bültmann MKR-2022-59* (CAN 10169261). Kangiqtualuk Agguqti: site 3.1, *Gillespie et al. 11815* (CAN 10114818, MT); site 8.7, *Gillespie et al. 11973* (CAN 10114824). Kangiqtualuk Uqquqti: site 30.1, *Gillespie et al. 11711* (CAN 10114817). Kuugaaluk: site 16.2, *Gillespie et al. 12203* (CAN 10114822). Refuge Harbour: site 11.2, *Gillespie et al. 12020* (CAN 10114820). Stewart Valley: site 15.1, *Gillespie et al. 12104* (CAN 10114821). Tingijattut: site 15.3, *Gillespie et al. 12149* (CAN 10114823). **Clyde Area.** Clyde River: site CR-1, *Dutilly 1452* (CAN 10040421, CAN 10040422), *1454* (CM409876), *Polunin 636* (CAN 10040425); site CR-2, *Forbes 34* (DAO 01-01667609), *133* (DAO 01-01667612). Inugsuin Head: site IG-1, *Hainault 4000* (QK 59539), *4076* (CAN 10040430, DAO 01-01667610, H 1311271, O V547340, QK 59817). Inugsuin Mouth: site IM-1, *Hainault 3868* (DAO 01-01667611, H 1311272, O V547339, QK 59768). Kangiqtugaapik Head: site KP-1, *Dansereau 500714-0157* (MT00279924), *500804-0783* (MT00279923), *Wynne-Edwards 8930* (CAN 10040448). **Home Bay.** Cape Hooper: site HO-1, *Elven 3462/99* (CAN 10040434, O V547351). Ekalugad FOX-C: site FC-1, *Richardson & Webber 49b* (CAN 10040453, COLO 02192177, QK 71091). Kangirlugag Fiord: site KG-1, *Webber 1189* (COLO 02192151), *1285* (CAN 10040447, QK 71092), *1290* (CAN 10040435, COLO 02192185, QK 71093).

##### *Juncus leucochlamys* V.J.Zinger ex V.I.Krecz. (≡*Juncus castaneus* subsp. *leucochlamys* (W.J.Zinger ex V.I.Krecz.) Hultén)—Many-flowered chestnut rush—Asian (N/C)–amphi-Beringian–North America (N)–amphi-Atlantic (Svalbard)

**Specimens examined: Agguttinni TP.** Kangiqtualuk Agguqti: site 8.5, *Gillespie et al. 11964* (CAN 10114786). Kangiqtualuk Uqquqti: site 28.5, *Gillespie et al. 11668* (CAN 10114799); site 30.1, *Gillespie et al. 11712* (CAN 10114800, CHARS, MT, npsp). **Clyde Area.** Clyde River: site CR-1, *Martin 51* (DAO 01-01667606). Inugsuin Head: site IG-1, *Hainault 3998* (O V547385, QK 59805); site IG-6, *Parmelee & Seaborn 3860* (DAO 01-01667602); site IG-9, *Parmelee & Seaborn 3947* (DAO 01-01667605); site IG-26, *Hainault 4036* (DAO 01-01667604, QK 59761); site IG-30, *Hainault 4059* (CAN 10040890, DAO 01-01667603, O V547386, QK 59740). Kangiqtugaapik Head: site KP-1, *Dansereau 500604-0396* (MT00279144), *500624-0462* (MT00279145), *500716-0363a* (MT00279142), *500716-0455* (MT00279143), *500725-0571* (MT00279147), *500819-0363* (MT00279170, MT00279589), *500828-0393* (MT00279146), *Wynne-Edwards 9083* (CAN 10040900). **Home Bay.** Ekalugad FOX-C: site FC-1, *Richardson & Webber 39* (CAN 10040891, COLO 02193803, QK 71090), *51* (CAN 10040905, COLO 02193779). Nudlung Fiord: site NF-1, *Ryder s.n.* (COLO 02193787).

##### Juncus triglumis L. subsp. albescens (Lange) Hultén (≡*Juncus albescens* (Lange) Fern.)—Northern white rush—Asian (N)–amphi-Beringian–North American (N)–amphi-Atlantic (Svalbard)

**Specimens examined: Agguttinni TP**. Kangiqtualuk Agguqti: site 8.2, *Gillespie et al. 11916* (CAN 10114826). Kangiqtualuk Uqquqti: site 28.5, *Gillespie et al. 11667* (CAN 10114825, npsp). **Clyde Area.** Inugsuin Head: site IG-15, *Parmelee & Seaborn 3878* (DAO 01-01667608). Kangiqtugaapik Head: site KP-1, *Wynne-Edwards 9013* (CAN 10041231). **Home Bay.** Rocknoser Fiord: site RF-1, *Smith VP-87-61* (CAN 10041246).

#### ﻿*Luzula* DC.

##### Key to species of *Luzula* [adapted from Swab (2000), Kirschner (2002), and Saarela et al. (2020b)]

**Table:** 

1	Basal leaves ± flat, usually up to 5 cm long, (2–)3–4 mm wide, tip obtuse, often slightly swollen; cauline leaves (1–)2, 1–2(–3) cm long; bracteole apices sparsely ciliate	***L. nivalis***
–	Basal leaves ± involute to subcanaliculate, sometimes ± flat, up to 6–9 cm long, 1.5–2.5 mm wide, tip acuminate; cauline leaves 1–2, usually 2–4 cm long; bracteole apices fimbriate-ciliate	***L. confusa***

##### *Luzula confusa* Lindeb. (Fig. 3C, 10A)—Northern woodrush—Circumpolar-alpine

**Specimens examined: Agguttinni TP.** Atagulisaktalik: site 22.1, *Gillespie et al. 11473* (ALTA, CAN 10114801, CHARS, MO, MT); site 24.1, *Gillespie et al. 11542* (ALA, CAN 10114802). Clark Fiord: site 20.1, *Gillespie et al. 12330* (CAN 10114811, npsp, US). Gee Lake: site 31.4, *Gillespie et al. 11751* (CAN 10114803, QFA). Generator Lake: site GL-11, *Raynolds & Bültmann MKR-2022-20* (CAN 10169269). Kangiqtualuk Agguqti: site 3.1, *Gillespie et al. 11803* (ALA, CAN 10114804, MT, NY); site 8.2, *Gillespie et al. 11933* (CAN 10114806, US, WIN). Kangiqtualuk Uqquqti: site 28.6, *Gillespie et al. 11674* (CAN 10114805, US). Kuugaaluk: site 16.1, *Gillespie et al. 12192* (CAN 10114810, npsp); site A22.2, *Gillespie et al. 12452* (CAN 10114812, QFA). Refuge Harbour: site 11.1, *Gillespie et al. 12006* (CAN 10114807, CHARS, MT). Remote Peninsula: site RL-1, *Philpot et al. s.n.* (COLO 02206407). Stewart Valley: site 15.1, *Gillespie et al. 12098* (ALTA, CAN 10114808, MO). Tingijattut: site 15.2, *Gillespie et al. 12138* (CAN 10114809); site TG-2, *Dare et al. 77* (CAN 10092423). **Clyde Area**. Cape Hewett: site CH-1, *Platt 485* (NY 3737125). Clyde River: site CR-1, *Dutilly 9380* (CM408633), *Malte s.n..* [118575] (CAN 10043482, GH barcode-02396909); site CR-2, *Forbes 9* (DAO 01-01000620396), *128* (DAO 01-01667616); site CR-3, *Dare 46* (CAN 10092426), *55* (CAN 10092427). Hoffman Cove: site HF-1, *Bartlett 222* (US 03845703). Inugsuin Head: site IG-20, *Hainault 3665* (DAO 01-01667614, H 1234766, O V547482, QK 59703); site IG-24, *Hainault 3644* (CAN 10041551, O V547485, QK 59575); site IG-30, *Hainault 4052* (QK 59730). Inugsuin Mouth: site IM-1, *Hainault 3871* (H 1234765, O V547483, QK 59770). Kangiqtugaapik Head: site KP-1, *Dansereau 500604-0255* (MT00286354, MT00286355), *500624-0769* (DAO 01-01667615, MT00286358, MT00286359, MT00286360), *500628-0764* (MT00286352, MT00286353), *500628-0865* (MT00286345, MT00286346), *500629-2185* (MT00286342, MT00286343), *500701-1056* (MT00286340, MT00286341), *500708-0961* (MT00286337, MT00286339), *500710-0154* (MT00286347, MT00286349), *500710-0291* (MT00286356, MT00286357), *500720-0158* (MT00286361), *500723-0353* (MT00286350, MT00286351), *500803-0184* (MT00286335, MT00286336), *500804-0655* (MT00286300), *500804-0655A* (MT00286299), *500825-0367* (MT00286294, MT00286297), *Wynne-Edwards 8863* (CAN 10041576), *8874* (CAN 10041571), *8888* (CAN 10041145), *8934* (CAN 10041144); site MB-1, *Hainault 3778* (DAO 01-01667613, O V547484, QK 59633). **Coutts Inlet.** Coutts Fiord: site CF-1, *Coombs 57* (DAO 01-01667635). Coutts Mouth: site CO-1, *Coombs 89* (DAO 01-01667634). **Home Bay.** Cape Hooper: site HO-1, *Elven 3464/99* (ALA V132433, CAN 10041490), *Hammar 3495/99* (CAN 10043477, O V547475). Ekalugad FOX-C: site FC-1, *Crompton et al. s.n.* (COLO 02206365), *Richardson & Webber 37* (COLO 02206423). Ekalugad Head: site EK-3, *Crompton et al. s.n.* (COLO 02206332); site EK-4, *Philpot et al. s.n.* (COLO 02206340). Kangirlugag Fiord: site KG-1, *Webber 1228* (CAN 10041563, COLO 02206431, QK 71089). Kangok Fiord: site KO-1, *Crompton et al. s.n.* (COLO 02206357). Nudlung Fiord: site NF-1, *Ryder s.n.* (COLO 02206373). Rocknoser Fiord: site RF-1, *Smith VP-55-61* (CAN 10041549), *Webber 1194* (COLO 02206415).

##### *Luzula nivalis* (Laest.) Spreng.—Arctic woodrush—Circumpolar-alpine

Also recorded from Coutts Inlet (https://www.inaturalist.org/observations/237624112).

**Specimens examined: Agguttinni TP.** Atagulisaktalik: site 22.1, *Gillespie et al. 11462* (CAN 10114844, US). Generator Lake: site GL-10, *Raynolds & Bültmann MKR-2022-21* (CAN 10169270). Gibbs Fiord: site 20.10, *Gillespie et al. 12387* (CAN 10114840). Kangiqtualuk Agguqti: site 3.1, *Gillespie et al. 11799* (ALTA, CAN 10114836); site 8.2, *Gillespie et al. 11932* (ALA, CAN 10114839). Kangiqtualuk Uqquqti: site 28.6, *Gillespie et al. 11675* (CAN 10114838, MT). Niaqurnaaluk-Qassialuit: site 18.2, *Gillespie et al. 12247* (CAN 10114843, CHARS, MO, npsp). Refuge Harbour: site 11.3, *Gillespie et al. 12030* (CAN 10114841). Remote Peninsula: site RL-1, *Philpot et al. s.n*. (COLO 02207710). Tingijattut: site 15.2, *Gillespie et al. 12139* (CAN 10114842). **Clyde Area.** Cape Hewett: site CH-1, *Platt 486* (NY 3737159). Clyde River: site CR-1, *Dutilly 1449* (CAN 10041917), *1450* (CAN 10043446), *1480a* (CM408866, DAO 01-01667620), *9379A* (CM362719), *Martin 36* (DAO 01-01667621), *Polunin 611* (CAN 10043502); site CR-2, *Forbes 131* (DAO 01-01667623). Inugsuin Head: site IG-1, *Hainault 3716* (DAO 837333, O V547551, QK 59634); site IG-7, *Parmelee & Seaborn 3923* (DAO 01-01667622), *3927* (DAO 01-01667624). Inugsuin Mouth: site IM-1, H*ainault 3854* (CAN 10043512, H 1234557, O V547550, QK 59790). Kangiqtugaapik Head: site KP-1, *Dansereau 500720-0662* (MT00286374, MT00286375), *500804-0655B* (MT00286372), *500804-0655Bis* (MT00286373), *Wynne-Edwards 8889* (CAN 10043507). Clyde Area. McBeth Valley: site MB-1, *Hainault 3769* (DAO 837334, H 1234556, O V547549, QK 59666). **Home Bay.** Cape Hooper: site HO-1, *Elven 3470/99* (ALA V132432, O V547538), *Elven 3501/99* (O V547537); site HO-3, *Parmelee & Seaborn 3801* (DAO 780578), *3810* (DAO 781594). Ekalugad FOX-C: site FC-1, *Richardson & Webber 44* (CAN 10043513, COLO 02207793, QK 71088). Ekalugad Head: site EK-1, *Crompton et al. s.n.* (COLO 02207785); site EK-3, *Crompton et al. s.n.* (COLO 02207728), *Crompton et al. s.n.* (COLO 02207744). Kangirlugag Fiord: site KG-1, *Webber 1215* (CAN 10043510, COLO 02207736). Rocknoser Fiord: site RF-1, *Smith VP-106-61* (CAN 10043506).

#### ﻿Poaceae

***Alopecurus* L.**

##### *Alopecurus borealis* Trin. (=*Alopecurus alpinus* Sm., illeg. hom.) —Alpine foxtail—Circumpolar-alpine

This species is often treated within a broader *A. magellanicus* Lam. (Soreng et al. 2003; Aiken et al. 2007; Crins 2007). Here we follow Elven et al. (2011) in treating it as a distinct Arctic species separate from the South American *A. magellanicus*, supported also by preliminary molecular data (Gillespie et al., unpubl.).

**Specimens examined: Agguttinni TP.** Generator Lake: site 31.5, *Gillespie et al. 11763* (ALA, CAN 10114847, CHARS); site GL-21, *Raynolds & Bültmann MKR-2022-44* (CAN 10169266). Kuugaaluk: site 16.3, *Gillespie et al. 12204* (CAN 10114848, MT); site 19.4, *Gillespie et al. 12304* (ALTA, CAN 10114849, npsp, QFA). Remote Peninsula: site RL-1, *Philpot et al. s.n.* (COLO 01420967). **Clyde Area.** Cape Hewett: site CH-1, *Platt 485A* (NY 1638207). Clyde River: site CR-1, *Dutilly 1428* (QFA0014217), *9389* (CAN 10008343, QFA0131963), *Malte s.n..* [118816] (CAN 10008465), *Sanson 68* (TRT00038257); site CR-2, *Forbes 11* (DAO 01-01626476), *60* (DAO 01-01611569), *125* (DAO 01-01612112). Hewett Cache: site HC-1, *Hainault 3798* (QK 59549). Inugsuin Mouth: site IM-1, *Hainault 3867* (DAO 01-01626481, O V545271, QFA 58194, QK 59769), *3853* (CAN 10008336, H 1159261, O V545270, QK 59789). Kangiqtugaapik Head: site KP-1, *Dansereau 500625-0457* (MT00286475), *500709-0351* (MT00286476), *500726-1684* (MT00286477), *Wynne-Edwards 8950* (CAN 10008335). McBeth Valley: site MB-1, *Hainault 3788* (DAO 01-01626482, QK 59551). **Coutts Inlet.** Coutts Mouth: site CO-1, *Coombs 86* (DAO 01-01626525). **Home Bay.** Cape Hooper: site HO-1, *Elven 3455/99* (O V545273), *3476/99* (CAN 10008339). Ekalugad FOX-C: site FC-1, *Philpot et al. s.n.* (COLO 01420959), *Philpot et al. s.n.* (COLO 01420983), *Richardson & Webber 18* (CAN 10008472, COLO 01420934, QK 71122); site FC-2, *Oswald 365* (QK 135285), *393* (QK 135325). Ekalugad Head: site EK-3, *Philpot et al. s.n.* (COLO 01420942), *Philpot et al. s.n.* (COLO 01420975). Kangirlugag Fiord: site KG-1, *Webber 1210* (CAN 10008467, COLO 01420991, QK 71121). Rocknoser Fiord: site RF-1, *Smith VP-104-61* (CAN 10008471).

#### ﻿*Anthoxanthum* L.

##### Anthoxanthum monticola (Bigelow) Veldkamp subsp. alpinum (Sw. ex Willd.) Soreng (≡*Hierochloe alpina* (Sw. ex Willd.) Roem. & Schult.) (Fig. 2D)—Alpine sweetgrass—Circumpolar-alpine

**Specimens examined: Agguttinni TP**. Atagulisaktalik: site 22.1, *Gillespie et al. 11472* (ALTA, CAN 10114874, MO, MT, npsp, US). Clark Fiord: site 20.1, *Gillespie et al. 12329* (CAN 10114852, QFA). Kangiqtualuk Agguqti: site 3.1, *Gillespie et al. 11801* (CAN 10114872, CHARS, npsp, US); site 8.2, *Gillespie et al. 11926* (CAN 10114871, MT). Kangiqtualuk Uqquqti: site 27.1, *Gillespie et al. 11617* (CAN 10114873, NY, QFA). Kuugaaluk: site A22.3, *Gillespie et al. 12462* (ALA, CAN 10114851, US). Marble Lake: site 31.1, *Gillespie et al. 11734* (ALA, ALTA, CAN 10114853, NY, UBC, WIN). Refuge Harbour: site 11.1, *Gillespie et al. 12010* (ALTA, CAN 10114870). Stewart Valley: site 15.1, *Gillespie et al. 12097* (CAN 10114850, MO). Tingijattut: site 15.2, *Gillespie et al. 12137* (CAN 10114854, MT, US); site TG-2, *Dare et al. 75* (CAN 10092534), *76* (CAN 10092531). **Clyde Area.** Cape Hewett: site. CH-1, *Platt 487* (NY 1640994). Clyde River: site CR-1, *Dutilly 9388* (CM412222, US 04014077), *Martin 68* (DAO 01-01625385, QFA0052976, UTC00157324), *Polunin 608* (CAN 10012948); site CR-2, *Forbes 19* (DAO 01-01611617), *117* (DAO 01-01625450). Inugsuin Head: site IG-3, *Parmelee & Seaborn 3999a* (DAO 01-01625453); site IG-24, *Hainault 3642* (CAN 10012893, DAO 01-01625389, H 1050564, O V545816, QK 59573). Inugsuin Mouth: site IM-1, *Hainault 3832* (DAO 01-01625390, H 1050562, O V545814, QFA 58263, QK 59684). Kangiqtugaapik Head: site KP-1, *Dansereau 500629-2187* (MT00286481), *500709-0453* (MT00286483), *500710-3753* (MT00286479), *500721-0354* (MT00286480), *500726-0864* (DAO 01-01625412, MT00286482), *Wynne-Edwards 8843* (CAN 10012952); site KP-17, *Dansereau 500623-1856* (MT00286478); site KP-18, *Wynne-Edwards 8826* (CAN 10012951). McBeth Valley: site MB-1, *Hainault 3746* (H 1050563, O V545815, QFA 58262, QK 59582). **Coutts Inlet.** Coutts Fiord: site CF-1, *Coombs 55* (DAO 01-01611621, DAO 01-01625439, MO 2577372, US 04014044). Coutts Mouth: site CO-1, *Coombs 84* (DAO 01-01625441). **Home Bay.** Cape Hooper: site HO-1, *Elven 3467/99* (ALA V132109, CAN 10012922, O V545792); site HO-3, *Parmelee & Seaborn 3792a* (DAO 01-01625413). Ekalugad FOX-C: site FC-1, *Crompton et al. s.n*. (COLO 01481068), *Philpot et al. s.n.* (COLO 01481118), *Richardson & Webber 21* (CAN 10012892, COLO 01481209, QK 71144); site FC-2, *Oswald 368* (QK 135287). Ekalugad Head: site EK-1, *Church 2* (COLO 01481167), *5* (COLO 01481159), *6* (COLO 01481142), *10* (COLO 01481175), *s.n.* (UBC V162142). Kangirlugag Fiord: site KG-1, *Webber 1221* (CAN 10012891, QK 71143), *1295* (COLO 01481324, QFA 20948), *1305* (CAN 10012916, COLO 01481217). Kangok Fiord: site KO-1, *Crompton et al. s.n.* (COLO 01481076), *Crompton et al. s.n.* (COLO 01481100). Nudlung Fiord: site NF-1, *Ryder s.n.* (COLO 01481092). Rocknoser Fiord: site RF-1, *Smith VP-18-61* (CAN 10012895).

#### ﻿*Arctagrostis* Griseb.

##### Arctagrostis latifolia (R.Br.) Griseb. subsp. latifolia—Wide-leaved polargrass—Circumpolar-alpine

**Specimens examined: Agguttinni TP.** Atagulisaktalik: site 22.1, *Gillespie et al. 11466* (CAN 10114833, MT); site AT-1, *Dansereau 500725-0573* (MT00286551). Clark Fiord: site 20.2, *Gillespie et al. 12353* (CAN 10114828). Kangiqtualuk Agguqti: site 3.1, *Gillespie et al. 11813* (ALA, ALTA, CAN 10114813, CHARS, MT); site 8.7, *Gillespie et al. 11982* (CAN 10114815, US). Kangiqtualuk Uqquqti: site 28.7, *Gillespie et al. 11680* (CAN 10114814, npsp). Kuugaaluk: site 16.2, *Gillespie et al. 12202* (CAN 10114830, MT); site 19.4, *Gillespie et al. 12319* (CAN 10114827, NY, UBC, WIN). Refuge Harbour: site 11.1, *Gillespie et al. 12014* (CAN 10114831, QFA). Stewart Valley: site 15.1, *Gillespie et al. 12107* (ALA, CAN 10114832). Tingijattut: site 15.2, *Gillespie et al. 12146* (CAN 10114829); site TG-2, *Dare et al. 74* (CAN 10092549). **Clyde Area.** Cape Hewett: site CH-1, *Platt 483* (NY 1474243), *484* (NY 1474232). Clyde River: site CR-1, *Dutilly 1431* (QFA0009586, QFA0131999), *9391* (CAN 10009043, CM413099, QFA0021004), *Oughton s.n.* (TRT00038901), *Polunin 633* (CAN 10008960); site CR-2, *Forbes 28* (DAO 01-01658177), *132* (DAO 01-01658178); site CR-3, *Dare 47* (CAN 10092548). Hoffman Cove: site HF-1, *Bartlett 218* (US 04052715). Inugsuin Head: site IG-19, *Hainault 4074* (ACAD 70035, H 1164456, O V545330, QK 59850). Inugsuin Mouth: site IM-1, *Hainault 3843* (H 1164458, O V545331, QFA 58204, QK 59653), *Hainault 3864* (DAO 01-01658897, H 1167459, O V545333, QFA 58203, QK 59795). Kangiqtugaapik Head: site KP-1, *Dansereau, 500624-0465* (MT00286556), *500709-0869* (MT00286533), *500713-0559* (MT00286553), *500721-0558* (MT00286531), *500726-1272* (MT00286536), *500801-0353* (DAO 01-01660497, MT00286532, MT00286537, MT00286552), *500819-0161* (MT00286549), *500819-0362* (MT00286548), *500822-0464* (MT00286550), *500827-0854* (MT00286529), *500827-0960* (MT00286554), *Dansereau s.n.* (MT00286534). Kangiqtugaapik Head: site KP-5, *Wynne-Edwards 9030* (CAN 10009102). McBeth Valley: site MB-1, *Hainault 3765* (CAN 10009101, DAO 01-01658896, H 1167457, O V545332, QK 59663). **Coutts Inlet.** Coutts Mouth: site CO-1, *Coombs 85* (DAO 01-01660503). **Home Bay.** Cape Hooper: site HO-1, *Elven 3435/99* (ALA V132104, CAN 10009219, O V545326). Ekalugad FOX-C: site FC-1, *Richardson & Webber 19* (CAN 10009104, COLO 01424936, QK 71124), *41* (COLO 01424928, QFA 20951, QK 71125). Ekalugad Head: site EK-1, *Church 2* (COLO 01425008), *Church s.n.* (UBC V162143), *Crompton et al. s.n.* (COLO 01424894); site EK-3, *Philpot et al. s.n.* (COLO 01424886). Kangirlugag Fiord: site KG-1, *Webber 1216* (CAN 10009105, COLO 01424902, QK 71123), *1278* (CAN 10009015, COLO 01425081). Nudlung Fiord: site NF-1, *Ryder s.n.* (COLO 01425016). Rocknoser Fiord: site RF-1, *Smith VP-77-61* (CAN 10009048).

#### ﻿*Calamagrostis* Adans.

##### *Calamagrostis purpurascens* R.Br.—Purple reedgrass—Asian (NE)–amphi-Beringian–North American–amphi-Atlantic (Svalbard)

In North America, this conspicuous species is relatively common in the western Cordillera and western mainland Arctic but is rare to uncommon with a very scattered distribution in the Canadian Arctic Islands. On Baffin Island it is known from only six localities. Aiken et al. (2007), Porsild (1957), and Porsild and Cody (1980) mapped it from three localities, Kangiqtugaapik Head and two localities on northern Baffin Island. Saarela et al. (2023) published the first record for southern Baffin Island from Katannilik Territorial Park, and Gillespie et al. (2024) first recorded it from Agguttinni TP. One additional record for Baffin Island (*LaFarge-England 00149*ALTA) from Maktak Fiord, just south of the flora area, has not yet been verified.

**Specimens examined: Agguttinni TP.** Kangiqtualuk Agguqti: site 8.2, *Gillespie et al. 11934* (CAN 10114755); site 8.6, *Gillespie et al. 11970* (CAN 10114754, npsp); site 21.1, *Gillespie et al. 12415* (CAN 10114863, MT). **Clyde Area.** Kangiqtugaapik Head: site KP-1, *Dansereau 500730-0954* (MT00286474), *500731-0569* (MT00286473), *500805-0587* [pro parte, plants B] (MT00286452), *500819-1064* (MT00286472), *Wynne-Edwards 9074* (CAN 10010565); site KP-8, *Wynne-Edwards 9085* (CAN 10010562), *9088* (CAN 10010561); site KP-22, *Wynne-Edwards 9075* (CAN 10010591).

#### ﻿*Deschampsia* P.Beauv.

##### Key to species of *Deschampsia* [adapted from Barkworth (2007)]

**Table:** 

1	Panicles usually dense, oblong-ovate to narrowly cylindrical, 0.5–2 cm wide, branches erect to ascending, straight, usually stiff; leaf blades flat, folded, or convolute, forming a basal tuft or not, longer or shorter than the flowering culms	***D. brevifolia***
–	Panicles usually open and pyramidal, 1.5–9 cm wide, branches spreading, divergent, or reflexed, flexuous; leaf blades usually strongly rolled, hairlike, usually forming a dense basal tuft, much shorter than the flowering culms	***D. sukatschewii***

##### *Deschampsia brevifolia* R.Br. (=Deschampsia cespitosa (L.) P.Beauv. subsp. septentrionalis Chiapella)—Short-leaved hairgrass—Asian (N)–amphi-Beringian–North American (N)

Although widespread but scattered across most of the Canadian Arctic Islands, this species is rather rare on Baffin Island. Polunin (1940) cited three collections from northern Baffin. Porsild (1957) and Porsild and Cody (1980) mapped a single locality on northernmost Baffin Island (likely based on *Polunin 2543*CAN from Arctic Bay). Aiken et al. (2007) mapped two additional localities on Baffin Island, one in the north (*Scotter & Zoltai 67273*DAO) and one on the Koudjuak River in the southwest (*Aiken 98-065*CAN). Additional localities based on verified collections include Bowman Bay on southwest Baffin (*Boles 00-196*CAN), Cape Searle on southeast Baffin (*Wynne-Edwards 9140*CAN), Bylot Island off the north coast (*Duclos s.n.*CAN 10013279), and Prince Charles Island off the central west coast (*Baldwin 1898*CAN). We report here and in Gillespie et al. (2024) (for those in Agguttinni TP) the first collections from east-central Baffin Island; Barkworth (2007) had previously mapped a dot on the Home Bay area, presumably based on one of these collections.

**Specimens examined: Agguttinni TP.** Kuugaaluk: site 19.4, *Gillespie et al. 12320* (ALA, CAN 10114837, CORD, npsp, US). Remote Peninsula: site RL-1, *Philpot et al. s.n.* (COLO 01451798). **Clyde Area.** Clyde River: site 10.1, *Gillespie et al. 11994* (ALTA, CAN 10114834, CHARS, CORD, MO, MT). **Home Bay.** Ekalugad FOX-C: site FC-1, *Richardson & Webber 12* (CAN 10031047, QFA 20935, QK 71134), *15* (COLO 01451806), *52* (CAN 10031050, COLO 01451780); site FC-2, *Oswald 394* (QK 135309). Ekalugad Head: site EK-3, *Philpot et al. s.n.* (COLO 01451814). Kangirlugag Fiord: site KG-1, *Webber 1224* (CAN 10031051, COLO 01451772).

##### *Deschampsia sukatschewii* (Popl.) Roshev (=*D. pumila* (Griseb.) Ostenf., illeg. hom.; =Deschampsia cespitosa subsp. borealis (Trautv.) Á. Löve & D. Löve)—Dwarf hairgrass—Circumpolar

This species has a very scattered distribution in the Canadian Arctic islands (Porsild and Cody 1980; Aiken et al. 2007; Saarela et al. 2020b). On Baffin Island, Porsild (1957) and Porsild and Cody (1980) mapped six sites on the south and central parts of the island; Aiken et al. (2007) confirmed only one of these sites and mapped two additional sites in the south. Saarela et al. (2020a, 2023) cited new collections from the vicinity of Kimmirut and from Dorset Island, just off Cape Dorset in southern Baffin Island. Polunin (1940) first recorded the species in the flora area at Clyde River based on his own collections made in 1934 and 1936 (the 1936 collection is cited here; we have not seen any Polunin collections from 1934). The three sites mapped in Porsild and Cody (1980) presumably correspond to the Clyde area collections cited here (the northernmost dot likely corresponds to the Clyde River collection but is misplaced).

**Specimens examined: Agguttinni TP**. Generator Lake: site GL-16, *Bültmann & Raynolds MKR-2022-33* (CAN 10169290). **Clyde Area.** Clyde River: site CR-1, *Polunin 2594* (CAN 10031429). Inugsuin Head: site IG-22, *Hainault 4047* (CAN 10031425, H 1168303, O V545514, QK 59727). Kangiqtugaapik Head: site KP-5, *Wynne-Edwards 9023* (CAN 10031436).

#### ﻿*Dupontia* R.Br.

##### *Dupontia fisheri* R.Br.—Fisher’s tundra grass—Circumpolar

**Specimens examined: Agguttinni TP.** Atagulisaktalik: site 24.4, *Gillespie et al. 11552* (CAN 10114845, MT, npsp). Generator Lake: site GL-22, *Raynolds & Bültmann MKR-2022-49* (CAN 10169282). Kangiqtualuk Agguqti: site 5.5, *Gillespie et al. 11872* (CAN 10114858, MO); site 8.1, *Gillespie et al. 11906* (CAN 10114860); site 8.4, *Gillespie et al. 11959* (CAN 10114857, QFA, US). Kangiqtualuk Uqquqti: site 1.2, *Gillespie et al. 11780* (ALTA, CAN 10114846, CHARS). Kuugaaluk: site 19.4, *Gillespie et al. 12317* (CAN 10114859, NY). Tingijattut: site 15.2, *Gillespie et al. 12142* (ALA, CAN 10114856, UBC). **Clyde Area.** Clyde River: site CR-1, *Malte s.n..* [118860] (CAN 10011249, CAN 10011489, QFA0013744); site CR-2, *Forbes 143* (DAO 01-01659871). Inugsuin Head: site IG-1, *Hainault 3999* (O V545544, QFA 58191, QK 59806), *4004* (CAN 10011449, H 1175743, O V545619, QK 59742), *4010* (DAO 01-01659878, H 1175741, O V545543, QFA 581189, QK 59745); site IG-19, *Hainault 4075* (CAN 10011458, H 1175762, O V545618, QK 59851); site IG-30, *Hainault 4060* (CAN 10011471, H 1175744, O V545620, QK 59839). Inugsuin Mouth: site IM-1, *Hainault 3977* (QFA 58188, QK 59798). Kangiqtugaapik Head: site KP-1, *Dansereau 500805-0587* [pro parte, plants A] (MT00286451), *500819-2387* (MT00286450), *Wynne-Edwards 9032* (CAN 10011271); site KP-9, *Wynne-Edwards 9005* (CAN 10011229), *9006* (CAN 10011270). **Home Bay.** Ekalugad FOX-C: site FC-1, *Richardson & Webber 34* (CAN 10011457, COLO 01458421, *53* (COLO 01458405, QFA 20946). Kangirlugag Fiord: site KG-1, *Webber 1183* (CAN 10011452, COLO 01458413, QFA 20936, QK 71136), *1291* (CAN 10011454, COLO 01458439, QK 71135). Kangirlugag Fiord: site KG-1, *Webber 1314* (CAN 10011453, COLO 01458447). Rocknoser Fiord: site RF-1, *Smith VP-86-61* (CAN 10011272).

#### ﻿*Festuca* L.

##### Key to species of *Festuca* [adapted from Aiken et al. (1995) and Darbyshire and Pavlick (2007)]

**Table:** 

1	Culms densely pubescent or pilose below the inflorescences; anthers 0.3–0.7(–1.1) mm long; ovary apex with a few sparse hairs	***F. baffinensis***
–	Culms usually glabrous below the inflorescences, occasionally slightly scabrous or sparsely puberulent; anthers (0.3–)0.4–1.3 mm long; ovary apex glabrous	**2**
2	Inflorescences 1.5–4(–5.5) cm long; flag leaf blades (3–)10–30 mm long, sheaths not inflated; upper glumes 2.9–4.6 mm long; lemmas 3–5.2 mm long; plants 5–35 cm tall, erect	**F. brachyphylla subsp. brachyphylla**
–	Inflorescences 1–2(–2.5) cm long; flag leaf blades 2–6(–8) mm long, sheaths somewhat inflated; upper glumes 2.2–3.2 mm long; lemmas 2.9–4 mm long; plants 2.5–10 (–17) cm tall, often semi-prostrate	***F. hyperborea***

##### *Festuca baffinensis* Polunin (Fig. 9G)—Baffin Island fescue—Asian (NE)–amphi-Beringian–North American–amphi-Atlantic

Our collection is the first verified record for the flora area. Although previously mapped from one locality in the Clyde area (Porsild 1957; Porsild 1964; Porsild and Cody 1980), no collections from this area were located. Polunin (1940) did not record the species on central Baffin Island. The dot in the Home Bay area on the map in Aiken et al. (2007) appears to be a mapping error since this is not a known collection site and no collection or locality information is available in their mapping database.

**Specimens examined**: Agguttinni TP. Kangiqtualuk Uqquqti: site 30.4, *Gillespie et al. 11718* (CAN 10114835).

##### Festuca brachyphylla Schult. & Schult.f. subsp. brachyphylla—Short-leaved fescue—Circumpolar-alpine

**Specimens examined: Agguttinni TP.** Atagulisaktalik: site 22.2, *Gillespie et al. 11484* (CAN 10114968); site 24.1, *Gillespie et al. 11541* (CAN 10114969, MT). Clark Fiord: site 20.1, *Gillespie et al. 1233*1 (ALTA, CAN 10114978, CHARS, MO, MT, npsp). Generator Lake: site GL-17, *Raynolds & Bültmann MKR-2022-3*1 (CAN 10169289). Gibbs Fiord: site 20.11, *Gillespie et al. 12391* (ALA, CAN 10114966, QFA, US, WIN). Kangiqtualuk Agguqti: site 3.1, *Gillespie et al. 11804* (CAN 10114972); site 8.2, *Gillespie et al. 11929* (CAN 10114973). Kuugaaluk: site 19.4, *Gillespie et al. 12301* (CAN 10114977, QFA, US). Stewart Valley: site 15.1, *Gillespie et al. 12102* (CAN 10114975). Tasialuk N: site 26.1, *Gillespie et al. 11577* (ALTA, CAN 10114970, CHARS, MO, QFA, UBC, US). Tingijattut: site 15.5, *Gillespie et al. 12166* (CAN 10114976); site TG-2, *Dare et al. 72* (CAN 10092581). **Clyde Area.** Clyde River: site 10.4, *Gillespie et al. 11998* (ALA, CAN 10114974, NY); site CR-1, *Dutilly 1444b* (CM412233, QFA0015731), *9390* (CAN 10012244, QFA0132426, QFA0021019), *9392A* (CM412241), *Malte s.n..* [118368] (CAN 10012535); site CR-2, *Forbes 42* (DAO 01-01758081), *140* (DAO 01-01758590). Hewett Cache: site HC-1, *Hainault 3805* (H 1176884, O V545700, QFA 58190, QK 30104). Inugsuin Head: site IG-2, *Hainault 3657* (CAN 10012241, DAO 01-01758002, H 1176888, O V545697, QK 59696); site IG-3, *Parmelee & Seaborn 4003* (DAO 01-01758597); site IG-11, *Parmelee & Seaborn 3984* (DAO 01-01758608); site IG-30, *Hainault 4053* (ACAD 70039, CAN 10012240, H 1176889, O V545699, QK 59731). Inugsuin Mouth: site IM-1, *Hainault 3820* (H 1176885, O V545698, QFA 58261, QK 59673). Kangiqtugaapik Head: site KP-1, *Dansereau 500710-0157bis* (MT00286587), *500710-2272* (MT00286586), *500723-0357* (MT00286588), *500726-1168* (MT00286589), *500803-0482* (MT00286585), *Wynne-Edwards 9087* (CAN 10012242); site KP-2, *Martin 53* (DAO 01-01758589, QFA0589063, UTC00157325); site KP-10, *Wynne-Edwards 8986* (CAN 10012549); site KP-20, *Wynne-Edwards 8992* (CAN 10012519); site KP-25, *Wynne-Edwards 9063* (CAN 10012433). McBeth Valley: site MB-1, *Hainault 3789* (DAO 01-01758000, H 1176886, O V545701, QFA 58196, QK 59623). **Coutts Inlet.** Coutts Fiord: site CF-1, *Coombs 54* (DAO 01-01758004). Coutts Mouth: site CO-1, *Coombs 83* (DAO 01-01758005). **Home Bay.** Cape Hooper: site HO-1, *Elven 3478/99* (O V545685), *3488/99* (ALA V131951, CAN 10012272, O V545684). Ekalugad FOX-C: site FC-1, *Richardson & Webber 23* (CAN 10012625, COLO 01473628, QK 71138). Ekalugad Head: site EK-1, *Church 7* (COLO 01471861); site EK-3, *Philpot et al. s.n.* (COLO 01473636). Kangirlugag Fiord: site KG-1, *Webber 1213* (CAN 10012494, COLO 01471838), *1289* (CAN 10012626, COLO 01473610), *1298b* (CAN 10012234, COLO 01471820, QK 71137), *Webber 1312* (COLO 01473149, QFA 20947, QFA 132426, QK 71139). Rocknoser Fiord: site RF-1, *Smith VP-102-61* (CAN 10012550).

##### *Festuca hyperborea* Holmen ex Fred.—High Arctic fescue—Circumpolar

**Specimens examined: Agguttinni TP.** Clark Fiord: site 20.7, *Gillespie et al. 12373* (ALA, CAN 10114965). Generator Lake: site 31.6, *Gillespie et al. 11765* (CAN 10114960, CHARS, MT). Kangiqtualuk Agguqti: site 6.3, *Gillespie et al. 11886* (ALTA, CAN 10114961, npsp). Kangiqtualuk Uqquqti: site 28.3, *Gillespie et al. 11655* (CAN 10114959); site 29.2, *Gillespie et al. 11688* (CAN 10114971). Kuugaaluk: site 16.1, *Gillespie et al. 12191* (CAN 10114964); site A22.3, *Gillespie et al. 12463* (CAN 10114967, US). Ravenscraig Harbour: site RH-1, *Dare 4* (CAN 10092572), *19* (CAN 10092573). Refuge Harbour: site 12.4, *Gillespie et al. 12045* (CAN 10114962). Tingijattut: site 15.9, *Gillespie et al. 12184* (CAN 10114963). **Clyde Area.** Inugsuin Mouth: site IM-1, *Hainault 3901* (CAN 301871, DAO 01-01758001, H 1176887, O V545749, QK 59807). **Home Bay.** Ekalugad Head: site EK-1, *Philpot et al. s.n.* (COLO 01473644); site EK-4, *Philpot et al. s.n.* (COLO 01473156).

#### *Koeleria* Pers.

##### *Koeleria spicata* (L.) Barberá, Quintanar, Soreng & P.M.Peterson (≡*Trisetum spicatum* (L.) K.Richt.)—Spike trisetum—Circumpolar-alpine

**Specimens examined: Agguttinni TP.** Atagulisaktalik: site AT-1, *Röthlisberger s.n.* (CAN 10021540). Kuugaaluk: site 19.4, *Gillespie et al. 12305* (ALA, CAN 10114862, CHARS, MT, npsp, QFA). Tasialuk S: site 21.12, *Gillespie et al. 12449* (ALTA, CAN 10114861, US). **Clyde Area.** Hoffman Cove: site HF-1, *Bartlett 240* (US 04008745), *242* (US 04008589). Inugsuin Mouth: site IM-1, *Hainault 3839* (DAO 01-01578422, QK 59543), *3848* (DAO 01-01578423, H 1168747, O V546440, QK 59658). Kangiqtugaapik Head: site KP-1, *Wynne-Edwards 9095* (CAN 10021539); site KP-20, *Wynne-Edwards 8997* (CAN 10021541); site KP-27, *Wynne-Edwards 9067* (CAN 10021526). **Home Bay.** Ekalugad FOX-C: site FC-1, *Richardson & Webber 54* (COLO 01542935). Kangirlugag Fiord: site KG-1, *Webber 1231* (CAN 10021563, COLO 01542885, QK 71132), *1299a* (COLO 01542943). Cape Hooper: site HO-1, *Elven 3504/99* (O V546406).

#### ﻿*Leymus* Hochst.

##### Leymus mollis (Trin.) Pilg. subsp. villosissimus (Scribn.) Á.Löve & D.Löve (≡ Elymus arenarius L. subsp. villosissimus (Scribn.) Á.Löve) (Fig. 12G)—Arctic lymegrass—Asian (NE)–amphi-Beringian–North American (N)

This beach and sand dune grass was found only at the head of two fiords in the flora area. Kangiqtugaapik Head was previously mapped in Porsild (1957) and Porsild and Cody (1980) as Elymus arenarius subsp. mollis (Trin.) Hultén; the Kangiqtualuk Agguqti collection is newly reported here and in Gillespie et al. (2024). Elsewhere in the Canadian Arctic it is common in suitable habitats in the low Arctic but sparse and widely scattered in the mid to high Arctic. On Baffin Island it is also recorded from the southern part of the island and several sites on the northern part (Porsild 1957; Porsild and Cody 1980; Aiken et al. 2007; Saarela et al. 2020a; Saarela et al. 2023). Porsild and Cody (1980) mapped it at one site in the high Arctic, on Ellesmere Island, but no specimen was found to verify this record.

**Specimens examined: Agguttinni TP**. Kangiqtualuk Agguqti: site 8.1, *Gillespie et al. 11911* (CAN 10114855, MT, npsp). **Clyde Area.** Kangiqtugaapik Head: site KP-1, *Dansereau 500710-0467* (DAO 01-01556157, MT00286488), *Wynne-Edwards 9038* (CAN 10016771); site KP-5, *Dansereau 500828-0562* (MT00286487).

#### ﻿*Phippsia* (Trin.) R.Br.

##### *Phippsia algida* (Sol.) R.Br.—Icegrass—Circumpolar

**Specimens examined: Agguttinni TP.** Arviqtujuq Kangiqtua NW: site 18.3, *Gillespie et al. 12250* (CAN 10114868). Gee Lake: site 31.4, *Gillespie et al. 11749* (CAN 10114866, CHARS, MT). Generator Lake: site 31.8, *Gillespie et al. 11772* (ALTA, CAN 10114869); site 31.9, *Gillespie et al. 11774* (ALA, CAN 10114867, npsp, UBC, US); site GL-16, *Raynolds & Bültmann MKR-2022-32* (CAN 10169262). Kangiqtualuk Agguqti: site 6.3, *Gillespie et al. 11883* (CAN 10114864). Kuugaaluk: site 16.8, *Gillespie et al. 12228* (CAN 10114865, MO, MT, QFA); site A22.3, *Gillespie et al. 12458* (CAN 10114876, UBC). Ravenscraig Harbour: site 18.6, *Gillespie et al. 12278* (CAN 10114875). **Clyde Area.** Clyde River: site CR-1, *Dutilly 1482* (CAN 514012), *1483* (QFA0012660, QFA0015767, US 04051014, US 04051059), *9393* (CAN 514021, CM520106, US 04051066), *9394* (QFA0021037, QFA0132653, QFA0624918, US 04051064). Clyde River: site CR-1, *Malte s.n.*. (CAN 10089472); site CR-2, *Forbes 25* (DAO 01-01551615). Inugsuin Head: site IG-12, *Parmelee & Seaborn 3977* (BABY 06184, DAO 01-01551620, DAO 01-01659369); site IG-21, *Hainault 4048* (DAO 01-01551632, H 1164074, O V545883, QK 59728). Inugsuin Mouth: site IM-1, *Hainault 3905* (DAO 01-01551631, H 1164076, O V545882, QK 59809), *3875* (CAN 10089273, H 1164075, O V545881, QK 59774). **Home Bay.** Cape Hooper: site HO-1, *Elven 3451/99* (ALA V129711, CAN 583380, O V545894). Ekalugad FOX-C: site FC-1, *Richardson & Webber 58* (QK 71141), *66* (CAN 10089270, QK 71140); site FC-2, *Oswald 392* (QK 135314). Kangirlugag Fiord: site KG-1, *Webber 1307* (CAN 312002, QK 71142). Rocknoser Fiord: site RF-1, *Smith VP-97-61* (CAN 10089268).

#### ﻿*Pleuropogon* R.Br.

##### *Pleuropogon sabinei* R.Br.—Sabine’s semaphoregrass—Circumpolar

Also recorded from Cape Adair and Scott’s Bay (Taylor 1863), but no collections located.

**Specimens examined: Agguttinni TP.** Atagulisaktalik: site 22.3, *Gillespie et al. 11500* (CAN 10114927, MT); site 24.4, *Gillespie et al. 11551* (CAN 10114925, US). Generator Lake: site 31.7, *Gillespie et al. 11776* (CAN 10114926); site GL-22, *Raynolds & Bültmann MKR-2022-62* (CAN 10169287). Kuugaaluk: site 16.8, *Gillespie et al. 12227* (ALA, CAN 10114933, CHARS, npsp). **Clyde Area.** Clyde River: site CR-1, *Dutilly 1438* (CM411970), *9386* (CM358023, US 03988433), *Polunin 648* (CAN 10016999); site CR-2, *Forbes 33* (DAO 01-01551115), *135* (DAO 01-01551109). Inugsuin Head: site IG-14, *Parmelee & Seaborn 3899* (DAO 01-01551130); site IG-20, *Hainault 4007* (CAN 10017001, DAO 01-01551108, H 1172239, O V545924, QK 59743). Inugsuin Mouth: site IM-1, *Hainault 3862* (CAN 10016958, DAO 01-01551107, QK 59793). Kangiqtugaapik Head: site KP-11, *Wynne-Edwards 8938* (CAN 10017007), *8978* (CAN 10016965). **Home Bay.** Rocknoser Fiord: site RF-1, Smith VP-88-61 (CAN 10017002).

#### ﻿*Poa* L.

##### Key to species and subspecies of *Poa* [adapted from Gillespie (2023) and Soreng (2007)]

**Table:** 

1	Plants rhizomatous (rhizomes short in P. arctica subsp. caespitans), loosely to densely cespitose, or culms solitary; sheaths closed 1/4–1/2 of length; callus with large (sometimes small) web of crinkled hairs	**2**
–	Plants not rhizomatous, usually densely cespitose, sometimes loosely cespitose (*P. hartzii*); sheaths closed 1/10–1/5 of length; callus glabrous, with small to large web of crinkled to straight hairs, or with crown of hairs	**4**
2	Lemmas not hairy between veins, lateral veins glabrous or sparsely short-villous proximally, rarely long-villous; paleas glabrous between veins; panicles lanceoloid or narrowly ellipsoid, sometimes narrowly pyramidal, contracted to somewhat open (and then opening tardily), usually moderately densely flowered, secondary axes with (2) 3–13 spikelets	**P. alpigena subsp. alpigena**
–	Lemmas short villous or puberulent proximally between veins, lateral veins long-villous; paleas pubescent between veins; panicles pyramidal, open (opening soon after emergence from sheath), sparsely flowered, secondary axes with 1-5 (9) spikelets (*P. arctica*)	**3**
3	Plants moderately to densely cespitose, rhizomes poorly developed, usually very short, arching upwards, culms densely clustered; leaf blades usually flat, broad (2–4.8 mm wide); panicle secondary axes with 1–3(–5) spikelets; anthers sterile or fertile	**P. arctica subsp. caespitans**
–	Plants not or loosely cespitose, rhizomes well-developed and usually long, culms solitary or clustered; leaf blades folded to flat, often narrow, sometimes broad (1–2.5(–4) mm wide); panicle secondary axes with 1–5(–9) spikelets; anthers fertile	**P. arctica subsp. arctica**
4	Panicles usually pyramidal, sometimes ovoid; leaves mostly basal (densely crowded at base of culm), blades 2–4.5 mm wide, apex broadly prow-shaped, cauline blades (0) 1–2; spikelets ovate to broadly ovate, base rounded, subcordate, or broadly obtuse; callus glabrous	***P. alpina***
–	Panicles lanceoloid, ovoid, or cylindrical; leaves mostly basal to mostly cauline, blades (0.6–)1–3 mm wide, apex narrowly prow-shaped, cauline blades 1–5; spikelets narrowly lanceolate, lanceolate, ovate, or broadly ovate, base narrowly acute to obtuse; callus usually with web or crown of hairs or rarely glabrous	**5**
5	Anthers 0.2–0.8 mm long; panicles 1–3 cm long, not or little exerted above leaves	**P. abbreviata subsp. abbreviata**
–	Anthers 1–2 mm long; panicles 3–7 cm long, usually much exerted above leaves	**6**
6	Spikelets weakly compressed; glumes weakly keeled, the back mostly rounded; callus usually with crown of soft villous to somewhat crinkled hairs; lemma surface between veins sparsely puberulent; anthers sterile; leaves mostly basal, often forming dense tufts, cauline blades 1 (2); ligules 2.5–4.5 mm long	**P. hartzii subsp. hartzii**
–	Spikelets strongly compressed; glumes distinctly keeled; callus with web of crinkled hairs or rarely glabrous; lemma surface between veins glabrous or puberulent; anthers fertile; leaves mostly cauline, basal blades usually early becoming dry and shrivelled, cauline blades (1) 2–3 (4); ligules 1–2.5(–3) mm long	**P. glauca subsp. glauca**

##### Poa abbreviata R.Br. subsp. abbreviata (Fig. 13E)—Dwarf bluegrass—Nearly circumpolar

This species, found at two sites, is newly reported for the flora area here and in Gillespie et al. (2024). In Canada this taxon is mostly restricted to the Canadian Arctic Islands, where it is common in the high Arctic. The two collections cited here are the easternmost records for Baffin Island, and along with one collection from near the Barnes Ice Cap just outside the flora area (*Webber 472*CAN), also the southernmost records for Baffin Island. Aiken et al. (2007) incorrectly mapped the species on Cambridge Fiord (south of Coutts Inlet) based on a specimen from Ellesmere Island (CAN 296221).

**Specimens examined: Agguttinni TP.** Kangiqtualuk Agguqti: site 8.1, *Gillespie et al. 11899* (ALA, ALTA, CAN 10114892, CHARS, MO, MT, npsp, NY, QFA, UBC, US). **Clyde Area**. Clyde River: site CR-3, *Dare 42* (CAN 10092703).

##### Poa alpigena Lindm. var. alpigena (≡Poa pratensis L. subsp. alpigena (Lindm.) Hiitonen)—Alpine meadow bluegrass—Circumboreal-polar

This species previously was mapped only from Kangiqtugaapik Head (Porsild 1957; Porsild and Cody 1980; Aiken et al. 2007); we record here one additional locality for the flora area and for central Baffin Island. The taxon has most recently been treated as a subspecies of *P. pratensis* [e.g., Soreng (2007), Soreng et al. (2009), and Gillespie (2023)]; however, molecular data support treating it as a separate species with two varieties, the second being P. alpigena var. colpodea (Th. Fr.) Schol. (≡Poa pratensis subsp. colpodea (Th. Fr.) Tzvelev) (Soreng et al. 2020; Gillespie and Soreng, unpubl. data).

**Specimens examined: Clyde Area.** Clyde River: site CR-1, *Dutilly 1435* (QFA 15787), *1481* (QFA 15788). Kangiqtugaapik Head: site KP-8, *Wynne-Edwards 9007* (CAN 10019057); site KP-28, *Wynne-Edwards 9071* (CAN 10019058).

##### *Poa arctica* R.Br.—Arctic bluegrass—pâturin arctique—Circumpolar-alpine

Two subspecies are recognized in the flora area. The specimens cited here are mostly immature or poorly collected and could not be identified to subspecies.

**Specimens examined: Clyde Area.** Clyde River: site CR-1, *Dutilly 9395* (CM411953). McBeth Valley: site MB-1, *Hainault 3747* (CAN 10017605, O V546072, QK 59583), *3768* (CAN 10017604, DAO 01-01000684867, H 1173596, O V546069, QK 59665). **Home Bay.** Ekalugad FOX-C: site FC-1, *Richardson & Webber 26a* (CAN 10017599, COLO 01516194, QK 71118). Ekalugad Head: site EK-4, *Philpot et al. s.n.* (COLO 01516178).

##### Poa arctica R.Br. subsp. arctica—Arctic bluegrass—Circumpolar-alpine

Also recorded from Feachem Bay, Buchan Gulf (https://www.inaturalist.org/observations/37255027).

**Specimens examined: Agguttinni TP.** Atagulisaktalik: site 22.3, *Gillespie et al. 11496* (CAN 10114877, CHARS, MT); site 23.3, *Gillespie et al. 11506* (ALTA, CAN 10114898, MO). Gee Lake: site 31.4, *Gillespie et al. 11752* (CAN 10114902, QFA). Generator Lake: site 31.6, *Gillespie et al. 11767* (ALA, CAN 10114900, US); site GL-14, *Raynolds & Bültmann MKR-2022-27* (CAN 10169256). Gibbs esker: site 20.12, *Gillespie et al. 12402* (ALA, CAN 10114909). Gibbs Fiord: site 20.10, *Gillespie et al. 12385* (CAN 10114908, US). Kangiqtualuk Agguqti: site 3.2, *Gillespie et al. 11825* (CAN 10114901); site 8.2, *Gillespie et al. 11925* (CAN 10114880). Kangiqtualuk Uqquqti: site 29.4, *Gillespie et al. 11704* (CAN 10114899, NY, UBC, US). Kuugaaluk: site 16.1, *Gillespie et al. 12190* (CAN 10114906, QFA, UBC, WIN); site 19.5, *Gillespie et al. 12322* (CAN 10114907, MT, US); site A22.3, *Gillespie et al. 12460* (CAN 10114910, npsp, US). Refuge Harbour: site 11.3, *Gillespie et al. 12034* (CAN 10114903). Stewart Valley: site 15.1, *Gillespie et al. 12121* (CAN 10114905). **Clyde Area.** Clyde River: site 10.1, *Gillespie et al. 11992* (CAN 10114879, CHARS, MT); site CR-1, *Dutilly 9392* (CAN 10017206, QFA0021069, QFA0132790, QFA0292120, US 04054813), *Malte s.n..* [118393] (CAN 10017184), *Malte s.n..* [118407] (CAN 10017215), *Malte s.n..* [118408] (CAN 10017183); site CR-2, *Forbes 142* (DAO 01-01468025). Hewett Cache: site HC-1, *Hainault 3802* (H 1173603, O V546077, QFA 58269, QK 60103). Hoffman Cove: site HF-1, *Bartlett 216* (US 04055018). Inugsuin Head: site IG-1, *Hainault 3706* (QFA 58267, QK 59649), *4041* (CAN 10017207, DAO 01-01468236, H 1173599, O V546026, QK 59764); site IG-3, *Parmelee & Seaborn 4001* (DAO 01-01468018); site IG-19, *Hainault 4069* (ACAD 70041, CAN 10017477, H 1173602, O V546074, QK 58945); site IG-24, *Hainault 3645* (DAO 01-01641022, O V546073, QK 59576); site IG-30, *Hainault 4055* (ACAD 70044, CAN 10017478, H 1173600, O V546075, QK 59732). Inugsuin Mouth: site IM-1, *Hainault 3816* (CAN 10017480, DAO 01-01468237, H 1173595, O V546070, QK 59565), *3926* (CAN 10017475, H 1173594, O V546071, QK 59814). Kangiqtugaapik Head: site KP-1, *Dansereau 500702-0763* (MT00286584, MT00286754), *500710-0157* (MT00286751), *500710-0258A* (MT00286755), *500725-0574* (MT00286753), *500726-1273* (MT00286752), *500730-1192* (MT00286750), *500822-0465* (MT00286747), *500827-0762* (MT00286583), *Wynne-Edwards 8924* (CAN 10017632); site KP-2, *Martin 54* (DAO 01-01468231); site KP-5, *Wynne-Edwards 9018* (CAN 10017186); site KP-8, *Wynne-Edwards 9008* (CAN 10017203); site KP-20, *Wynne-Edwards 8999* (CAN 10017192); site KP-25, *Wynne-Edwards 9080* (CAN 10017481). McBeth Valley: site MB-1, *Hainault 3791* (DAO 01-01468234, H 1173598, O V546076, QFA 58268, QK 59622). **Coutts Inlet.** Coutts Fiord: site CF-1, *Coombs 56* (DAO 01-01467995). Coutts Mouth: site CO-1, *Coombs 87* (DAO 01-01467969), *88* (DAO 01-01467988). **Home Bay.** Cape Hooper: site HO-1, *Elven 3452/99* (ALA V132241, O V546063), *3454/99* (CAN 10017586). Ekalugad FOX-C: site FC-2, *Oswald 355* (QK 135318). Ekalugad Head: site EK-1, *Crompton et al. s.n.* (COLO 01516160). Kangirlugag Fiord: site KG-1, *Webber 1207* (CAN 10017479, COLO 01516202, QK 71120), *1257* (CAN 10017648, COLO 01516186), *1294* (CAN 10017642, COLO 01516327, QFA 20922, QK 71119). Rocknoser Fiord: site RF-1, *Webber 1193* (COLO 01516343).

##### Poa arctica subsp. caespitans Simmons ex Nannf.—High Arctic bluegrass—North American (NE)–amphi-Atlantic–European (N)

**Specimens examined: Agguttinni TP.** Kangiqtualuk Agguqti: site 6.3, *Gillespie et al. 11889* (CAN 10114878). Tingijattut: site 15.9, *Gillespie et al. 12181* (CAN 10114904, npsp). **Clyde Area.** Inugsuin Head: site IG-3, *Parmelee & Seaborn 3998a* (DAO 01-01468026). Kangiqtugaapik Head: site KP-1, *Wynne-Edwards 8977* (CAN 10017627). **Home Bay.** Cape Hooper: site HO-1, *Hammar 3494/99* (O V546064).

##### Poa glauca Vahl subsp. glauca—Glaucous bluegrass—Circumpolar-alpine

**Specimens examined**: Agguttinni TP. Clark Fiord: site 20.1, *Gillespie et al. 12323* (ALTA, CAN 10114920, CHARS, MO, US). Clark Fiord: site 20.1, *Gillespie et al. 12335* (ALA, CAN 10114921, QFA). Generator Lake: site GL-18, *Raynolds & Bültmann MKR-2022-34* (CAN 10169258). Gibbs Fiord: site 20.9, *Gillespie et al. 12376* (CAN 10114922, NY), *12383* (CAN 10114923, MT). Kangiqtualuk Agguqti: site 6.2, *Gillespie et al. 11878* (ALTA, CAN 10114916, CHARS, MO, QFA, US, WIN); site 8.2, *Gillespie et al. 11939* (CAN 10114917, NY); site 8.6, *Gillespie et al. 11969* (CAN 10114918, UBC, WIN). Kangiqtualuk Uqquqti: site 29.2, *Gillespie et al. 11687* (CAN 10114915, MT, npsp, US). Tasialuk S: site 26.5, *Gillespie et al. 11592* (ALA, CAN 10114914, US). **Clyde Area.** Clyde River: site 10.2, *Gillespie et al. 11995* (ALA, CAN 10114919, MT); site CR-1, *Martin 67* (DAO 01-01490990, UTC00157327). Inugsuin Head: site IG-1, *Hainault 3717* (CAN 10015948, DAO 01-01490816, O V546173, QK 59601), *4002* (DAO 01-01490928, H 1174383, O V546175, QK 59741); site IG-3, *Parmelee & Seaborn 4002a* (DAO 01-01490978); site IG-30, *Hainault 4054* (CAN 10015962, DAO 01-01490927, O V546176). Kangiqtugaapik Head: site KP-1, *Dansereau 500604-0562* (MT00286108), *500710-0258* (DAO 01-01490958, MT00286103), *500710-0258bis* (MT00286106), *500710-0366* (MT00286105, MT00286756), *500710-2273* (MT00286757), *500716-0859* (MT00286107), *500721-0455* (MT00286109), *500723-0870* (MT00286101), *500726-1170* (MT00286104, MT00286758), *500803-0473* (MT00286100), *500819-2184* (MT00286748, MT00286749); site KP-2, *Martin 52* (DAO 01-01490976, UTC00157326); site KP-5, *Wynne-Edwards 9020* (CAN 10015865); site KP-8, *Wynne-Edwards 9009* (CAN 10015949); site KP-10, *Wynne-Edwards 8987* (CAN 10016484); site KP-20, *Wynne-Edwards 8991* (CAN 10015924), *8998* (CAN 10015867). McBeth Valley: site MB-1, *Hainault 3752* (DAO 01-01490929, H 1174385, O V546174, QK 59588). **Home Bay.** Ekalugad Head: site EK-2, *Ryder s.n.* (COLO 01520865).

##### Poa hartzii Gand. subsp. hartzii (Fig. 12H)—Hartz’s bluegrass—Amphi-Beringian–North American (N)–amphi-Atlantic (Svalbard)

In Canada, this taxon is uncommon to common in the High and western Arctic islands but was previously recorded on Baffin Island only from Kangiqtugaapik Head (Porsild 1957; Gillespie et al. 1997; Aiken et al. 2007). We report here and in Gillespie et al. (2024) a second locality for the flora area and Baffin Island. Further south in eastern Canada, it has been collected from a single locality in northern Quebec (Blondeau and Cayouette 2002; Payette 2023b). Two collections are known from Bylot Island, off the north coast of Baffin Island (one verified here: *Drury 54421*CAN).

**Specimens examined: Agguttinni TP.** Kangiqtualuk Uqquqti: site 30.6, *Gillespie et al. 11729* (CAN 10114911, CAN 10114912, CAN 10114913, CHARS, MT, npsp). **Clyde Area.** Kangiqtugaapik Head: site KP-5, *Wynne-Edwards 9019* (CAN 10016708), *9055* (CAN 10016704); site KP-23, *Wynne-Edwards 9079* (CAN 10016706); site KP-28, *Wynne-Edwards 9072* (CAN 10033440).

#### ﻿*Puccinellia* Parl.

##### Key to species of *Puccinellia* [adapted from Davis and Consaul (2007)]

**Table:** 

1	Plants stoloniferous, forming low, often extensive mats; anthers, when present, 2–2.5 mm long; most plants lacking inflorescences, when present, usually not producing mature pollen or caryopses	**P. phryganodes subsp. neoarctica**
–	Plants cespitose, not mat-forming; anthers 0.6–1.5 mm long; plants reproducing sexually, forming mature pollen and caryopses	**2**
2	Palea veins glabrous, smooth; lemmas 2–2.5 mm long, glabrous or with few hairs	**P. tenella subsp. langeana**
–	Palea veins with curly, intertwined hairs proximally, scabrous distally; lemmas 2.8–5.2 mm long, hairy	**3**
3	Pedicels smooth; lower glumes ≥ 2/3 lemma length; lemma apical margins smooth	***P. vahliana***
–	Pedicels scabrous; lower glumes < 2/3 lemma length; lemma apical margins scabrous, sometimes minutely so	**4**
4	Lemmas 3.5–5.2 mm long; panicles (4–)5–13 cm long; upper glumes 2.2–4.2 mm long	***P. angustata***
–	Lemmas 2.8–3.8 mm long; panicles 1–4 cm long; upper glumes 1.5–2.8 mm long	***P. bruggemannii***

##### *Puccinellia angustata* (R.Br.) E.L.Rand & Redfield—Narrow alkaligrass—Circumpolar

**Specimens examined: Agguttinni TP.** Kangiqtualuk Agguqti: site 8.3, *Gillespie et al. 11954* (CAN 10114934, npsp). Kuugaaluk: site 19.4, *Gillespie et al. 12312* (ALA, ALTA, CAN 10114936, CHARS, MO, MT, npsp, QFA, UBC, US). Remote Peninsula: site RL-1, *Philpot et al. s.n.* (COLO 01530278). **Clyde Area**. Inugsuin Head: site IG-1, *Hainault 3996* (CAN 10018860, DAO 01-01660334, H 1176585, O V546349); site IG-18, *Hainault 4005* (CAN 10018801, DAO 01-01657470, H 1176256, O V546288, QK 59557). Kangiqtugaapik Head: site KP-5, *Wynne-Edwards 9054* (CAN 10018800); site KP-23, *Wynne-Edwards 9076* (CAN 10018809), *9077* (CAN 10018798); site KP-28, *Wynne-Edwards 9078* (CAN 10018802). **Home Bay.** Ekalugad FOX-C: site FC-1, *Richardson & Webber 11* (CAN 10018792, COLO 01530179, QK 71127), *Richardson & Webber 69* (CAN 10018859, COLO 01530914, QK 71117). Kangirlugag Fiord: site KG-1, *Webber 1185* (CAN 10018793, COLO 01530062, QK 71128), *1225* (COLO 01530070, QK 71129).

##### *Puccinellia bruggemannii* T.J.Sørensen (Fig. 13A)—Bruggemann’s alkaligrass—North American (N)

Our collection is the first record from Baffin Island (Gillespie et al. 2024). This species is a Canadian Arctic endemic, found primarily in the high Arctic and western Arctic islands.

**Specimens examined: Agguttinni TP.** Kuugaaluk: site 16.1, *Gillespie et al. 12193* (ALA, CAN 10114935, CHARS, MT, npsp, US).

##### Puccinellia phryganodes (Trin.) Scribn. & Merr. subsp. neoarctica (Á.Löve & D.Löve) Elven—Creeping alkaligrass—North American (N)

**Specimens examined: Agguttinni TP.** Kangiqtualuk Agguqti: site 6.3, *Gillespie et al. 11882* (CAN 10114944). Kuugaaluk: site 16.5, *Gillespie et al. 12217* (ALTA, CAN 10114946, CHARS, MO, US). Niaqurnaaluk-Qassialuit: site 18.1, *Gillespie et al. 12230* (CAN 10114947, MT), *12238* (ALA, CAN 10114948, npsp, NY, UBC). Tingijattut: site 15.9, *Gillespie et al. 12179* (CAN 10114945, MT, QFA). **Clyde Area.** Clyde River: site CR-1, *Dutilly 9387* (CAN 10019804, QFA0021042, QFA0133023), *Polunin 610* (CAN 10019809). Inugsuin Head: site IG-3, *Parmelee & Seaborn 4005* (BABY 06180, DAO 01-01658734). Inugsuin Mouth: site IM-1, *Hainault 3967A* (CAN 10019819, DAO 01-01658731, H 1176537, O V546336, QK 59796). Kangiqtugaapik Head: site KP-1, *Wynne-Edwards 9014* (CAN 10019917); site KP-5, *Wynne-Edwards 9053* (CAN 10019914); site KP-28, *Wynne-Edwards 9069* (CAN 10019913). **Home Bay.** Ekalugad FOX-C: site FC-1, *Richardson & Webber 68* (CAN 10019827, COLO 01586544, QK 71126). Kangirlugag Fiord: site KG-1, *Webber 1308* (COLO 01586577).

##### Puccinellia tenella (Lange) Holmb. subsp. langeana (Berlin) Tzvelev (≡*Puccinellia langeana* (Berlin) T.J.Sørensen) —Lange’s alkaligrass—Amphi-Beringian?–North American (N)

**Specimens examined: Agguttinni TP.** Atagulisaktalik: site 23.9, *Gillespie et al. 11527* (CAN 10114937); site AT-1, *Dansereau 500723-1275* (MT00286453, MT00286471). Kangiqtualuk Agguqti: site 6.3, *Gillespie et al. 11881* (CAN 10114939); site 8.1, *Gillespie et al. 11905* (CAN 10114940). Kangiqtualuk Uqquqti: site 30.5, *Gillespie et al. 11722* (ALA, ALTA, CAN 10114938, CHARS, MO, MT, npsp). Refuge Harbour: site 12.4, *Gillespie et al. 12043* (CAN 10114941, MT, NY, US, WIN). Tingijattut: site 15.9, *Gillespie et al. 12185* (ALA, ALTA, CAN 10114942, CHARS, MO, MT, NFLD, npsp, QFA, US), *12186* (CAN 10114943). **Clyde Area.** Inugsuin Head: site IG-1, *Hainault 3994* (CAN 10020028, DAO 01-01657469, H 1176254, O V546287, QK 59801). Inugsuin Mouth: site IM-1, *Hainault 3967b* (CAN 10020054, DAO 01-01659380, H 1176391, O V546304, QK 59837). Kangiqtugaapik Head: site KP-28, *Wynne-Edwards 9068* (CAN 10020008). **Home Bay.** Ekalugad FOX-C: site FC-1, *Richardson & Webber 72* (CAN 10020052, COLO 01530526, QK 71116). Kangirlugag Fiord: site KG-1, *Webber 1303* (COLO 01530500, QK 71115), *1190* (COLO 01530716).

##### *Puccinellia vahliana* (Liebm.) Scribn. & Merr. (≡*Colpodium vahlianum* (Liebm.) Nevski)—Vahl’s alkaligrass—North American (N)–Amphi-Atlantic

This species is known only from Kangiqtugaapik Head in the flora area, as was first mapped by Porsild (1957) and Porsild and Cody (1980). Aiken et al. (2007) incorrectly mapped three additional localities in the flora area: Inugsuin Head (based on *Hainault 3673*CAN, *3878*CAN), Ekalugad Fox-C (*Richardon & Webber 35*CAN), and Cape Hooper (based on specimens at O). This was a mapping error since the CAN specimens are *Potentilla vahliana* and the O collections are likely that species (there are several O specimens of *Potentilla vahliana* from Cape Hooper, but the Aiken et al. mapping database gives insufficient information to determine which specimens the dots are based on). Elsewhere on Baffin Island, the species is scattered and generally uncommon.

**Specimens examined: Clyde Area.** Kangiqtugaapik Head: site KP-5, *Wynne-Edwards 9031* (CAN 10020158); site KP-27, *Wynne-Edwards 9070* (CAN 10020180).

#### ﻿Tofieldiaceae

***Tofieldia* Hudson**

##### Key to species of *Tofieldia* [adapted from Packer (2002)]

**Table:** 

1	Inflorescence bracts deeply 3-lobed; bracteoles absent; tepals white or cream; capsules 2.5–3 mm long	***T. pusilla***
–	Inflorescence bracts ovate, not lobed (sometimes absent distally); bracteoles 3-lobed; tepals pale pink or whitish, usually tinged pink to deep purplish; capsules 2–2.3 mm long	***T. coccinea***

##### *Tofieldia coccinea* Richardson—Northern tofieldia—Asian (N/C)–amphi-Beringian–North American (N)

Taylor (1863) recorded a species of *Tofieldia* (*T. palustris* “L.” [*T. palustris* Huds. is a synonym of the white-flowered European *T. calyculata* (L.) Wahlenb.]) from Cape Adair and Scott’s Bay, but no collections located; given the locations, his records likely refer to *T. coccinea*.

**Specimens examined: Agguttinni TP.** Kangiqtualuk Agguqti: site 6.1, *Gillespie et al. 11874* (ALTA, CAN 10114932); site 8.2, *Gillespie et al. 11941* (CAN 10114928). Kangiqtualuk Uqquqti: site 28.1, *Gillespie et al. 11624* (CAN 10114930, MT). Refuge Harbour: site 12.8, *Gillespie et al. 12056* (CAN 10114929, MO). Stewart Valley: site 15.1, *Gillespie et al. 12125* (CAN 10114757). Tasialuk S: site 26.5, *Gillespie et al. 11596* (CAN 10114931, CHARS, npsp). **Clyde Area.** Inugsuin Head: site IG-9, *Parmelee & Seaborn 3982* (DAO 01-01667558); site IG-24, *Hainault 3721* (CAN 10044055, H 1313079, QK 59605). Kangiqtugaapik Head: site KP-1, *Wynne-Edwards 8920* (CAN 10044051), *8967* (CAN 10044050), *8971* (CAN 10044049), *9042* (CAN 10044057); site KP-20, *Dansereau 500731-0281* (DAO 01-01667559, MT00285801). McBeth Valley: site MB-1, *Hainault 3761* (QK 59661).

##### *Tofieldia pusilla* (Michx.) Pers.—Small tofieldia—Circumpolar-alpine

Found in the flora area only from the heads of three fiords, these collections are among the northernmost records in the eastern Canadian Arctic. Only two collections are known from further north (*Morris s.n.*ALTA 86675 from Ellesmere Island (det. J.G. Packer) and *Bennett et al. 16-0520*BABY from northern Baffin Island, neither verified here). Elsewhere in the Canadian Arctic, it is known from southern and western Baffin Island, Southampton and Victoria Islands, and from across the mainland Arctic and subarctic (Porsild 1957; Porsild and Cody 1980; Aiken et al. 2007; Saarela et al. 2020b).

**Specimens examined: Agguttinni TP.** Kangiqtualuk Agguqti: site 8.2, *Gillespie et al. 11940* (CAN 10114881). Kangiqtualuk Uqquqti: site 28.1, *Gillespie et al. 11623* (CAN 10114882, CHARS, MT, npsp). **Clyde Area.** Kangiqtugaapik Head: site KP-1, *Dansereau 500703-0857* (MT00285800), *500731-0279* (MT00286629), *500803-0862* (MT00286628), *Wynne-Edwards 8970* (CAN 10042146); site KP-5, *Wynne-Edwards 9026* (CAN 10042159); site KP-17, *Wynne-Edwards 9044* (CAN 10042158); site KP-24, *Wynne-Edwards 9065* (CAN 10042147).

##### Tofieldia coccinea × pusilla

Plants intermediate between the two *Tofieldia* species were identified from one site (collections of the parent species were not made at this site, although they are present at the locality). They had the three-lobed inflorescence bract of *T. pusilla* and the stem bract and pink- or purplish-tinged flowers and flowering stem characteristic of *T. coccinea*.

**Specimens examined: Agguttinni TP**. Kangiqtualuk Agguqti: site 8.7, *Gillespie et al. 11977* (CAN 10114883).

### ﻿﻿Eudicots

**Asteraceae**

#### ﻿*Antennaria* Gaertn.

Antennaria is a taxonomically difficult genus with conflicting recent taxonomies. Here, we follow Chmielewski (1994), Chmielewski (1997), and Elven et al. (2011), rather than Bayer (2006).

##### Key to species of *Antennaria* [adapted from Chmielewski (1994), Chmielewski (1997), and Saarela et al. (2020b)]

**Table:** 

1	Basal leaf blades linear-oblanceolate to narrowly spathulate-oblanceolate, apex acute or acuminate, abaxial surfaces tomentose, adaxial green-glabrescent to gray-pubescent; stems tomentose and stipitate-glandular	**A. friesiana subsp. friesiana**
–	Basal leaf blades spathulate to spathulate-oblanceolate, apex rounded or obtuse, both surfaces densely tomentose; stems densely tomentose, not stipitate-glandular	**A. media subsp. compacta**

##### Antennaria friesiana (Trautv.) E.Ekman. subsp. friesiana (=*Antennaria ekmaniana* A.E.Porsild)— Fries’s pussytoes—Asian (NE)–amphi-Beringian–North American (N)

**Specimens examined: Agguttinni TP.** Atagulisaktalik: site 23.10, *Gillespie et al. 11531* (ALA, CAN 10114958). Clark Fiord: site 20.1, *Gillespie et al. 12328* (CAN 10114950, npsp). Gibbs esker: site 20.12, *Gillespie et al. 12399* (CAN 10114951, MT). Kangiqtualuk Agguqti: site 3.5, *Gillespie et al. 11851* (CAN 10114957, MO, QFA); site 6.3, *Gillespie et al. 11890* (CAN 10114954, NY, US); site 8.9, *Gillespie et al. 11987* (CAN 10114952). Kangiqtualuk Uqquqti: site 28.3, *Gillespie et al. 11654* (CAN 10114956). Kuugaaluk: site 19.3, *Gillespie et al. 12297* (ALA, CAN 10114891, CHARS, UBC). Marble Lake: site 31.1, *Gillespie et al. 11735* (CAN 10114955, CHARS, MT). Stewart Valley: site 15.1, *Gillespie et al. 12127* (CAN 10114953, MT). Tasialuk N: site 26.2, *Gillespie et al. 11579* (ALTA, CAN 10114890, MT, npsp). Tasialuk S: site 26.5, *Gillespie et al. 11590* (CAN 10114889). **Clyde Area.** Hoffman Cove: site HF-1, *Bartlett 238* (US 01730141). Inugsuin Head: site IG-2, *Hainault 3633A* (ACAD 70032, QK 59598); site IG-6, *Parmelee & Seaborn 3863* (DAO 01-01384510); site IG-19, *Hainault 4067* (CAN 10082509, DAO 01-01381218, O V556191, QK 59843); site IG-26, *Hainault 3685* (CAN 10083522, O V556243, QK 59709); site IG-29, *Hainault 4038* (QK 59762). Inugsuin Mouth: site IM-1, *Hainault 3821* (DAO 01-01381470, O V556198, QK 59674), *3932* (CAN 10082510, DAO 01-01381219, O V556192, QK 59779). Kangiqtugaapik Head: site KP-1, *Dansereau 500627-3257* (MT00286132), *500629-2186* (MT00286131), *500811-0855* (MT00286130), *Wynne-Edwards 8875* (CAN 10082496); site KP-8, *Wynne-Edwards 8946* (CAN 10082495, TRH 4427); site KP-20, *Wynne-Edwards 9040* (CAN 10082494, E00607482); site KP-25, *Dansereau 500731-0454* (MT00286133). McBeth Valley: Site MB-1, *Hainault 3754* (CAN 10082517, DAO 01-01381471, O V556199, QK 59590), *3785* (O V556197, QK 59626). **Coutts Inlet.** Coutts Mouth: site CO-1, *Coombs 92* (DAO 01-01381572). **Home Bay.** Cape Hooper: site HO-1, *Elven 3506/99* (ALA V131974, CAN 10082508). Ekalugad FOX-C: site FC-1, *Crompton et al. s.n.* (COLO 01195718), *Richardson & Webber 8* (COLO 01195684), *8b* (COLO 01195692), *67* (CAN 10083281, COLO 01195668). Ekalugad Head: site EK-4, *Philpot et al. s.n.* (COLO 01195700). Kangirlugag Fiord: site KG-1, *Webber 1253* (CAN 10083491, COLO 01195676).

##### Antennaria media Greene subsp. compacta (Malte) Chmiel. (≡*Antennaria compacta* Malte)—Alpine pussytoes—Amphi-Beringian–North American (N)

We follow the taxonomy of Chmielewski (1997) and Elven et al. (2011), rather than Bayer (2006), who included this taxon in a broadly circumscribed *A. alpina* (L.) Gaertn. This species is known from only two localities in the flora area and central Baffin Island, both previously reported and/or mapped (Porsild and Cody 1980; Chmielewski 1997; Aiken et al. 2007). Elsewhere in the Canadian Arctic, it is known primarily from three disjunct areas: southern Baffin Island, Ellesmere and Axel Heiberg islands, and the western Arctic (Porsild and Cody 1980; Chmielewski 1997; Aiken et al. 2007).

**Specimens examined: Clyde Area.** Inugsuin Head: site IG-1, *Hainault 3633B* (QK 59529), *3678* (CAN 10083435, DAO 01-01381221, O V556244, QK 59705); site IG-2, *Hainault 3661* (CAN 10083436, DAO 01-01381220, O V556210, QK 59699). Kangiqtugaapik Head: site KP-24, *Wynne-Edwards 8832* (CAN 10083433), *8834* (CAN 10083430).

#### ﻿*Arnica* L.

##### Arnica angustifolia Vahl. subsp. angustifolia (=Arnica alpina var. angustifolia (Vahl) Fernald)—Narrow-leaved arnica—North American (N)–amphi-Atlantic (Svalbard)

**Specimens examined: Clyde Area.** Inugsuin Head: site IG-6, *Parmelee & Seaborn 3850* (DAO 01-01406187); site IG-26, *Hainault 4027* (CAN 10082853, DAO 01-01406198, QK 59831). Inugsuin Mouth: site IM-1, *Hainault 3847* (DAO 01-01406197, QK 59657), *3953* (ACAD 70031, QK 59785). Kangiqtugaapik Head: site KP-1, *Dansereau 500802-0369* (MT00204146), *Wynne-Edwards 8942* (CAN 10082869, TRH 4371); site KP-20, *Wynne-Edwards 8994* (CAN 10082868); site KP-27, *Wynne-Edwards 9064* (CAN 10082870).

#### ﻿*Askellia* W.A.Weber

##### *Askellia pygmaea* (Ledeb.) Sennikov (=*Crepis nana* Richardson; *Askellia nana* (Richardson) W.A.Weber)—Dwarf hawksbeard—Asian (C-NE)–amphi-Beringian–North American (N)

This species is a habitat specialist on scree and gravel and has a very scattered distribution in the Canadian Arctic (Porsild 1957; Aiken et al. 2007; Saarela et al. 2020b).

**Specimens examined: Agguttinni TP.** Atagulisaktalik: site AT-1, *Röthlisberger s.n.* (CAN 10084414), *Wynne-Edwards 9100* (CAN 10084412, E00554397). Gibbs Fiord: site GF-1, *Dansereau 500724-0697* (MT00286206). Kangiqtualuk Uqquqti: site 30.7, *Gillespie et al. 11731* (CAN 10114949). Swiss Bay: site SB-1, *Röthlisberger s.n.* (CAN 10084413). **Home Bay.** Ekalugad Head: site EK-1, *Ryder s.n.* (COLO 01211572).

#### ﻿*Erigeron* L.

##### Key to species of *Erigeron*

**Table:** 

1	Leaf blades ternately deeply lobed or dissected	***E. compositus***
–	Leaf blades not lobed or dissected	***E. eriocephalus***

##### *Erigeron compositus* Pursh—Cut-leaved fleabane—Amphi-Beringian–North American (N)–Cordilleran

This species was found only at a single locality, Kangiqtugaapik Head, in the flora area and on central Baffin Island. Elsewhere in the Canadian Arctic, it has a scattered distribution, known from several localities on northern Baffin Island, Axel Heiberg and Ellesmere islands, the western Arctic islands and mainland, and several sites in northern Quebec (Porsild 1957; Porsild and Cody 1980; Aiken et al. 2007; Saarela et al. 2020b; Payette 2023a).

**Specimens examined: Clyde Area.** Kangiqtugaapik Head: site KP-1, *Dansereau 500703-0356* (MT00204134), *500703-0562* (MT00204136), *500703-1451* (MT00204138), *500708-0556* (MT00204141, MT00204142), *500803-0476* (MT00204135), *500823-0681* (MT00204140); site KP-13, *Dansereau 500608-0355* (MT00204133); site KP-17, *Dansereau 500623-1852* (MT00204139); site KP-17, *Wynne-Edwards 8895* (CAN 10086650); site KP-18, *Wynne-Edwards 9047* (CAN 10086657); site KP-25, *Wynne-Edwards 9061* (CAN 10086649).

##### *Erigeron eriocephalus* J.Vahl. (≡Erigeron uniflorus L. subsp. eriocephalus (J.Vahl) Cronquist)—One-flowered fleabane—Circumpolar

Also recorded from Cape Adair and Scott’s Bay (as *Erigeron uniflorus*) (Taylor 1863), but no collections located. We did not find the species in Agguttinni TP or Clyde River in 2021, possibly because of the cold summer and short growing season that year.

**Specimens examined: Agguttinni TP.** Atagulisaktalik: site AT-4, *Röthlisberger s.n.* (CAN 10087006). Gee Lake: site GE-1, *Dansereau 500811-0252* (MT00165025). **Clyde Area.** Clyde River: site CR-1, *Martin 43* (DAO 01-01370117, TRT00042293). Kangiqtugaapik Head: site KP-8, *Dansereau 500822-1776* (MT00273178); site KP-18, *Wynne-Edwards 9046* (CAN 10087156); site KP-20, *Wynne-Edwards 8995* (CAN 10086981), *9041* (CAN 10087005); site KP-25, *Dansereau 500731-0453* (MT00273179); site KP-27, *Dansereau 500716-0666* (MT00273176). **Home Bay.** Ekalugad Head: site EK-1, *Stock s.n.*. (COLO 01267780).

#### *Hulteniella* Tzvelev

##### *Hulteniella integrifolia* (Richardson) Tzvelev (≡ *Chrysanthemum integrifolium* Richardson) (Fig. 12F)—Entire-leaved daisy—Amphi-Beringian–North American (N)

Widespread on the Canadian Arctic Islands and adjacent mainland, this species is newly reported for the flora area here and in Gillespie et al. (2024). Although Aiken et al. (2007) mapped this species from the Clyde area purportedly based on Porsild and Cody (1980), this appears to be a mistake. Porsild and Cody (1980) did not map the species in the flora area, and there is no record of a collection in the Aiken et al. (2007) mapping database.

**Specimens examined: Agguttinni TP.** Kangiqtualuk Agguqti: site 8.4, *Gillespie et al. 11958* (CAN 10114897). Kangiqtualuk Uqquqti: site 28.6, *Gillespie et al. 11672* (CAN 10114896, MT, npsp).

#### ﻿*Taraxacum* F.H.Wigg.

*Taraxacum* is a taxonomically difficult genus; here we follow Brouillet (2006).

##### ﻿Key to species of *Taraxacum* [adapted from Brouillet (2006)]

**Table:** 

1	All or some phyllary apices notably horned; calyculus bractlets notably horned	***T. ceratophorum***
–	Phyllary apices usually hornless (sometimes callous or small horns in *T. holmenianum*); calyculus bractlets usually hornless or horns relatively small	**2**
2	Leaf blade margins usually entire or toothed to denticulate (sometimes somewhat runcinate and then mostly irregularly and shallowly triangular-lobed); corollas pale yellow, sometimes lemon-colored; cypselae dark brown, grayish, or blackish, muricate 1/2–3/4+	***T. phymatocarpum***
–	Leaf blade margins runcinate, regularly and usually deep triangular-lobed; corollas dark yellow; cypselae yellowish or straw-colored, muricate in distal 1/2	***T. holmenianum***

##### *Taraxacum ceratophorum* (Ledeb.) DC. (=*Taraxacum lacerum* Greene)—Horned dandelion—Circumboreal-polar

**Specimens examined: Agguttinni TP.** Atagulisaktalik: site 23.11, *Gillespie et al. 11535* (CAN 10114989, CHARS, MT). Clark Fiord: site 20.3, *Gillespie et al. 12364* (CAN 10114987, npsp, NY, QFA, UBC, US). Kangiqtualuk Agguqti: site 6.4, *Gillespie et al. 11892* (CAN 10114985, MT, US); site 8.10, *Gillespie et al. 11988* (CAN 10114984, UBC, WIN). Kangiqtualuk Uqquqti: site 30.3, *Gillespie et al. 11715* (CAN 10114990, MO, QFA). Kuugaaluk: site 19.3, *Gillespie et al. 12298* (CAN 10114981, npsp); site 19.4, *Gillespie et al. 12309* (ALA, ALTA, CAN 10114988, CHARS, MO, MT). Remote Peninsula: site RL-1, *Philpot et al. s.n.* (COLO 01382662). Tasialuk N: site 26.3, *Gillespie et al. 11582* (ALA, ALTA, CAN 10114986, MT). Tasialuk S: site 21.12, *Gillespie et al. 12447* (CAN 10114982). Tingijattut: site 15.5, *Gillespie et al. 12167* (CAN 10114983, MT); site TG-2, Dare et al. 84 (CAN 10092116). **Clyde Area.** Clyde River: site CR-4, *Dansereau 500730-1191* (MT00059333). Inugsuin Head: site IG-1, *Hainault 3709* (QK 59638); site IG-2, *Hainault 3655* (CAN 10085248, DAO 01-01001129412, QK 59694). Inugsuin Mouth: site IM-1, *Hainault 3951* (DAO 01-01001129411, QK 59783), *3966* (CAN 10085387, QK 59836). Kangiqtugaapik Head: site KP-1, *Dansereau 500708-0555* (MT00059331), *500726-1169* (MT00059319), *Wynne-Edwards 8964* (CAN 10085116), *9003* (CAN 10085078); site KP-12, *Wynne-Edwards 8965* (CAN 10085113); site KP-15, *Dansereau 500718-0651* (MT00059330); site KP-17, *Wynne-Edwards 8897* (CAN 10085287); site KP-21, *Dansereau 500730-0951* (MT00059329); site KP-24, *Wynne-Edwards 8913* (CAN 10085289). **Home Bay.** Ekalugad Head: site EK-2, *Stock s.n..* (COLO 01382845).

##### *Taraxacum holmenianum* Sahlin (≡*Taraxacum pumilum* Dahlst. non Gaudich. nom. illeg.)—Holmen’s dandelion—North American (N)

This species is newly reported for the flora area here and in Gillespie et al. (2024); it is known only from two sites in Agguttinni TP. Aiken et al. (2007) mapped the species from Kangiqtugaapik Head (based on *Wynne-Edwards 8897*CAN) and Inugsuin Head (*Hainault 3655*CAN), but these collections are reidentified here as *T. ceratophorum.* The species is known from across the Canadian Arctic Islands (Aiken et al. 2007; Saarela et al. 2020b), but is rare on Baffin Island, where it is known from only three other collections and sites (Isortoq River, *Webber 444*CAN; Katannilik TP, *Saarela et al. 2420*CAN (Saarela et al. 2023); Resolution Island, *Wynne-Edwards 7229*CAN).

**Specimens examined: Agguttinni TP.** Kangiqtualuk Uqquqti: site 29.2, *Gillespie et al. 11690* (CAN 10114980). Kuugaaluk: site 16.5, *Gillespie et al. 12214* (CAN 10114979, MT, npsp).

##### *Taraxacum phymatocarpum* J.Vahl—Northern dandelion—Circumpolar

**Specimens examined: Agguttinni TP.** Atagulisaktalik: site 23.10, *Gillespie et al. 11528* (CAN 10114991). Kangiqtualuk Agguqti: site 8.2, *Gillespie et al. 11921* (CAN 10114992). Kangiqtualuk Uqquqti: site 30.3, *Gillespie et al. 11714* (CAN 10114993). Remote Peninsula: site RL-1, *Philpot et al. s.n.* (COLO 01382647). **Clyde Area.** Inugsuin Head: site IG-1, *Hainault 3696* (CAN 10085341, O V556498, QK 59717). Inugsuin Head: site IG-17, *Hainault 3727* (CAN 10088856, DAO 01-01001129960, O V556499, QK 59611). Kangiqtugaapik Head: site KP-1, *Dansereau 500710-0364* (MT00059324), *Wynne-Edwards 8929* (CAN 10088858); site KP-5, *Wynne-Edwards 9059* (CAN 10088857, LSU00067072); site KP-8, *Wynne-Edwards 9002* (CAN 10088861); site KP-22, *Dansereau 500710-3879* (MT00059323); site KP-24, *Wynne-Edwards 8912* (CAN 10088853).

#### ﻿*Tripleurospermum* (L.) Sch.Bip.

##### Tripleurospermum maritimum (L.) W.D.J.Koch subsp. phaeocephalum (Rupr.) Hämet-Ahti (≡Matricaria maritima L. subsp. phaeocephala (Rupr.) Rauschert) (Fig. 9H)—Arctic chamomile—Circumpolar

We consider this species as newly reported for the flora area here and in Gillespie et al. (2024) based on a single collection. Porsild (1957) and Porsild and Cody (1980) mapped the species (as *Matricaria ambigua* (Ledeb.) Maxim. ex Kom.) from the Agguttinni TP area, but no specimen has been located for verification, nor is it mentioned in previous literature; we consider it a possible mapping error. Aiken et al. (2007) subsequently mapped the species based on the map in Porsild and Cody (1980). In the Canadian Arctic, this species is mostly scattered along seashores; reports from northern Baffin Island are the northernmost records in the Eastern Canadian Arctic (Porsild and Cody 1980; Aiken et al. 2007; plus verified iNaturalist observations).

**Specimens examined: Agguttinni TP.** Niaqurnaaluk-Qassialuit: site 18.1, *Gillespie et al. 12235* (CAN 10114893, npsp).

#### ﻿Boraginaceae

***Mertensia* Roth**

##### Mertensia maritima (L.) Gray subsp. tenella (Fr.) Elven & Skarpaas—Arctic seaside bluebells—Amphi-Beringian–North American (N)–amphi-Atlantic (Svalbard, Jan Meyen)

**Specimens examined: Agguttinni TP.** Kuugaaluk: site A22.3, *Gillespie et al. 12457* (CAN 10114895, UBC, US). Niaqurnaaluk-Qassialuit: site 18.1, *Gillespie et al. 12234* (ALA, ALTA, CAN 10114894, CHARS, MO, MT, npsp, QFA). **Clyde Area.** Clyde River: site CR-1, *Dansereau 500716-1174* (MT00285499). Inugsuin Head: site IG-8, *Parmelee & Seaborn 3952* (DAO 01-01485545). Kangiqtugaapik Head: site KP-1, *Wynne-Edwards 8914* (CAN 10077975); site KP-2, *Martin 55* (DAO 01-01485550).

#### ﻿Brassicaceae

***Arabidopsis* Heynh.**

##### *Arabidopsis arenicola* (Richardson ex Hook.) Al-Shehbaz, Elven, D.F.Murray & S.I.Warwick (≡*Arabis arenicola* (Richardson ex Hook.) Gelert) (Fig. 9E)—Arctic rockcress—North American (NE)

This species is newly recorded for the flora area here and in Gillespie et al. (2024), based on a single collection from shifting sand on the steep side of an inland valley. It is among the northernmost records in Canada, along with one collection from Ellesmere Island (*Brassard 1731*CAN; a possible second locality mapped in Porsild (1957) and Porsild and Cody (1980) but not verified), one from west central Baffin Island (Isortoq Valley, *Webber 69*CAN, mapped in Aiken et al. (2007)), and two from northern Baffin Island (*Bennett 16-0528*BABY [not verified], *Drury 5491*CAN).

**Specimens examined: Agguttinni TP.** Tasialuk S: site 21.12, *Gillespie et al. 12438* (CAN 10114994, MT, npsp).

#### ﻿*Braya* Sternb. & Hoppe

##### Braya glabella Richardson subsp. purpurascens (R.Br.) Cody (≡*Braya purpurescens* R.Br.)—Purple braya—Circumpolar–Cordilleran

Braya glabella subsp. purpurascens is found throughout the Canadian Arctic Archipelago. A second subspecies, subsp. glabella, more southern Arctic in distribution, was mapped on Kangiqtualuk Uqquqti (within Agguttinni TP) in Aiken et al. (2007) based on a map in Harris (1985); unfortunately, Harris did not cite this specimen. This locality is considerably further north than the two known localities of subsp. glabella on southern Baffin Island (Saarela et al. 2023). The two subspecies may be distinguished by their fruit shape and relative width and raceme elongation (Harris 2010), characters that often overlap. All specimens of *B. glabella* examined here were identified as subsp. purpurescens.

**Specimens examined: Agguttinni TP.** Kangiqtualuk Agguqti: site 8.3, *Gillespie et al. 11953* (CAN 10114996, npsp). Kangiqtualuk Uqquqti: site 28.1, *Gillespie et al. 11627* (CAN 10114995, npsp) **. Clyde Area.** Kangiqtugaapik Head: site KP-1, *Dansereau 500702-1591* (MT00286110, MT00286111); site KP-4, *Dansereau 500711-5658* [pro parte] (MT00286879, MT00286880); site KP-5, *Wynne-Edwards 8851* (CAN 10052920); site KP-7, *Dansereau 500801-0661* (MT00286114, MT00286115); site KP-11, *Wynne-Edwards 8884* (CAN 10052921), *9027* (CAN 10053122); site KP-18, *Dansereau 500707-0852* (MT00286112, MT00286113); site KP-27, *Wynne-Edwards 8922* (CAN 10052922). **Home Bay.** Ekalugad FOX-C: site FC-1, *Richardson & Webber 10* (CAN 10052896, COLO 01938968).

#### ﻿*Cardamine* L.

##### *Cardamine bellidifolia* L.—Alpine bittercress—Circumpolar-alpine

Also recorded from Scott’s Bay (Taylor 1863), but no collection located.

**Specimens examined: Agguttinni TP.** Atagulisaktalik: site 22.2, *Gillespie et al. 11482* (CAN 10116452); site 24.2, *Gillespie et al. 11549* (CAN 10116453). Clark Fiord: site 20.7, *Gillespie et al. 12372* (CAN 10116459, MO, npsp). Generator Lake: site GL-21, *Raynolds & Bültmann MKR-2022-42* (CAN 10169279). Kangiqtualuk Agguqti: site 5.3, *Gillespie et al. 11865* (CAN 10116455). Kangiqtualuk Uqquqti: site 28.2, *Gillespie et al. 11648* (CAN 10116454, CHARS). Kuugaaluk: site 19.2, *Gillespie et al. 12295* (CAN 10116458, MT). Refuge Harbour: site 11.1, *Gillespie et al. 12012* (CAN 10116456). Tingijattut: site 15.4, *Gillespie et al. 12162* (CAN 10116457). **Clyde Area**. Clyde River: site CR-1, *Martin 66* (DAO 01-01000556466), *Polunin 625* (CAN 10054144); site CR-2, *Forbes 92* (DAO 01-01000556404), *109* (DAO 01-01000556423). Inugsuin Head: site IG-9, *Parmelee & Seaborn 3940* (DAO 01-01000556463); site IG-14, *Parmelee & Seaborn 3903* (DAO 01-01000556462). Inugsuin Mouth: site IM-1, *Hainault 3941* (DAO 01-01000556405). Kangiqtugaapik Head: site KP-1, *Dansereau 500705-0268* [pro parte, plants 2] (MT00286030), *500714-0153* (MT00286121), *Wynne-Edwards 8860* (CAN 10051978), *8878-B* (CAN 10051979); site KP-11, *Wynne-Edwards 9094* (CAN 10054110); site KP-25, *Wynne-Edwards 9062* (CAN 10054157). **Home Bay.** Cape Hooper: site HO-1, *Elven 3449/99* (CAN 10054125), *3463/99* (ALA V133477, O V548877). Ekalugad Head: site EK-1, *Church s.n.* (UBC V162145), *Ryder s.n.* (COLO 01940477). Kangirlugag Fiord: site KG-1, *Webber 1252* (CAN 10054149), *1274* (COLO 01940469).

#### ﻿*Cochlearia* L.

##### *Cochlearia groenlandica* L. (≡Cochlearia officinalis L. subsp. groenlandica (L.) A.E.Porsild)—Greenland scurvygrass—Circumpolar

Also recorded from Scott’s Bay (as *C. officinalis*) (Taylor 1863), but no collection located.

**Specimens examined: Agguttinni TP.** Atagulisaktalik: site 23.10, *Gillespie et al. 11529* (CAN 10116461, CHARS, MO); site AT-3, *Röthlisberger 96* (ZT-00137297); site AT-5, *Röthlisberger 50* (ZT-00137294), *64* (ZT-00137293), *125* (ZT-00137295). Kangiqtualuk Agguqti: site 8.3, *Gillespie et al. 11952* (CAN 10116462). Kangiqtualuk Uqquqti: site 29.3, *Gillespie et al. 11695* (CAN 10116460). Kuugaaluk: site 16.1, *Gillespie et al. 12196* (CAN 10116464, MT). Niaqurnaaluk-Qassialuit: site 18.1, *Gillespie et al. 12236* (CAN 10116463, npsp). **Clyde Area.** Clyde River: site CR-1, *Martin 22* (DAO 01-01000524495); site CR-2, *Forbes 50* (DAO 01-01000524404), *65* (DAO 01-01000524403), *102* (DAO 01-01000524406); site CR-3, *Dare 54* (CAN 10092130); site CR-4, *Dansereau 500730-1196* (MT00286128). Inugsuin Head: site IG-2, *Hainault 3689* (DAO 01-01000524402, MT - no acc. num., O V548968, QK18297633). Inugsuin Mouth: site IM-1, *Hainault 3829* (CAN 10053379, DAO 01-01000524401, O V548967, QK18297634). Kangiqtugaapik Head: site KP-1, *Dansereau 500614-1754* (MT00286126), *500719-0254* (MT00286127); site KP-11, *Wynne-Edwards 8885* (CAN 10053435); site KP-14, *Röthlisberger 34* (ZT-00137296); site KP-3, *Dansereau 500714-0156* (MT00286617); site KP-4, *Dansereau 500711-5658* [pro parte] (MT00286125); site KP-6, *Dansereau 500630-2059* (MT00286124); site KP-7, *Dansereau 500801-0664* (MT00286618); site KP-8, *Wynne-Edwards 9004* (CAN 10053474). **Home Bay.** Ekalugad FOX-C: site FC-1, *Richardson & Webber 60* (CAN 10053424, COLO 01950591, QK 71106), *70* (COLO 01950609).

#### ﻿*Crucihimalaya* Al-Shehbaz, O’Kane & R.A.Price

##### *Crucihimalaya bursifolia* (DC.) D.A.German & A.L.Ebel (≡*Transberingia bursifolia* (DC.) Al-Shehbaz & O’Kane; =*Halimolobos mollis* (Hook.) Rollins) (Fig. 9A)—Soft fissurewort—Asian (NE)–amphi-Beringian–North American (N)

This species is known from only one locality in the flora area, which is also the only locality known for Baffin Island. At the time it was first mapped by Porsild (1957), it was the only locality known in the Canadian Arctic Islands. The species was subsequently mapped from Banks (Porsild and Cody 1980) and Ellesmere islands (Aiken et al. 2007) and reported from Victoria Island (Saarela et al. 2020b). The flora area locality is over 500 km from the closest locality, in western Greenland, and over 1000 km from the closest locality in the Canadian Arctic, Alexandra Fiord on eastern Ellesmere Island. This peculiar distribution of a few highly disjunct populations may be due to dispersal by animals or humans (Porsild 1957). Persistence of these populations should be studied. This species is considered vulnerable in Nunavut (Canadian Endangered Species Conservation Council 2022).

**Specimens examined: Clyde Area.** Kangiqtugaapik Head: site KP-1, *Wynne-Edwards 8853* (CAN 10057913); site KP-8, *Wynne-Edwards 8945* (CAN 10057914).

#### ﻿*Draba* L.

We follow Al-Shehbaz et al. (2010) for the taxonomy of North American *Draba*.

##### Key to species of *Draba* [adapted from Al-Shehbaz et al. (2010)]

**Table:** 

1	Flowers bright yellow	***D. corymbosa***
–	Flowers white	**3**
2	Ovaries and fruits densely covered in multi-rayed stellate hairs	**4**
–	Ovaries and fruits glabrous or with sparse simple or 2–4-rayed hairs (*D. nivalis* rarely with few stellate hairs, sometimes many-rayed, near valve margins)	**6**
3	Leaf blade surfaces with simple and stalked 2–3-rayed trichomes (0.4–1 mm long) and with shorter 8–12-rayed trichomes	***D. oblongata***
–	Leaf blade surfaces with short-stalked 8–12-rayed trichomes (*D. arctica* also with simple and 2-rayed hairs near leaf apex)	**5**
4	Stems leafless or 1(–3)-leaved; basal leaf blades with apical tuft of simple and/or 2-rayed trichomes, midveins distinct abaxially; petals 3.5–6 mm long; seeds (0.8–)0.9–1.1 × (0.6–)0.7–0.8 mm	***D. arctica***
–	Stems 1–3(–5)-leaved; basal leaf blades lacking simple hairs (except cilia mostly on proximal margin), midveins usually obscure abaxially; petals 3.5–4.5 mm long; seeds 0.6–0.8 × 0.4–0.6 mm	***D. cinerea***
5	Basal leaf blades 10–25 (–35) mm long, usually denticulate (smaller leaves sometimes entire); stems with long simple and 2-rayed hairs (to 1 mm long) and multi-rayed hairs (to 0.3 mm long); cauline leaves 1+, well developed	***D. glabella***
–	Basal leaf blades 2–12 mm long, usually entire, sometimes with 1 (2) teeth/side; stems glabrous or with 2- to multi-rayed hairs (to 0.3 mm long) or simple and 2-rayed hairs (to 0.6 mm long); cauline leaves 0 or 1 (2), bractlike	**7**
5	Stems hairy throughout; rachises hairy; sepals with simple and/or 2–5-rayed hairs; fruits glabrous or with few hairs	**8**
–	Stems glabrous or sometimes with few hairs proximally (*D. lactea*); rachises glabrous; sepals glabrous or with simple hairs; fruits glabrous	**9**
7	Basal leaf blades not ciliate, abaxial surface with many rayed stellate hairs, ± 1 mm long; fruits elliptic to narrowly oblong-elliptic, 1.5–2.2 mm wide, flattened, glabrous or with few multi-rayed hairs	***D. nivalis***
–	Basal leaf blades ciliate, abaxial surface with simple and 2-rayed hairs (0.3–0.8 mm long) and sometimes branched hairs, 0.1–0.3 mm long; fruits ovoid to oblong, 2–3 mm wide, slightly inflated, glabrous or with simple hairs	***D. subcapitata***
8	Petals 2–2.5 mm long; leaf blade abaxial surface glabrous or with simple or sometimes 2-rayed hairs; sepals glabrous or with scattered simple hairs; fruits lanceolate, elliptic-lanceolate, or oblong-lanceolate, 1.5–2 mm wide	***D. fladnizensis***
–	Petals 3–5 mm long; leaf blade abaxial surface usually with multi-rayed hairs distally or sometimes glabrous: sepals glabrous or with few simple hairs distally; fruits usually ovate or broadly ovate, sometimes oblong to elliptic-lanceolate, (1.5–)2–3 mm wide	***D. lactea***

##### *Draba arctica* J.Vahl—Arctic draba—amphi-Atlantic (Svalbard)

Aiken et al. (2007) first mapped this species in the flora area, from Kangiqtugaapik Head based on collections cited here and from Inugsuin Head based on *Hainault 3680*; we have redetermined the latter collection as *Draba cinerea*.

**Specimens examined: Agguttinni TP.** Kangiqtualuk Agguqti: site 8.4, *Gillespie et al. 11956* (CAN 10114997, npsp). Kangiqtualuk Uqquqti: site 29.2, *Gillespie et al. 11692b* (CAN 10114998). **Clyde Area**. Kangiqtugaapik Head: site KP-1, *Dansereau 500703-0366* [pro parte, plants 2] (MT00285847), *Wynne-Edwards 8899* (CAN 10094294); site KP-11, *Wynne-Edwards 8883* (CAN 10053987); site KP-21, *Dansereau 500611-1451* [pro parte, plants 2] (MT00285845), *500730-0952A* (MT00285843).

##### *Draba cinerea* Adams—Grey-leaved draba—Circumboreal-polar

**Specimens examined: Agguttinni TP.** Gibbs esker: site 20.12, *Gillespie et al. 12403* [cf.] (CAN 10116447). Kangiqtualuk Agguqti: site 8.2, *Gillespie et al. 11913* (CAN 10116439); site 8.4, *Gillespie et al. 11957* (CAN 10116445, MO); site 8.6, *Gillespie et al. 11972* (CAN 10116443, CHARS, MT); site 21.5, *Gillespie et al. 12426* (CAN 10116436, npsp); site 27.1, *Gillespie et al. 11616* (CAN 10116442). Kangiqtualuk Uqquqti: site 29.2, *Gillespie et al. 11689* (CAN 10116444), *11692a* (CAN 10116437); site 30.6, *Gillespie et al. 11730* (CAN 10116438). Kuugaaluk: site 19.4, *Gillespie et al. 12313* (CAN 10116446). Tasialuk S: site 21.12, *Gillespie et al. 12445* (CAN 10116440). **Clyde Area**. Inugsuin Head: site IG-26, *Hainault 3680* (CAN 10073064, DAO 01-01000587804, O V549025, QK18299546). Kangiqtugaapik Head: site KP-1, *Dansereau 500625-0353* (MT00285851), *500628-3257* (MT00285850), *500630-1754* (MT00285848), *500703-0366* [pro parte, plants 1] (MT00285846), *500803-0480B* (MT00285852), *Wynne-Edwards 8876* [pro parte] (CAN 10055018); site KP-5, *Wynne-Edwards 9092* (CAN 10054092); site KP-11, *Wynne-Edwards 8882* (CAN 10056214); site KP-17, *Dansereau 500623-1857* (MT00285849); site KP-21, *Dansereau 500611-1451* [pro parte, plants 1] (MT00285844); site KP-24, *Wynne-Edwards 8837* (CAN 10056208).

##### *Draba corymbosa* R.Br. ex DC. (=*Draba bellii* Holm)—Flat-top draba—Circumpolar

This species is the only yellow-flowered *Draba* species present in the flora area. In the absence of flowers, *D. corymbosa* may be identified by its leaves with large multibranched hairs, often large scattered simple hairs, and long-ciliate margins. The fruiting collection *Gillespie et al. 12208*, previously identified as *D. micropetala* (Gillespie et al. 2024), is here reidentified as D. cf. corymbosa. The abaxial leaf surface and margin indumentum are consistent with *D. corymbosa*; plants lack the subcruciform hairs characteristic of *D. micropetala*. However, style length (0.4-0.5 mm), relative stigma width (equal to or somewhat wider than style), and pedicel length (3–4 mm) are intermediate between the two species (styles 0.6–1.0 mm long, stigma distinctly wider than style, pedicels 4–11(–16) mm in *D. corymbosa*, styles 0.05-0.3 mm long, stigma same width as style, pedicels 1-3(-4) mm in *D. micropetala* according to Al-Shehbaz et al. 2010). This collection is from flat tundra above coastal bluffs in the Coastal Lowland Ecoregion, whereas all other collections of *D. corymbosa* in the flora area are from the heads of fiords in the Baffin Mountain Ecoregion and a single collection from the Baffin Uplands Ecoregion. More collections, specifically flowering ones, are needed for a more definitive identification; the two species differ in petal shape, size, and color.

**Specimens examined: Agguttinni TP.** Kangiqtualuk Uqquqti: site 29.3, *Gillespie et al. 11694* (CAN 10116450). Kuugaaluk: site 16.3, *Gillespie et al. 12208* [cf.] (CAN 10116448). Marble Lake: site 31.2, *Gillespie et al. 11742* (CAN 10116449, npsp). **Clyde Area.** Kangiqtugaapik Head: site KP-1, *Dansereau 500702-1497* (MT00285839), *500810-1254* (MT00285838), *Wynne-Edwards 8878* [7 Jul 1950] (CAN 10054781), *8878-A* (CAN 10056223); site KP-5, *Wynne-Edwards 8840* (CAN 10056233); site KP-11, *Wynne-Edwards 8878* [2 Jul 1950] (CAN 10056272); site KP-16, *Dansereau 500702-1669* (MT00285840), *Wynne-Edwards 9086* (CAN 10056295). **Home Bay**. Kangirlugag Fiord: site KG-1, *Webber 1176* (QK18299443).

##### *Draba fladnizensis* Wulfen—Austrian draba—Circumpolar-alpine

**Specimens examined: Agguttinni TP.** Atagulisaktalik: site AT-1, *Dansereau 500723-0355-56* [pro parte, plants 2] (MT00286062). Kangiqtualuk Uqquqti: site 28.4, *Gillespie et al. 11663b* (CAN 10116451). **Clyde Area.** Hoffman Cove: site HF-1, *Bartlett 221* (US 03751617). Inugsuin Head: site IG-6, *Parmelee & Seaborn 3854* (DAO 01-01000560597); site IG-26, *Hainault 4030* (O V549112, QK18301663). Kangiqtugaapik Head: site KP-1, *Dansereau 500630-1752A* [pro parte, plants 2] (MT00286032), *500630-1753* [pro parte, plants 1] (MT00286064), *500710-0155* [pro parte, plants 1] (MT00286056); site KP-5, *Wynne-Edwards 9093* (CAN 10054950); site KP-24, *Wynne-Edwards 8838* (CAN 10054953). **Coutts Inlet.** Coutts Mouth: site CO-1, *Coombs 81* (DAO 01-01000560599). **Home Bay.** Cape Hooper: site HO-1, *Elven 3484/99* (O V549111).

##### *Draba glabella* Pursh—Smooth draba—Circumboreal-polar

In the flora area, *D. glabella* may be confused with the common *D. lactea* but may be distinguished by its more robust habit, stems with mix of long, simple, and stellate/dendritic hairs (versus stems glabrous or with few, scattered small stellate/dendritic hairs), cauline leaves always present (versus absent or present), many basal leaves denticulate or with a few teeth (versus mostly entire), and leaf abaxial surface evenly covered in stellate hairs (versus glabrous or with stellate hairs clustered near apex). Aiken et al. (2007) also mapped *D. glabella* from the Home Bay area purportedly based on the map in Porsild and Cody (1980), but this appears to be a mistake since the latter includes only the two localities cited here. *Draba hirta* L. may be the correct name for this taxon (Elven et al. 2020, 2022).

**Specimens examined: Clyde Area**. Inugsuin Head: site IG-3, *Parmelee & Seaborn 3995* (DAO 01-01000560703); site IG-10, *Parmelee & Seaborn 3967* (DAO 01-01000560702). Kangiqtugaapik Head: site KP-1, *Dansereau 500710-0156* (MT00286135); site KP-5, *Wynne-Edwards 9091* (CAN10054846); site KP-11, *Wynne-Edwards 8880* (CAN 10054901).

##### *Draba lactea* Adams (Fig. 10E)—Milky draba—Circumpolar

Also recorded from Scott’s Bay (as *Draba lapponica* Willd. ex DC.) (Taylor 1863), but no collection located.

**Specimens examined: Agguttinni TP.** Atagulisaktalik: site 22.2, *Gillespie et al. 11480* (CAN 10116423, CHARS, MT, npsp); site AT-1, *Dansereau 500723-0355-56* [pro parte, plants 1] (MT00286060). Clark Fiord: site 20.4, *Gillespie et al. 12365* (CAN 10116411, MO). Generator Lake: site GL-18, *Raynolds & Bültmann MKR-2022-39* (CAN 10169255). Gibbs esker: site 20.12, *Gillespie et al. 12400* (CAN 10116422). Gibbs Fiord: site 20.10, *Gillespie et al. 12389* (CAN 10116415). Kangiqtualuk Agguqti: site 3.4, *Gillespie et al. 11843* (CAN 10116413); site 3.5, *Gillespie et al. 11855* (CAN 10116412). Kangiqtualuk Uqquqti: site 28.1, *Gillespie et al. 11639* (CAN 10114999, MO), *11640* (CAN 10116418), *11641* (ALTA, CAN 10115000, MO, MT); site 28.4, *Gillespie et al. 11661* (CAN 10116419); site 29.3, *Gillespie et al. 11696* (CAN 10116417), *11697* (CAN 10116416), *11698* (CAN 10116407, MO); site 29.5, *Gillespie et al. 11706* (ALA, CAN 10116408, CHARS, MT, npsp, QFA); site 30.9, *Gillespie et al. 11733* (CAN 10116409, MO). Marble Lake: site 31.1, *Gillespie et al. 11740* (CAN 10116414). Refuge Harbour: site 11.1, *Gillespie et al. 12057* (CAN 10116420); site 11.3, *Gillespie et al. 12059* (CAN 10116421). Remote Peninsula: site RL-1, *Philpot et al. s.n.* (COLO 01958651 [pro parte, plants A]). Tingijattut: site 15.4, *Gillespie et al. 12152* (ALA, CAN 10116410, MT); site TG-2, *Dare et al. 90* (CAN 10092158). **Clyde Area.** Cape Hewett: site CH-1, *Platt 480B* (NY 3185215). Clyde River: site CR-1, *Dutilly 1461* (CM417370), *Martin 65* (DAO 01-01000587101, RBCM V135200); site CR-2, *Forbes 14* (DAO 01-01000587050), *63* (DAO 01-01000568394), *93* (DAO 01-01000568396), *107* (DAO 01-01000587098), *111* (DAO 01-01000568382). Hewett Cache: site HC-1, *Hainault 3807* (QK18299770). Inugsuin Head: site IG-1, *Hainault 3668* (QK18299769); site IG-5, *Parmelee & Seaborn 3969* (DAO 01-01000587038), *3970* (DAO 01-01000568389); site IG-7, *Parmelee & Seaborn 3919* (DAO 01-01000568391), *3922* (DAO 01-01000587112); site IG-9, *Parmelee & Seaborn 3944* (DAO 01-01000568388); site IG-15, *Parmelee & Seaborn 3881* (DAO 01-01000568390); site IG-26, *Hainault 4030* (CAN 10056550, DAO 01-01000587042); site IG-29, *Hainault 3713* (QK18299768). Inugsuin Mouth: site IM-1, *Hainault 3825* (QK18299772), *3837* (QK18299766), *3949* (CAN 10056549, DAO 01-01000568467, O V549178, QK18299767). Kangiqtugaapik Head: site KP-1, *Dansereau 500625-0354* (DAO 01-01000587107, DAO 01-01000568387, MT00198424), *500628-6091* [pro parte, plants 1] (MT00286022), *500630-1753* [pro parte, plants 2] (MT00286066), *500701-1055* (MT00286067), *500710-0155* [pro parte, plants 2] (MT00286058), *500710-0262A* (MT00286074), *500716-0862B* (DAO 01-01000587039), *500725-0162* (MT00286077), *500822-0352* (MT00286080), Wynne-Edwards *8812* (CAN 10056838), *8858* (CAN 10056543); site KP-8, *Dansereau 500705-0268* [pro parte, plants 1] (MT00286029), *500705-0269* (MT00286079); site KP-11, *Wynne-Edwards 8879* (CAN 10056547), *8879-A* (CAN 10056546), *8881* (CAN 10053746); site KP-18, *Wynne-Edwards 8823-A* (CAN 10056544); site KP-24, *Wynne-Edwards 8818* (CAN 10056792); site KP-27, *Dansereau 500716-0570* (MT00286075). McBeth Valley: site MB-1, *Hainault 3794* (QK18299771). **Home Bay.** Cape Hooper: site HO-1, *Elven 3479/99* (ALA V131950, CAN 10056819, O V549192), *3509/99* (O V549193), *3524/99* (O V549194); site HO-3, Parmelee & Seaborn *3802* (DAO 01-0100568383), *3786* (DAO 01-01000568407, DAO 01-01000568449). Ekalugad FOX-C: site FC-1, *Philpot et al. s.n.* (COLO 01958693), *Richardson & Webber 26b* (COLO 01958842, QK18299773), *55* (CAN 10056554, COLO 01958859, QK18299774); site FC-2, *Oswald 362* (QK18266407). Ekalugad Head: site EK-3, *Philpot et al. s.n.* (COLO 01958701). Kangirlugag Fiord: site KG-1, *Webber 1131* (COLO 01958669), *1206* (CAN 10056552, COLO 01958867, QK18299775), Rocknoser Fiord: site RF-1, *Smith VP-93-61* (CAN 10056536).

##### *Draba nivalis* Lilj.—Snow draba—Circumpolar-alpine

Also recorded from Scott’s Bay (as *Draba muricella* Wahlenb.) (Taylor 1863; Polunin 1940), but no collection located.

**Specimens examined: Agguttinni TP.** Atagulisaktalik: site 23.10, *Gillespie et al. 11530a* (CAN 10116424),*11530b* [cf.] (CAN 10116441); site 23.11, *Gillespie et al. 11537* (CAN 10116429); site AT-1, *Dansereau 500723-0355-56* [pro parte, plants 3] (MT00286063). Clark Fiord: site 20.1, *Gillespie et al. 12349* (CAN 10116425); site 20.4, *Gillespie et al. 12366* (CAN 10116435). Generator Lake: site GL-18, *Raynolds & Bültmann MKR-2022-38* (CAN 10169265). Kangiqtualuk Agguqti: site 3.3, *Gillespie et al. 11840* (CAN 10116434); site 8.9, *Gillespie et al. 11985* [cf.] (CAN 10116433); site 21.5, *Gillespie et al. 12425* (CAN 10116432); site 21.7, *Gillespie et al. 12430* (CAN 10116430). Kangiqtualuk Uqquqti: site 28.4, *Gillespie et al. 11663a* (CAN 10116431, npsp); site 30.5, *Gillespie et al. 11725* (CAN 10116427). Kuugaaluk: site 19.4, *Gillespie et al. 12311* [cf.] (CAN 10116428). Remote Peninsula: site RL-1, *Philpot et al. s.n*. (COLO 01958651 [pro parte, plants B]). Tasialuk S: site 26.5, *Gillespie et al. 11593* (CAN 10116426). **Clyde Area**. Clyde River: site CR-1, *Martin 63* (DAO 01-01000568841); site CR-2, *Forbes 7* (DAO 01-01000568837), *56* (DAO 01-01000568798), *122* (DAO 01-01000568833). Hewett Cache: site HC-1, *Hainault 3810* (QK18299639). Hoffman Cove: site HF-1, *Bartlett 220* (US 03751814). Inugsuin Head: site IG-1, *Hainault 3663* (CAN 10055317, DAO 01-01000568816, O V549235, QK18299637); site IG-5, *Parmelee & Seaborn 3968* (DAO 01-01000568803), *3969* (DAO 01-01001147618); site IG-6, *Parmelee & Seaborn 3855* (DAO 01-01000568831); site IG-7, *Parmelee & Seaborn 3918* (DAO 01-01000568854); site IG-14, *Parmelee & Seaborn 3902* (DAO 01-01000568835); site IG-19, *Hainault 4073* (CAN 10055256, DAO 01-01000568819, O V549141, QK18301709); site IG-26, *Hainault 3679* (CAN 10055305, O V549236, QK18299638), *4031* (CAN 10055300, O V549233, QK18299647). Inugsuin Mouth: site IM-1, *Hainault 3887* (O V549237, QK18299634), *3927* (CAN 10055312, O V549238, QK18299646), *3960* (CAN 10055304, DAO 01-01000568818, O V549234, QK18299545). Kangiqtugaapik Head: site KP-1, *Dansereau 500628-3257* (DAO 01-01000568834), *500628-6091* [pro parte, plants 2] (MT00286023), *500630-1752A* [pro parte, plants 1] (MT00286031), *500709-0556* (MT00286213), *500710-0262B* (MT00286211), *500716-0862A* (MT00286207), *500731-0565* (MT00286210), *500803-0480A* (MT00286208), *Wynne-Edwards 8876* [pro parte] (CAN 10055302), *8900* (CAN 10055303); site KP-21, *Dansereau 500730-0952B* (MT00286209); site KP-24, *Wynne-Edwards 8839* (CAN 10055253); site KP-27, *Wynne-Edwards 9066* (CAN 10055252). McBeth Valley: site MB-1, *Hainault 3748* (DAO 01-01000568817, O V549232, QK18299636), *3751* (QK18299635), *3784* (QK18266933), *3787* (QK18299645). **Coutts Inlet.** Coutts Mouth: site CO-1, *Coombs 80* (DAO 01-01000568805). **Home Bay.** Ekalugad Head: site EK-3, *Philpot et al. s.n.* (COLO 01960426). Kangirlugag Fiord: site KG-1, *Webber 1306* (COLO 01960418).

##### *Draba oblongata* R.Br. ex DC.—Canadian Arctic draba—Circumpolar?

This species is found scattered throughout the Canadian Arctic Archipelago, most commonly in the north and west, but absent from southeastern Baffin Island (Aiken et al. 2007). The single record cited here is the easternmost site for Baffin Island. The species was mapped for the Clyde area by Porsild (1957) and Porsild and Cody (1980), presumably based on the single collection cited here. There are few records from Baffin Island; the closest one to the flora area is *Webber 62* (CAN 10055160) from Isortoq Valley on the northeast side of the Barnes Ice Cap. The species may sometimes be confused with *D. arctica* or *D. micropetala* (Al-Shehbaz et al. 2010); in Greenland *D. oblongata* and *D. arctica* overlap morphologically and are difficult to separate (Elven et al. 2011). More work on this complex is needed in the Canadian Arctic.

**Specimens examined: Clyde Area.** Kangiqtugaapik Head: site KP-1, *Dansereau 500703-0698* (MT00285841).

##### *Draba subcapitata* Simmons—Ellesmere Island draba—Circumpolar

This species is newly recorded for the flora area here and in Gillespie et al. (2024), and this is the first confirmed record for eastern Baffin Island. In Canada it is primarily restricted to the Arctic Islands and is mostly rare and scattered on Baffin Island. Only one other collection is known from central Baffin Island, from the southwest corner (*Manning 75*CAN).

**Specimens examined: Agguttinni TP.** Generator Lake: site GL-18, *Raynolds & Bültmann MKR-2022-41* (CAN 10169284).

#### ﻿*Eutrema* R.Br.

##### *Eutrema edwardsii* R.Br.—Edwards’ mock wallflower—Circumpolar-alpine

This species was first recorded in the flora area from the Clyde area on a map in Porsild (1957), likely based on *Wynne-Edwards 8887* (CAN), which was seen and determined by Porsild; curiously, the species was not mapped in the flora area by Porsild and Cody (1980) or Aiken et al. (2007).

**Specimens examined: Agguttinni TP.** Kangiqtualuk Agguqti: site 8.7, *Gillespie et al. 11979* (ALTA, CAN 10116465, npsp). Kangiqtualuk Uqquqti: site 28.6, *Gillespie et al. 11677* (CAN 10116467); site 29.1, *Gillespie et al. 11710* (CAN 10116468, CHARS, MO, MT). Remote Peninsula: site RL-1, *Philpot et al. s.n.* (COLO 01965441). Tasialuk S: site 21.12, *Gillespie et al. 12442* (ALA, CAN 10116466). **Clyde Area.** Clyde River: site CR-1, *Martin 64* (DAO 01-01000524688, QFA0615056); site CR-2, *Forbes 40* (DAO 01-01000524684), *64* (DAO 01-01000524735), *113* (DAO 01-01000524685). Kangiqtugaapik Head: site KP-1, *Wynne-Edwards 8887* (CAN 10057857); site KP-2, *Martin 60* (DAO 01-01000524687).

#### ﻿*Physaria* (Nutt.) A.Gray

##### *Physaria arctica* (Wormsk. ex Hornem.) O’Kane & Al-Shehbaz (≡*Lesquerella arctica* (Wormsk. ex Hornem.) S. Watson)—Arctic bladderpod—Asian (N)–amphi-Beringian–North American (N)

**Specimens examined: Agguttinni TP.** Kangiqtualuk Agguqti: site 8.2, *Gillespie et al. 11918* (CAN 10116471, MT). Kangiqtualuk Uqquqti: site 29.2, *Gillespie et al. 11691* (CAN 10116469); site 30.8, *Gillespie et al. 11732* (CAN 10116470, npsp). **Clyde Area.** Inugsuin Head: site IG-1, *Hainault 3695* (CAN 10055521, DAO 01-01000558173, DAO 01-01000558173, O V549413, QK18298713). Kangiqtugaapik Head: site KP-1, *Dansereau, 500630-2058* (MT00286118), *500708-0554* (MT00286116), *500803-0475* (MT00286117); site KP-11, *Wynne-Edwards 8886* (CAN 10055462); site KP-17, *Dansereau 500623-1051* (MT00286119), *500623-1855* (MT00286620); site KP-18, *Wynne-Edwards 8806* (CAN 10055541), *8824* (CAN 10055469).

#### ﻿Campanulaceae

***Melanocalyx* Morin**

##### *Melanocalyx uniflora* (L.) Morin (≡*Campanula uniflora* L.)—Arctic bellflower—Amphi-Beringian–North American (N)–amphi-Atlantic

Also recorded from Cape Adair (as *Campanula uniflora*) (Taylor 1863), but no collection located.

**Specimens examined: Agguttinni TP.** Atagulisaktalik: site 23.11, *Gillespie et al. 11536* (CAN 10116472). Clark Fiord: site 20.1, *Gillespie et al. 12340* (CAN 10116475, npsp). Kangiqtualuk Agguqti: site 3.5, *Gillespie et al. 11852* (CAN 10116474). Kangiqtualuk Uqquqti: site 28.3, *Gillespie et al. 11650* (CAN 10116473, MT). Remote Peninsula: site RL-1, *Philpot et al. s.n.* (COLO 01997469), *Philpot et al. s.n.* (COLO 01997501). **Clyde Area.** Clyde River: site CR-1, *Martin 18* (DAO 01-01431612). Hoffman Cove: site HF-1, *Bartlett 236* (US 00935646). Inugsuin Head: site IG-5, *Parmelee & Seaborn 396*6 (DAO 01-01431605); site IG-6, *Parmelee & Seaborn 3856* (DAO 01-01431611); site IG-21, *Hainault 3738* (DAO 01-01431600, O V556173, QK 59615). Inugsuin Mouth: site IM-1, *Hainault 3849* (QK 59659), *3956* (CAN 10082061, DAO 01-01431599, O V556174, QK 59788). Kangiqtugaapik Head: site KP-1, *Dansereau 500726-0763* (MT00285498), *Wynne-Edwards 8974* (CAN 10082076); site KP-8, *Wynne-Edwards 9011* (CAN 10082082); site KP-20, *Wynne-Edwards 8996* (CAN 10082077); site KP-25, *Dansereau 500731-0455* (MT00286868). **Home Bay.** Cape Hooper: site HO-1, *Elven 3482/99* (CAN 10082083, O V556157). Cape Hooper: site HO-1, *Elven 3572/99* (ALA V131954). Ekalugad Head: site EK-1, *Ryder s.n.* (COLO 01997550); site EK-2, *Stock s.n..* (UBC V162137). Kangirlugag Fiord: site KG-1, *Webber 1223* (CAN 10082069, COLO 01997493, QK 71155). Rocknoser Fiord: site RF-1, *Smith VP-68-61* (CAN 10082080).

#### ﻿Caryophyllaceae

***Arenaria* L.**

##### *Arenaria humifusa* Wahlenb.—Creeping sandwort—North American (N)–amphi-Atlantic

Here and in Gillespie et al. (2024) we cite the first records for the flora area and central Baffin Island as defined here. This boreal-low Arctic species is uncommon with a very scattered distribution mostly in the southeastern Canadian Arctic Islands (Polunin 1940; Porsild 1957; Porsild and Cody 1980; Aiken et al. 2007); on northern Baffin Island it is known from two collections (*Bennett 16-0419*CAN; *Polunin 2551*CAN) (Polunin 1940; Aiken et al. 2007). A single locality on Victoria Island is known from the western islands (Gillespie et al. 2015; Saarela et al. 2020b). Polunin (1940) recorded the species on Ellesmere Island (based on a Simmons collection from 1899, not located). Porsild (1957) and Porsild and Cody (1980) also mapped it on Cornwallis Island, likely based on a collection cited in Polunin (1940) (*Simmon 2675* collected in 1899, not located). Two recent iNaturalist observations verified here confirm its presence on Ellesmere Island (https://www.inaturalist.org/observations/182731409; https://www.inaturalist.org/observations/182731404). Note that all dots on mainland western North America in Aiken et al. (2007), Porsild (1957), and Porsild and Cody (1980) belong to the related amphi-Beringian species *A. longipedunculata* Hultén.

**Specimens examined: Agguttinni TP.** Kangiqtualuk Agguqti: site 21.1, *Gillespie et al. 12410* (CAN 10116652, npsp). Kangiqtualuk Uqquqti: site 28.1, *Gillespie et al. 11632* (CAN 10116651).

#### ﻿*Cerastium* L.

*Cerastium* in the Arctic is taxonomically difficult due to extensive hybridization, polyploidy, and reticulate evolution (Brysting and Elven 2000; Brysting et al. 2007).

##### Key to species of *Cerastium* [adapted from Morton (2005a)]

**Table:** 

1	Long flexuous nonglandular hairs present on stems and/or leaf blades, some hairs usually also short and glandular	***C. alpinum***
–	Long flexuous hairs absent, pubescence of mostly straight, usually glandular (on stems), short and/or long hairs	***C. arcticum***

##### Cerastium alpinum L. subsp. alpinum—Alpine chickweed—Amphi-Atlantic

**Specimens examined: Agguttinni TP.** Kangiqtualuk Uqquqti: site 28.1, *Gillespie et al. 11626* (CAN 10116645). Ravenscraig Harbour: site RH-1, *Dare 2* (CAN 10092201), *39* (CAN 10092208). **Clyde Area.** Clyde River: site A22.4, *Gillespie et al. 12465* (CAN 10116644); site CR-2, *Forbes 124* (DAO 01-01667495). Hoffman Cove: site HF-1, *Bartlett 249* (MT00286138, US 03600887). Kangiqtugaapik Head: site KP-1, *Dansereau 500628-3256* (MT00286137), *500630-1762* (MT00286136), *Wynne-Edwards 8857* (CAN 10045808 [pro parte, plants b]); site KP-5, *Wynne-Edwards 9090* (CAN 10018144).

##### *Cerastium arcticum* Lange—Arctic chickweed—North American (N)–amphi-Atlantic–European (N)

Aiken et al. (2007) mapped this species from six localities in the flora area, following confirmation of the species in North America by Brysting and Elven (2000) in their study of the *C. alpinum*-*C. arcticum* complex. Previous Canadian Arctic floras treated collections under *C. beeringianum* or *C. alpinum* (Polunin 1940; Porsild 1957; Porsild and Cody 1980), which accounts for mapping of the former species in the Clyde area. *Cerastium beeringianum*, most common in the western and southern Arctic and on Baffin Island, found only scattered on the southern part, is characterized by smaller flowers and more flowers per inflorescence (Morton 2005a; Saarela et al. 2020b). One collection (*Smith VP-25-61*) from Home Bay was found to be somewhat intermediate morphologically between *C. arcticum* and *C. beeringianum* and is included here under *C. arcticum*.

**Specimens examined: Agguttinni TP.** Atagulisaktalik: site 23.3, *Gillespie et al. 11511* (CAN 10116647); site 23.6, *Gillespie et al. 11520* (CAN 10116642); site 23.9, *Gillespie et al. 11526* (CAN 10116646, QFA). Clark Fiord: site 20.3, *Gillespie et al. 12363* (ALA, CAN 10116635, QFA); site 20.6, *Gillespie et al. 12368* (CAN 10116636, MT). Generator Lake: site GL-20, *Raynolds & Bültmann MKR-2022-54* (CAN 10169278). Gibbs Fiord: site 20.9, *Gillespie et al. 12381* (CAN 10116637, MT); site 20.11, *Gillespie et al. 12390* (ALA, ALTA, CAN 10116648, MO, npsp). Kangiqtualuk Agguqti: site 5.3, *Gillespie et al. 11862* (CAN 10116633, MT); site 8.1, *Gillespie et al. 11900* (CAN 10116638, NY, UBC); site 8.9, *Gillespie et al. 11984* (CAN 10116640, CHARS, MO, QFA). Kangiqtualuk Uqquqti: site 29.4, *Gillespie et al. 11702* (ALA, CAN 10116639, CHARS, MO, US). Kuugaaluk: site 16.4, *Gillespie et al. 12210* (ALA, CAN 10116650, US, WIN). . Refuge Harbour: site 12.5, *Gillespie et al. 12049* (CAN 10116643). Scott Inlet: site SI-1, *Taylor s.n.* (MEL 2490490). Tasialuk N: site 26.1, *Gillespie et al. 11576* (ALTA, CAN 10116641, MT). Tingijattut: site 15.9, *Gillespie et al. 12177* (ALTA, CAN 10116649, MT). **Clyde Area.** Cape Hewett: site CH-1, *Platt 480* (NY 3160982). Clyde River: site 10.1, *Gillespie et al. 11990* (CAN 10116634, npsp); site CR-1, *Dutilly 1459* (CAN 10018333), *1462* (CAN 10045807, DAO 01-01667497), *Oughton s.n*. (TRT00042380), *Polunin 618* (CAN 10045814); site CR-1, *Martin 6* (DAO 525285); site CR-2, *Forbes 12* (DAO 01-01667503), *49* (DAO 01-01667504). Hewett Cache: site HC-1, *Hainault 3809* (QK 59541). Inugsuin Head: site IG-1, *Hainault 3632* (QK 60122), *3662* (CAN 10045832, DAO 01-01667501, QK 59700), *4042* (CAN 10045841, DAO 01-01667500, QK 59734); site IG-6, *Parmelee & Seaborn 3871* (DAO 01-01667496); site IG-8, *Parmelee & Seaborn 3949* (DAO 01-01667502); site IG-19, Hainault 4077 (CAN 10045840, DAO 838842, QK 59818). Inugsuin Mouth: site IM-1, *Hainault 3824* (CAN 10045843, DAO 525297, QK 59677), *3866* (CAN 10045889, DAO 01-01667498, QK 59767), *3930* (CAN 10045839, DAO 834854, QK 59700). Kangiqtugaapik Head: site KP-1, *Wynne-Edwards 8857* (CAN 10045808 [pro parte, plants a]). McBeth Valley: site MB-1, *Hainault 3792* (CAN 10045838, DAO 01-01667499, QK 59641). **Coutts Inlet.** Coutts Mouth: site CO-1, *Coombs 82* (DAO 01-01667643). **Home Bay.** Cape Hooper: site HO-1, *Elven 3442/99* (ALA V132406, CAN 10018170, O V548130), *3444/99* (ALA V132409, CAN 10045902); site HO-3, *Parmelee & Seaborn 3824* (DAO 525297). Ekalugad FOX-C: site FC-1, *Richardson & Webber 7* (CAN 10045837, COLO 01851468, QK 71098); site FC-2, *Oswald 354* (QK 135591). Ekalugad Head: site EK-1, *Ryder s.n.* (COLO 01851393), *Ryder s.n.* (COLO 01851401). Kangirlugag Fiord: site KG-1, *Webber 1222* (COLO 01851427), *1298a* (CAN 10045836, COLO 01851419, QK 71099). Rocknoser Fiord: site RF-1, *Smith VP-25-61* [cf.] (CAN 10046016).

#### ﻿*Cherleria* L.

##### *Cherleria biflora* (L.) A.J.Moore & Dillenb. (≡*Minuartia biflora* (L.) Schinz & Thell.; =*Arenaria sajanensis* Willd. ex D.F.K.Schltdl.)—Mountain stitchwort—Circumpolar-alpine

This species has a very scattered distribution in the Canadian Arctic Islands and is most frequent on southern Baffin Island. Polunin (1940) first recorded the species from the flora area based on a 1936 Dutilly collection from Clyde River and a Taylor collection (labelled *Arenaria arctica*) from Scott’s Bay (Taylor 1863); neither specimen has been located. Aiken et al. (2007) mapped Clyde River and two localities from the Home Bay area, Ekalugad FOX-C and Kangirlugag Fiord, based on the collections cited here.

**Specimens examined: Agguttinni TP.** Kuugaaluk: site 16.7, *Gillespie et al. 12222* (CAN 10116551, MT, npsp). **Clyde Area.** Clyde River: site CR-2, *Forbes 90b* (DAO 588793), *95* (DAO 588796). **Home Bay.** Cape Hooper: site HO-1, *Elven 3443/99* (CAN 10046791), *3483/99* (ALA V132445), *Elven 3519/L1-4* (O V222269). Ekalugad FOX-C: site FC-1, *Richardson & Webber 56* (CAN 10046767). Kangirlugag Fiord: site KG-1, *Webber 1178* (CAN 10046765, COLO 01859651, QK 71105).

#### ﻿*Honckenya* Ehrh.

##### Honckenya peploides (L.) Ehrh. subsp. diffusa (Hornem.) Hultén ex V.V.Petrovsky (≡Arenaria peploides var. diffusa Hornem.) (Fig. 12E)—Northern seabeach sandwort—Circumpolar

**Specimens examined: Agguttinni TP.** Atagulisaktalik: site 23.9, *Gillespie et al. 11525* (CAN 10116505, MO). Kangiqtualuk Agguqti: site 6.3, *Gillespie et al. 11898* (CAN 10116506); site 8.1, *Gillespie et al. 11910* (CAN 10116507). Kangiqtualuk Uqquqti: site 30.5, *Gillespie et al. 11723* (ALTA, CAN 10116512, CHARS, MT). Kuugaaluk: site 16.5, *Gillespie et al. 12216* (ALA, CAN 10116511, npsp, QFA, US); site A22.3, Gillespie et al. 12456 (CAN 10116504). Refuge Harbour: site 12.4, *Gillespie et al. 12047* (CAN 10116509). Tingijattut: site 15.9, *Gillespie et al. 12180* (CAN 10116510, MT). **Clyde Area**. Clyde River: site 10.1, *Gillespie et al. 11991* (CAN 10116508, MT); site CR-1, *Martin 27* (DAO 528143), *Polunin 656* (CAN 10046609); site CR-3, *Dare 53* (CAN 10092184). Inugsuin Head: site IG-2, *Hainault 3637* (CAN 10046607, O V548195, QK 59568); site IG-3, *Parmelee & Seaborn 3913* (DAO 528141). Kangiqtugaapik Head: site KP-1, *Dansereau 500604-0251* (MT00285795), *500710-0468* (MT00285796), *Wynne-Edwards 8927* (CAN 10046617). **Home Bay.** Ekalugad Head: site EK-1, *Crompton et al. s.n.* (COLO 01859768). Rocknoser Fiord: site RF-1, *Smith VP-46-61* (CAN 10046616).

#### ﻿*Sabulina* Rchb.

##### Key to species of *Sabulina* [adapted from Porsild and Cody (1980) and Rabeler et al. (2005)]

**Table:** 

1	Stems and pedicels stipitate-glandular; leaves stiff, 3-nerved (clearly visible in marcescent leaves)	***S. rubella***
–	Stems and pedicels glabrous; leaves 1-nerved or nerves not apparent	***S. stricta***

##### *Sabulina rubella* (Wahlenb.) Dillenb. & Kadereit (≡*Arenaria rubella* (Wahlenb.) Sm.; ≡*Minuartia rubella* (Wahlenb.) Hiern)—Reddish stitchwort—Circumpolar-alpine

**Specimens examined: Agguttinni TP.** Generator Lake: site GL-18, *Raynolds & Bültmann MKR-2022-40* (CAN 10169280). Kangiqtualuk Agguqti: site 6.3, *Gillespie et al. 11885* (CAN 10116670); site 8.4, *Gillespie et al. 11955* (CAN 10116671). Kangiqtualuk Uqquqti: site 29.5, *Gillespie et al. 11708* (CAN 10116678); site 30.4, *Gillespie et al. 11719* (CAN 10116676, npsp); site 30.5, *Gillespie et al. 11727* (CAN 10116679). Tasialuk S: site 21.12, *Gillespie et al. 12444* (CAN 10116672, MT); site 26.5, *Gillespie et al. 11589* (CAN 10116677). **Clyde Area.** Hewett Cache: site HC-1, *Hainault 3811* (QK 59545). Inugsuin Head: site IG-1, *Hainault 3664* (DAO 837438, O V548244, QK 59702), *3681* (QK 59708), *4043* (QK 59535); site IG-6, *Parmelee & Seaborn 3873* (DAO 527876); site IG-9, *Parmelee & Seaborn 3936* (DAO 527875); site IG-24, *Hainault 3646* (QK 59533); site IG-30, *Hainault 3745* (CAN 10044176, DAO 837439, O V548243, QK 59620). Kangiqtugaapik Head: site KP-1, *Dansereau 500701-1058* (MT00285835), *500723-0359* (MT00285834), *500731-0567* (MT00286978), *Wynne-Edwards 8921* (CAN 10044182), *8944* (CAN 10044180), *8972* (CAN 10044181); site KP-7, *Dansereau 500709-0663* (MT00285833); site KP-10, *Wynne-Edwards 8981* (CAN 10044203). McBeth Valley: site MB-1, *Hainault 3764* (QK 59553).

##### *Sabulina stricta* (Sw.) Rchb. (≡*Minuartia stricta* (Sw.) Hiern; =*Arenaria uliginosa* Schleich. ex DC.)—Bog stitchwort—Circumpolar-alpine

Collections from the flora area are among the northernmost records in Canada. Porsild (1957) first recorded the species in the flora area from Kangiqtugaapik Head; he also mapped two localities on northern Baffin Island (collections not located). Since then, several collections have been made on west central Baffin Island (e.g., *Beschel & Webber 634a*CAN) and one on Axel Heiberg Island (*Kuk 77*CAN).

**Specimens examined: Agguttinni TP.** Kangiqtualuk Agguqti: site 21.2, *Gillespie et al. 12419a* (CAN 10116680). Kangiqtualuk Uqquqti: site 28.6, *Gillespie et al. 11676* (CAN 10116673); site 29.3, *Gillespie et al. 11699* (CAN 10116674, npsp); site 29.5, *Gillespie et al. 11707* (CAN 10116675). **Clyde Area**. Kangiqtugaapik Head: site KP-1, *Dansereau 500805-0482* (MT00286980), *Wynne-Edwards 8956* (CAN 10044624), *8979* (CAN 10044647); site KP-5, *Wynne-Edwards 9029* (CAN 10044644).

#### ﻿*Sagina* L.

##### Key to species of *Sagina* [adapted from Crow (1978) and Saarela et al. (2020b)]

**Table:** 

1	Flowers (4) 5-merous; petals 2.5–3 mm long, ≥ sepal length; primary basal rosette of leaves absent, secondary rosettes of linear leaves often present; pedicels usually glandular-pubescent, rarely glabrous	***S. caespitosa***
–	Flowers 4 (5)-merous; petals 1.5–2 mm long, ≤ sepal length; primary basal rosette of fleshy, subulate leaves present, secondary rosettes absent; pedicels glabrous	***S. nivalis***

##### *Sagina caespitosa* (J.Vahl) Lange—Lange Tufted pearlwort—Amphi-Atlantic

In the Canadian Arctic Islands, this species is known from central and southern Baffin Island and one locality each on Southampton and Victoria islands (Aiken et al. 2007; Saarela et al. 2020b). The single collection (*Gillespie 6568*US) mapped on Ellesmere Island by Aiken et al. (2007) was a misidentified specimen of *Sabulina rossii*. The Clark Fiord record cited here is the northernmost record for Canada.

**Specimens examined: Agguttinni TP.** Clark Fiord: site 20.7, *Gillespie et al. 12371* (CAN 10121704, npsp). **Clyde Area.** Hewett Cache: site HC-1, *Hainault 3812* (QK 59547). Inugsuin Mouth: site IM-1, *Hainault 3836* (CAN 10046904, QK 59687). **Home Bay.** Cape Hooper: site HO-1, *Elven 3508/99* (ALA V132304, CAN 10083240).

##### *Sagina nivalis* (Lindblom) Fr. (=*Sagina intermedia* Fenzl)—Snow pearlwort—Circumpolar

**Specimens examined: Agguttinni TP.** Atagulisaktalik: site 22.3, *Gillespie et al. 11494* (CAN 10121705). Generator Lake: site GL-16, *Raynolds & Bültmann MKR-2022-30* (CAN 10169276). Kangiqtualuk Agguqti: site 21.2, *Gillespie et al. 12419b* (CAN 10121710); site 6.3, *Gillespie et al. 11884* (CAN 10121707). Kangiqtualuk Uqquqti: site 30.5, *Gillespie et al. 11726* (CAN 10121706, npsp). Kuugaaluk: site 16.1, *Gillespie et al. 12198* (ALA, CAN 10121709, CHARS). Refuge Harbour: site 12.4, *Gillespie et al. 12046* (CAN 10114756). Tingijattut: site 15.9, *Gillespie et al. 12178* (CAN 10121708, MT). **Clyde Area.** Clyde River: site CR-1, *Martin 23* (DAO 01-01667483), *Polunin 595* (CAN 10083259); site CR-2, *Forbes 90a* (DAO 588792). Inugsuin Head: site IG-1, *Hainault 3634* (CAN 10083247, QK 59599), *3726* (CAN 10083261, DAO 01-01667481, O V548317, QK 59610). Inugsuin Mouth: site IM-1, *Hainault 3826* (DAO 01-01667482, QK 59678), *3874* (DAO 01-01667480, O V548314, QK 59773). **Home Bay.** Cape Hooper: site HO-1, *Elven 3438/99* (CAN 10046883, O V548318), *3507/99* (O V548319), *3523/99* (ALA V132305). Ekalugad FOX-C: site FC-1, *Richardson & Webber 30* (CAN 10083254, COLO 01862234). Kangirlugag Fiord: site KG-1, *Webber 1286* (COLO 01862259), *1302* (CAN 10083257, COLO 01862267).

#### ﻿*Silene* L.

##### Key to species and subspecies of *Silene* [adapted from Morton (2005b), Elven et al. (2011), and Saarela et al. (2020b)]

**Table:** 

1	Plants cushion-forming, 2–5 cm high, petals bright pink, rarely white, styles 3	***S. acaulis***
–	Plants tufted, 4–30 cm high; petals white to pink or purple; styles 5	**2**
2	Calyces inflated; petals dull pink to purple; flowers nodding (*S. uralensis*)	**3**
–	Calyces not inflated; petals white, pink, or purple-tinged; flowers erect	**4**
3	Calyces strongly inflated, in fruit becoming globose or broader than long; petals much emerging from calyx	**S. uralensis subsp. arctica**
–	Calyces weakly inflated, in fruit usually longer than broad; petals slightly emerging from the calyx	**S. uralensis subsp. uralensis**
4	Calyces densely glandular-hairy throughout, hairs often obscuring veins; styles and stamens longer than calyx, often equaling petals; seeds not winged; inflorescence (1–) 3-flowered, usually compact; pedicels usually shorter than calyx, sometimes absent or up to 2x as long	***S. sorensenis***
–	Calyces usually sparsely hairy, with distinct, dark purple, usually hairy veins, pale cream, and often glabrous between; styles and stamens shorter than and included in calyx; seeds winged; inflorescence 1–3-flowered, open; pedicels longer than calyx (*S. involucrata*)	**5**
5	Calyces 10–20 mm in fruit; flowering stems sturdy, usually < 20 cm tall	**S. involucrata subsp. involucrata**
–	Calyces 8–10(–12) mm in fruit; flowering stems slender, usually > 20 cm tall	**S. involucrata subsp. tenella**

##### *Silene acaulis* (L.) Jacq.—Moss campion—Amphi-Beringian–North American–amphi-Atlantic–European–Asian (NW)

**Specimens examined: Agguttinni TP.** Atagulisaktalik: site 23.5, *Gillespie et al. 11514* (CAN 10121728, CHARS, MT). Gibbs Fiord: site 20.11, *Gillespie et al. 12393* (CAN 10121730). Kangiqtualuk Agguqti: site 3.3, *Gillespie et al. 11836* (CAN 10121733, npsp); site 8.1, *Gillespie et al. 11908* (CAN 10121734). Kangiqtualuk Uqquqti: site 27.1, *Gillespie et al. 11608* (CAN 10121729). Kuugaaluk: site 16.1, *Gillespie et al. 12199* (CAN 10121732). Ravenscraig Harbour: site RH-1, *Dare 26* (CAN 10092891). Refuge Harbour: site 11.2, *Gillespie et al. 12024* (CAN 10121735). Tingijattut: site 15.2, *Gillespie et al. 12130* (CAN 10121731). **Clyde Area.** Cape Hewett: site CH-1, *Platt 482a* (NY 3308579). Clyde River: site CR-1, *Martin 15* (DAO 01-01667551); site CR-2, *Forbes 35* (DAO 01-01667554), *120* (DAO 01-01667484). Hoffman Cove: site HF-1, *Bartlett 247* (US 03630037). Inugsuin Head: site IG-2, *Hainault 3659* (DAO 01-01667552, QK 59718); site IG-6, *Parmelee & Seaborn 3870* (DAO 01-01667553). Kangiqtugaapik Head: site KP-1, *Dansereau 500604-0254* (MT00285794), *500619-451* [pro parte, plants 2] (MT00282884), *Wynne-Edwards 8923* (CAN 10044822). **Coutts Inlet.** Coutts Fiord: site CF-1, *Coombs 63* (DAO 01-01667644). Coutts Mouth: site CO-1, *Coombs 93* (DAO 01-01667646). **Home Bay.** Cape Hooper: site HO-1, *Elven 3458/99* (CAN 10044786); site HO-3, *Parmelee & Seaborn 3821* (DAO 530652). Ekalugad FOX-C: site FC-1, *Crompton et al. s.n.* (COLO 02017424), *Richardson & Webber 3* (COLO 02017408, QK 71104). Ekalugad Head: site EK-3, *Crompton et al. s.n.* (COLO 02017416). Kangirlugag Fiord: site KG-1, *Webber 1229e* (COLO 02017390). Rocknoser Fiord: site RF-1, *Smith VP-21-61* (CAN 10044799).

##### Silene involucrata (Cham. & Schltdl.) Bocquet subsp. involucrata (=*Melandrium affine* (J.Vahl ex Fr.) J.Vahl)—Arctic catchfly—Circumpolar

We follow the taxonomy of Morton (2005b) rather than Elven et al. (2011) but note that more taxonomic work is needed.

**Specimens examined: Agguttinni TP.** Atagulisaktalik: site 23.6, *Gillespie et al. 11519* (CAN 10116484); site 23.11, *Gillespie et al. 11539* (CAN 10116485). Clark Fiord: site 20.1, *Gillespie et al. 12325* (CAN 10116494, MT). Gibbs esker: site 20.12, *Gillespie et al. 12395* (CAN 10116486). Kangiqtualuk Agguqti: site 3.3, *Gillespie et al. 11839* (ALTA, CAN 10116495, MT); site 8.1, *Gillespie et al. 11901* (CAN 10116490). Kangiqtualuk Uqquqti: site 29.4, *Gillespie et al. 11703* (CAN 10116493, CHARS). Refuge Harbour: site 14.4, *Gillespie et al. 12090* (CAN 10116492, npsp). Remote Peninsula: site RL-1, *Philpot et al. s.n.* (COLO 02011823). Stewart Valley: site 15.1, *Gillespie et al. 12119* (CAN 10116488). Tingijattut: site 15.3, *Gillespie et al. 12148* (CAN 10116489); site 15.4, *Gillespie et al. 12159* (CAN 10116487). Tingijattut: site TG-2, *Dare et al. 83* (CAN 10092190). **Clyde Area.** Clyde River: site 10.4, *Gillespie et al. 11999* (CAN 10116491). Inugsuin Head: site IG-1, *Hainault 4072* (CAN 10047209, QK 59348); site IG-6, *Parmelee & Seaborn 3838* (DAO 01-01667488); site IG-14, *Parmelee & Seaborn 3897* (DAO 01-01667485); site IG-15, *Parmelee & Seaborn 3880* (DAO 01-01667486); site IG-24, *Hainault 3641* (DAO 01-01667489, MT00286216, O V548344, QK 59572); site IG-26, *Hainault 4028* (CAN 10045026, DAO 01-01667487, QK 59832). Inugsuin Mouth: site IM-1, *Hainault 3929* (CAN 10047170, DAO 839229, O V548346, QK 59777). Kangiqtugaapik Head: site KP-1, *Dansereau 500628-3259* (MT00285826), *500628-6090* (MT00285823), *500630-1760* (MT00285825), *500702-1498* (MT00285824), *500702-1499* (MT00285822), *500708-0553* (MT00286971), *500709-0455* (MT00286975), *500709-0559* (MT00286973), *500716-0861* (MT00286968), *500724-0598* (MT00286760), *500731-0568* (MT00286969); site KP-3, *Dansereau 500709-0591* (MT00286972); site KP-17, *Wynne-Edwards 8893* (CAN 10045066); site KP-22, *Dansereau 500710-3878* (MT00286974); site KP-24, *Wynne-Edwards 8829* (CAN 10045067); site KP-25, *Dansereau 500731-0456* (MT00286970). McBeth Valley: site MB-1, *Hainault 3757* [pro parte] (DAO 839228). **Home Bay.** Ekalugad Head: site EK-1, *Ryder s.n.* (COLO 02011815).

##### *Silene sorensenis* (B.Boivin) Bocquet (=*Melandrium triflorum* (R.Br. ex Sommerf.) J.Vahl)—Sorensen’s catchfly—Amphi-Beringian–North American (N)

**Specimens examined: Agguttinni TP.** Kuugaaluk: site 19.3, *Gillespie et al. 12296* (CAN 10116477, CHARS, MT, npsp); site 19.4, *Gillespie et al. 12308* (ALA, CAN 10116476). Tasialuk S: site 26.6, *Gillespie et al. 11598* (CAN 10116478). **Clyde Area.** Clyde River: site CR-4, *Dansereau 500730-1193* (MT00286203, MT00286204); site CR-5, *Martin 62* (RBCM V135002). Hewett Cache: site HC-1, *Hainault 3808* (MT00286205, O V548392, QK 59561). Inugsuin Head: site IG-1, *Hainault 3728* (CAN 10047217, O V548393, QK 59612); site IG-2, *Hainault 3654* (CAN 10047239, O V548394, QK 59581). Inugsuin Mouth: site IM-1, *Hainault 3817* (CAN 10047248, O V548390, QK 60106), *3885* (CAN 10047245, O V548391, QK 60106). Kangiqtugaapik Head: site KP-1, *Wynne-Edwards 8943* (CAN 10047237); site KP-22, *Dansereau 500710-3754* (MT00286977).

##### Silene uralensis (Rupr.) Bocquet subsp. arctica (Fr.) Bocquet (≡Melandrium apetalum subsp. arcticum (Th.Fr.) Hultén)—Nodding catchfly—Circumpolar

We follow Elven et al. (2011), not Morton (2005b), and recognize two subspecies of S. uralensis in the Canadian Arctic. Subspecies arctica may be distinguished from the more southerly subsp. uralensis by its more strongly inflated calyces that become globose or broader than long in fruit and by its petals much emerging from the calyx (versus fruiting calyces longer than broad and petals only slightly emerging from the calyx) (Elven et al. 2011; Saarela et al. 2020b).

**Specimens examined: Agguttinni TP.** Atagulisaktalik: site 23.12, *Gillespie et al. 11540* (CAN 10116479, npsp). Generator Lake: site GL-20, *Raynolds & Bültmann MKR-2022-58* (CAN 10169275). Kangiqtualuk Agguqti: site 21.1, *Gillespie et al. 12409* (CAN 10116480, MT); site 5.4, *Gillespie et al. 11869* (CAN 10116481). Kangiqtualuk Uqquqti: site 28.1, *Gillespie et al. 11635* (CAN 10116483). Tingijattut: site 15.10, *Gillespie et al. 12187* (CAN 10116482). **Clyde Area.** Clyde River: site CR-1, *Martin 12* (DAO 01-01667490), *Oughton s.n.* (TRT00042394), *Polunin 639* (CAN 10047515), *Sanson 67* (TRT00042395), site CR-2, *Forbes 32* (DAO 573326), *112* (DAO 01-01667491). Hoffman Cove: site HF-1, *Bartlett 250* (US 03632250). Inugsuin Head: site IG-1, *Hainault 3691* (QK 59532); site IG-9, *Parmelee & Seaborn 3948* (DAO 01-01667493); site IG-14, *Parmelee & Seaborn 3896* (DAO 01-01667492). Inugsuin Mouth: site IM-1, *Hainault 3870* (QK 59544). Kangiqtugaapik Head: site KP-1, *Dansereau 500709-0866* (MT00286976), *Wynne-Edwards 8898* (CAN 10047374), *8931* (CAN 10047375); McBeth Valley: site MB-1, *Hainault 3757* [pro parte] (QK 59523). **Home Bay.** Cape Hooper: site HO-1, *Elven 3440/99* (ALA V132313, CAN 10047194, O V548428); site HO-3, *Parmelee & Seaborn 3803* (DAO 531726). Kangirlugag Fiord: site KG-1, *Webber 1184* (CAN 10047571). Rocknoser Fiord: site RF-1, *Smith VP-107-61* (CAN 10047370).

#### ﻿*Stellaria* L.

##### Key to species of *Stellaria* [adapted from Porsild and Cody (1980)]

**Table:** 

1	Leaf blades succulent, not prominently keeled; seashore plants	***S. humifusa***
–	Leaf blades firm, prominently keeled; plants of moist to wet tundra	***S. longipes***

##### *Stellaria humifusa* Rottb.—Saltmarsh starwort—Circumpolar–amphi-Pacific (N)

Also recorded from Cape Adair (Taylor 1863), but no collection located.

**Specimens examined: Agguttinni TP.** Kangiqtualuk Uqquqti: site 30.5, *Gillespie et al. 11721* (CAN 10116629). Kuugaaluk: site 16.5, *Gillespie et al. 12218* (CAN 10116630, CHARS). Niaqurnaaluk-Qassialuit: site 18.1, *Gillespie et al. 12231* (CAN 10116628, npsp). Scott Inlet: site SI-1, *Taylor s.n.* (MEL 2514764). **Clyde Area.** Clyde River: site CR-1, *Martin 24* (DAO 01-01667544), *35* (DAO 0101667543), *Polunin 622* (CAN 10047608); site CR-2, *Forbes 44* (DAO 01-01667540), *144* (DAO 01-01667542); site CR-3, *Dare 51* (CAN 10092219). Inugsuin Head: site IG-3, *Parmelee & Seaborn 4006* (DAO 01-01667541). Kangiqtugaapik Head: site KP-1, *Wynne-Edwards 8915* (CAN 10045469). Inugsuin Mouth: site IM-1, *Hainault 3827* (QK 59679). **Home Bay.** Ekalugad FOX-C: site FC-1, *Richardson & Webber 43* (COLO 01866128), *57* (CAN 10045456, COLO 01865922, QK 71103). Ekalugad Head: site EK-4, *Philpot et al. s.n*. (COLO 01865948). Rocknoser Fiord: site RF-1, *Smith VP-73-61* (CAN 10045475), *Webber 1191b* (CAN 10045455, COLO 01866136).

##### Stellaria longipes Goldie subsp. longipes (=*Stellaria crassipes* Hultén; =*S. edwardsii* R.Br.; =*S. laeta* Richardson; =*S. monantha* Hultén; =*S. stricta* Richardson; =*S. subvestita* Greene) (Fig. 10F)—Long-stalked starwort—Circumboreal-polar

Also recorded from Scott’s Bay (as *S. stricta*) (Taylor 1863), but no collection located, and from Icy Arm, Quernbiter Fiord (https://www.inaturalist.org/observations/20818437) and Rannoch Arm, Cambridge Fiord (https://www.inaturalist.org/observations/20079715) in the Buchan Gulf area. Porsild (1957), Hainault (1966), and Porsild and Cody (1980) treated the *S. longipes* complex as multiple species, mapping/recording 1, 4, and 4 species, respectively, in the flora area.

**Specimens examined: Agguttinni TP.** Atagulisaktalik: site 23.2, *Gillespie et al. 11507* (CAN 10121736, MO, MT). Clark Fiord: site 20.1, *Gillespie et al. 1*2348 (ALA, CAN 10121746). Generator Lake: site GL-21, *Raynolds & Bültmann MKR-2022-43* (CAN 10169253). Kangiqtualuk Agguqti: site 6.2, *Gillespie et al. 11877* (ALA, CAN 10121737, CHARS, QFA, UBC, US); site 8.2, *Gillespie et al. 11942* (CAN 10121739). Kangiqtualuk Uqquqti: site 29.4, *Gillespie et al. 11700* (CAN 10121738), *11701* (CAN 10121743, MT, npsp). Kuugaaluk: site 19.3, *Gillespie et al. 12300* (CAN 10121744, MT); site 19.4, *Gillespie et al. 12310* (ALTA, CAN 10121745). Ravenscraig Harbour: site RH-1, *Dare 1* (CAN 10092211), *8* (CAN 10092212). Refuge Harbour: site 12.5, *Gillespie et al. 12050* (CAN 10121740). Stewart Valley: site 15.1, *Gillespie et al. 12120* (CAN 10121741). Tasialuk S: site 21.12, *Gillespie et al. 12440* (CAN 10121747). **Clyde Area**. Clyde River: site CR-1, *Dansereau 500723-0358* (MT00285830), *500730-1195* (MT00285829), *Dutilly 1441* (CM414754, DAO 01-01667531, MT00286606), *Polunin 640* (CAN 10049929); site CR-2, *Forbes 5* (DAO 01-01667537), *21* (DAO 01-01667536), *55* (DAO 01-01667549), *134* (DAO 01-01667548). Hewett Cache: site HC-1, *Hainault 3803* (CAN 10047628, O V548491, QK 60108), *3806* (CAN 10047627, O V548490, QK 59560). Hoffman Cove: site HF-1, *Bartlett 243* (US 03608584). Inugsuin Head: site IG-1, *Hainault 3660* (QK 59698) site IG-2, *Hainault 3656* (DAO 01-01667550, QK 59695), *3686* (CAN 10049694, QK 59710), *3687* (MT00286217, QK 59711); site IG-3, *Parmelee & Seaborn 4007* (DAO 01-01667538), *4008a* (DAO 01-01667539); site IG-19, *Hainault 4066* (CAN 10047629, DAO 01-01667530, O V548487, QK 59842); site IG-24, *Hainault 3725* (DAO 01-01667547, O V548524, QK 59609); site IG-25, *Hainault 4013* (QK 59721); site IG-26, *Hainault 4029* (CAN 10047800, O V548494, QK 59833); site IG-30, *Hainault 4050* (CAN 10047799, DAO 01-01667532, QK 59736); site IM-1, *Hainault 3917* (O V548489), *3819* (MT00286215, O V548493, QK 59672), *3846* (CAN 10047797, DAO 01-01667535, O V548496, QK 59656), *3865A* (CAN 10049697, O V548497, QK 59765), *3831* (QK 59683), *3834* (QK 59685), *3865B* (CAN 10049696, QK 59766), *3889* (CAN 10049695, DAO 01-01667545, QK 60107), *3914* (CAN 10047630, QK 59813). Inugsuin Mouth: site IM-1, *Hainault 3928* (CAN 10049388, DAO 01-01667529, QK 59816), *3959* (CAN 10047798, DAO 01-01667533, O V548495, QK 59555), *3962* (CAN 10047796, QK 59835). Kangiqtugaapik Head: site KP-1, Dansereau *500628-3258* (MT00285831), *500703-0367* (MT00285827), *500710-1051* (MT00285828), *Wynne-Edwards 8947* (CAN 10049946); site KP-22, *Dansereau 500710-3877* (MT00285832). McBeth Valley: site MB-1, *Hainault 3755a* (DAO 01-01667534, MT00286218, QK 59591), *3755b* (DAO 01-01667546, QK 59525), *3755c* (QK 59524), *3755d* (QK 59592), *3786* (QK 59625). **Coutts Inlet.** Coutts Fiord: site CF-1, *Coombs 65* (DAO 524748). Coutts Mouth: site CO-1, *Coombs 94* (DAO 01-01667649). **Home Bay.** Cape Hooper: site HO-1, *Elven 3481/99* (O V548518). Ekalugad FOX-C: site FC-1, *Richardson & Webber 25* (CAN 10047780, COLO 01865534, QK 71101). Ekalugad Head: site EK-1, *Ryder s.n.* (UBC V162134). Kangirlugag Fiord: site KG-1, *Webber 1282* (CAN 10047755, COLO 01865518, MT00286219, QK 71102), *1297* (CAN 10047756, COLO 01865526, QK 71100).

#### ﻿Diapensiaceae

***Diapensia* L.**

##### *Diapensia lapponica* L. (Fig. 12G)—Lapland diapensia—North American (NE)–amphi-Atlantic–European (N)–Asian (NW)

Our Agguttinni TP collections are the northernmost records of this low Arctic and subarctic species in Canada (Gillespie et al. 2024). Elsewhere in the Canadian Arctic Islands, *D. lapponica* is found in west-central and southern Baffin, Southampton, and Coats islands (Aiken et al. 2007; Saarela et al. 2020a; Saarela et al. 2023).

**Specimens examined: Agguttinni TP.** Kangiqtualuk Agguqti: site 3.1, *Gillespie et al. 11794* (CAN 10114924, MT); site 6.1, *Gillespie et al. 11873* (ALA, CAN 10116601, CHARS, npsp). **Clyde Area.** Inugsuin Head: site IG-7, *Parmelee & Seaborn 3928* (DAO 01-01522818); site IG-24, *Hainault 3710* (CAN 10077051, O V555753, K 59637). Inugsuin Mouth: site IM-1, *Hainault 3952* (CAN 10077052, O V555746, QK 59784). Kangiqtugaapik Head: site KP-1, *Dansereau, 500619-451* [pro parte, plants 1] (MT00204131), *500629-2298* (MT00204090), *5007*… (MT00204130), *500706-2299* (MT00204091), *500731-0172* (MT00204089); site KP-5, *Wynne-Edwards 8869* (CAN 10077064); site KP-27, *Wynne-Edwards 8817* (CAN 10077065). McBeth Valley: site MB-1, *Hainault 3793* (QK 59642). **Home Bay.** Ekalugad Head: site EK-3, *Crompton et al. s.n.* (COLO 02049203), Kangirlugag Fiord: site KG-1, *Webber 1251* (CAN 10077066, COLO 02049229, QK 71152), *1187* (COLO 02049237, QK 71153). Rocknoser Fiord: site RF-1, *Smith VP-29-61* (CAN 10077078), *VP-30-61* (CAN 10077049).

#### ﻿Ericaceae

***Arctous* (A.Gray) Nied.**

##### *Arctous alpina* (L.) Nied. (≡*Arctostaphylos alpina* (L.) Spreng.) (Fig. 9D)—Alpine bearberry—Circumpolar-alpine

Aiken et al. (2007) and Porsild and Cody (1980) first mapped the species from the flora area based on the single collection cited here. This is the northernmost record for the species in eastern Canada.

**Specimens examined: Clyde Area.** McBeth Valley: site MB-1, *Hainault 3795* (CAN 10075863, O V555516, QK 59643).

#### ﻿*Cassiope* D.Don

##### Cassiope tetragona (L.) D.Don subsp. tetragona—Four-angled mountain heather—Circumpolar-alpine

Also recorded from Feachem Bay (https://www.inaturalist.org/observations/242269896; https://www.inaturalist.org/observations/37255394) and Icy Arm, Quernbiter Fiord (https://www.inaturalist.org/observations/20818268) in the Buchan Gulf area.

**Specimens examined: Agguttinni TP.** Atagulisaktalik: site 22.1, *Gillespie et al. 11464* (ALA, CAN 10116718, CHARS, MT, npsp). Generator Lake: site GL-13, *Raynolds & Bültmann MKR-2022-24* (CAN 10169268). Kangiqtualuk Agguqti: site 3.1, *Gillespie et al. 11795* (CAN 10116720); site 8.2, *Gillespie et al. 11943* (CAN 10116721). Kangiqtualuk Uqquqti: site 28.1, *Gillespie et al. 11631* (ALTA, CAN 10116719, MO). Kuugaaluk: site 16.8, *Gillespie et al. 12223* (CAN 10116727); site 19.1, *Gillespie et al. 12289* (CAN 10116723, US). Ravenscraig Harbour: site RH-1, *Dare 12* (CAN 10092600), *34* (CAN 10092599). Refuge Harbour: site 11.1, *Gillespie et al. 12009* (CAN 10116722, QFA). Stewart Valley: site 15.1, *Gillespie et al. 12108* (CAN 10116725). Tingijattut: site 15.2, *Gillespie et al. 12134* (CAN 10116726). **Clyde Area.** Clyde River: site A22.4, *Gillespie et al. 12466* (CAN 10116724); site CR-1, *Dutilly 1485* (MT00286609), *9362* (CM520473), *Martin 11* (DAO 000001101), *McMillan 77, 313* (potentially old herbarium number) (CAN 10074681); site CR-2, *Forbes 22* (DAO 000000996), *115* (DAO 000000981). Hoffman Cove: site HF-1, *Bartlett 229* (US 02982154). Inugsuin Head: site IG-1, Hainault 3635 (DAO 000000973, O V555543, QK 59600); site IG-6, *Parmelee & Seaborn 3858* (DAO 000001107); site KP-1, *Dansereau 500604-0667* (MT00285501, MT00286876), *500624-0258* (MT00285500, MT00286874, MT00286875), *500630-1857* (MT00286640), *500630-1957* (DAO 000001099, DAO 000001118, MT00285503), *500731-0360* (MT00286865, MT00286866), *500731-0671* (MT00285502, MT00286877), *500827-1058* (MT00285504, MT00286878), *Wynne-Edwards 8873* (CAN 10074602). **Coutts Inlet.** Coutts Fiord: site CF-1, *Coombs 47* (DAO 000001119). Coutts Mouth: site CO-1, *Coombs 70* (DAO 000000976). **Home Bay.** Cape Hooper: site HO-1, *Elven 3439/99* (ALA V132022, CAN 10074687, O V555537); site HO-3, *Parmelee & Seaborn 3787a* (DAO 000000980). Ekalugad FOX-C: site FC-1, *Crompton et al. s.n.* (COLO 02074631), *Crompton et al. s.n.* (COLO 02074649), *Richardson & Webber 16* (CAN 10074675, COLO 02074656); site FC-2, *Oswald 388* (QK 135610). Kangirlugag Fiord: site KG-1, *Webber 1237* (CAN 10074676, COLO 02074664). Kangok Fiord: site KO-1, *Crompton et al. s.n.* (COLO 02074672). Rocknoser Fiord: site RF-1, *Smith VP-6-61* (CAN 10074601).

#### ﻿*Empetrum* L.

##### *Empetrum nigrum* L.—Black crowberry—Circumboreal-polar

**Specimens examined: Agguttinni TP.** Atagulisaktalik: site 23.7, *Gillespie et al. 11522* (ALTA, CAN 10116552, CHARS, MT, npsp). Kangiqtualuk Agguqti: site 3.3, *Gillespie et al. 11838* (CAN 10116553); site 8.8, *Gillespie et al. 11983* (CAN 10116554, MT). Kangiqtualuk Uqquqti: site 28.3, *Gillespie et al. 11653* (ALA, CAN 10116560, US). Kuugaaluk: site 16.8, *Gillespie et al. 12224* (CAN 10116559, npsp). Refuge Harbour: site 12.3, *Gillespie et al. 12041* (CAN 10116555, QFA). Stewart Valley: site 15.1, *Gillespie et al. 12126* (CAN 10116557, MO). Tingijattut: site 15.7, *Gillespie et al. 12173* (CAN 10116558, NY); site TG-2, *Dare et al. 85* (CAN 10092395). **Clyde Area.** Clyde River: site CR-1, *Dutilly 9363* (DAO 01-01275391), *Martin 25* (DAO 01-01275390); site CR-2, *Forbes 119* (DAO 01-01275491). Inugsuin Head: site IG-1, *Hainault 3720* (DAO 01-01275399, O V555720, QK 59604); site IG-3, *Parmelee & Seaborn 4000a* (DAO 01-01275396); site IG-6, *Parmelee & Seaborn 3828* (DAO 01-01275389). Kangiqtugaapik Head: site KP-1, *Dansereau 500604-0666* (MT00286089), *500624-0360* (MT00286093, MT00286095), *500827-1459* (MT00286633), *Wynne-Edwards 8872* (CAN 10072674); site KP-16, *Wynne-Edwards 8935* (CAN 10072547). McBeth Valley: site MB-1, *Hainault 3783* (DAO 01-01275400, O V555719, QK 59628). **Home Bay.** Cape Hooper: site HO-3, *Parmelee & Seaborn 3820* (DAO 01-01276378). Kangirlugag Fiord: site KG-1, *Webber 1204* (COLO 02066454, QK 71147). Rocknoser Fiord: site RF-1, *Webber 1200* (CAN 10072705, COLO 02066488).

#### ﻿*Harrimanella* Coville

##### *Harrimanella hypnoides* (L.) Coville (≡*Cassiope hypnoides* (L.) D.Don)—Moss heather—North American (NE)–amphi-Atlantic–European (N)–Asian (NW)

First mapped in Aiken et al. (2007), the two collections cited here from the Home Bay area are the northernmost verified records for Canada. Also recorded from Scott’s Bay (Taylor 1863), but no collection was located; Polunin (1940) cited the Taylor reference but apparently saw no specimens. Elsewhere in the Canadian Arctic Islands, it is found only on southern and southeastern Baffin Island and Coats and Mansel islands to the south (Aiken et al. 2007; Saarela et al. 2020a).

**Specimens examined: Home Bay.** Cape Hooper: site HO-1, *Elven 3502/99* (CAN 10074703, O V555580). Kangirlugag Fiord: site KG-1, *Webber 1317* (CAN 10074707, QK 71151).

#### ﻿*Pyrola* L.

##### *Pyrola grandiflora* Radius (Figs 10H, 13C)—Arctic pyrola—Circumpolar

Also recorded from Feachem Bay (https://www.inaturalist.org/observations/248850423; https://www.inaturalist.org/observations/20333233; https://www.inaturalist.org/observations/178358964), Icy Arm, Quernbiter Fiord (https://www.inaturalist.org/observations/20818246), and Rannoch Arm, Cambridge Fiord (https://www.inaturalist.org/observations/20079565) in the Buchan Gulf area.

**Specimens examined: Agguttinni TP.** Arviqtujuq Kangiqtua NW: site 18.4, *Gillespie et al. 12276* (ALA, CAN 10116654, CHARS). Atagulisaktalik: site 22.2, *Gillespie et al. 11492* (CAN 10116657); site AT-1, *Dansereau 500723-0772* (MT00285790). Clark Fiord: site 20.1, *Gillespie et al. 12343* (ALTA, CAN 10116653). Gibbs Fiord: site 20.10, *Gillespie et al. 12386* (CAN 10116655, QFA). Kangiqtualuk Agguqti: site 3.2, *Gillespie et al. 11829* (CAN 10116658); site 8.2, *Gillespie et al. 11946* (CAN 10116656). Kangiqtualuk Uqquqti: site 27.1, *Gillespie et al. 11611* (CAN 10116662, MT). Refuge Harbour: site 12.2, *Gillespie et al. 12038* (CAN 10116660). Remote Peninsula: site RL-1, *Philpot et al. s.n.* (COLO 02378156). Stewart Valley: site 15.1, *Gillespie et al. 12124* (CAN 10116661, npsp). Tingijattut: site TG-2, *Dare et al. 80* (CAN 10092613). **Clyde Area.** Clyde River: site 10.3, *Gillespie et al. 11997* (CAN 10116659); site CR-1, *Martin 17* (DAO 01-01476918). Hoffman Cove: site HF-1, *Bartlett 228* (CAN 10074064, US 03104019). Inugsuin Head: site IG-1, *Crompton et al. s.n.* (COLO 02378131), *Hainault 3997* (CAN 10074057, DAO 01-01476907, O V555482); site IG-6, *Parmelee & Seaborn 3857* (DAO 01-01476925). Inugsuin Mouth: site IM-1, *Hainault 3943* (DAO 01-01476908, O V555481). Kangiqtugaapik Head: site KP-1, Dansereau 500629-2182 (MT00285793), *500712-3953* (MT00285792), *500731-0357* (MT00285791). Kangiqtugaapik Head: site KP-1, *Wynne-Edwards 8959* (CAN 10074077). McBeth Valley: site MB-1, *Hainault 3759* (DAO 01-01476909). **Coutts Inlet.** Coutts Mouth: site CO-1, *Coombs 77* (DAO 01-01476922); site CO-2, *Bull 13* (CAN 10092061). **Home Bay.** Cape Hooper: site HO-1, *Elven 3480/99* (ALA V132024, CAN 10073862, O V555478); site HO-3, *Parmelee & Seaborn 3814* (DAO 01-01476926). Ekalugad Head: site EK-1, *Ryder s.n.* (UBC V142127); site EK-4, *Crompton et al. s.n.* (COLO 02378164). Kangirlugag Fiord: site KG-1, *Webber 1226* (CAN 10074062, COLO 02378107), *1316* (COLO 02378123). Rocknoser Fiord: site RF-1, *Smith VP-61-61* (CAN 10074078).

#### ﻿Rhododendron L.

##### Key to species of *Rhododendron* [adapted from Judd and Kron (2009)]

**Table:** 

1	Corolla pink or purple, rarely white; petals connate ¾+ length; inflorescences 3–6-flowered; leaf blades elliptic or ovate	***R. lapponicum***
–	Corolla white or cream; petals connate only at base; inflorescences 10–35-flowered; leaf blades ± linear	**R. tomentosum subsp. decumbens**

##### *Rhododendron lapponicum* (L.) Wahlenb.—Lapland rosebay—Asian (NE)–amphi-Beringian–North American (N)–amphi-Atlantic

Also recorded from Scott’s Bay (Taylor 1863), but no collection located.

**Specimens examined: Agguttinni TP.** Clark Fiord: site 20.8, *Gillespie et al. 12374* (CAN 10116502). Kangiqtualuk Agguqti: site 3.5, *Gillespie et al. 11848* (CAN 10116499); site 6.6, *Gillespie et al. 11895* (CAN 10116503, CHARS, npsp); site 8.2, *Gillespie et al. 11950* (CAN 10116500); site 21.2, *Gillespie et al. 12416* (CAN 10116498). Kangiqtualuk Uqquqti: site 28.2, *Gillespie et al. 11644* (CAN 10116497, npsp). Stewart Valley: site 15.1, *Gillespie et al. 12116* (CAN 10116501). Tasialuk S: site 26.5, *Gillespie et al. 11599* (CAN 10116496, MT). **Clyde Area.** Inugsuin Head: site IG-9, *Parmelee & Seaborn 3943a* (DAO 000001024); site IG-30, *Hainault 3652* (CAN 10076417, DAO 000001015, O V555619, QK 59579). Kangiqtugaapik Head: site KP-1, *Dansereau 500630-1856* (MT00286636), *500630-1956* (DAO 000001025), *500827-1252* (MT00286639), *500827-B29* (MT00286635), *Wynne-Edwards 8825* (CAN 10076421). McBeth Valley: site MB-1, *Hainault 3762* (DAO 000001016, QK 59662). **Home Bay.** Ekalugad Head: site EK-1, *Ryder s.n.* (COLO 02095339). Rocknoser Fiord: site RF-1, *Smith VP-65-61* (CAN 10076405).

##### Rhododendron tomentosum Harmaja subsp. decumbens (Aiton) Elven & D.F.Murray (≡*Ledum decumbens* (Aiton) Lodd. ex Steud.; ≡Ledum palustre L. subsp. decumbens (Aiton) Hultén)—Northern Labrador tea—Asian (N/C)–amphi-Beringian–North American (N)

Also recorded from Cape Adair (as *Ledum palustre*) (Taylor 1863), but no collection located. In the Canadian Arctic Islands this species is found on Baffin, Southampton, and Victoria islands; the northernmost records in Canada are from northern Baffin Island (Porsild and Cody 1980; Aiken et al. 2007).

**Specimens examined: Agguttinni TP.** Atagulisaktalik: site 23.5, *Gillespie et al. 11515* (ALTA, CAN 10116513, npsp). Kuugaaluk: site 19.2, *Gillespie et al. 12293* (ALA, CAN 10116515, CHARS). Tingijattut: site 15.7, *Gillespie et al. 12172* (CAN 10116514, MT). **Clyde Area.** Clyde River: site CR-1, *Dutilly 1484* (DAO 01-01474748). Inugsuin Head: site IG-1, *Hainault 3731* (CAN 10076606, O V555632); site IG-6, *Parmelee & Seaborn 3846a* (DAO 01-01474746). Kangiqtugaapik Head: site KP-1, *Dansereau 500629-2096* (MT00285785, MT00285786), *500710-5198* (MT00286083, MT00286637), *500731-0359* (MT00286638), *500801-0154* (MT00285787); site KP-18, *Wynne-Edwards 8890* (CAN 10076563). McBeth Valley: site MB-1, *Hainault 3749* (O V555630, QK 59585), *3780* (O V555631, QK 59631). **Home Bay.** Ekalugad Head: site EK-1, *Ryder s.n.* (COLO 02077618). Kangirlugag Fiord: site KG-1, *Webber 1320* (COLO 02077592). Rocknoser Fiord: site RF-1, *Smith VP-60-61* (CAN 10076573).

#### ﻿*Vaccinium* L.

##### Key to species of *Vaccinium* [adapted from Saarela et al. (2020b) and Vander Kloet (2009)]

**Table:** 

1	Leaves deciduous, blades usually glaucous abaxially, green to glaucous adaxially; inflorescences axillary; berries blue, 6–8 mm diam.	***V. uliginosum***
–	Leaves persistent, blades pale and glandular abaxially, bright green and shiny adaxially; inflorescences terminal; berries red, 8–10 mm diam.	***V. vitis-idaea***

##### *Vaccinium uliginosum* L. (Fig. 13C)—Bog bilberry—Circumboreal-polar

Also recorded from Coutts Inlet (https://www.inaturalist.org/observations/237449966) and Feachem Bay, Buchan Gulf (https://www.inaturalist.org/observations/20333351).

**Specimens examined: Agguttinni TP.** Atagulisaktalik: site 22.2, *Gillespie et al. 11487* (CAN 10116517, CHARS, MT). Clark Fiord: site 20.1, *Gillespie et al. 12345* (CAN 10116523). Kangiqtualuk Agguqti: site 3.2, *Gillespie et al. 11826* (CAN 10116518, US); site 8.7, *Gillespie et al. 11981* (ALA, CAN 10116519); site 21.7, *Gillespie et al. 12429* (CAN 10116521). Kangiqtualuk Uqquqti: site 28.1, Gillespie et al. 11633 (ALTA, CAN 10116524, MO, npsp). Ravenscraig Harbour: site RH-1, *Dare 32* (CAN 10092377). Stewart Valley: site 15.1, *Gillespie et al. 12118* (CAN 10116520, npsp). Tingijattut: site TG-2, *Dare et al. 89* (CAN 10092382). **Clyde Area.** Clyde River: site CR-1, *Dutilly 9361* (CM408752), *Martin 13* (DAO 01-01521241); site CR-2, *Forbes 29* (DAO 01-01521249), *127* (DAO 01-0152150). Hoffman Cove: site HF-1, *Bartlett 231* (US 02990093). Inugsuin Head: site IG-6, *Parmelee & Seaborn 3829* (DAO 01-01521242); site IG-28, *Hainault 3671* (O V555666, QK 59690). Kangiqtugaapik Head: site KP-1, *Dansereau 500604-0668* (MT00286086), *500624-0259* (MT00286084), *500629-2095* (MT00286088), *Wynne-Edwards 8949* (CAN 10075175); site KP-16, *Dansereau 500707-0451* (MT00286643); site KP-25, *Dansereau 500731-0362* (MT00286642); site KP-27, *Dansereau 500716-0751* (MT00286641). McBeth Valley: site MB-1, *Hainault 3782* (O V555671, QK 59629). **Home Bay.** Cape Hooper: site HO-1, *Elven 3471/99* (ALA V132025, CAN 10076630, O V555660); site HO-3, *Parmelee & Seaborn 3795a* (DAO 01-01521243). Ekalugad FOX-C: site FC-1, *Crompton et al. s.n.* (COLO 02100022), *Richardson & Webber 48* (CAN 10075155, COLO 02100006, QK 71150); site FC-2, *Oswald 358* (QK 135586). Ekalugad Head: site EK-1, *Ryder s.n.* (UBC V162129). Kangirlugag Fiord: site KG-1, *Webber 1234* (CAN 10075210, COLO 02099976). Rocknoser Fiord: site RF-1, *Smith VP-10-61* (CAN 10075146), *VP-51-61* (CAN 10075154).

##### *Vaccinium vitis-idaea* L. (Fig. 13C, D)—Mountain cranberry—Circumboreal-polar

This species is newly reported for the flora area here and in Gillespie et al. (2024) and represents the northernmost record for Canada. A single population several meters wide was found growing among rocks with lichen and some moss on a low bank between two benches on a steep south-facing slope above Arviqtujuq Kangiqtua fiord near its mouth. In the Canadian Arctic this species is recorded from Baffin, Coats, Southampton, and Victoria islands and across the mainland (Polunin 1940; Porsild 1957; Porsild and Cody 1980; Saarela et al. 2020b). Elsewhere on Baffin Island, it is found on the southern part as far north as Nettilling Lake (*Soper s.n.*CAN 10076886, CAN 10076893), about 400 km south of the Agguttinni TP locality. Although Aiken et al. (2007) correctly reported the distribution of *V. vitis-idaea* in the text, their dot map mistakenly shows the distribution of *V. uliginosum*; their mapping database lists 21 localities for *V. vitis-idaea*, consistent with the distribution given above. Therefore, the two localities mapped for the flora area in Aiken et al. (2007) are incorrect.

**Specimens examined: Agguttinni TP.** Arviqtujuq Kangiqtua NW: site 18.4, *Gillespie et al. 12275* (CAN 10116516, npsp).

#### ﻿Onagraceae

***Chamaenerion* Ség.**

##### *Chamaenerion latifolium* (L.) Sweet (≡*Epilobium latifolium* L.; ≡*Chamerion latifolium* (L.) Holub) (Fig. 12H)—Sweet Alpine fireweed—Circumpolar-alpine

Also recorded from Scott’s Bay (as *Epilobium latifolium*) (Taylor 1863), but no collection located.

**Specimens examined: Agguttinni TP.** Atagulisaktalik: site 24.5, *Gillespie et al. 11554* (CAN 10121748, CHARS). Clark Fiord: site 20.1, *Gillespie et al. 12347* (CAN 10121750, npsp). Generator Lake: site GL-14, *Raynolds & Bültmann MKR-2022-25* (CAN 10169267). Kangiqtualuk Agguqti: site 3.6, *Gillespie et al. 11858* (CAN 10121752, CHARS, QFA, US); site 8.2, *Gillespie et al. 11919* (CAN 10121753); site 21.8, *Gillespie et al. 12431* (CAN 10121756). Kangiqtualuk Uqquqti: site 28.1, *Gillespie et al. 11628* (ALTA, CAN 10121742, MO, MT, npsp). Refuge Harbour: site 12.4, *Gillespie et al. 12044* (CAN 10121751). Stewart Valley: site 15.1, *Gillespie et al. 12123* (CAN 10121754). Tasialuk S: site 26.5, *Gillespie et al. 11585* (ALA, CAN 10121749). Tingijattut: site 15.5, *Gillespie et al. 12168* (CAN 10121755, UBC). **Clyde Area.** Clyde River: site CR-1, *Martin 40* (DAO 01-01363431). Hoffman Cove: site HF-1, *Bartlett 235* (US 01362017). Inugsuin Head: site IG-1, *Hainault 3741* (DAO 01-01363444, O V555354, QK18300673); site IG-6, *Parmelee & Seaborn 3831* (DAO 01-01363693); site IG-9, *Parmelee & Seaborn 3933* (ALA V152167, ALTA VP 135699, BC-927824, BISH 753833, BR0000009384415, BR0000031228213, DAO 01-01363694, F V0358789F, L.4175791, MO 100802539, PE 2002755, PE 2002756, S13-12092, TRH 254763, TROM 135121, TRT00024920, UTC00259544, W 2013-0002619); site IG-15, *Parmelee & Seaborn 3857a* (DAO 01-01363691). Inugsuin Mouth: site IM-1, *Hainault 3823* (DAO 01-01363443, O V555355, QK18300670). Kangiqtugaapik Head: site KP-1, *Dansereau 500710-0259* (MT00204053), *500803-0477* (MT00204054), *Wynne-Edwards 8922* (CAN 10001715); site KP-13, *Dansereau 500608-0352* (MT00204052); site KP-15, *Dansereau 500713-0869* (MT00204055); site KP-25, *Dansereau 500731-0358* (DAO 01-01363666, MT00204051). **Coutts Inlet.** Coutts Mouth: site CO-1, *Coombs 71* (DAO 01-01363668); site CO-2, *Bull 12* (CAN 10092056). **Home Bay.** Cape Hooper: site HO-1, *Elven 3486/99* (ALA V132042, CAN 10001716, O V555349). Ekalugad FOX-C: site FC-1, *Richardson & Webber 64* (CAN 10001727). Rocknoser Fiord: site RF-1, *Smith VP-48-61* (CAN 10001724), *VP-78-61* (CAN 10001714), *Webber 1196* (CAN 10001725).

#### ﻿*Epilobium* L.

##### *Epilobium arcticum* Sam.—Arctic willowherb—Nearly circumpolar

This species has a sparse, scattered distribution across the Canadian Arctic islands and adjacent mainland. It was first mapped for the flora area in the Home Bay area by Porsild and Cody (1980) and Aiken et al. (2007).

**Specimens examined: Agguttinni TP.** Generator Lake: site GL-20, *Raynolds & Bültmann MKR-2022-19* (CAN 10169254). **Home Bay.** Kangirlugag Fiord: site KG-1, *Webber 1288* (CAN 10001811, COLO 02282721). Rocknoser Fiord: site RF-1, *Smith VP-84-61* (CAN 10001800).

#### ﻿Orobanchaceae

***Pedicularis* L.**

##### Key to species of *Pedicularis* [adapted from Porsild and Cody (1980) and Saarela et al. (2020b)]

**Table:** 

1	Corollas yellow with upper half of galea dark red or purple	***P. flammea***
–	Corollas pink	**2**
2	Corollas bright pink; taproot bright yellow; flowering stems stout, 3.5–6 mm wide; inflorescences 3–4.5 cm wide	***P. lanata***
–	Corollas pale pink; taproot pale yellow; flowering stems slender, 2–4 mm wide; inflorescences 2–3 cm wide	***P. hirsuta***

##### *Pedicularis flammea* L.—Red-tipped lousewort—North American (N)–amphi-Atlantic (Iceland)

**Specimens examined: Agguttinni TP.** Kangiqtualuk Agguqti: Site 6.1, *Gillespie et al. 11876* (CAN 10116669); site 8.7, *Gillespie et al. 11978* (CAN 10116666); site 21.5, *Gillespie et al. 12424* (CAN 10116667, npsp). Kangiqtualuk Uqquqti: site 27.1, *Gillespie et al. 11620* (CAN 10116668, npsp). **Clyde Area.** Inugsuin Head: site IG-1, *Hainault 3730* (DAO 01-01643080, QK 59526); site IG-7, *Parmelee & Seaborn 3925* (DAO 01-01643072); site IG-11, *Parmelee & Seaborn 3983* (DAO 01-01643073); site IG-15, *Parmelee & Seaborn 3876* (DAO 01-01643071); site IG-21, *Hainault 3743* (CAN 10081002, DAO 01-01643081, O V556018, QK 59619). Kangiqtugaapik Head: site KP-1, *Dansereau 500711-4254* (MT00204114), *500801-0455* (MT00204115), *Wynne-Edwards 88*94 (CAN 10081046). **Home Bay.** Cape Hooper: site HO-1, *Elven 3594/99* (CAN 10081036). Ekalugad Head: site EK-1, *Ryder s.n.* (COLO 01661735). Rocknoser Fiord: site RF-1, *Smith VP-62-61* (CAN 10080996).

##### *Pedicularis hirsuta* L. (Fig. 10B)—Hairy lousewort—Circumpolar

Also recorded from Scott’s Bay (Taylor 1863), but no collection located, and Feachem Bay, Buchan Gulf (https://www.inaturalist.org/observations/178358816).

**Specimens examined: Agguttinni TP.** Atagulisaktalik: site 22.2, *Gillespie et al. 11486* (CAN 10116697). Clark Fiord: site 20.5, *Gillespie et al. 12367* (CAN 10116701). Generator Lake: site GL-21, *Raynolds & Bültmann MKR-2022-53* (CAN 10169274). Gibbs Fiord: site 20.9, *Gillespie et al. 12384* (CAN 10116702). Kangiqtualuk Agguqti: site 3.2, *Gillespie et al. 11827* (CAN 10116696); site 8.2, *Gillespie et al. 11948* (CAN 10116699); site 21.6, *Gillespie et al. 12428* (CAN 10116703). Kangiqtualuk Uqquqti: site 29.1, *Gillespie et al. 11683* (CAN 10116705). Kuugaaluk: site 19.1, *Gillespie et al. 12292* (CAN 10116704). Marble Lake: site 31.3, *Gillespie et al. 11745* (CAN 10116706). Niaqurnaaluk-Qassialuit: site 18.2, *Gillespie et al. 12245* (CAN 10116707, npsp). Refuge Harbour: site 11.1, *Gillespie et al. 12008* (CAN 10116698); site 12.8, Gillespie et al. 12055 (CAN 10116694). Tasialuk S: site 21.12, *Gillespie et al. 12443* (CAN 10116700). Tingijattut: site 15.4, *Gillespie et al. 12157* (CAN 10116695). **Clyde Area.** Clyde River: site CR-1, *Dutilly 9355* (QFA0158294), *Martin 10* (DAO 01-0161197, QFA0615045), *Polunin 634* (CAN 10080961); site CR-2, *Forbes 20* (DAO 01-01641164), *62* (DAO 01-01641153), *110* (DAO 01-01641152). Hewett Cache: site HC-1, *Hainault 3801* (QK 60112). Hoffman Cove: site HF-1, *Bartlett 226* (CAN 10080913, US 03922225). Inugsuin Head: site IG-2, *Hainault 3729* (DAO 01-01641154, O V556029, QK 59613); site IG-6, *Parmelee & Seaborn 3830* (DAO 01-01641191); site IG-15, *Parmelee & Seaborn 3877* (DAO 01-01641190); site IG-21, *Hainault 3627* (QK 59596). Inugsuin Mouth: site IM-1, *Hainault 3841* (CAN 10080929, DAO 01-01641156, O V556030, QK 59651). Kangiqtugaapik Head: site KP-1, *Dansereau 500628-3155* (MT00204103), *500709-0352* (MT00204101), *500709-0870* (MT00204108), *500720-0156* (MT00204105), *Wynne-Edwards 8861* (CAN 10080950); site KP-18, *Wynne-Edwards 8891* (CAN 10080960); site KP-24, *Wynne-Edwards 8831* (CAN 10080949). McBeth Valley: site MB-1, *Hainault 3775* (DAO 01-01641155, QK 59669). **Coutts Inlet.** Coutts Mouth: site CO-1, *Coombs 79* (DAO 01-01641170). **Home Bay.** Cape Hooper: site HO-1, *Elven 3461/99* (ALA V131932, CAN 10080909, O V556024); site HO-3, *Parmelee & Seaborn 3799* (DAO 01-01641188), *3800* (DAO 01-01641202), *3809* (DAO 01-01641189), *3816* (DAO 01-01641192). Ekalugad FOX-C: site FC-1, *Richardson & Webber 33* (CAN 10080926, COLO 01662717). Ekalugad Head: site EK-1, *Ryder s.n.* (COLO 01662733); site EK-3, *Crompton et al. s.n.* (COLO 01662808). Kangirlugag Fiord: site KG-1, *Webber 1235* (CAN 10080921, COLO 01662725). Rocknoser Fiord: site RF-1, *Smith VP-16-61* (CAN 10080957).

##### *Pedicularis lanata* Willd. ex Cham. & Schltdl.—Woolly lousewort—amphi-Beringian–North American (N)

**Specimens examined: Agguttinni TP.** Kangiqtualuk Agguqti: site 3.5, *Gillespie et al. 11847* (CAN 10116682, npsp). Kangiqtualuk Uqquqti: site 28.6, *Gillespie et al. 11669* (CAN 10116681). Refuge Harbour: site 12.1, *Gillespie et al. 12037* (CAN 10116683). **Clyde Area.** Kangiqtugaapik Head: site KP-18, *Wynne-Edwards 8820* (CAN 10080268).

#### ﻿Papaveraceae

##### *Oreomecon* Banfi, Bartolucci, J.-M.Tison & Galasso

Arctic poppies have previously been placed in Papaver L. sect. Meconella, but this section has recently been segregated as a distinct genus, *Oreomecon*, based on molecular phylogenetic data (Carolan et al. 2006; Banfi et al. 2022; Elvebakk and Bjerke 2024). Two species of *Oreomecon* are present in the flora area. Although apparently not closely related based on AFLP data (Solstad 2009), they can be difficult to distinguish. Most key characters are variable and overlap. While some specimens can easily be identified to species, others seem to show characters of both species or in the range of overlap and are difficult to place. Both species are high polyploids (octoploid), and reticulate evolution may be at play here. Solstad and Elven (unpubl.), in their latest key, described the hairs on the leaf, scape, and calyx of *O. labradorica* to be strongly and longer-toothed (dentate) than those of *O. lapponica*, but we found this to be only sometimes true. Further study is needed to more fully understand the boundary between these two species. Species circumscriptions have changed considerably over time, with Porsild (1957) treating all *Papaver* in the eastern Canadian Arctic within a broadly defined *P. radicatum* Rottb. Porsild and Cody (1980) considered two species to occur in this region, with only *P. radicatum* s.l. in the flora area, whereas Solstad in Aiken et al. (2007) treated these and other species under *Papaver* spp., discussing but not mapping the separate species.

##### Key to *Oreomecon* [adapted from Solstad (2009) and Solstad and Elven (unpubl.)]

**Table:** 

1	Leaf blades usually lobed more than 75% and often to mid-axis, lobes usually undivided, rarely divided, strap-shaped, or lanceolate to oblanceolate, lowermost pair at 20–40° (–45°) to mid-axis, usually pointing ± forward; hairs on distal scape pale to medium brown, usually distinctly contrasting with darker sepal hairs; petals usually as wide as long	***O. labradorica***
–	Leaf blades usually lobed less than 75% to mid-axis, sometimes more, lobes divided or undivided, lanceolate to oblanceolate or obovate, lowermost pair at 30–50° to mid-axis, usually spreading; hairs on distal scape medium to dark brown, similar in color to the sepal hairs; petals usually some longer than wide	***O. lapponica***

##### *Oreomecon labradorica* (Fedde) Krivenko (≡*Papaver labradoricum* (Fedde) Solstad & Elven)—Labrador poppy—North American (NE)

Also recorded from Icy Arm, Quernbiter Fiord (https://www.inaturalist.org/observations/20818541).

**Specimens examined: Agguttinni TP.** Atagulisaktalik: site 23.3, *Gillespie et al. 11512* (CAN 10116527, MT, npsp, UBC). Kangiqtualuk Uqquqti: site 30.2, *Gillespie et al. 11713* (ALTA, CAN 10116528, CHARS, MO, US). Niaqurnaaluk-Qassialuit: site 18.2, *Gillespie et al. 12249* (ALA, CAN 10116526, QFA). Tasialuk S: site 26.5, *Gillespie et al. 11586* (CAN 10116534). Tingijattut: site 15.4, *Gillespie et al. 12151* (CAN 10116530). **Clyde Area.** Cape Hewett: site CH-1, *Platt 481* (NY 3702224), *482* (NY 3702278). Clyde River: site CR-1, *Martin 7* (DAO 01-01667449); site CR-2, *Forbes 18* (DAO 01-01667450), *57* (DAO 01-01667451); site CR-3, *Dare 48* (CAN 10092509). Inugsuin Head: site IG-1, *Hainault 3658* (CAN 10051407, DAO 01-01000592384, QK 59697); site IG-6, *Parmelee & Seaborn 3849* (DAO 01-01667448). Hoffman Cove: site HF-1, *Bartlett 237* (US 03592703). Inugsuin Mouth: site IM-1, *Hainault 3954* (DAO 01-01667453, QK 59786). Kangiqtugaapik Head: site KP-1, *Dansereau 500625-0351* (MT00285814), *500628-3260* (MT00285821), *500630-1751* (MT00285818), *500705-0162* (MT00285816), *500708-0959* (DAO 01-01667452, MT00285815), *Dansereau 500708-0960* (MT00285817), *Wynne-Edwards 9012* (CAN 10051472). McBeth Valley: site MB-1, *Hainault 3766* (DAO 01-01667454, QK 59664). **Coutts Inlet.** Coutts Mouth: site CO-1, *Coombs 68* (DAO 01-01667648). **Home Bay.** Cape Hooper: site HO-1, *Elven 3456/99* (ALA V132061, CAN 10051410, CAN 10051411), *3489/99* (ALA V132072, CAN 10051420); site HO-3, *Parmelee & Seaborn 3797* (DAO 552415). Ekalugad FOX-C: site FC-1, *Richardson & Webber 1* (CAN 10051415, QK 71109). Kangirlugag Fiord: site KG-1, *Webber 1217* (CAN 10051465). Rocknoser Fiord: site RF-1, *Smith VP-49-61* (CAN 10051466), *VP-8-61* (CAN 10051423).

##### *Oreomecon lapponica* (Tolm.) Galasso, Banfi & Bertolucci subsp. occidentalis (C.E.Lundstr.) Elvebakk & Bjerke (≡Papaver lapponicum (Tolm.) Nordh. subsp. occidentale (C.E.Lundstr.) Knaben)—Lapland poppy—North American (N)–amphi-Atlantic–European (N)–Asian (N)

Although typically with yellow flowers, one population on the Barnes Plateau was seen with both yellow- and white-flowered individuals (*Gillespie et al. 11766*). This population resembles *O. dahliana* (Nordh.) Galasso, Banfi & Bartolucci and *O. cornwallisensis* (D.Löve) Elvebakk & Bjerke in flower color and their ascending flowering stems, but flowers have a greater number of stamens (40-50) and also lack the long stigmatic papillae characteristic of *O. dahliana*.

**Specimens examined: Agguttinni TP.** Atagulisaktalik: site 24.3, *Gillespie et al. 11550* (CAN 10116531, WIN). Generator Lake: site 31.6, *Gillespie et al. 11766* (ALTA, CAN 10116525, CHARS, MO, MT, NY); site GL-18, *Raynolds & Bültmann MKR-2022-56* (CAN 10169277). Gibbs esker: site 20.12, *Gillespie et al. 12396* (CAN 10116533). Gibbs Fiord: site 20.11, *Gillespie et al. 12392* (CAN 10116532). Kangiqtualuk Agguqti: site 3.2, *Gillespie et al. 11834* (ALA, CAN 10116529, npsp, US); site 8.2, *Gillespie et al. 11920* (CAN 10116537, MT). Kuugaaluk: site 16.4, *Gillespie et al. 12209* (CAN 10116538, MO, WIN). Niaqurnaaluk-Qassialuit: site 18.1, *Gillespie et al. 12232* (ALA, CAN 10116540, MT, QFA). Refuge Harbour: site 11.3, *Gillespie et al. 12033* (CAN 10116539); site 14.4, *Gillespie et al. 12091* (CAN 10116536). Stewart Valley: site 15.1, *Gillespie et al. 12110* (CAN 10116535). **Clyde Area.** Clyde River: site CR-1, *Polunin 2593* (CAN 10052532); site CR-2, *Forbes 108* [cf.] (DAO 01-01000592383). Inugsuin Head: site IG-23, *Hainault 3648* (DAO 01-01667455, QK 59577). Kangiqtugaapik Head: site KP-1, *Dansereau 500627-4181* (MT00285820), *500708-1063* (MT00285819), *Wynne-Edwards 8856* (CAN 10052529). **Coutts Inlet.** Coutts Fiord: site CF-1, *Coombs 44* (DAO 01-01667647). Coutts Mouth: site CO-1, *Coombs 68* (DAO 01-01667648). **Home Bay.** Ekalugad FOX-C: site FC-2, *Oswald 352* (QK 135585).

#### ﻿Plantaginaceae

***Hippuris* L.**

##### *Hippuris lanceolata* Retz.—Lance-leaved mare’s-tail—Circumpolar

The Kangiqtugaapik Head locality was mapped (as H. × lanceolata) in Aiken et al. (2007). The two cited records and one from west central Baffin (*Beschel & Webber 642*CAN 10073797) are the northernmost records of this aquatic species on Baffin Island. Further north it is uncommon on Axel Heiberg, Devon, and Ellesmere islands. The Inugsuin Head specimen, *Parmelee & Seaborn 3898*, was incorrectly mapped by Aiken et al. (2007) as *H. vulgaris* L. (see Excluded Taxa).

**Specimens examined: Clyde Area.** Kangiqtugaapik Head: site KP-9, *Wynne-Edwards 9082* (CAN 10073799). Inugsuin Head: site IG-14, *Parmelee & Seaborn 3898* (DAO 01-01435907).

#### ﻿Plumbaginaceae

***Armeria* Willd.**

##### Armeria maritima (Mill.) Willd. subsp. sibirica (Turcz. ex Boiss.) Nyman (=Armeria maritima subsp. labradorica (Wallr.) Hultén)—Sea thrift—Circumpolar

**Specimens examined: Agguttinni TP.** Atagulisaktalik: site 23.6, *Gillespie et al. 11518* (CAN 10116711). Clark Fiord: site 20.7, *Gillespie et al. 12369* (CAN 10116716, QFA, UBC). Gibbs Fiord: site 20.11, *Gillespie et al. 12405* (CAN 10116710). Kangiqtualuk Agguqti: site 6.3, *Gillespie et al. 11887* (ALA, CAN 10116713, MO, MT); site 8.1, *Gillespie et al. 11903* (CAN 10116708, US). Kangiqtualuk Uqquqti: site 27.1, *Gillespie et al. 11605* (CAN 10116712). Kuugaaluk: site 16.1, *Gillespie et al. 12188* (CAN 10116715). Tasialuk N: site 26.3, *Gillespie et al. 11581* (CAN 10116709, MT, npsp). Tasialuk S: site 21.12, *Gillespie et al. 12446* (CAN 10116717); site 26.5, *Gillespie et al. 11587* (ALTA, CAN 10116714, CHARS). **Clyde Area.** Inugsuin Head: site IG-1, *Hainault 3742* (CAN 10079158, DAO 01-01533722, O V548093, QK 59618); site IG-6, *Parmelee & Seaborn 3848* (DAO 01-01533731); site IG-8, *Parmelee & Seaborn 3957* (DAO 01-01533727). Inugsuin Mouth: site IM-1, *Hainault 3909* (DAO 01-01533723, O V548094). Kangiqtugaapik Head: site KP-1, *Wynne-Edwards 8948* (CAN 10079153); site KP-2, *Martin 56* (DAO 01-015333725). **Coutts Inlet.** Coutts Fiord: site CF-1, *Coombs 46* (DAO 01-01533941). **Home Bay.** Ekalugad Head: site EK-4, *Crompton et al. s.n.* (COLO 02218808). Kangirlugag Fiord: site KG-1, *Webber 1300* (CAN 10079159, COLO 02218790, QK 71154). Rocknoser Fiord: site RF-1, *Smith VP-99-61* (CAN 10079174).

#### ﻿Polygonaceae

***Bistorta* (L.) Scop.**

##### *Bistorta vivipara* (L.) Delarbre (≡*Polygonum viviparum* L.)—Alpine bistort—Circumboreal-polar

**Specimens examined: Agguttinni TP.** Atagulisaktalik: site 22.2, *Gillespie et al. 11481* (CAN 10116728, npsp). Clark Fiord: site 20.1, *Gillespie et al. 12344* (CAN 10116737). Gibbs Fiord: site 20.11, *Gillespie et al. 12406* (CAN 10116729). Kangiqtualuk Agguqti: site 3.5, *Gillespie et al. 11845* (CAN 10116730); site 3.6, *Gillespie et al. 11859* (CAN 10116731); site 8.2, *Gillespie et al. 11931* (CAN 10116732, CHARS). Kangiqtualuk Uqquqti: site 29.2, *Gillespie et al. 11685* (CAN 10116739, npsp). Kuugaaluk: site 16.1, *Gillespie et al. 12189* (CAN 10116736). Refuge Harbour: site 11.2, *Gillespie et al. 12018* (CAN 10116733, MT). Stewart Valley: site 15.1, *Gillespie et al. 12109* (ALA, CAN 10116734). Tasialuk S: site 21.12, *Gillespie et al. 12448* (CAN 10116738, MT). Tingijattut: site 15.3, *Gillespie et al. 12150* (CAN 10116735); site TG-2, *Dare et al. 82* (CAN 10092725). **Clyde Area.** Clyde River: site CR-1, *Dutilly 1426* (CM412984, QFA 39611), *9360* (QFA0157886), *Martin 21* (DAO 01-01001133805), *Polunin 640* (CAN 10033097); site CR-2, *Forbes 38* (DAO 01-01001133816), *141* (DAO 01-01001133815). Hoffman Cove: site HF-1, *Bartlett 223* (US 03226066). Inugsuin Head: site IG-6, *Parmelee & Seaborn 3844* (DAO 01-01001133813), *3840a* (DAO 01-01001133809); site IG-16, *Hainault 3707* (CAN 10033231, DAO 01-01001133890, O V548067, QK 59640). Kangiqtugaapik Head: site KP-1, *Dansereau 500625-0261* (MT00285747), *500710-0153* (MT00285750), *500803-0153* (MT00285751), *500803-0287* (MT00285779), *Wynne-Edwards 8940* (CAN 10033228); site KP-5, *Wynne-Edwards 9043* (CAN 10033229); site KP-15, *Dansereau 500713-0872* (MT00285749). **Coutts Inlet.** Coutts Fiord: site CF-1, *Coombs 42* (DAO 01-01001133803). Coutts Mouth: site CO-1, *Coombs 66* (DAO 01-01001133810). **Home Bay.** Cape Hooper: site HO-1, *Elven 3460/99* (O V548054), *3499/99* (O V548055); site HO-3, *Parmelee & Seaborn 3790* (DAO 01-01001133806). Ekalugad FOX-C: site FC-1, *Richardson & Webber 29* (CAN 10033224, COLO 01690395); site FC-2, *Oswald 345* (QK 135590). Ekalugad Head: site EK-4, *Crompton et al. s.n.* (COLO 01690445). Kangirlugag Fiord: site KG-1, *Webber 1232b* (CAN 10033232, COLO 01690296). Rocknoser Fiord: site RF-1, *Smith VP-105-61* (CAN 10033226), *Webber 1192* (COLO 01690403).

#### ﻿*Koenigia* L.

##### *Koenigia islandica* L.—Iceland purslane—Circumpolar-alpine

**Specimens examined: Agguttinni TP.** Atagulisaktalik: site 23.8, *Gillespie et al. 11524* (CAN 10121762). Kangiqtualuk Agguqti: site 5.4, *Gillespie et al. 11870* (ALTA, CAN 10121760, CHARS, MT); site 21.2, *Gillespie et al. 12420* (CAN 10121770, MO, npsp). Niaqurnaaluk-Qassialuit: site 18.2, *Gillespie et al. 12246* (CAN 10121761). Refuge Harbour: site 12.4, *Gillespie et al. 12048* (ALA, CAN 10121759). **Clyde Area.** Clyde River: site CR-1, *Dutilly 634* (CM416164, QFA 39617). Inugsuin Head: site IG-22, *Hainault 4046* (CAN 10035802, O V548074, QK 59730). Inugsuin Mouth: site IM-1, *Hainault 3879* (CAN 10035801, O V548075, QK 60101). Kangiqtugaapik Head: site KP-1, *Dansereau 500713-1067* (MT00285746), *500801-0467* (MT00285745), *Wynne-Edwards 8955* (CAN 10035794). **Home Bay.** Cape Hooper: site HO-1, *Elven 3468/99* (ALA V132358, CAN 10035795). Rocknoser Fiord: site RF-1, *Smith VP-58-61* (CAN 10035799), *VP-91-61* (CAN 10035803).

#### ﻿*Oxyria* Hill

##### *Oxyria digyna* (L.) Hill—Mountain-sorrel—Circumpolar-alpine

**Specimens examined: Agguttinni TP.** Atagulisaktalik: site 22.1, *Gillespie et al. 11468* (CAN 10116602, CHARS, MT, npsp); site 23.3, *Gillespie et al. 11505* (ALTA, CAN 10116605, MO). Clark Fiord: site 20.1, *Gillespie et al. 12342* (CAN 10116612, npsp). Generator Lake: site GL-20, *Raynolds & Bültmann* MKR-2022-46 (CAN 10169285). Kangiqtualuk Agguqti: site 3.2, *Gillespie et al. 11828* (ALA, CAN 10116604, QFA); site 8.2, *Gillespie et al. 11935* (CAN 10116607). Kangiqtualuk Uqquqti: site 28.1, *Gillespie et al. 11630* (CAN 10116603, US, WIN). Kuugaaluk: site 16.1, *Gillespie et al. 12197* (CAN 10116610). Niaqurnaaluk-Qassialuit: site 18.1, *Gillespie et al. 12233* (ALA, CAN 10116611, NY, WIN). Ravenscraig Harbour: site RH-1, *Dare 5* (CAN 10092732). Refuge Harbour: site 11.3, *Gillespie et al. 12029* (CAN 10116608). Tingijattut: site 15.2, *Gillespie et al. 12132* (CAN 10116609, MT). **Clyde Area.** Clyde River: site A22.5, *Gillespie et al. 12470* (CAN 10116606); site CR-1, *Dutilly 1448* (MT00286599, QFA 20103, QFA (no acc. num.)), *9348* (QFA0137746), *Martin 5* (DAO 01-01001126323), *Polunin 609* (CAN 10031764); site CR-2, *Forbes 4* (DAO 01-01001126331), *46* (DAO 01-01001126330), *100* (DAO 01-01001126329). Hoffman Cove: site HF-1, *Bartlett 248* (US 03286703). Inugsuin Head: site IG-1, *Hainault 3693* (DAO 01-01001126326, QK 59714); site IG-6, *Parmelee & Seaborn 3847* (DAO 01-01001126339). Kangiqtugaapik Head: site KP-1, *Dansereau 500624-0156* (MT00285508), *500627-2454* (MT00285744), *500630-1755* (MT00285780), *500710-0260* (MT00285506), *500710-0365* (MT00285505), *500801-0260* (MT00285507, MT00285788), *Wynne-Edwards 8911* (CAN 10015296). **Coutts Inlet.** Coutts Fiord: site CF-1, *Coombs 43* (DAO 01-01000126325). Coutts Mouth: site CO-1, *Coombs 67* (DAO 01-01001126324). **Home Bay.** Cape Hooper: site HO-1, *Elven 3469/99* (CAN 10031815); site HO-3, *Parmelee & Seaborn 3813* (DAO 01-01001126340). Ekalugad FOX-C: site FC-1, *Crompton et al. s.n.* (COLO 01724855), *Crompton et al. s.n.* (COLO 01724863), *Richardson & Webber 4* (CAN 10031808, COLO 01724848, QK 71096); site FC-2, *Oswald 353* (QK 135589). Kangirlugag Fiord: site KG-1, *Webber 1219* (CAN 10031765, COLO 01724822, QK 71097). Rocknoser Fiord: site RF-1, *Smith VP-23-61* (CAN 10031809), *VP-83-61* (CAN 10015297).

#### ﻿Ranunculaceae

***Ranunculus* L.**

##### Key to *Ranunculus* [adapted from Porsild and Cody (1980), Saarela et al. (2020b), and Whittemore (1997)]

**Table:** 

1	Plants aquatic, stems floating; leaf blades filiform-dissected; corollas white with yellow centre	***R. trichophyllus***
–	Plants terrestrial, paludal, or amphibious; stems erect, decumbent, creeping, or floating; leaf blades lobed or divided but never filiform-dissected, sometimes unlobed; corollas yellow	**2**
2	Plants amphibious, glabrous; stems prostrate, creeping, or floating, rooting at nodes; petals 3–4	**R. hyperboreus subsp. hyperboreus**
–	Plants terrestrial or paludal, glabrous or hairy; stems usually erect or ascending, if decumbent, rooting only at base, never floating; petals 5(–10)	**3**
3	Abaxial surface of sepals with dense brown pubescence	**4**
–	Abaxial surface of sepals glabrous or with colorless hairs	**5**
4	Receptacle glabrous; basal leaf blades 3-parted, at least lateral segments again lobed or with toothed margins	***R. nivalis***
–	Receptacle brown-pilose; basal leaf blades usually shallowly lobed, sometimes unlobed with crenate margins	***R. sulphureus***
5	Petals 7–15 mm long; leaf blades pedately (5–)7(–9)-parted or -divided, segments undivided or again lobed or parted; flowering stems 6–35 cm tall	***R. arcticus***
–	Petals 1–8 mm long; leaf blades 3-lobed or -divided, segments undivided or again lobed; flowering stems 0.6–12 cm tall	**6**
6	Petals 1.2–3.5 × 1.1–2.8 mm; sepals 2–4 × 1.2–1.6 mm; pedicels glabrous or pubescent; flowering stems 0.6–3.5 cm (sometimes longer in fruit); leaf blades 0.45–0.9 × 0.6–1.3 cm; heads of achenes nearly globose to cylindric, 2.5–7 × 2.5–5 mm	***R. pygmaeus***
–	Petals 5–8 × 3–4 mm; sepals 4–7 × 2–3 mm; pedicels pilose; flowering stems 1–12 cm; leaf blades 0.9–3 × 0.8–3.4 cm; heads of achenes cylindric, 6–9 × 4 mm	***R. sabinei***

##### *Ranunculus arcticus* Richardson (=*Ranunculus affinis* R.Br.; =Ranunculus pedatifidus var. leiocarpus (Trautv.) Fernald) —Northern buttercup—Circumpolar-alpine

This species was found only at the heads of two fiords in the flora area, Kangiqtualuk Agguqti and Kangiqtugaapik. Aiken et al. (2007) mapped a second collection from the Clyde area (*Bartlett 224*CAN, Hoffman Bay), but this collection has been redetermined as *R. nivalis*; Porsild (1957) had mapped the same two Clyde area localities, but curiously Porsild and Cody (1980) mapped neither. Although found across the Canadian Arctic Islands except the central part (Porsild 1957; Porsild and Cody 1980; Aiken et al. 2007), *R. arcticus* is rare in central and northern Baffin Island, known from only three localities: the two cited here and Arctic Bay (*Warwick s.n.*DAO 211524).

**Specimens examined: Agguttinni TP.** Kangiqtualuk Agguqti: site 21.1, *Gillespie et al. 12408* (CAN 10116561, MT, npsp). **Clyde Area.** Kangiqtugaapik Head: site KP-8, *Wynne-Edwards 9001* (CAN 10051067).

##### Ranunculus hyperboreus Rottb. subsp. hyperboreus—Far-northern buttercup—Circumpolar-alpine

Also recorded from Cape Adair (as *R. hyperboreus*) (Taylor 1863), but no collections located.

**Specimens examined: Agguttinni TP.** Atagulisaktalik: site 22.3, *Gillespie et al. 11499* (CAN 10121772, CHARS, MT). Generator Lake: site GL-15, *Raynolds & Bültmann MKR-2022-26* (CAN 10169251). Kangiqtualuk Agguqti: site 21.2, *Gillespie et al. 12418* (ALA, CAN 10121773). Refuge Harbour: site 14.2, *Gillespie et al. 12088* (CAN 10121771, npsp). Scott Inlet: site SI-1, *Taylor s.n.* (MEL 2424352). **Clyde Area.** Inugsuin Head: site IG-3, *Parmelee & Seaborn 3996* (DAO 01-01667507, DAO 01-01667509); site IG-14, *Parmelee & Seaborn 3900* (DAO 01-01667508); site IG-20, *Hainault 4008* (CAN 10050634, DAO 01-01667510, O V548668, QK 59838). Kangiqtugaapik Head: site KP-1, *Dansereau 500804-0781* (MT - no acc. num.), *Wynne-Edwards 8941* (CAN 10050583); site KP-2, *Martin 41* (DAO 91605); site KP-5, *Wynne-Edwards 9039* (CAN 10050581). **Home Bay.** Rocknoser Fiord: site RF-1, *Smith VP-90-61* (CAN 10050624).

##### *Ranunculus nivalis* L.—Snow buttercup—Circumpolar

**Specimens examined: Agguttinni TP.** Atagulisaktalik: site 22.2, *Gillespie et al. 11479* (CAN 10121757, MT, npsp, UBC); site 23.1, *Gillespie et al. 11504* (ALTA, CAN 10121777, MO, npsp). Generator Lake: site GL-19, *Raynolds & Bültmann MKR-2022-52* (CAN 10169293). Kangiqtualuk Agguqti: site 3.5, *Gillespie et al. 11853* (ALA, CAN 10121758, US), *Gillespie et al. 11854* (CAN 10121763). Kangiqtualuk Uqquqti: site 28.4, *Gillespie et al. 11660* (CAN 10121778). Kuugaaluk: site 16.7, *Gillespie et al. 12221* (CAN 10121765, npsp); site 19.4, *Gillespie et al. 12314* (CAN 10121766, MT), *Gillespie et al. 12321* (CAN 10121767, CHARS, QFA, WIN). Refuge Harbour: site 11.3, *Gillespie et al. 12058* (ALA, CAN 10121769). Tingijattut: site 15.4, *Gillespie et al. 12155* (CAN 10121764). **Clyde Area.** Clyde River: site 10.2, *Gillespie et al. 11996* (CAN 10121768, MT); site CR-1, *Martin 1* (DAO 01-01667460, QFA0615087), *Dutilly 1458* (QFA0615084); site CR-2, *Forbes 1* (DAO 01-01667459), *Forbes 47* (DAO 01-01667463), *Forbes 98* (DAO 01-01667464). Hewett Cache: site HC-1, *Hainault 3796* (CAN 10050838, DAO 01-01667458, O V548716, QK 59644). Hoffman Cove: site HF-1, *Bartlett 224* (CAN 10050833, US 03568667). Inugsuin Head: site IG-1, *Hainault 3653* (DAO 01-01667456, O V548715, QK 59580); site IG-6, *Parmelee & Seaborn 3832* (DAO 01-01000677185, DAO 01-01667461), *3839* (DAO 538983); site IG-9, *Parmelee & Seaborn 3938* (DAO 01-01000677573, DAO 01-01667462), *3942* (DAO 538982). Inugsuin Mouth: site IM-1, *Hainault 3813* (DAO 01-01667457, O V548717, QK 59563). Kangiqtugaapik Head: site KP-1, *Dansereau 500610-2553* (MT00203790, MT00203793), *500614-1351* (MT00203791), *500701-0452* (MT00203792), *500717-0151* (MT00203795), *500720-0452* (MT00203794), *Wynne-Edwards 8811* (CAN 10050820), *8932* (CAN 10050799), *9096* (CAN 10050821); site KP-6, *Wynne-Edwards 9024* (CAN 10050798); site KP-20, *Wynne-Edwards 8868* (CAN 10050801); site KP-24, *Wynne-Edwards 8830* (CAN 10050797). **Home Bay.** Cape Hooper: site HO-1, *Elven 3484/99* (CAN 10050787), *3487/99* (ALA V132050, O V548696); site HO-3, *Parmelee & Seaborn 3811* (DAO 91878), *3815* (DAO 91880). Ekalugad FOX-C: site FC-1, *Philpot et al. s.n.* (COLO 02403194). *Richardson & Webber 61* (CAN 10050837, COLO 02403145). Ekalugad Head: site EK-1, *Ryder s.n.* (COLO 02403160). Kangirlugag Fiord: site KG-1, *Webber 1179* (CAN 10050836, COLO 02403152, QK 71107), *1220* (COLO 02403186). Rocknoser Fiord: site RF-1, *Smith VP-22-61* (CAN 10050782).

##### *Ranunculus pygmaeus* Wahlenb.—Pygmy buttercup—Circumpolar-alpine

Also recorded from Scott’s Bay (Taylor 1863), but no collection located.

**Specimens examined: Agguttinni TP.** Atagulisaktalik: site 23.4, *Gillespie et al. 11513* (CAN 10121775). Kuugaaluk: site 16.5, *Gillespie et al. 12215* (CAN 10121774, CHARS, npsp); site 16.6, *Gillespie et al. 12219* (CAN 10121776, MT). **Clyde Area.** Clyde River: site CR-1, *Martin 3* (DAO 01-01667465, DAO 01-01667466), *Polunin 647* (GH01837336), *2602* (CAN 10050995); site CR-2, *Forbes 116* (DAO 01-01667468), *126* (DAO 01-01667467). Inugsuin Mouth: site IM-1, *Hainault 3838* (CAN 10050996, DAO 01-01667469, QK 59650). Kangiqtugaapik Head: site KP-1, *Dansereau 500720-0661* (MT - no acc. num.), *500804-0252* (MT00286612), *500822-0354* (MT00286611); site KP-6, *Wynne-Edwards 9025* (CAN 10051000); site KP-18, *Wynne-Edwards 9050* (CAN 10050998); site KP-20, *Wynne-Edwards 8988* (CAN 10050999). **Home Bay.** Cape Hooper: site HO-1, *Elven 3485/99* (CAN 10050980), *3514/99* (O V548721). Rocknoser Fiord: site RF-1, *Smith VP-66-61* (CAN 10050986).

##### *Ranunculus sabinei* R.Br. (Fig. 13F) —Sabine’s buttercup—Asian (N)–amphi-Beringian–North American (N)

This primarily high Arctic and western Arctic Island species is known on Baffin Island only from these three cited collections from two sites, about 10 km apart. Richardson and Webber’s Ekalugad collection was on coarse sand on a very gradual north-facing slope at 15 m elevation; Oswald’s was collected on a substrate of sand, clay, and stones on a mesic-wet 0–5° slope. The Kangirlugag collection was from a wet clay slope at 10 m elevation. Aiken et al. (2007) mapped the *Richardson & Webber 32* collection. These collections are highly disjunct by almost 1000 km from the species’ main range in Canada: ca. 990 km E of the nearest locality on the Boothia Peninsula (*Laverdière 19*CAN) and ca. 1000 km SSE of localities on southern Ellesmere Island (e.g., Goose Fiord, *Simmons 3787*GH 01837898). Aiken et al. (2007) incorrectly mapped a closer locality on the Melville Peninsula (ca. 670 km W of the Home Bay localities) based on a mapping error of *Manning 154*CAN 223437 from Cape Crozier on Banks Island, not Cape Crozier on Melville Island. Localities on Greenland are equally distant (e.g., *Ekblaw 357*CAN 10116815 from Etoh in northwest Greenland, ca. 1060 km).

**Specimens examined: Home Bay.** Ekalugad FOX-C: site FC-1, *Richardson & Webber 32* (CAN 10052157, COLO 02407302, QK 71108); site FC-2, *Oswald 364* (QK 135602). Kangirlugag Fiord: site KG-1, *Webber 1230* (CAN 10052156).

##### *Ranunculus sulphureus* Sol. (Fig. 9C)—Sulphur buttercup—Circumpolar-alpine

This species is recorded in the flora area from a single collection that was previously mapped in Aiken et al. (2007) and Porsild and Cody (1980). Identification of this specimen was confirmed based on its shallowly lobed to crenate-margined leaf blades; ovaries are too tightly packed on this flowering specimen to determine if hairs are present on the receptacle. Elsewhere on Baffin Island, it is known from the northern, southern, and south-central parts, including just southeast of the flora area in Auyiittuq National Park (*Ironside s.n.*CAN 10052018). Also recorded from Cape Adair and Scott’s Bay (Taylor 1863), but no collections located for verification.

**Specimens examined: Clyde Area.** Clyde River: site CR-1, *Martin 2* (DAO 01-01667470).

##### *Ranunculus trichophyllus* Chaix (=Ranunculus aquatilis var. diffusus With.)—Thread-leaved water-crowfoot—Subcosmopolitan

This species is newly reported for the flora area. Here we follow Wiegleb et al. (2017) for the taxonomy of aquatic buttercups (R. sect. Batrachium DC.). The single collection from the flora area is a small sterile specimen with flaccid leaves. Wiegleb et al. (2017) considered 2 species to occur in the Canadian Arctic. *Ranuculus trichophyllus* is the only one to have flaccid leaves; *R. codyanus* B. Boivin [included in a broader *R. subrigidus* W.B.Drew in Aiken et al. (2007)] has rigid leaves, as do the two species from the Canadian subarctic and boreal zone (*R. longirostis* Godr., *R. subrigidus*). Leaves of *R. codyanus* are also more densely hairy and smaller with shorter petioles (2–3(–5) mm long) than those of *R. trichophyllus* (leaves glabrous or hairy, petioles 5–20 mm) (Wiegleb et al. 2017). The specimen examined here has glabrous to sparsely hairy leaves with petioles 8 mm long. *Ranunculus trichophyllus* is widespread around the world in temperate, boreal, and subarctic regions; in the Canadian Arctic islands, it is found elsewhere only on southern Baffin Island (Saarela et al. 2020a; Saarela et al. 2023; collections at CAN). The collection cited here from Kangiqtugaapik Head is the northernmost record in eastern Canada by over 500 km and among the northernmost in Canada. *Ranunculus codyanus*, on the other hand, is considered to be an amphi-Beringian-North American Arctic species (Wiegleb et al. 2017) that is found scattered across the Canadian Arctic islands.

**Specimens examined: Clyde Area.** Kangiqtugaapik Head: site KP-1, *Dansereau 500805-0685* (MT00273066).

#### ﻿Rosaceae

***Dryas* L.**

##### Dryas integrifolia Vahl subsp. integrifolia—Entire-leaved mountain avens—Amphi-Beringian–North American (N)

Also recorded from Icy Arm, Quernbiter Fiord (https://www.inaturalist.org/observations/20079587) in the Buchan Gulf area.

**Specimens examined: Agguttinni TP.** Atagulisaktalik: site 22.2, *Gillespie et al. 11488* (CAN 10116693, CHARS, npsp). Clark Fiord: site 20.1, *Gillespie et al. 12338* (CAN 10116684). Gibbs Fiord: site 20.9, *Gillespie et al. 12380* (CAN 10116691). Kangiqtualuk Agguqti: site 3.5, *Gillespie et al. 11844* (CAN 10116686); site 8.2, *Gillespie et al. 11944* (CAN 10116687); site 21.8, *Gillespie et al. 12433* (CAN 10116692, npsp). Kangiqtualuk Uqquqti: site 27.1, *Gillespie et al. 11610* (CAN 10116685, MT). Refuge Harbour: site 12.1, *Gillespie et al. 12036* (CAN 10116688). Stewart Valley: site 15.1, *Gillespie et al. 12115* (CAN 10116689). Tingijattut: site 15.4, *Gillespie et al. 12153* (CAN 10116690); site TG-2, *Dare et al. 88* (CAN 10092777). **Clyde Area.** Clyde River: site CR-1, *Dutilly 9359* (QFA0141801), *Malte s.n..* [119072] (CAN 10062446, GH barcode-01588284), *Martin 8* (DAO 01-01292299), *Polunin 641* (CAN 10062432); site CR-2, *Forbes 15* (DAO 01-01294394), *61* (DAO 01-01292301), *129* (DAO 01-01292300). Hoffman Cove: site HF-1, *Bartlett 233* (US 03749654). Inugsuin Head: site IG-1, *Hainault 4068* (CAN 10062452, DAO 01-01292264); site IG-2, *Hainault 3636* (DAO 01-01292265, QK 59693); site IG-6, *Parmelee & Seaborn 3834* (DAO 01-01292306); site IG-9, *Parmelee & Seaborn 3937a* (DAO 01-01292307). Kangiqtugaapik Head: site KP-1, *Dansereau 500604-0258* [pro parte, plants 2] (MT00287251), *500607-2551* (MT00204015), *500607-2752* (MT00204030), *500607-2853* (MT00203984), *500607-2954* (MT00203987), *500607-3255* (MT00203989), *500607-3356* (MT00203990), *500607-3457* (MT00203986), *500624-0151* (MT00203988), *500627-3258* (MT00203985), *500701-0960* (MT00204034), *500702-0559* (MT00204037), *500713-0355* (MT00204031), *500713-1861* (MT00204036), *500716-0857* (MT00204035), *500726-1481* (MT00204032)3804, *500827-1165* (MT00204038), *Wynne-Edwards 8870* (CAN 10062418), *8910* (CAN 10062866); site KP-6, *Dansereau 500804-0454* (MT00204033); site KP-8, *Wynne-Edwards 8849* (CAN 10062441); site KP-17, *Dansereau 500623-1853* (MT00204029); site KP-18, *Wynne-Edwards 8822* (CAN 10062437), *8827* (CAN 10062431). **Coutts Inlet.** Coutts Fiord: site CF-1, *Coombs 45* (DAO 01-01292225). Coutts Mouth: site CO-1, *Coombs 69* (DAO 01-01294395). **Home Bay.** Cape Hooper: site HO-1, *Elven 3436/99* (ALA V132468, CAN 10061799, O V549977); site HO-3, *Parmelee & Seaborn 3971* (DAO 01-01294397). Ekalugad FOX-C: site FC-1, *Crompton et al. s.n.* (COLO 01107390), *Richardson & Webber 5* (CAN 10062455, COLO 01107408); site FC-2, *Oswald 346* (QK 135614). Kangirlugag Fiord: site KG-1, *Webber 1238* (CAN 10062430, COLO 01107598). Rocknoser Fiord: site RF-1, *Smith VP-17-61* (CAN 10062439).

#### ﻿*Potentilla* L.

*Potentilla* is a large and taxonomically complex genus, with frequent hybridization and numerous stabilized hybrids. Here we follow the taxonomy in Ertter et al. (2014).

##### Key to species and subspecies of *Potentilla* [adapted from Ertter et al. (2014)]

**Table:** 

1	Leaves pinnate (sect. Pensylvanicae)	***P. pulchella***
–	Leaves ternate or palmate	**2**
2	Leaflets pale to dark green abaxially, cottony and crisped hairs absent (*P. hyparctica*, sect. Aureae) 3
–	Leaflets white to gray abaxially, cottony and/or crisped (short, twisted) hairs abundant to dense	**4**
3	Epicalyx bractlets narrowly ovate or elliptic, 1.5–2 mm wide; central leaflets: petiolules 0–2 mm long, blade bases cuneate	**P. hyparctica subsp. hyparctica**
–	Epicalyx bractlets broadly oblong or ovate, 2–5 mm wide; central leaflets: petiolules (0–)2–3(–5) mm long, blade bases broadly cuneate to rounded	**P. hyparctica subsp. elatior**
4	Leaflets 3–5, usually more than 3 on at least some leaves (sect. Rubricaules)	***P. pedersenii***
–	Leaflets 3, rarely more, on all basal leaves (sect. Niveae)	**5**
5	Epicalyx bractlets ≤ 1/2 as wide as sepals; petiole indumentum either primarily of cottony hairs or of ± stiff verrucose hairs (appearing white, opaque); inflorescences usually more than 1-flowered; central leaflet blades incised 1/3–1/2 to midvein in distal 3/4, teeth (2–)3–5(–6) per side	**6**
–	Epicalyx bractlets (1/2–)2/3 to as wide as sepals; petiole indumentum primarily of soft to weak smooth hairs; inflorescences often only 1-flowered; central leaflet blades incised (1/3–)1/2–3/4 to midvein in distal half, teeth (1–)2–3(–4) per side	**8**
6	Petioles: long hairs usually absent, sometimes sparse to common, usually soft, usually ± appressed, smooth, cottony hairs usually abundant to dense; central leaflets subsessile or short-petiolulate	***P. nivea***
–	Petioles: long hairs sparse to abundant, usually stiff, spreading to ± ascending, verrucose, cottony hairs absent; central leaflets usually petiolulate (to 5 mm long) (*P. arenosa*)	**7**
7	Petioles with both long verrucose hairs and common to abundant short and/or stiff crisped hairs	**P. arenosa subsp. arenosa**
–	Petioles usually only with long verrucose hairs, short or soft crisped hairs usually absent, sometimes sparse	**P. arenosa subsp. chamissonis**
8	Petioles: crisped/short-cottony hairs usually absent, sometimes sparse, long hairs ± weak, rarely stiff; plants usually cushion-forming; basal leaves 0.5–2.5(–3) cm	***P. subvahliana***
–	Petioles: crisped/short-cottony hairs usually sparse to abundant, long hairs soft to weak; plants ± densely tufted to cushion-forming; basal leaves 1–10(–15) cm	**9**
9	Epicalyx bractlets elliptic-lanceolate to ovate, (3–)4–6(–7) × (0.8–)1.2–2(–2.5) mm, (1/2–)2/3 to as wide as sepals; sepals 4–6(–7) mm long; petals (5–)6–9 mm long, 1.5–1.8 × sepal length	***P. subgorodkovii***
–	Epicalyx bractlets broadly ovate, 2.5–4(–5) × 1.5–3 mm, ± as wide as sepals; sepals 2.5–5(–6) mm long; petals 8–10 mm long, 1.8–2.1 × sepal length	***P. vahliana***

##### Potentilla arenosa (Turcz.) Juz. subsp. arenosa—Bluff cinquefoil—Asian (N/C)–amphi-Beringian–North American (N)

This taxon is newly recorded for the flora area and for Baffin Island here and in Gillespie et al. (2024). This subspecies is known in the Canadian Arctic islands from the western and high Arctic, whereas subsp. chamissonis is mostly found in the southeast (Elven et al. 2011). Largely allopatric (Ertter et al. 2014), the two *P. arenosa* subspecies overlap in the flora area, where both are recorded only from the heads of large fiords.

**Specimens examined: Agguttinni TP.** Kangiqtualuk Agguqti: site 6.3, *Gillespie et al. 11891* (CAN 10116664, npsp). Kangiqtualuk Uqquqti: site 29.2, *Gillespie et al. 11693* (CAN 10116613, MT, npsp); site 29.4, *Gillespie et al. 11705* (ALA, CAN 10116615, CHARS, MT, npsp). **Clyde Area.** Inugsuin Head: site IG-30, *Hainault 4017* (CAN 10063010, DAO 01-01289200, QK 59723). Kangiqtugaapik Head: site KP-1, *Dansereau 500703-0365* (MT00287123, MT00287125), *500707-0954* (MT00287117, MT00287119, MT00287121), *500710-0363* (MT00287131), *500726-1171* (MT00287134), *500731-0564* (MT00287135), *500803-0474* [pro parte, plants A,B] (MT00287057), *500823-0782* (MT00287132), *500823-0984* (MT00287137), *Wynne-Edwards 8847* (CAN 10063012), *8854* (CAN 10063011); site KP-18, *Wynne-Edwards 8823* (CAN 10063013 [pro parte, plants A]).

##### Potentilla arenosa subsp. chamissonis (Hultén) Elven & D.F.Murray (≡*Potentilla chamissonis* Hultén; ≡Potentilla nivea subsp. chamissonis (Hultén) Hiitonen)—Chamisso’s cinquefoil—North American (NE)–amphi-Atlantic–European (N)–Asian (NW)

**Specimens examined: Agguttinni TP.** Kangiqtualuk Agguqti: site 21.5, *Gillespie et al. 12427* (ALA, ALTA, CAN 10116614, MT, npsp). **Clyde Area.** Kangiqtugaapik Head: site KP-1, *Dansereau 500627-3156* (MT00287072), *Wynne-Edwards 8841* (CAN 10064010).

##### Potentilla hyparctica Malte subsp. hyparctica (=*P. emarginata* Pursh) (Fig. 10C)—Arctic cinquefoil—Circumpolar

**Specimens examined: Agguttinni TP.** Atagulisaktalik: site 22.1, *Gillespie et al. 11461* (ALA, CAN 10116627, CHARS, MT, npsp); site AT-1, *Dansereau 500722-0259* (MT00287257, MT00287258), *500722-0388* (MT00287259). Generator Lake: site 31.5, *Gillespie et al. 11764* (ALA, CAN 10116617); site GL-22, *Raynolds & Bültmann MKR-2022-47* (CAN 10169252). Kangiqtualuk Agguqti: site 3.1, *Gillespie et al. 11792* (CAN 10116625, npsp); site 5.3, *Gillespie et al. 11861* (CAN 10116624, MT). Kangiqtualuk Uqquqti: site 28.4, *Gillespie et al. 11662* (CAN 10116620). Kuugaaluk: site 16.5, *Gillespie et al. 12213* (ALTA, CAN 10116623, MO, WIN); site 19.1, *Gillespie et al. 12288* (CAN 10116619); site 19.3, *Gillespie et al. 12299* (CAN 10116618, MT). Marble Lake: site 31.1, *Gillespie et al. 11739* (ALTA, CAN 10116622, MO, US). Ravenscraig Harbour: site RH-1, *Dare 33* (CAN 10092684), *35* (CAN 10092687); site 11.2, *Gillespie et al. 12023* (CAN 10116626); site 11.3, *Gillespie et al. 12027* (CAN 10116616). Tingijattut: site 15.6, *Gillespie et al. 12170* (ALA, CAN 10116621). **Clyde Area.** Cape Hewett: site CH-1, *Platt 480A* (NY 2830553). Clyde River: site CR-1, *Dutilly 1457* (CAN 10063209, CM520285), *Malte s.n..* [119075] (CAN 10063233), *Malte s.n..* [119106] (CAN 10063215), *Martin 9* (DAO 01-01284793), *Polunin 610* (CAN 10063402), *615* (CAN 10063234); site CR-2, *Forbes 6* (DAO 01-01285121), *48* (DAO 01-01285171), *97* (DAO 01-01285156); site CR-3, *Dare 50* (CAN 10092780). Hewett Cache: site HC-1, *Hainault 3800* (QK 60110), *3804* (QK 60111). Inugsuin Head: site IG-2, *Hainault 3638* (CAN 10063213, DAO 01-01284800, QK 59569); site IG-5, *Parmelee & Seaborn 3964* (DAO 01-01284808). Inugsuin Mouth: site IM-1, *Hainault 3818* (QK 59671), *3886* (DAO 01-01273220, QK 60109), *3904* (QK 59810), *3931* (CAN 10063211, QK 59558), *3950* (DAO 01-01273221). Kangiqtugaapik Head: site KP-1, *Dansereau 500625-0352* (MT00287256, MT00287253), *500628-3151* (MT00287252), *500703-0454* (MT00287255), *500705-0163* (MT00287254), *500705-0351* (MT00287236), *500713-1166* (MT00287231), *500715-0392* (MT00287230), *500719-0255* (MT00287234), *500720-0763* (MT00287229), *500724-0781* (MT00287232, MT00287233, MT00287235), *500803-0154* (MT00287228), *500804-0656* (MT00287227), *500822-0462* (MT00287238), *Wynne-Edwards 8855* (CAN 10063231); site KP-3, *Wynne-Edwards 8939* (CAN 10063237); site KP-4, *Wynne-Edwards 8819* (CAN 10063232); site KP-20, *Wynne-Edwards 8867* (CAN 10063226); site KP-24, *Wynne-Edwards 8828* (CAN 10063225); site KP-27, *Dansereau 500716-0667* (MT00287237). McBeth Valley: site MB-1, *Hainault 3773* (DAO 01-01284802, QK 59667). **Coutts Inlet.** Coutts Fiord: site CF-1, *Coombs 97* (DAO 01-01285122). Coutts Mouth: site CO-1, *Coombs 95* (DAO 01-01285125). **Home Bay.** Cape Hooper: site HO-1, *Elven 3447/99* (ALA V132379, CAN 10063389); site HO-3, *Parmelee & Seaborn 3788* (DAO 01-01284813). Ekalugad FOX-C: site FC-1, *Richardson & Webber 14a* (CAN 10063217, COLO 01126309, QK 71145), *71* (CAN 10063214, COLO 01126325); site FC-2, *Oswald 391* (QK 135608). Ekalugad Head: site EK-1, *Philpot et al. s.n.* (COLO 01126267); site EK-3, *Philpot et al. s.n.* (COLO 01126358). Kangirlugag Fiord: site KG-1, *Webber 1214* (CAN 10063216, COLO 01126671), *1280* (COLO 01126291), *1313* (COLO 01126317). Kangok Fiord: site KO-1, *Crompton et al. s.n.* (COLO 01126275). Rocknoser Fiord: site RF-1, *Smith VP-27-61* (CAN 10063229), *VP-3-61* (CAN 10063228), *Webber 1199* (COLO 01126689).

##### *Potentilla nivea* L.—Snow cinquefoil—Circumpolar-alpine

**Specimens examined: Agguttinni TP.** Tasialuk S: site 26.5, *Gillespie et al. 11591* (CAN 10116663, npsp). **Clyde Area.** Inugsuin Head: site IG-1, *Hainault 4040* (DAO 01-01273568 [pro parte, plants b], QK 59763 [pro parte, plants a]). Kangiqtugaapik Head: site KP-16, *Wynne-Edwards 8976* [cf.] (CAN 10064391); site KP-18, *Wynne-Edwards 8823* (CAN 10063013 [pro parte, plants B]).

##### *Potentilla pedersenii* Rydb.—Pedersen’s cinquefoil—European (N)–Asian (N)–amphi-Beringian–North American (N)

This species is newly reported for the flora area here and in Gillespie et al. (2024). It is considered to be a stabilized intersectional hybrid, putatively between *P. pulchella* (sect. Pensylvanicae) and P. arenosa subsp. arenosa (sect. Niveae) (Ertter et al. 2014). Considerable variation within the species suggests it evolved through multiple hybridization events (Ertter et al. 2014). The species is a member of section Rubricaules (Rydb.) A. Nelson, a group of intersectional hybrids characterized by at least some leaves having five leaflets. Many of the cited collections were previously determined to be *P. rubricaulis* Lehm., a species now considered restricted to subarctic western North America.

**Specimens examined: Clyde Area.** Inugsuin Head: site IG-1, *Hainault 3704* (CAN 10087383, O V554935, QK 59647), *3718* (CAN 10064489, O V554934, QK 59647); site IG-24, *Hainault 3643* (CAN 10064491, DAO 01-01289202, O V554937, QK 59574). Kangiqtugaapik Head: site KP-1, *Dansereau 500709-0557* (MT00273062, MT00273068), *500709-0661* (MT00273063a, MT00273063b), *500710-3976* (MT00273067), *500718-0652* (MT00273065), *500718-0753* (MT00273064); site KP-5, *Wynne-Edwards 9058* (CAN 10064497).

##### *Potentilla pulchella* R.Br.—Pretty cinquefoil—Circumpolar

**Specimens examined: Agguttinni TP.** Kangiqtualuk Agguqti: site 8.1, *Gillespie et al. 11904* (CAN 10116665). **Clyde Area.** Inugsuin Head: site IG-1, *Hainault 3694* (CAN 10069724, DAO 01-01293100, O V555020, QK 59715); site IG-8, *Parmelee & Seaborn 3950* (DAO 01-01293378). Kangiqtugaapik Head: site KP-1, *Dansereau 500604-0258* [pro parte] (MT00287250, MT00287071), *500628-3261* (MT00287070, MT00287248), *500730-1194* (MT00287246), *500801-0665* (MT00287245). *Wynne-Edwards 8852* (CAN 10069725); site KP-2, *Martin 59* (DAO 01-01293367); site KP-5, *Wynne-Edwards 9057* (CAN 10069726); site KP-15, *Dansereau 500713-0874* [pro parte, plants C,D] (MT00287065); site KP-22, *Dansereau 500710-3751bis* (MT00287249), *500710-3761* (MT00287247). **Home Bay.** Ekalugad FOX-C: site FC-1, *Richardson & Webber 14b* (COLO 01130186); site FC-2, *Oswald 359* (QK 135587), *408* (QK 135682).

##### *Potentilla subgorodkovii* Jurtzev—Sheenjek River cinquefoil—Amphi-Beringian–North American (N)

This is a presumed stabilized hybrid species or collective entity for plants combining characteristics of species belonging to the two main species groups within section Nivea, the nivea group (including *P. nivea*, *P. arenosa*) and the uniflora/villosa group (including *P. subvahliana*) (Ertter et al. 2014). Its exact status and geographic distribution are not fully known. The cited specimens match the concept of *P. subgorodkovii* (Ertter et al. 2014) more than they do *P. vahliana* or *P. subvahliana*. Plants are tufted, not cushion-forming, and not densely marcescent columnar-sheathed (versus cushion-forming and densely marcescent columnar-sheathed in *P. subvahliana*), stems are 8–15 cm long (versus < 8 (11) in the other two species), and epicalyx bractlets are lanceolate to narrowly ovate and < 3/4 width of sepals (versus ovate and same width as sepals in *P. vahliana*). Plants have (1) 2–3 flowers per inflorescence, more like *P. vahliana* but within the range of *P. subgorodkovii* (versus 1 (2) in *P. subvahliana*). Petioles have sparse short crisped/cottony hairs, characteristic of *P. subgorodkovii* (versus hairs infrequent in *P. subvahliana* and usually dense in *P. vahliana*). In North America *P. subgorodkovii* is mostly found in the western Arctic and has not previously been reported so far east (Elven et al. 2011). It is newly reported for the flora area here and in Gillespie et al. (2024) and is the first record for Baffin Island.

**Specimens examined: Agguttinni TP.** Kangiqtualuk Agguqti: site 21.1, *Gillespie et al. 12413* (CAN 10116563, npsp); site 21.8, *Gillespie et al. 12432* (ALA, CAN 10116562); site 8.2, *Gillespie et al. 11936* (CAN 10116564, MT). **Clyde Area.** Inugsuin Head: site IG-2, *Hainault 3631* (CAN 10087382, DAO 01-01289201, O V554936, QK 59597). Kangiqtugaapik Head: site KP-1, *Dansereau 500628-3262* (MT00287138), *500710-0152* [pro parte] (MT00287140), *500803-0481* (MT00287141).

##### *Potentilla subvahliana* Jurtzev—High Arctic cinquefoil—Amphi-Beringian–North American (N)

This species is newly recognized for the flora area. Previous Canadian Arctic floras did not distinguish between this species and *P. vahliana* (and *P. subgorodkovii*), referring all collections to *P. vahliana* (Polunin 1940; Porsild 1957; Porsild and Cody 1980; Aiken et al. 2007).

**Specimens examined: Agguttinni TP.** Clark Fiord: site 20.7, *Gillespie et al. 12370b* [cf.] (CAN 10116741). Gee Lake: site GE-1, *Dansereau 500811-0854* (MT00287115). Gibbs esker: site 20.12, *Gillespie et al. 12398* (CAN 10116745), *12401* (CAN 10116746). Kangiqtualuk Agguqti: site 3.5, *Gillespie et al. 11846* (ALA, CAN 10116743, CHARS); site 8.2, *Gillespie et al. 11947* (CAN 10116744). Kangiqtualuk Uqquqti: site 30.3, *Gillespie et al. 11716* [cf.] (CAN 10116740, MT). Marble Lake: site 31.2, *Gillespie et al. 11743* (CAN 10116742, MT, npsp). **Clyde Area.** Inugsuin Head: site IG-1, *Hainault 3697* (DAO 01-01282742, QK 59534); site IG-6, *Parmelee & Seaborn 3852* (DAO 01-01289242); site IG-9, *Parmelee & Seaborn 3935* (DAO 01-01289241). Kangiqtugaapik Head: site KP-1, *Dansereau 500701-1057* (MT00287114); site KP-20, *Wynne-Edwards 8866* (CAN 10063382), *500731-0563* [pro parte, plants 2] (MT00287069). McBeth Valley: site MB-1, *Hainault 3760* (DAO 01-01289238, MT00287077, O V555088, QK 59660).

##### *Potentilla vahliana* Lehm.—Vahl’s cinquefoil—North American (NE)

*Potentilla vahliana* is considered a geographically restricted stabilized hybrid species, likely formed from crosses between *P. nivea* and *P. subvahliana* (Ertter et al. 2014). Taylor (1863) recorded the species from Scott’s Bay, but no collection was located; his record could refer to either *P. subvahliana* or *P. vahliana* but is likely the latter based on the localities.

**Specimens examined: Agguttinni TP.** Atagulisaktalik: site 23.6, *Gillespie et al. 11521* (CAN 10121697, npsp); site AT-1, *Dansereau 500722-0388* (MT00287224). Clark Fiord: site 20.1, *Gillespie et al. 12339* (ALA, CAN 10121702); site 20.7, *Gillespie et al. 12370a* (CAN 10121693, MO); site 20.9, *Gillespie et al. 12377* (CAN 10121694, npsp), *12382* (CAN 10121703, MO). Kangiqtualuk Uqquqti: site 28.4, *Gillespie et al. 11658* (CAN 10121695, CHARS, MT). Stewart Valley: site 15.1, *Gillespie et al. 12122* (CAN 10121698). Tasialuk S: site 26.5, *Gillespie et al. 11588* (CAN 10121696). Tingijattut: site 15.2, *Gillespie et al. 12131* (ALTA, CAN 10121699); site 15.4, *Gillespie et al. 12154* (CAN 10121700), *12164* (CAN 10121701). **Clyde Area.** Hoffman Cove: site HF-1, *Bartlett 234* (US 03741706). Inugsuin Head: site IG-1, *Hainault 3673* (CAN 10063683, DAO 01-01289239, QK 59691); site IG-5, *Parmelee & Seaborn 3965* (DAO 01-01289229); site IG-6, *Parmelee & Seaborn 3853* (DAO 01-01289240). Inugsuin Mouth: site IM-1, *Hainault 3878* (CAN 10063693, DAO 01-01289302, O V555087, QK 59776). Kangiqtugaapik Head: site KP-1, *Dansereau 500604-0563* (MT00287074), *500629-2190* (MT00287212), *500710-2271* (MT00287222), *500713-0151* (MT00287073), *500725-0767* (MT00287211), *500731-0563* [pro parte] (MT00287068, MT00287226), *500731-0564A* (MT00287215), *500803-0474* [pro parte, plants C] (MT00287060), *500822-0353* [cf.] (MT00287075), *500823-0683* (MT00287076); site KP-8, *Dansereau 500705-0352* (MT00287210), *500715-0593* (MT00287225); site KP-15, *Dansereau 500713-0874* [pro parte, plants A,B] (MT00287063); site KP-24, *Wynne-Edwards 8842* (CAN 10063677); site KP-27, *Dansereau 500716-0569* (MT00287217), *500716-0868* (MT00287221), *500716-0868A* (MT00287220). **Coutts Inlet.** Coutts Fiord: site CF-1, *Coombs 96* (DAO 01-01289244). **Home Bay.** Cape Hooper: site HO-1, *Elven 3475/99* (ALA V132388, CAN 10063674, O V555090), *Molau 3628/99* (O V555089). Ekalugad FOX-C: site FC-1, *Philpot et al. s.n.* (COLO 01130178), *Richardson & Webber 35* (CAN 10063675, COLO 01130210, QK 71146). Ekalugad Head: site EK-3, *Philpot et al. s.n.* (COLO 01130269). Kangirlugag Fiord: site KG-1, *Webber 1205* (COLO 01130194). Kangok Fiord: site KO-1, *Philpot et al. s.n.* (COLO 01130251). Rocknoser Fiord: site RF-1, *Smith VP-14-61* (CAN 10063676), *VP-26-61* (CAN 10063691), *VP-50-61* (CAN 10063680), *VP-64-61* (CAN 10063692), *Webber 1201* (COLO 01130202).

##### Potentilla arenosa × vahliana

**Specimens examined: Clyde Area.** Inugsuin Head: Site IG-1, *Hainault 4040* (DAO 01-01273568 [pro parte, plants a], QK 59763 [pro parte, plants b]). Kangiqtugaapik Head: site KP-1, *Dansereau 500703-0453* [cf.] (MT00287126), *500710-0152* [pro parte] [cf.] (MT00287127), *500730-0955* [cf.] (MT00287128).

##### Potentilla × prostrata Rottb.—Prostrate cinquefoil—Amphi-Atlantic

This hybrid species is a cross between *P. arenosa* and *P. nivea* (Elven et al. 2014) and is characterized by cottony hairs on the petioles and stems as in *P. nivea* and the presence of verrucose hairs as in *P. arenosa*.

**Specimens examined: Clyde Area.** Inugsuin Head: site IG-1, *Hainault 3705* (DAO 01-01269246, QK 59648); site IG-6, *Parmelee & Seaborn 3867* (DAO 01-01269247). Kangiqtugaapik Head: site KP-5, *Wynne-Edwards 9056* (CAN 10087385); site KP-16, *Wynne-Edwards 8975* (CAN 10089154).

#### ﻿Salicaceae

***Salix* L.**

##### Key to species of *Salix* [adapted from Argus (2010)]

**Table:** 

1	Shrubs, not dwarf, 0.2–2+ m tall; ovaries and capsules glabrous	***S. richardsonii***
–	Dwarf shrubs, 0.01–0.2 m tall; ovaries and capsules glabrous or hairy	**2**
2	Ovaries and capsules glabrous; catkins 0.3–1.2 cm long, few-flowered, subglobose or ellipsoid; leaf blades 0.5–1.5 cm long	***S. herbacea***
–	Ovaries and capsules hairy; catkins 1–10+ cm long, usually many-flowered, cylindrical; leaf blades 1.5–8 cm long	**3**
3	Leaf blades prominently reticulate-veined, appearing bullate; catkins from subterminal buds; plants clonal by layering	***S. reticulata***
–	Leaf blades not prominently reticulate-veined, not bullate; catkins from lateral buds; plants not clonal	**4**
4	Leaf blades hairy abaxially (entire surface or only on midrib or near apex), margins entire; young branches hairy; ovary hairs not crinkled, not refractive (appearing white)	***S. arctica***
–	Leaf blades glabrous, margins usually closely and prominently serrulate or crenulate, rarely entire; young branches glabrous; ovary hairs usually crinkled and refractive (appearing shiny)	***S. arctophila***

##### *Salix arctica* Pall. (Fig. 3F, 10D)—Arctic willow—Circumpolar-alpine

This is the most common willow in the flora area and throughout the Canadian Arctic Archipelago. See below for hybrid with *S. arctophila*. Also recorded from Feachem Bay, Buchan Gulf (https://www.inaturalist.org/observations/31842189).

**Specimens examined: Agguttinni TP.** Atagulisaktalik: site 22.1, *Gillespie et al. 11469* (CAN 10116594, CHARS, MT). Clark Fiord: site 20.1, *Gillespie et al. 12327* (CAN 10116600); site 20.3, *Gillespie et al. 12362* (CAN 10116585, MO). Generator Lake: site GL-20, *Raynolds & Bültmann MKR-2022-45* (CAN 10169259). Gibbs Fiord: site 20.9, *Gillespie et al. 12379* (ALTA, CAN 10123191). Kangiqtualuk Agguqti: site 3.2, *Gillespie et al. 11830* (CAN 10116595, MT), *11831* (CAN 10116596, MT); site 6.2, *Gillespie et al. 11880* (ALTA, CAN 10116597, CHARS); site 8.1, *Gillespie et al. 11907* (CAN 10116598), *11912* (ALA, CAN 10116589). Kangiqtualuk Uqquqti: site 28.2, *Gillespie et al. 11645* (CAN 10116592, npsp), *11646* (CAN 10116593, npsp, US). Ravenscraig Harbour: site RH-1, *Dare 6* (CAN 10092817), *9* (CAN 10092818), *17* (CAN 10092816), *18* (CAN 10092819). Refuge Harbour: site 11.1, *Gillespie et al. 12011* (CAN 10116590); site 11.3, *Gillespie et al. 12028* (CAN 10116599). Stewart Valley: site 15.1, *Gillespie et al. 12114* (CAN 10116586). Tasialuk N: site 26.1, *Gillespie et al. 11575* (CAN 10116591, MO). Tingijattut: site 15.2, *Gillespie et al. 12133* (CAN 10116587, MO); site TG-2, *Dare et al. 87* (CAN 10092824, US). **Clyde Area.** Cape Hewett: site CH-1, *Platt 484A* (NY 2404864). Clyde River: site CR-1, *Malte s.n..* [118619] (CAN 10001981), *Martin 29* (DAO 01-01000684711), *30* (DAO 01-01684706), *Polunin 588* (CAN 10002007), *1348* (US 03423323); site CR-2, *Forbes 3* (DAO 01-01360760), *27* (DAO 01-01339675), *53* (DAO 01-01360783), *99* (DAO 01-01360738); site CR-5, *Martin 61* (DAO 01-01000684696). Hoffman Cove: site HF-1, *Bartlett 230* (US 03422946). Inugsuin Head: site IG-1, *Hainault 3639* (CAN 10001983, CAN 10002018, O V547708, QK 59570), *3698* (ACAD 70019, CAN 10001984, O V547707, QK 59720); site IG-6, *Parmelee & Seaborn 3845* (DAO 01-01361107); site IG-24, *Hainault 3723* (ACAD 70018, O V547703, QK 59607). Inugsuin Mouth: site IM-1, *Hainault 3815* (CAN 10002016, O V547704, QK 59564), *3903* (CAN 10001977, CAN 10002020, O V547783, QK 59808). Kangiqtugaapik Head: site KP-1, *Dansereau 500723-0773* (MT00285736), *500726-1583* (MT00285737), *Wynne-Edwards 8816* (CAN 10001879), *8816-A* (CAN 10001938), *8961* (CAN 10001876); site KP-22, *Dansereau 500710-3875* (MT00286649); site KP-24, *Wynne-Edwards 8835* (CAN 10001944); site KP-26, *Dansereau 500731-0752* (MT00286648). McBeth Valley: site MB-1, *Hainault 3781* (CAN 10002017, O V547706, QK 59630), *3790* (CAN 10002009, O V547705, QK 59621). **Coutts Inlet.** Coutts Fiord: site CF-1, *Coombs 60* (DAO 01-01000684715), *61* (DAO 01-01000684713). Coutts Mouth: site CO-1, *Coombs 90* (DAO 01-01000684705), *91* (DAO 01-01000684712). **Home Bay.** Cape Hooper: site HO-1, *Elven 3448/99* (CAN 10002006, O V547721), *3473/99* (ALA V132461, CAN 10001995), *3473b/99* (ALA V132464, ALA V132457, CAN 10001985, O V547778), *3496/99* (O V547736); site HO-3, *Parmelee & Seaborn 3789* (DAO 01-01000684728). Ekalugad FOX-C: site FC-1, *Crompton et al. s.n.* (COLO 01735109), *Crompton et al. s.n.* (COLO 01735273), *Richardson & Webber 22* (CAN 10001953, QK 71095), *27* (COLO 01735257); site FC-2, *Oswald 348* (QK 135262), *351* (QK 135261), *369* (QK 135265); site FC-4, *Oswald 360* (QK 135259); site FC-6, *Oswald 356* (QK 135260). Kangirlugag Fiord: site KG-1, *Webber 1233* (COLO 01735281), *1254* (CAN 10001952, COLO 01735299), *1299* (COLO 01735307). Kangok Fiord: site KO-1, *Crompton et al. s.n.* (COLO 01735315).

##### *Salix arctophila* Cock. ex Heller—Northern willow—North American (N)

This species has a more southerly distribution in the Canadian Arctic compared to *S. arctica*, which is widespread and common throughout. Although not collected from the northern half of the flora area, the species is to be expected based on the presence of the hybrid *S. arctica* × *S. arctophila* (see below) in Agguttinni TP. The northernmost verified record in the eastern Canadian Arctic is from Pond Inlet (*Malte* CAN118713) on Baffin Island, just north of the flora area.

**Specimens examined: Clyde Area.** Inugsuin Head: site IG-3, *Parmelee & Seaborn 4009a* (DAO 01-01000684697); site IG-6, *Parmelee & Seaborn 3865* (DAO 01-01361102), *3864a* (DAO 01-01361103); site IG-13, *Parmelee & Seaborn 3906* (DAO 01-01000684716). **Home Bay.** Cape Hooper: site HO-1, *Elven 3505/99* (ALA V132470, O V547777), *3511/99* (CAN 10023432), *3571/99* (O V547776).

##### Salix arctica × arctophila

Argus (2010) described this hybrid as resembling *S. arctophila* in its toothed leaves and *S. arctica* in its hairy leaves and branchlets.

**Specimens examined: Agguttinni TP.** Kuugaaluk: site 16.1, *Gillespie et al. 12195* (CAN 10116588, npsp). **Clyde Area.** Inugsuin Mouth: site IM-1, *Hainault 3908* (CAN 10023674, O V547784, QK 59811). Kangiqtugaapik Head: site KP-1, *Wynne-Edwards 8961A* (CAN 10023196).

##### *Salix herbacea* L.—Snowbed willow—North American (NE)–amphi-Atlantic–European (N/C)

Also recorded from Feachem Bay, Buchan Gulf (https://www.inaturalist.org/observations/31842191).

**Specimens examined: Agguttinni TP.** Atagulisaktalik: site 22.1, *Gillespie et al. 11470* (CAN 10121677, npsp), *11471* (CAN 10116749, npsp). Clark Fiord: site 20.2, *Gillespie et al. 12359* (CAN 10121674). Generator Lake: site GL-20, *Raynolds & Bültmann MKR-2022-60* (CAN 10169257). Kangiqtualuk Agguqti: site 3.1, *Gillespie et al. 11797* (CAN 10121666), *11798* (CAN 10121667); site 8.8, *Gillespie et al. 11989* (CAN 10121668); site 21.1, *Gillespie et al. 12412* (CAN 10121675). Kangiqtualuk Uqquqti: site 28.3, *Gillespie et al. 11651* (CAN 10116750), *11652* (CAN 10121665). Kuugaaluk: site 16.2, *Gillespie et al. 12200* (CAN 10121673); site 16.5, *Gillespie et al. 12212* (CAN 10121670); site 16.6, *Gillespie et al. 12220* (CAN 10121671); site A22.3, *Gillespie et al. 12459* (CAN 10121676, MT). Ravenscraig Harbour: site RH-1, *Dare 7* (CAN 10092788), *25* (CAN 10092790). Refuge Harbour: site 11.4, *Gillespie et al. 12035* (CAN 10121669). Tingijattut: site 15.2, *Gillespie et al. 12129* (CAN 10121672). **Clyde Area.** Clyde River: site CR-1, *Martin 28* (DAO 01-01000684879), *Polunin 589 & 625* (CAN 10025633); site CR-2, *Forbes 37* (DAO 01-01000684), *54* (DAO 01-01000684684), *103* (DAO 01-01000684673). Inugsuin Head: site IG-1, *Hainault 3670* (CAN 10025568, CAN 10025608, O V547883, QK 59689); site IG-6, *Parmelee & Seaborn 3861* (DAO 01-01000684688). Kangiqtugaapik Head: site KP-1, *Dansereau 500628-5994* (MT00285741), *500629-2297* (MT00285740), *500703-0960* (MT00285738, MT00285739), *500713-1165* (MT00286644), *500713-1264* (MT00286645), *500713-1368* (MT00286646), *500803-0972* (MT00286647), *Wynne-Edwards 8846* (CAN 10025585), *8865* (CAN 10025570); site KP-13, *Wynne-Edwards 8896* (CAN 10025569). **Home Bay.** Cape Hooper: site HO-1, *Elven 3465/99* (ALA V132469, CAN 10025574, O V547879). Ekalugad FOX-C: site FC-1, *Crompton et al. s.n.* (COLO 01617836), *Richardson & Webber 6* (COLO 01617794), *Richardson & Webber 17* (CAN 10025595, COLO 01617802, QK 71094); site FC-2, *Oswald 366* (QK 135250), *464* (QK 135251). Kangirlugag Fiord: site KG-1, *Webber 1229a* (CAN 10025590, COLO 01617844). Rocknoser Fiord: site RF-1, *Smith VP-11-61* (CAN 10025634).

##### *Salix reticulata* L.—Net-veined willow—Circumpolar-alpine

Also recorded from Scott’s Bay (Taylor 1863), but no collection located, and Feachem Bay, Buchan Gulf (https://www.inaturalist.org/observations/37255415).

**Specimens examined: Agguttinni TP.** Atagulisaktalik: site 22.2, *Gillespie et al. 11489* (CAN 10121678, CHARS, MT, npsp), *11490* (ALTA, CAN 10121685, MT, npsp). Clark Fiord: site 20.2, *Gillespie et al. 12360* (CAN 10121691). Kangiqtualuk Agguqti: site 3.2, *Gillespie et al. 11818* (ALA, CAN 10121679), *11819* (ALA, CAN 10121680); site 8.2, *Gillespie et al. 11949a* (CAN 10121681), *11949b* (CAN 10121682); site 21.9, *Gillespie et al. 12434* (CAN 10121692, CHARS, QFA). Kangiqtualuk Uqquqti: site 28.6, *Gillespie et al. 11670* (CAN 10121686, MO), *11671* (CAN 10121687, MO). Refuge Harbour: site 12.5, *Gillespie et al. 12051* (CAN 10121690). Remote Peninsula: site RL-1, *Philpot et al. s.n.* (COLO 01625250). Tingijattut: site 15.2, *Gillespie et al. 12140* (CAN 10121683), *12141* (CAN 10121684); site TG-2, *Dare et al. 86* (CAN 10092823). **Clyde Area.** Clyde River: site 10.3, *Gillespie et al. 12000* (CAN 10121688), *12001* (CAN 10121689); site CR-1, *Malte s.n..* [118639] (CAN 10030310), *Martin 31* (DAO 01-01000684513); site CR-2, *Forbes 16* (DAO 01-01000684527), *106* (DAO 01-01000684528). Inugsuin Head: site IG-1, *Hainault 3703* (CAN 10030263, CAN 10030269, O V547958, QK 59646); site IG-6, *Parmelee & Seaborn 3859* (DAO 01-01000684491); site IG-7, *Parmelee & Seaborn 3931a* (DAO 01-01000684533); site IG-15, *Parmelee & Seaborn 3882a* (DAO 01-01000684525). Kangiqtugaapik Head: site KP-1, *Dansereau 500611-1553* (MT00285742), *500627-1961* (MT00285743), *Wynne-Edwards 8862* (CAN 10030311). **Coutts Inlet.** Coutts Fiord: site CF-1, *Coombs 62* (DAO 01-01000684518).

##### *Salix richardsonii* Hook. (≡Salix lanata L. subsp. richardsonii) (Fig. 2G)—Richardson’s willow—Asian (N)–amphi-Beringian–North American (N)

*Salix richardsonii* is by far the largest shrub in the flora area, reaching a height of 1.5 m. It is restricted mostly to warmer sheltered inland valleys, but small plants were also found at two locations on the Barnes Plateau in Agguttinni TP (Gillespie et al. 2024).

**Specimens examined: Agguttinni TP.** Generator Lake: site GL-20, *Bültmann & Raynolds MKR-2022-50* (CAN 10169263). Kangiqtualuk Agguqti: site 3.2, *Gillespie et al. 11820* (ALA, ALTA, CAN 10116572, MT); site 3.4, *Gillespie et al. 11842* (CAN 10116571, US); site 8.2, *Gillespie et al. 11951* (CAN 10116570); site 21.1, *Gillespie et al. 12407* (CAN 10116567, npsp). Kangiqtualuk Uqquqti: site 27.1, *Gillespie et al. 11606* (CAN 10116565, npsp), *11607* (CAN 10116573, npsp), *11678* (CAN 10116569, CHARS, MT). Tasialuk S: site 26.6, *Gillespie et al. 11604* (CAN 10116566). Tingijattut: site 15.8, *Gillespie et al. 12175* (CAN 10116568, QFA). **Clyde Area.** Inugsuin Head: site IG-7, *Parmelee & Seaborn 3916* (DAO 01-01667562); site IG-19, *Hainault 4065* (CAN 10023915, CAN 10032116, O V547943, QK 59841). Kangiqtugaapik Head: site KP-1, *Dansereau 500624-0257* (MT00285733), *500624-0564* (MT00285729), *500713-0356* (MT00285734), *500713-0560* (MT00285731), *500721-0151* (MT00285732), *500721-0556* (MT00285730), *500726-0865* (MT00285784), *500726-0966* (MT00285782), *500803-0258* (MT00285781), *Wynne-Edwards 8808* (CAN 10032115); site KP-17, *Wynne-Edwards 8901* (CAN 10032114).

#### ﻿Saxifragaceae

***Micranthes* Haw.**

##### Key to *Micranthes* [adapted from Brouillet and Elvander (2009a) and Healy and Gillespie (2004)]

**Table:** 

1	Inflorescences with some or all flowers replaced with bulbils; basal leaves oblanceolate, petiole ± absent	***M. foliolosa***
–	Inflorescences without bulbils; basal leaf blades elliptic, oblong, obovate, or ovate, petiole present, flattened	**2**
2	Inflorescences one to several dense head-like clusters of numerous flowers; petals white or essentially so (sometimes becoming pink with age); flowering stems (0.5–)1–2.5 mm wide, moderately to densely hairy, with conspicuous long coarse white hairs; plants usually robust in appearance	***M. nivalis***
–	Inflorescences open cymes of fewer flowers; petals pink or less often white; flowering stems 0.3–1 mm wide, usually sparsely hairy, with short fine hairs that are usually inconspicuous; plants delicate in appearance	***M. tenuis***

##### *Micranthes foliolosa* (R.Br.) Gornall (≡*Saxifraga foliolosa* R.Br.; =Saxifraga stellaris L. var. comosa Retz.)—Leafy saxifrage—Circumpolar

**Specimens examined: Agguttinni TP.** Atagulisaktalik: site 22.3, *Gillespie et al. 11498* (CAN 10116546); site 24.2, *Gillespie et al. 11545* (CAN 10116545, npsp). Clark Fiord: site 20.2, *Gillespie et al. 12358* (CAN 10116550). Gee Lake: site 31.4, *Gillespie et al. 11746* (CAN 10116544, MO). Generator Lake: site GL-14, *Raynolds & Bültmann MKR-2022-28* (CAN 10169291). Kangiqtualuk Agguqti: site 3.2, *Gillespie et al. 11824* (CAN 10116547). Kuugaaluk: site 16.3, *Gillespie et al. 12206* (CAN 10116542); site 16.8, *Gillespie et al. 12226* (ALTA, CAN 10116541, CHARS, MT, npsp); site 19.4, *Gillespie et al. 12315* (ALA, CAN 10116549, QFA). Refuge Harbour: site 11.2, *Gillespie et al. 12019* (CAN 10116543). Tasialuk N: site 26.4, *Gillespie et al. 11584* (CAN 10116548). **Clyde Area.** Cape Hewett: site CH-1, *Platt 480C* (NY 3481078). Clyde River: site CR-1, *Dutilly 1443* (CAN 10060533, MIN 1382173), *1444* (CAN 10060458, S18-20195), *Martin 19* (DAO 794971), *Polunin 2604* (CAN 10060507); site CR-2, Forbes 39 (DAO 73306). Hewett Cache: site HC-1, *Hainault 3797* (DAO 838971, QK 60121). Hoffman Cove: site HF-1, *Bartlett 245a* (US 03778958). Inugsuin Head: site IG-15, Parmelee & Seaborn 3885a (DAO 794972), 3885b (DAO 794969), site IG-30, *Hainault 3640* (DAO 838970, QK 59571). Inugsuin Mouth: site IM-1, *Hainault 3822* (CAN 10060497, DAO 838972, QK 59675). Kangiqtugaapik Head: site KP-1, *Dansereau 500705-0454* (MT00286867), *500822-0461* (MT00286869), *Wynne-Edwards 8951* (CAN 10060559); site KP-5, *Wynne-Edwards 9089* (CAN 10060511). McBeth Valley: site MB-1, *Hainault 3772* (QK 59552). **Home Bay.** Cape Hooper: site HO-1, *Elven 3453/99* (ALA V133505, CAN 10060543); site HO-3, *Parmelee & Seaborn 3804* (DAO 463901). Ekalugad FOX-C: site FC-1, *Richardson & Webber 42* (CAN 10060501). Kangirlugag Fiord: site KG-1, *Webber 1232* (COLO 02447464), *1275* (COLO 02447472). Rocknoser Fiord: site RF-1, Smith VP-74-61 (CAN 10060498).

##### *Micranthes nivalis* (L.) Small (≡*Saxifraga nivalis* L.)—Snow saxifrage—Circumpolar-alpine

Also recorded from Scott’s Bay (as *S. nivalis*) (Taylor 1863), but no collection located.

**Specimens examined: Agguttinni TP.** Arviqtujuq Kangiqtua NW: site 18.4, *Gillespie et al. 12277* (CAN 10121721, MO). Atagulisaktalik: site 23.11, *Gillespie et al. 11538* (CAN 10121716, npsp). Clark Fiord: site 20.2, *Gillespie et al. 12361* (CAN 10121726). Generator Lake: site GL-18, *Raynolds & Bültmann MKR-2022-37* (CAN 10169264). Kangiqtualuk Agguqti: site 5.3, *Gillespie et al. 11863* (CAN 10121717, MT); site 8.2, *Gillespie et al. 11914* (CAN 10121718); site 21.4, *Gillespie et al. 12423* (CAN 10121722). Kangiqtualuk Uqquqti: site 28.2, *Gillespie et al. 11647* (CAN 10121724). Refuge Harbour: site 11.3, *Gillespie et al. 12031* (ALTA, CAN 10121720, npsp). Stewart Valley: site 15.1, *Gillespie et al. 12112* (CAN 10121719). Tasialuk S: site 26.6, *Gillespie et al. 11597* (CAN 10121725, CHARS). Tingijattut: site 15.4, *Gillespie et al. 12160* (CAN 10121727). **Clyde Area.** Clyde River: site A22.4, *Gillespie et al. 12468* (CAN 10121723); site CR-1, *Dutilly 1442* (QFA0040233), *Martin 32* (DAO 01-01667475); site CR-2, *Forbes 91* (DAO 01-01667474). Hoffman Cove: site HF-1, *Bartlett 245* (US 03780162). Inugsuin Head: site IG-1, *Hainault 3649b* (QK 59530), *3672* (QK 59531); site IG-6, *Parmelee & Seaborn 3836* (DAO 01-01667476). Inugsuin Mouth: site IM-1, *Hainault 3955* (DAO 01-01667473, O V549717, QK 59787). Kangiqtugaapik Head: site KP-1, *Dansereau 500629-2183* (MT00285799), *500723-0352* (MT00286759), *Wynne-Edwards 8957* (CAN 10001512); site KP-24, *Wynne-Edwards 8833* (CAN 10001612). **Home Bay.** Cape Hooper: site HO-1, *Elven 3513/99* (O V549706). Ekalugad FOX-C: site FC-1, *Richardson & Webber 31* (COLO 02441608); site FC-2, *Oswald 344* (QK 135588). Kangirlugag Fiord: site KG-1, *Webber 1226b* (COLO 02441590). Rocknoser Fiord: site RF-1, *Smith VP-53-61* (CAN 10100389 [pro parte, plants y]).

##### *Micranthes tenuis* (Wahlenb.) Small (≡ *Saxifraga tenuis* (Wahlenb.) Harry Sm.)—Slender saxifrage—Circumpolar

**Specimens examined: Agguttinni TP.** Kangiqtualuk Agguqti: site 5.4, *Gillespie et al. 11868* (CAN 10116748, npsp). Kangiqtualuk Uqquqti: site 28.1, *Gillespie et al. 11638* (CAN 10116747). **Clyde Area.** Inugsuin Head: site IG-1, *Hainault 3649A* (CAN 10100390, DAO 01-01667478, QK 59578); site IG-9, *Parmelee & Seaborn 3939* (DAO 01-01667477). **Home Bay.** Ekalugad Head: site EK-2, *Stock s.n..* (UBC V162138); site EK-4, *Philpot et al. s.n.* (COLO 02441616). Rocknoser Fiord: site RF-1, *Smith VP-53-61* (CAN 10100389 [pro parte, plants x]).

#### ﻿*Saxifraga* L.

##### Key to species of *Saxifraga* [adapted from Porsild and Cody (1980), Brouillet and Elvander (2009b), and Blondeau (2015)]

**Table:** 

1	Leaves opposite, imbricated; flowers bright pink or pink-purple; plants low, mat-forming	***S. oppositifolia***
–	Leaves alternate; flowers white, yellow, or pale purple; plants tufted, cespitose or sometimes mat-forming	**2**
2	Leaves entire; petals yellow, often with orange spots	**3**
–	Leaves toothed or lobed; petals white or pale purple	**4**
3	Leaves cauline, petioles absent, blades linear to narrowly oblong, succulent, margins usually spinose-ciliate; ovary ½-inferior; plants matted or cushion forming	***S. aizoides***
–	Leaves basal and cauline, petioles present (except on distal leaves), blades oblanceolate, thin or slightly fleshy, margins eciliate or sparsely reddish brown-ciliate; ovary superior; plants loosely tufted	***S. hirculus***
4	Inflorescences with most flowers replaced by red bulbils, sometimes with one large white flower borne on the tip of the flowering stem	***S. cernua***
–	Inflorescences lacking bulbils	**5**
5	Leaves sessile, blades narrowly cuneate, sharply 3-toothed; petals white with yellow to dark orange spots	***S. tricuspidata***
–	Leaves petiolate, blades orbicular, reniform, or cuneate-flabellate, 3–5 lobed, lobes rounded or obtuse, not sharply pointed; petals white or pale purple, without spots	**6**
6	Basal leaf blades cuneate-flabellate, surfaces stipitate-glandular	***S. cespitosa***
–	Basal leaf blades orbicular or reniform, surfaces glabrous or sometimes sparsely hairy	**7**
7	Underground stolons absent; flowering stems usually much longer than and much exserted above leaves; basal leaf blades mostly 3–5-lobed; petals white to pale purple, usually with purple veins or central stripe	***S. hyperborea***
–	Underground stolons present; flowering stems usually about same length as leaves (sometimes shorter in subsp. arctolitoralis), not or only somewhat exserted above leaves; basal leaf blades mostly 5-lobed; petals white (*S. rivularis*)	**8**
8	Hypanthia sparsely short stipitate-glandular, hairs 0.1–0.3(–0.4) mm long; flowering stems 2.7–7 cm tall, glabrous or sparsely stipitate-glandular; hair crosswalls usually colorless, rarely pale purple; plants wholly green or purple only in inflorescences	**S. rivularis subsp. rivularis**
–	Hypanthia sparsely to densely long stipitate-glandular, hairs (0.2–)0.3–0.6(–1.1) mm long, flowering stems 1.7–3 cm tall, sparsely to densely stipitate-glandular; hair crosswalls usually purple; plants mostly purple (at least inflorescences)	**S. rivularis subsp. arctolitoralis**

##### *Saxifraga aizoides* L. (Fig. 12D, E)—Yellow mountain saxifrage—North American (N)–amphi-Atlantic–European

**Specimens examined: Agguttinni TP.** Kangiqtualuk Agguqti: site 8.5, *Gillespie et al. 11963* (CAN 10116632, npsp). Kangiqtualuk Uqquqti: site 27.1, *Gillespie et al. 11709* (CAN 10116631, MT). **Clyde Area.** Inugsuin Head: site IG-11, *Parmelee & Seaborn 3981* (DAO 01-01667511). Kangiqtugaapik Head: site KP-1, *Dansereau 500803-1763* (MT00203860), *Wynne-Edwards 8966* (CAN 10060007); site KP-18, *Wynne-Edwards 9045* (CAN 10060006); site KP-25, *Wynne-Edwards 9060* (CAN 10060072).

##### *Saxifraga cernua* L.—Nodding saxifrage—Circumpolar-alpine

Also recorded from Scott’s Bay (Taylor 1863), but no collection located, and Coutts Inlet (https://www.inaturalist.org/observations/237459897).

**Specimens examined: Agguttinni TP.** Atagulisaktalik: site 22.2, *Gillespie et al. 11483* (CAN 10121779). Gee Lake: site 31.4, *Gillespie et al. 11753* (CAN 10121781). Generator Lake: site GL-14, *Raynolds & Bültmann MKR-2022-57* (CAN 10169292). Kangiqtualuk Agguqti: site 5.3, *Gillespie et al. 11864* (CAN 10121783); site 8.2, *Gillespie et al. 11945* (CAN 10121784). Kangiqtualuk Uqquqti: site 28.4, *Gillespie et al. 11659* (CAN 10121780, CHARS); site 31.10, *Gillespie et al. 11777* (CAN 10121782, MT, npsp). Kuugaaluk: site 19.4, *Gillespie et al. 12316* (ALTA, CAN 10121788). Refuge Harbour: site 12.5, *Gillespie et al. 12052* (CAN 10121785); site 14.2, *Gillespie et al. 12094* (CAN 10121786). Tasialuk S: site 21.12, *Gillespie et al. 12441* (CAN 10121789). Tingijattut: site 15.4, *Gillespie et al. 12163* (CAN 10121787). **Clyde Area.** Cape Hewett: site CH-1, *Platt 480D* (NY 3474335). Clyde River: site CR-1, *Dutilly 9350* (QFA0140042), *Martin 20* (DAO 01-01667515, MT00203839); site CR-2, *Forbes 139* (DAO 01-01667514); site CR-3, *Dare 45* (CAN 10092840). Hoffman Cove: site HF-1, *Bartlett 241* (US 03778802). Inugsuin Head: site IG-6, *Parmelee & Seaborn 3837* (DAO 01-01667973). Inugsuin Mouth: site IM-1, *Hainault 3873* (CAN 10058811, DAO 01-01667512, O V549537, QK 59772). Kangiqtugaapik Head: site KP-1, *Dansereau 500629-2188* (MT00203814), *500726-1274* (MT00203831); site KP-11, *Wynne-Edwards 8877* (CAN 10058760). McBeth Valley: site MB-1, *Hainault 3763* (DAO 01-01667513, QK 59554). **Home Bay.** Cape Hooper: site HO-1, *Elven 3097/99* (O V549544), *3457/99* (ALA V13350, CAN 10058815). Ekalugad FOX-C: site FC-1, *Richardson & Webber 2* (CAN 10058805, COLO 02445989, QK 71114). Ekalugad Head: site EK-1, *Ryder s.n.* (COLO 02445757); site EK-4, *Crompton et al. s.n.* (COLO 02445724). *Crompton et al. s.n.* (COLO 02445765). Kangirlugag Fiord: site KG-1, *Webber 1223b* (COLO 02445708), *Webber 1292* (COLO 02445690), *Webber 1310* (COLO 02445773). Rocknoser Fiord: site RF-1, *Smith VP-81-61* (CAN 10058813).

##### Saxifraga cespitosa L. subsp. cespitosa (=Saxifraga cespitosa subsp. uniflora (R.Br.) A.E.Porsild)—Tufted saxifrage—Circumpolar-alpine

**Specimens examined: Agguttinni TP.** Atagulisaktalik: site 23.3, *Gillespie et al. 11510* (CAN 10121714, MT, US). Gibbs esker: site 20.12, *Gillespie et al. 12394* (CAN 10121711, npsp, QFA, WIN). Kangiqtualuk Agguqti: site 8.2, *Gillespie et al. 11938* (CAN 10121712). Kangiqtualuk Uqquqti: site 28.3, *Gillespie et al. 11649* (ALTA, CAN 10121713, CHARS, MO, US). **Clyde Area.** Clyde River: site A22.4, *Gillespie et al. 12467* (ALA, CAN 10121715). Hoffman Cove: site HF-1, *Bartlett 219* (CAN 10060260, US 03778487). Inugsuin Head: site IG-1, *Hainault 3692* (QK 59719), *4039* (QK 59536); site IG-7, *Parmelee & Seaborn 3917* (DAO 01-01667472); site IG-15, *Parmelee & Seaborn 3884* (DAO 01-01667471). Kangiqtugaapik Head: site KP-1, *Dansereau 500629-2191* (MT00285797, MT00285798), *Wynne-Edwards 8845* (CAN 10085505), *8958* (CAN 10060282); site KP-26, *Wynne-Edwards 8815* (CAN 10085504). **Coutts Inlet.** Coutts Fiord: site CF-1, *Coombs 51* (DAO 01-01667639).

##### *Saxifraga hirculus* L.—Yellow marsh saxifrage—Circumboreal-polar

Although widespread across the Canadian Arctic, including Baffin Island, *S. hirculus* is known only from one locality in the flora area, first mapped by Porsild (1957). Porsild and Cody (1980) also mapped a second locality; the dot apparently is on the northernmost fiord complex in the Home Bay area. However, no plant collections are known from this area, and the dot possibly corresponds to Isabella Bay in the Clyde area, where Bartlett collected at Hoffman Cove; no collection has been found for verification. Also recorded from Scott’s Bay (Taylor 1863), but no collection located. Polunin (1940), apparently incorrectly, mentioned there were numerous records from almost all localities (known at the time) on central Baffin Island, but did not cite any collections.

**Specimens examined: Clyde Area.** Kangiqtugaapik Head: site KP-1, *Dansereau 500628-3152* (MT00203919), *Wynne-Edwards 8848* (CAN 10059492); site KP-2, *Martin 46* (DAO 01-01667516, QFA0615111).

##### *Saxifraga hyperborea* R.Br.—Pygmy saxifrage—Circumpolar-alpine

In North America, prior to Aiken et al. (2007), this species was usually included in a broadly defined *Saxifraga rivularis* (Polunin 1940; Porsild 1957; Porsild and Cody 1980). This species complex remains taxonomically difficult.

**Specimens examined: Agguttinni TP.** Atagulisaktalik: site 22.3, *Gillespie et al. 11493* (CAN 10122140); site 24.2, *Gillespie et al. 11548* (CAN 10122142, MT, npsp). Generator Lake: site GL-19, *Raynolds & Bültmann MKR-2022-35* (CAN 10169283). Kangiqtualuk Uqquqti: site 30.5, *Gillespie et al. 11728* (CAN 10122141). **Clyde Area.** Clyde River: site CR-1, *Martin 33* (DAO 01-01667442), *Polunin 594* (CAN 10001402 [pro parte, plants 1]); site CR-3, *Dare 49* (CAN 10092863 [pro parte, plants 1]), *52* (CAN 10092862). Hewett Cache: site HC-1, *Hainault 3799* (QK 59548). Kangiqtugaapik Head: site KP-1, *Dansereau 500725-0361* (MT00287048); site KP-20, *Wynne-Edwards 8993* (CAN 10001409). **Home Bay.** Cape Hooper: site HO-1, *Elven 3520/99* (ALA V133503). Ekalugad FOX-C: site FC-1, *Richardson & Webber 63* (CAN 10001406). Ekalugad Head: site EK-4, *Philpot et al. s.n.* (COLO 02451540). Kangirlugag Fiord: site KG-1, *Webber 1226a* (CAN 10001405, COLO 02451342), *1293* (COLO 02451573).

##### Saxifraga oppositifolia L. subsp. oppositifolia (Fig. 3F)—Purple mountain saxifrage—Circumpolar-alpine

Also recorded from Scott’s Bay (Taylor 1863), but no collection located.

**Specimens examined: Agguttinni TP.** Atagulisaktalik: site 22.3, *Gillespie et al. 11497* (CAN 10116584, MT). Clark Fiord: site 20.1, *Gillespie et al. 12337* (CAN 10116579), *12346* (CAN 10116580). Generator Lake: site GL-18, *Raynolds & Bültmann MKR-2022-36* (CAN 10169286). Gibbs esker: site 20.12, *Gillespie et al. 12397* (CAN 10116574). Kangiqtualuk Agguqti: site 3.2, *Gillespie et al. 11823* (CAN 10116575); site 8.1, *Gillespie et al. 11909* (CAN 10116578, WIN). Kangiqtualuk Uqquqti: site 28.1, *Gillespie et al. 11629* (ALTA, CAN 10116577, CHARS, MO). Marble Lake: site 31.2, *Gillespie et al. 11741* (CAN 10116576, npsp, US). Refuge Harbour: site 11.2, *Gillespie et al. 12022* (CAN 10116582, npsp). Tingijattut: site 15.4, *Gillespie et al. 12158* (CAN 10116583). **Clyde Area.** Clyde River: site A22.4, *Gillespie et al. 12469* (CAN 10116581); site CR-1, *Martin 16* (DAO 01-01667524, QFA0615132); site CR-2, *Forbes 17* (DAO 01-01667523), *52* (DAO 01-01667525), *114* (DAO 01-01667527). Inugsuin Head: site IG-1, *Hainault 3667* (CAN 10060806, DAO 01-01667522, O V549758, QK 59688); site IG-14, *Parmelee & Seaborn 3893* (DAO 01-01667528); site IG-15, *Parmelee & Seaborn 3883* (DAO 01-01667526). Kangiqtugaapik Head: site KP-1, *Dansereau 500604-0257* (MT00203966), *500614-1552* (MT00203958), *500614-1753* (MT00203955), *500614-1754* (MT00203962), *500624-0155* (MT00203946), *500624-0666* (MT00203965), *500624-0771* (MT00203963), *500628-3153* (MT00203945), *500629-2189* (MT00203964), *500701-0959* (MT00203947, MT00203967), *500705-0165* (MT00203944), *500723-0354* (MT00203949), *500723-1274* (MT00203943), *500803-0479* (MT00203950), *500822-0351* (MT00203959), *Wynne-Edwards 8850* (CAN 10001571); site KP-3, *Wynne-Edwards 8809* (CAN 10060800); site KP-8, *Dansereau 500823-1675* (MT00203948); site KP-18, *Wynne-Edwards 8821* (CAN 10001572). **Coutts Inlet.** Coutts Fiord: site CF-1, *Coombs 49* (DAO 01-01667641). Coutts Mouth: site CO-1, *Coombs 73* (DAO 01-01667640). **Home Bay.** Cape Hooper: site HO-1, *Elven 3446/9* (CAN 10001560), *3446/99* (O V549745), *3498/99* (ALA V133488); site HO-3, *Parmelee & Seaborn 3812* (DAO 01-01667974). Ekalugad FOX-C: site FC-1, *Richardson & Webber 28* (CAN 10061009). Ekalugad Head: site EK-1, *Ryder s.n.* (UBC V162126). Rocknoser Fiord: site RF-1, *Smith VP-1-61* (CAN 10061035), *VP-19-61* (CAN 10061034).

##### Saxifraga rivularis L. subsp. arctolitoralis (Jurtzev & V.V.Petrovsky) M.H.Jørg. & Elven—Arctic seashore saxifrage—Amphi-Beringian–North American (N)

Previously thought to be restricted to the western North American Arctic (Brouillet and Elvander 2009b), Westergaard (2010) and Westergaard et al. (2010) determined that it is also present in the eastern North American Arctic. The taxon is newly reported here for the flora area. Stolons may not be present on herbarium specimens, making this taxon difficult to distinguish from *S. hyperborea.* Further study on the taxonomically difficult *S. rivularis*-*S. hyperborea* complex is needed to more precisely determine taxon boundaries in the Canadian Arctic. Characters used to distinguish taxa often overlap, and some specimens are not easily identifiable.

**Specimens examined: Agguttinni TP.** Gee Lake: site 31.4, *Gillespie et al. 11747* (CAN 10122143). Kangiqtualuk Agguqti: site 3.1, *Gillespie et al. 11793* (CAN 10122144); site 6.3, *Gillespie et al. 11888* (CAN 10122145, MT, npsp). Refuge Harbour: site 11.3, *Gillespie et al. 12032* (CAN 10122139). **Clyde Area.** Clyde River: site CR-1, *Dutilly 1445* (MT00287053), *Polunin 594* (CAN 10001402 [pro parte, plants 2]). Inugsuin Head: site IG-7, *Parmelee & Seaborn 3920* (DAO 01-01667439 [pro parte, plants b]). Inugsuin Mouth: site IM-1, *Hainault 3830* (DAO 01-01667446, O V549668, QK 59682). Kangiqtugaapik Head: site KP-1, *Dansereau 500720-0453* (MT00287049), *Wynne-Edwards 8916* (CAN 10001401); site KP-7, *Dansereau 500801-0663* (MT00287050); site KP-11, *Dansereau 500707-1653* (MT00287051). **Home Bay.** Cape Hooper: site HO-1, *Elven 3450/99* (CAN 10001415, O V549673); site HO-3, *Parmelee & Seaborn 3806* (DAO). Rocknoser Fiord: site RF-1, *Smith VP-52-61* (CAN 10087451).

##### Saxifraga rivularis L. subsp. rivularis—Alpine brook saxifrage—Amphi-Atlantic–European (N)–Asian (NW)

Taylor (1863) listed *S. rivularis* as occurring at Cape Adair and Scott’s Bay, but no collections located; his records could apply to either subspecies or to *S. hyperborea*.

**Specimens examined: Agguttinni TP.** Kangiqtualuk Agguqti: site 6.2, *Gillespie et al. 11879* (CAN 10122147, CHARS, MT). Kuugaaluk: site 16.3, *Gillespie et al. 12207* (CAN 10122149, MT); site 16.8, *Gillespie et al. 12229* (CAN 10122150, MT, npsp). Ravenscraig Harbour: site 18.6, *Gillespie et al. 12282* (CAN 10122148, MT). Tingijattut: site 15.9, *Gillespie et al. 12182* (CAN 10122146, MT). **Clyde Area.** Clyde River: site 10.1, *Gillespie et al. 11993* (CAN 10122151); site CR-1, *Martin 4* (DAO 01-01667440); site CR-2, *Forbes 13* (DAO 01-01667437), *51* (DAO 01-01667445), *123* (DAO 01-01667441); site CR-3, *Dare 49* (CAN 10092863 [pro parte, plants 2]). Inugsuin Head: site IG-1, *Hainault 3690* (CAN 10061374, DAO 01-01667447, O V549675, QK 59713); site IG-7, *Parmelee & Seaborn 3920* (DAO 01-01667439 [pro parte, plants a]); site IG-8, *Parmelee & Seaborn 3951* (DAO 01-01667438); site IG-9, *Parmelee & Seaborn 3941* (DAO 01-01667444). Kangiqtugaapik Head: site KP-1, *Dansereau 500719-0252* (MT00287052). **Home Bay.** Ekalugad Head: site EK-1, *Philpot et al. s.n.* (COLO 02451565); site EK-4, *Philpot et al. s.n.* (COLO 02451557). Kangirlugag Fiord: site KG-1, *Webber 1180* (COLO 02451516), *Webber 1180b* (COLO 02451581), *Webber 1301* (CAN 10061392, COLO 02451524).

##### *Saxifraga tricuspidata* Rottb. (Fig. 2D)—Three-toothed saxifrage—North American (N)

Also recorded from Cape Adair (Taylor 1863), but no collection located, and from Icy Arm, Quernbiter Fiord (https://www.inaturalist.org/observations/20818576) and Rannoch Arm, Cambridge Fiord (https://www.inaturalist.org/observations/20079472) in the Buchan Gulf area.

**Specimens examined: Agguttinni TP.** Atagulisaktalik: site 23.3, *Gillespie et al. 11508* (CAN 10121790, MO, npsp). Clark Fiord: site 20.1, *Gillespie et al. 12324* (CAN 10122138). Kangiqtualuk Agguqti: site 3.3, *Gillespie et al. 11837* (ALTA, CAN 10122133); site 8.2, *Gillespie et al. 11937* (CAN 10122135). Kangiqtualuk Uqquqti: site 27.1, *Gillespie et al. 11609* (CAN 10122132, MT). Refuge Harbour: site 14.1, *Gillespie et al. 12085* (CAN 10122134, CHARS, US). Stewart Valley: site 15.1, *Gillespie et al. 12117* (CAN 10122136, QFA, WIN). Tingijattut: site 15.5, *Gillespie et al. 12165* (ALA, CAN 10122137); site TG-2, *Dare et al. 91* (CAN 10092866). **Clyde Area.** Hoffman Cove: site HF-1, *Bartlett 244* (US 03781442).Inugsuin Head: site IG-6, *Parmelee & Seaborn 3868* (DAO 01-01667520); site IG-16, *Hainault 3724* (DAO 01-01667518, O V549860, QK 59608). Kangiqtugaapik Head: site KP-1, *Dansereau 500604-0564* (MT00203890), *500629-2193* (MT00203887, MT00203889), *500716-0864* (MT00203892, MT00203893), *Wynne-Edwards 8909* (CAN 10118965); site KP-2, *Martin 47* (DAO 01-01667521, QFA0615093); site KP-13, *Dansereau 500608-0353* (MT00203888, MT00203891). McBeth Valley: site MB-1, *Hainault 3753* (DAO 01-01667519, O V549861, QK 59589). **Coutts Inlet.** Coutts Fiord: site CF-1, *Coombs 50* (DAO 01-01667642). **Home Bay.** Ekalugad Head: site EK-2, *Stock s.n..* (COLO 02445740).

### ﻿﻿Excluded taxa

Antennaria alpina (L.) Gaertn subsp. canescens (Lange) Chmiel.—Porsild and Cody (1980) and Aiken et al. (2007) mapped one locality in the Clyde area. Three collections (*Hainault 3661, 3678*, *4067*) from the head of Inugsuin Fiord, determined by Porsild as this species, have been located; two have been redetermined to A. media subsp. compacta (*3661, 3678*), and one to A. friesiana subsp. friesiana (*4067*). Chmielewski (1997) mapped and cited *Hainault 3661* as *A. media* subsp. *compacta. Hainault 4038*, originally determined as A. alpina subsp. canescens but not seen by Porsild, has been redetermined to A. friesiana subsp. friesiana.

Antennaria monocephala (Torr. and A. Gray) DC. subsp. angustata (Greene) Hultén—This species was mapped in the Clyde area in Porsild and Cody (1980) (as *A. angustata* Greene) and specifically from Inugsuin Fiord and Kangirlugag Fiord in Aiken et al. (2007), based on *Parmelee & Seaborn 3863* and *Webber 1253.* Both collections, plus *Hainault 3685*, were determined as A. monocephala subsp. angustata by J. Chmielewski in 1995. Here we redetermine all three collections to A. friesiana subsp. friesiana based on all inflorescences with multiple heads and on leaf shape and vestiture. In the Canadian Arctic Islands, this species is known from numerous collections on south and southeastern Baffin Island and is recorded from scattered localities on Banks, Melville, Southampton, and Victoria Islands (Porsild and Cody 1980; Aiken et al. 2007; Saarela et al. 2020a; Saarela et al. 2023; Saarela et al. 2020b); although previously mapped from Axel Heiberg (Aiken et al. 2007), these collections have been redetermined to A. friesiana subsp. friesiana.

*Anthoxanthum arcticum* Veldkamp—Porsild (1957) and Porsild and Cody (1980) mapped this species (as *Hierochloe pauciflora* R.Br.) in the northern part of the flora area, with the dot possibly corresponding to Cambridge Fiord or the Buchan Gulf area, respectively (dot placement differs somewhat). No plant collections are known from this area, suggesting the dot may be misplaced; the closest collection localities are Coutts Inlet to the north and Cape Adair to the south. Polunin (1940) indicated that he had seen no authenticated record of the species from central Baffin, and that he had redetermined Taylor’s (1863)*H. pauciflora* specimens at K from the Cumberland Gulf area (south of the flora area) to *Dupontia fisheri* and *Deschampsia caespitosa* s.l. If Porsild’s dot is based on a 19^th^-century historical record, it could well be misidentified. The species is scattered across the Canadian Arctic Islands, including on northern and western Baffin Island. Although we tentatively exclude the species from the flora area, it should be looked for.

*Astragalus alpinus L.*— Porsild (1957) and Porsild and Cody (1980) mapped this species from the northern part of the flora area, the dot possibly corresponding to the Cape Adair area; Aiken et al. (2007) mistakenly mapped the same dot near Clyde River. Polunin (1940) cited no records from central Baffin Island, and Taylor (1863) included no Fabaceae in his list. The position of Porsild’s dot in an area where, at the time, the only collector known to have visited was Taylor, suggests the dot may have been misplaced. This arctic-alpine species is found on northern, western, and southern Baffin Island, but no collections are known from the flora area nor from the Cumberland Peninsula to the south. The species is known from the Isortoq River area on the northwest side of the Barnes Ice Cap, not far from the flora area (*Webber 583*CAN, QK).

Braya glabella Richardson subsp. glabella—Harris (1985) mapped a collection in the Agguttinni TP/Clyde area but did not cite it in his specimens examined. Based on his map, Aiken et al. (2007) mapped the taxon on Kangiqtualuk Uqquqti (within Agguttinni TP). No specimen was found to document this taxon in the flora area. See notes under B. glabella subsp. purpurescens.

*Campanula rotundifolia* L.— Porsild (1957) and Porsild and Cody (1980) mapped one locality from the Agguttinni TP/Clyde area, likely based on Polunin (1940), who cited Taylor’s (1863) record (as “Campanula linifolia Haenk.”) from Scott’s Bay. If correct, this would be the northernmost record of the species in North America (excluding Greenland). No collections from the flora area have been located. This species is found in the Canadian Arctic Islands only on southeastern Baffin Island (Aiken et al. 2007). The closest known localities to the flora area are located about 100 km southeast of Cape Hooper, the southern and easternmost locality in the flora area (e.g, *Raynolds MKR-2018-92*CAN). The species should be looked for in the flora area.

*Carex microglochin* Wahlenb—Presence in the flora area at Clyde River is based on *Dutilly 1483* (CM414569), but this appears likely to be a labelling error. Four specimens of the same number (*Dutilly 1483*) at QFA and US are *Phippsia algida*. Labels indicate the same locality and date, but the CM label header is “Flora of Ungava and Labrador,” and specific collection information is hand-written, whereas those of the QFA and US sheets have the header “Flore de l”Arctique” and are typed. This species is rare in the Canadian Arctic Islands, known from only a few localities on Victoria, Banks, and southernmost Baffin islands (Aiken et al. 2007; Saarela et al. 2020b; Saarela et al. 2023). This distribution, plus the fact that it has not been recollected at Clyde River, suggests the CM specimen was mislabelled and the species is not present in the flora area. Nevertheless, it should be looked for in the vicinity of Clyde River. If present, it would be the northernmost record for the Eastern Canadian Arctic.

*Cerastium beeringianum* Cham. & Schltdl.— Porsild (1957) and Porsild and Cody (1980) mapped this species as occurring in the Clyde area. They did not recognize *C. arcticum* as a species present in the Canadian Arctic and instead included collections of *C. arcticum* under *C. beeringianum* and *C. alpinum*. See notes under *C. arcticum*.

*Cerastium regelii* Ostenf.— Polunin (1940) tentatively cited a single collection from Clyde River (*Polunin 2592*, not located) but noted that it approached *C. alpinum*. Porsild (1957) and Porsild and Cody (1980) mapped the species from one locality in the Agguttinni TP/Clyde area, possibly based on Polunin’s collection; however, the dot is not on Clyde River but rather closer to the Scott Inlet/Cape Adair area. A specimen from along a sandy roadside at Cape Hooper, *Parmelee & Seaborn 3824*, was identified as *C. regelii* by J.K. Morton in 2001; however, except for the cushion-like habit, the specimen does not fit his concept of *C. regelii* (Morton 2005a), and we reidentified it as *C. arcticum*, which can have a cushion-like habit when growing in sand. In Canada, *C. regelii* is mostly restricted to the Canadian Arctic Islands and is most common in the northern and western islands; on Baffin Island, it has been recorded from a few scattered localities (Aiken et al. 2007; Saarela et al. 2020a) (2 CAN specimens from Koudjuak plain verified here: *Boles RB00-206*, *Manning 155*). We have seen no specimens to validate the putative report(s) and thus exclude the species from the flora area.

*Draba alpina* L.—This species was recorded by Polunin (1940) from Clyde River based on his collections from 1934 and 1936, mapped by Porsild (1957) and Porsild and Cody (1980) in the Clyde area, but not mapped in the flora area by Aiken et al. (2007). *Draba alpina* was more broadly circumscribed and the name often misapplied in the past (Al-Shehbaz et al. 2010); here we follow Al-Shehbaz et al. (2010) and Elven et al. (2011) in treating the species in a narrower sense. All specimens we have seen that have been referred to *D. alpina* in the flora area (e.g., *Wynne-Edwards 8881*CAN, *8878*CAN) have been redetermined to either *D. corymbosa* or *D. lactea*.

*Draba arctogena* (Ekman) Ekman—Mapped by Aiken et al. (2007) from the head of Clyde Inlet (possibly also the head of Inugsuin Fiord), but no specimens from the flora area are listed in the Aiken et al. (2007) mapping database, nor are there any specimens at CAN previously determined as this species. Aiken et al. (2007) noted that their treatment of this species is provisional and its distribution unclear.

*Draba crassifolia* Graham—Mapped by Aiken et al. (2007) from Coutts Inlet (based on *Coombs 81*DAO) and the head of Inugsuin Fiord (*Parmelee & Seaborn 3854*DAO). Both specimens have been reidentified as *D. fladnizensis*.

Festuca rubra L. subsp. rubra.— Aiken et al. (2007) incorrectly mapped a collection at Home Bay; this mapping error resulted from an incorrect latitude on the label of *Elven 3553/99* (CAN 10013373), a collection gathered in the vicinity of Iqaluit.

*Hippuris vulgaris* L.— Aiken et al. (2007) mapped this species from Inugsuin Head based on *Parmelee & Seaborn 3898* (DAO 448193/01-01435907). This specimen has been reidentified as *H. lanceolata*.

*Micranthes hieraciifolia* (Waldst. & Kit.) Haw. (=*Saxifraga hieraciifolia* Waldst. & Kit.)— Taylor (1863: 328) recorded this species (as *S. hieraciifolia*) at Scott’s Bay, specifically on the “banks of a river south of Scott’s Bay”, the only locality he collected it in the area covered in his account (numerous localities along Baffin Bay and Davis Straits). No collection has been found for verification. The species is found scattered throughout most of the Canadian Arctic Islands, including the northern and west-central Baffin area, but is not known from southern and eastern Baffin Island (Porsild and Cody 1980; Aiken et al. 2007; Saarela et al. 2020b). The closest known localities to the flora area are in the vicinity of Pond Inlet (e.g., *Gillespie 6032*CAN) on northern Baffin Island (ca. 100 km northwest of the flora area) and Air Force Island (*Baldwin & Packer 1995*CAN) off the west-central coast of Baffin Island. The species should be looked for in the flora area.

*Omalotheca sylvatica* (L.) Sch.Bip. & F.W.Schultz—Taylor (1863) listed this species (as *Gnaphalium sylvaticum* L.) from Cape Adair and Scott’s Bay. No species of *Omalotheca* Cass. or *Gnaphalium* St.-Lag. are known from the Canadian Arctic Islands (Porsild 1957; Porsild 1964; Porsild and Cody 1980; Aiken et al. 2007; Gillespie et al. 2015). The furthest north that *Omalotheca* is known to be present in the eastern Canadian Arctic is northern Quebec and Labrador (including one adjacent offshore island now part of Nunavut), where three species of *Omalotheca* (*O. sylvatica*, *O. supina* (L.) DC., and *O. norvegica* (Gunnerus) Sch.Bip. & F.W.Schultz) are known, with the latter two present in the Arctic zone and *O. supina* occurring as far north as the northern tip of Quebec (Brouillet 2023; Payette 2023a). Polunin (1940: 358) listed, with “considerable hesitation”, *Gnaphalium norvegicum* Gunn. as present on Baffin Island based on Taylor’s account of its presence (as *G. sylvaticum*) at four localities on eastern Baffin. He mentioned that he had seen no specimens from north of Ramah in Labrador and that some of Taylor’s Greenland records were considered erroneous by Porsild and Porsild (1920). Given that the genus has not been recollected nor seen anywhere on Baffin Island over the past 160 years, we consider (Taylor 1863) records to be erroneous and conclude that *Omalotheca* is not present in the flora area.

Pedicularis langsdorffii Fisch. ex Steven subsp. arctica (R.Br.) Pennell ex Hultén—Taylor (1863) recorded this species (as *P. langsdorffii*) from Scott’s Bay. Polunin (1940) tentatively treated this and a Taylor record from Cape Searle as *P. lanata* (neither specimen was apparently seen by Polunin because he cited Taylor’s 1863 article rather than collections). Other than this possible record, the species has not been recorded from the flora area (Polunin 1940; Porsild 1957; Porsild and Cody 1980; Aiken et al. 2007; Sokoloff et al. 2016), and no specimens were located. Although found throughout most of the Canadian Arctic Islands and mapped on northern and southwest Baffin Island (Porsild 1957; Porsild and Cody 1980; Aiken et al. 2007), the species appears to be absent on Baffin Island. All specimens from Baffin Island previously determined as this species at CAN have been redetermined (mostly to *P. hirsuta*), including *Polunin 2471*CAN and *Gillespie 6051*CAN (the only specimen in the Aiken et al. (2007) mapping database), both from Pond Inlet. The species is known to be present on Bylot Island, off the northern coast of Baffin Island (e.g., *Duclos*CAN 10081110).

*Phippsia concinna* (Th.Fr.) Lindeb.— Aiken et al. (2007) mapped two collections in the flora area, *Dutilly 1482* (CAN 514012) from Clyde River and *Webber 1307* (CAN 312002) from Botany Bay Kangerdluak Fiord. We have redetermined both as *Phippsia algida*.

*Poa alpina* L.—Aiken et al. (2007) mapped *P. alpina* in the flora area based on two collections. The identity of the specimen *Wynne-Edwards 9080A* (CAN 10015681), collected at the head of Clyde Inlet in 1950, has long been problematic. R.J. Soreng first determined it as “? *Poa alpina* X” in 1990. The specimen has since been redetermined as “*Poa* sp., plant abnormal, likely infected” (det. L.J. Gillespie & R.J. Soreng, 2024). Although superficially resembling *P. arctica* in some features (e.g., extravaginal branching) *and P. alpina* in others (e.g., callus web absent), spikelets appear somewhat abnormal with abnormal, sterile “seeds.” This appears to be a mutant form with development distorted by infection, and it is not possible to identify to species. A second collection mapped to Home Bay by Aiken et al. (2007) is a mapping error resulting from an incorrect latitude on the label of *Elven 3554/99* (CAN 10015644), a collection gathered near Iqaluit (Saarela et al. 2023). Taylor (1863) reported *P. alpina* at Scott’s Bay, but no collection was located, and this is likely a misidentification or an erroneous report.

*Poa flexuosa* Sm.—Porsild (1957) mapped one locality and Porsild and Cody (1980) two localities in the Clyde/Home Bay area, likely based on *Wynne-Edwards 9009*CAN from the head of Clyde Inlet and *Hainault 3717*CAN from Inugsuin Fiord, both determined by Porsild as *P. flexuosa* and subsequently redetermined to *P. glauca*. Poa flexuosa subsp. consauliae L.J.Gillespie & Soreng, the only subspecific taxon in the Canadian Arctic, is known on Baffin Island only from the Frobisher Bay area (Soreng et al. 2017); it is morphologically similar to *P. glauca*, and the two taxa can sometimes be difficult to distinguish (Soreng et al. 2017). The name *P. flexuosa* has also often been misapplied in the past to specimens of *P. arctica* (Polunin 1940). *Poa flexuosa* is unlikely to be present in the flora area and is excluded here.

*Poa nascopieana* Polunin—Polunin (1940) described this species based on a single collection from Pangnirtung, south of the flora area. Porsild (1957) mapped a second collection from the Clyde area, most likely based on *Wynne-Edwards 2072*CAN, which was originally identified as this species by Porsild but was subsequently redetermined as *Poa hartzii*. Soreng et al. (2003) considered the type specimen to be a diseased and deformed plant that is not possible to identify with certainty. The species has been treated as a synonym of P. glauca subsp. glauca (e.g., Plants of the World Online, https://powo.science.kew.org, accessed 10 Sept 2025) or as a diseased *P. arctica* (Soreng in Tropicos, www.tropicos.org/name/25557684, accessed 22 Jan 2025).

Potentilla hyparctica subsp. elatior (Abrom.) Elven & D.F.Murray—Aiken et al. (2007) mapped a collection from the Clyde area [as “southern race,” subsequently treated as subsp. elatior in Elven et al. (2011)]. This appears to be based on *Dutilly 1457* (CAN), on which most individuals were identified as the “northern type,” with one individual as “(a) southern type?” (det. R. Elven, 1999). All individuals on the sheet have been redetermined as P. hyparctica subsp. hyparctica.

*Puccinellia vaginata* (Lange) Fernald & Weath.—Porsild and Cody (1980) and Aiken et al. (2007) mapped one locality in the Home Bay area (based on *Richardson & Webber 69*CAN). This specimen has been redetermined as *P. angustata*.

*Salix calcicola* Fernald & Wiegand—Aiken et al. (2007) mapped the species on Inugsuin Fiord in the Clyde area based on *Hainault 4065* (CAN 10023915). Argus (2007) also included the southern half of the flora area in his distribution map for the species, presumably based on this collection. Argus appeared to have some hesitation with his determination, noting the prominent “richardsonsii-like stipules.” We have redetermined this specimen as *S. richardsonii* based on the narrowly elliptic leaf blades and longer lanceolate stipules (L/W = 3.5–4) (compared to blades and stipules usually ovate or broadly ovate with L/W < 2 in *S. calcicola*). We also note that a duplicate specimen (CAN 10032116) of the same collection was determined by Argus in 2010 as *S. richardsonii*. We exclude *S. calcicola* from the flora area.

Salix glauca L. var. cordifolia (Pursh) Dorn—Porsild (1957) and Porsild and Cody (1980) mapped this taxon (as *Salix cordifolia* vars. *callicarpaea* Fernald/*intonsa* Fernald and S. glauca subsp. callicarpaea (Trautv.) Böcher, respectively) from the Clyde area. The dot appears to be placed on Isabella Bay, where Bartlett collected at Hoffman Bay, but no collection has been located. Alternatively, their dot may have been based on Taylor’s (1863) record of the taxon (as *S. desertorum* Richardson) from Scott’s Bay, although the dot would be misplaced. Polunin (1940) also cited the Taylor record (as *Salix*cordifolia Pursh) but appeared to treat it as questionable. Aiken et al. (2007) and Argus (2007) did not consider *S. glauca* to be present in the flora area. Outside the flora area, the species is widespread across the North American low Arctic and is known on the Arctic Islands from southern Banks and Victoria Islands (var. stipulata Flod.) and on Baffin Island (var. cordifolia) from the southeast and one locality on the west-central part of the island (*Webber 368*QK) (Aiken et al. 2007; Argus 2007, 2010; Saarela et al. 2020b). Since we have not seen any collections, previous records are questionable, and the species is a conspicuous shrub that would likely not be missed by subsequent collectors, we consider *Salix glauca* s.l. not present in the flora area.

*Taraxacum lapponicum* Kihlm. ex Hand.-Mazz.—Porsild (1957) and Porsild and Cody (1980) mapped one locality in the Clyde area, presumably based on *Wynne-Edwards 9003* (CAN) from the head of Clyde Inlet. The specimen was originally determined as this species by Porsild but was subsequently redetermined as *T. ceratophorum*.

*Woodsia ilvensis* (L.) R.Br.—Porsild and Cody (1980) mapped *W. ilvensis* near the southeast boundary of the study area, a locality repeated in Aiken et al. (2007). Imprecision due to dot size and sometimes placement makes it impossible to know if the locality lies in or outside the flora area, although it is likely outside. No voucher specimen has been located. The species is considered not present in the flora area.

## ﻿﻿Additional information

### Use of AI

No use of AI was reported.

### Author ORCIDs

Lynn J. Gillespie https://orcid.org/0000-0003-3129-434X

Paul C. Sokoloff https://orcid.org/0000-0002-7053-8557

Geoffrey A. Levin https://orcid.org/0000-0003-1514-1570

### Data availability

All of the data that support the findings of this study are available in the main text or Supplementary Information.

## Supplementary materials

﻿Supplementary material 1 Notes on historical vascular plant collections from east-central Baffin Island, Nunavut, Canada Data type pdf Explanation note Notes on William E. Parry’s botanical observations on east-central Baffin Island (1819–20), Taylor’s specimens from Scott’s Inlet (1856–61), and Platt’s collections from Cape Hewitt (1947). https://binary.pensoft.net/file/1436936This dataset is made available under the Open Database License (http://opendatacommons.org/licenses/odbl/1.0/). The Open Database License (ODbL) is a license agreement intended to allow users to freely share, modify, and use this Dataset while maintaining this same freedom for others, provided that the original source and author(s) are credited.Lynn J. Gillespie, Paul C. Sokoloff, Geoffrey A. Levin

﻿Supplementary material 2 Dataset of 3206 voucher collections documenting vascular plant diversity in east-central Baffin Island, Nunavut, Canada Data type xlsx Explanation note Herbarium acronyms follow Thiers (2025 [continuously updated]). The acronym NBCC refers to the National Biodiversity Cryobank of Canada located at the Canadian Museum of Nature. https://binary.pensoft.net/file/1436937This dataset is made available under the Open Database License (http://opendatacommons.org/licenses/odbl/1.0/). The Open Database License (ODbL) is a license agreement intended to allow users to freely share, modify, and use this Dataset while maintaining this same freedom for others, provided that the original source and author(s) are credited.Lynn J. Gillespie, Paul C. Sokoloff, Geoffrey A. Levin

﻿Supplementary material 3 Maps of P. Dansereau and V.C. Wynne-Edwards’ collection sites at “Head of Clyde” (Kangiqtugaapik Head) made during the Baird 1950 Arctic expedition Data type pdf Explanation note Locations at Kangiqtugaapik Head for sites visited by P. Dansereau and V.C. Wynne-Edwards in 1950. **A.** Georeferenced sites and locations overlaid on ESRI satellite imagery (via QGIS). **B.** Dansereau’s hand-drawn map found in his field notes at the Marie-Victorin Herbarium (MT). https://binary.pensoft.net/file/1436938This dataset is made available under the Open Database License (http://opendatacommons.org/licenses/odbl/1.0/). The Open Database License (ODbL) is a license agreement intended to allow users to freely share, modify, and use this Dataset while maintaining this same freedom for others, provided that the original source and author(s) are credited.Lynn J. Gillespie, Paul C. Sokoloff, Geoffrey A. Levin

﻿Supplementary material 4 Primary collectors, dates collected, and the number of vascular plant collections made in east-central Baffin Island Data type pdf https://binary.pensoft.net/file/1436939This dataset is made available under the Open Database License (http://opendatacommons.org/licenses/odbl/1.0/). The Open Database License (ODbL) is a license agreement intended to allow users to freely share, modify, and use this Dataset while maintaining this same freedom for others, provided that the original source and author(s) are credited.Lynn J. Gillespie, Paul C. Sokoloff, Geoffrey A. Levin

﻿Supplementary material 5 Vascular plants recorded from east-central Baffin Island, with main areas and localities where they were found Data type xlsx Explanation note Taxa are listed by major clade and then alphabetically by family. The dataset records the presence of each taxon in the four main areas (Agguttinni Territorial Park, Clyde area, Coutts Inlet, and Home Bay) and in the 21 main localities. Presence indicated by “X” refers to documentation by voucher herbarium specimens examined and cited in the text; “[X]” indicates unnamed hybrids or species documented with specimens identifiable only to the species level that are represented in the flora by multiple subspecies (neither are included in the locality totals); “iN” indicates taxa known in a locality only from verified iNaturalist records. Also recorded for each taxon are the total number of localities and collections, and the global distribution (adapted from Elven et al. 2011). The number of collections recorded for each main area and locality is also given. Values in the “Number of collections” column reflect the number of collections containing each species; collections with multiple species are counted more than once, so the sum in this column (3241) exceeds the total number of collections (3206). https://binary.pensoft.net/file/1436940This dataset is made available under the Open Database License (http://opendatacommons.org/licenses/odbl/1.0/). The Open Database License (ODbL) is a license agreement intended to allow users to freely share, modify, and use this Dataset while maintaining this same freedom for others, provided that the original source and author(s) are credited.Lynn J. Gillespie, Paul C. Sokoloff, Geoffrey A. Levin

﻿Supplementary material 6 Photograph-documented iNaturalist observations of vascular plant species in east-central Baffin Island Data type xlsx Explanation note For each observation, the scientific name, locality, coordinates, date observed, and the iNaturalist URL are provided. All potential new records for a locality were examined and verified, if possible. Observations documenting new localities for a species are mentioned in the species notes in the annotated checklist with their iNaturalist URL. https://binary.pensoft.net/file/1436941This dataset is made available under the Open Database License (http://opendatacommons.org/licenses/odbl/1.0/). The Open Database License (ODbL) is a license agreement intended to allow users to freely share, modify, and use this Dataset while maintaining this same freedom for others, provided that the original source and author(s) are credited.Lynn J. Gillespie, Paul C. Sokoloff, Geoffrey A. Levin

﻿Supplementary material 7 Vascular plant taxa recorded and/or mapped for east-central Baffin Island in previous Canadian Arctic floras (Polunin 1940; Porsild 1957; Porsild and Cody 1980; Aiken et al. 2007) and other floristic publications pertinent to the flora area (Taylor 1863; Hainault 1967) Data type xlsx Explanation note Taxa are organized by major clade, family, and then alphabetically by species. Taxa recorded with a specific locality or mapped are indicated with “x”; those that Taylor (1863) or Polunin (1940) recorded as widespread in their flora areas but without specific localities are indicated with “w.” Taxon names, if different from those used here, are given in parentheses under each publication. https://binary.pensoft.net/file/1436942This dataset is made available under the Open Database License (http://opendatacommons.org/licenses/odbl/1.0/). The Open Database License (ODbL) is a license agreement intended to allow users to freely share, modify, and use this Dataset while maintaining this same freedom for others, provided that the original source and author(s) are credited.Lynn J. Gillespie, Paul C. Sokoloff, Geoffrey A. Levin

﻿Supplementary material 8 Review of vascular plant taxa reported for east-central Baffin Island in previous floras and other publications Data type pdf Explanation note Review of vascular plant taxa reported or mapped for east-central Baffin Island in Taylor (1863), Polunin (1940), Porsild (1957), Hainault (1967), Porsild and Cody (1980), and Aiken et al. (2007). https://binary.pensoft.net/file/1436943This dataset is made available under the Open Database License (http://opendatacommons.org/licenses/odbl/1.0/). The Open Database License (ODbL) is a license agreement intended to allow users to freely share, modify, and use this Dataset while maintaining this same freedom for others, provided that the original source and author(s) are credited.Lynn J. Gillespie, Paul C. Sokoloff, Geoffrey A. Levin

﻿Supplementary material 9 List of species from east-central Baffin Island recorded in Taylor (1863) Data type xlsx Explanation note Species names used by Taylor (1863), current accepted names used in the annotated checklist or in the excluded taxa section, and relevant distribution in the flora area, as listed by Taylor (Scott’s Bay, Cape Adair, or widespread), are provided. https://binary.pensoft.net/file/1436944This dataset is made available under the Open Database License (http://opendatacommons.org/licenses/odbl/1.0/). The Open Database License (ODbL) is a license agreement intended to allow users to freely share, modify, and use this Dataset while maintaining this same freedom for others, provided that the original source and author(s) are credited.Lynn J. Gillespie, Paul C. Sokoloff, Geoffrey A. Levin

﻿Supplementary material 10 List of species from east-central Baffin Island recorded in Polunin (1940) Data type xlsx Explanation note Species names used in Polunin (1940), current accepted names used in the annotated checklist or in the excluded taxa section, whether the taxon is accepted in or excluded from the flora area, location recorded (or widespread according to Polunin), source of record, specific collection cited in Polunin, collections verified here based on pre-1940 collections, and notes on taxa and/or collections. https://binary.pensoft.net/file/1436945This dataset is made available under the Open Database License (http://opendatacommons.org/licenses/odbl/1.0/). The Open Database License (ODbL) is a license agreement intended to allow users to freely share, modify, and use this Dataset while maintaining this same freedom for others, provided that the original source and author(s) are credited.Lynn J. Gillespie, Paul C. Sokoloff, Geoffrey A. Levin
