# Supplementary material for: ﻿Vascular plants of east-central Baffin Island, Nunavut, Canada: an annotated checklist of a mid-Arctic flora
Source: PhytoKeys. 2025 Oct 13;264:1–176. doi: 10.3897/phytokeys.264.162520 (PMC12538218; doi:10.3897/phytokeys.264.162520)
Supplement: ﻿Supplementary material 1 — Notes on historical vascular plant collections from east-central Baffin Island, Nunavut, Canada [file phytokeys-264-001_article-162520__-s001.pdf]

Supplementary File 1. Notes on historical vascular plant collections from east-central Baffin Island, Nunavut, Canada

Parry's botanical observations on east-central Baffin Island

William E. Parry (1821: 273) in his journal of his 1819-20 voyages in the Canadian Arctic recorded seeing several plants along the east coast of Baffin Island at a latitude longitude of 71° 15' 34", 71° 17' 23".6, which corresponds to the area on the north side of Scotts Inlet. His notes (p.273) record "The vegetation was tolerably luxuriant in some places upon the low land which borders the sea, consisting principally of the dwarf-willow, sorrel, saxifrage, (*Saxifraga cernua*), and poppy, with a few roots of scurvy-grass". Simmon (1913) included these observations as *Cochlearia officinalis* and *Papaver radicatum* from Cape Adair and *Oxyria digyna* and *Salix arctica* from Scott's Inlet, citing Parry (1821: 273). It is unclear why Simmons gave Cape Adair as the locality of the first two species. Parry does not mention whether collections were made, and it seems unlikely they were. The botanical account by Robert Brown (Appendix XI) in Parry's publication (1821) includes only plants collected on Melville Island in the western Canadian Arctic.

Taylor's specimens from Scott's Inlet and Cape Adair

In Taylor's (1863: 234) account of vascular plants collected, he used Scott's Inlet and Scott's Bay interchangeably (the coordinates he gives for Scott's Inlet and his statement "Cape Adair, a little north of Scott's Bay" confirm they are the same location), while Scott's Inlet appears on specimens we have examined.

Taylor's collections are reported to be at BM, C, E, and K (Harvard University Herbaria & Libraries Index of Botanists https://kiki.huh.harvard.edu/databases/botanist_index.html) (Simmons 1913) (herbarium acronyms according to Thiers (2025 [continuously updated]). While we located some at BM and K (GBIF.org (28 November 2024) GBIF Occurrence Download https://doi.org/10.15468/dl.5r7ak4; GBIF.org (28 November 2024) GBIF Occurrence Download https://doi.org/10.15468/dl.d7mvxr), none of these are from the flora area. Simmons (1913) mentioned having seen much of Taylor's collection when visiting both K and BM. However, he cited Taylor's publication for localities in the flora area, which he did when he could not find the actual specimen; it thus seems that Taylor's specimens from Scott's Inlet and Cape Adair may not be at either herbarium.

Three databased specimens at the National Herbarium of Victoria (MEL) in Australia are labelled (in handwriting) with the location "Scott's Inlet, Davis Straights", without a collector or date; they are, however, appended with a small blue label: "Aberdeen. Prof. Dickie". George Dickie was a Scottish Botanist based in Aberdeen who amassed a collection of material from European explorations of North America from Taylor (1863). The handwriting on the blue label of these specimens belongs to Ferdinand Mueller, the founder of the National Herbarium of Victoria (Alison Vaughan, personal

communication, 2022), while the handwriting on the specimen labels bearing the species name and locality matches Dickie's (Senders, 1831-1895). Letters at the archives of the National Herbarium of Victoria indicated that Mueller and Dickie exchanged specimens, with MEL receiving specimens from Dickie in 1863 and 1865 (Alison Vaughan, personal communication, 2022) - these two specimens were almost certainly sent to MEL as a part of these exchanges.

Several specimens at MEL bearing Dickie's handwriting indicate that they were collected by Dr. Walker at Port Kennedy – these were gathered by David Walker, surgeon on the McClintock Arctic expedition (M'Clintock 1859). However, neither McClintock's account or Hooker's synopsis of Walker's Flora (Hooker 1860) show the party stopping at either Davis Straights or Scott's Inlet. Later, Dickie (1869a) published on specimens collected by E.P. Philpots on "the North East Shore of Lancaster Sound", however both Dickie's and Philpott's (1868) own account indicate that most collecting occurred near Cape Horsburgh (on Devon Island), and these collections were made in 1866, the year after MEL received their last known shipment from Dickie.

The only other Arctic Canadian specimens known to be in Dickie's possession (in the literature at least), are those collected by James Taylor, a fellow Aberdeen-based surgeon who collected on both the Canadian and Greenlandic sides of Baffin Bay from 1856-1861 (Taylor 1863). While Taylor's (1863) synopsis of his vascular plant collections did not indicate the specimens were in Dickie's possession, it does explicitly state Scott's Inlet (used interchangeably here with Scott's Bay), Cape Adair (now Talluruti Tulliit), and Davis Straights as collection localities, and Dickie was known to possess Taylor's collections of algae, moss, and lichens from these voyages (Dickie 1866, 1869b). For these reasons, and as these specimens were collected prior to MEL receiving specimens from Dickie, we assume that the three known specimens from Scott's Inlet at MEL were collected by James Taylor between 1856-1861.

Platt's collections from Cape Hewitt

Some of Platt's specimens from Cape Hewitt bear the date August 19, 1948, but Platt only joined the 1947 and 1954 MacMillan Arctic Expeditions (Platt 1948,1956), and his name is not present on the 1948 crew manifest (1948 MacMillan Arctic Expedition, crew list and general information, M118.04.01, Box: 32, Folder: 10. Donald and Miriam MacMillan collection, M118. George J. Mitchell Department of Special Collections & Archives, Bowdoin College Library, Brunswick, Maine). The archival records of the 1947 floristic survey don't include the data from Cape Hewitt (1947 MacMillan Chicago Geographical N. Greenland Expedition, "Copy of Report on Botanical Notes Sent to New York Botanical Garden, Rutherford Platt", 1947, 1954, M118.04.01, Box: 32, Folder: 4. Donald and Miriam MacMillan collection, M118. George J. Mitchell Department of Special Collections & Archives, Bowdoin College Library, Brunswick, Maine) nor does Platt (1948) mention Cape Hewitt, contributing to this confusion. Pending further research, these specimens seem most likely to have been collected in 1947.

Dickie G (1866) Notes on a collection of algae procured in Cumberland Sound by Mr. James Taylor, and remarks on arctic species in general. Botanical Journal of the Linnean Society, 9(36): 235–243.

Dickie G (1869a) Notes on a collection of plants from the north-east shore of Lancaster Sound. Botanical Journal of the Linnean Society, 11(49): 32–35.

Dickie G (1869b) Notes on Mosses, &c., collected by Mr. James Taylor on the shores of Davis Straits. Botanical Journal of the Linnean Society, 10(48): 461–467.

Hooker JD(1860) An Account of the Plants collected by Dr. Walker in Greenland and Arctic America during the Expedition of Sir Francis McClintock, RN, in the Yacht 'Fox.'. Botanical Journal of the Linnean Society, 5(18):79–89.

M'Clintock FL (1859) In the Arctic Seas: A Narrative of the Discovery of the Fate of Sir John Franklin and His Companions. Porter & Coates, Philadelphia.

Parry WE (1821) Journal of a voyage for the Discovery of a North-West Passage from the Atlantic to the Pacific; performed in the years 1819–20 in H. M. S. Hecla and Griper. John Murray, London.

Philpots, P. 1868. An Account of the Land in the Vicinity of Cape Horsburgh, Lat. 74° 44' 24", N., Long. 79° W., and of the Island Discovered There. Proceedings of the Royal Geographical Society of London, 13(5): 372–375. https://doi.org/10.2307/1799691

Platt R (1948) Flowers at the Edge of the Polar Ice Cap. Journal of the New York Botanical Garden 49: 77–87.

Platt R (1956) Flowers in the Arctic. Scientific American 194: 88–100.

Senders D (1831-1895) Asa Gray correspondence files of the Gray Herbarium, 1820-1904. gra00078. Archives of the Gray Herbarium, Harvard University. https://iiif.lib.harvard.edu/manifests/view/drs:49717478$335i

Simmons HG (1913) A survey of the phytogeography of the Arctic American archipelago, with some notes about its exploration. Lunds universitets årsskrift 9: 1–183.

Taylor J (1863) Notice of Flowering Plants and Ferns collected on both sides of Davis Straits and Baffin's Bay. Transactions of the Botanical Society of Edinburgh 7: 323–334. doi:10.1080/03746606309467845

Thiers B (2025 [continuously updated]) Index Herbariorum: A global directory of public herbaria and associated staff. New York Botanical Garden's Virtual Herbarium. http://sweetgum.nybg.org/science/ih/ [accessed January 2025]
