# Supplementary material for: ﻿Vascular plants of east-central Baffin Island, Nunavut, Canada: an annotated checklist of a mid-Arctic flora
Source: PhytoKeys. 2025 Oct 13;264:1–176. doi: 10.3897/phytokeys.264.162520 (PMC12538218; doi:10.3897/phytokeys.264.162520)
Supplement: ﻿Supplementary material 4 — Primary collectors, dates collected, and the number of vascular plant collections made in east-central Baffin Island [file phytokeys-264-001_article-162520__-s004.pdf]

Supplementary File 4. Primary collectors, dates collected, and the number of vascular plant collections made in east-central Baffin Island.

Primary Collector	Year(s)	Total number of sheets	Total number of collections
Bartlett	1938	38	33
Bull	2017	2	2
Bültmann	2022	2	2
Church	1966 (8), 1967 (3)	11	11
Coombs	1948	53	50
Crompton	1966	35	35
Dansereau	1950	750	571
Dare	2017	50	49
Dutilly	1936 (33), 1941 (27)	100	60
Elven	1999	146	80
Forbes	1988 (45), 1989 (24), 1990 (47)	116	116
Gillespie	2021	1825	850
Hainault	1965	950	326
Hammar	1999	3	3
Malte	1927	27	20
Martin	1958	80	63
McMillan	1909	1	1
Molau	1999	1	1
Oswald	1993	31	31
Oughton	1939	4	4
Parmelee	1967	186	159
Philpot	1966	47	47
Platt	1947 (4), 1948 (14)	19	18
Polunin	1934 (28), 1936 (15)	43	43
Raynolds	2022	42	42
Richardson	1967	152	72
Röthlisberger	1950	10	10
Ryder	1967	24	24
Sadler	1983	1	1
Sanson	1938	2	2
Smith	1961	63	63
Starr	2008	2	2
Stock	1967	5	5
Taylor	1856-1861	3	3
Webber	1967	207	107
Wynne-Edwards	1950	306	300
	Sum	5337	3206
