# Supplementary material for: ﻿Vascular plants of east-central Baffin Island, Nunavut, Canada: an annotated checklist of a mid-Arctic flora
Source: PhytoKeys. 2025 Oct 13;264:1–176. doi: 10.3897/phytokeys.264.162520 (PMC12538218; doi:10.3897/phytokeys.264.162520)
Supplement: ﻿Supplementary material 8 — Review of vascular plant taxa reported for east-central Baffin Island in previous floras and other publications [file phytokeys-264-001_article-162520__-s008.pdf]

Supplementary File 8. Review of vascular plant taxa reported for east-central Baffin Island in previous floras and other publications.

Taylor (1863) recorded 47 species from the flora area, 14 at Cape Adair and 42 at Scott's Inlet (Supplementary Files 7, 9). Only 3 collections from Scott's Inlet were found and verified (*Cerastium arcticum*, *Ranunculus hyperboreus* subsp. *hyperboreus*, *Stellaria humifusa*). Of the remaining 44 species, 34 are mentioned in the notes in the Annotated Checklist. Six species are discussed in the Excluded Species section (*Campanula linifolia* (see *C. rotundifolia*), *Gnaphalium sylvaticum* (see *Omalotheca sylvatica*), *Pedicularis langsdorfii*, *Poa alpina*, *Salix desertorum* (see *Salix glauca* L. var. *cordifolia*), and *Saxifraga hieraciifolia*. Four species names were not considered because we are unsure which species they refer to due to circumscriptions having changed considerably over time (*Potentilla nivea*) or due to misapplied names or misidentifications (*Carex rigida* Gooden., *Draba hirta* L., *Draba rupestris* "Br"). This list of 47 species is an underestimate since Taylor considered an additional 19 species to be common in the broader area surveyed (eastern Baffin Island and western Greenland) and did not mention specific localities for these (*Carex rariflora* (Wahlenb.) Sm., *Cerastium alpinum*, *Colpodium latifolium* R.Br. (=*Arctagrostis latifolia*), *Diapensia lapponica*, *Empetrum nigrum*, *Eriophorum angustifolium*, *Festuca brevifolia* (=*F. brachyphylla*), *Hierochloe alpina*, *Luzula arcuata* (Wahlenb.) Sw. (misapplied name for *Luzula confusa* or *L. nivalis*), *Lycopodium alpinum* L. (misapplied name for *Huperzia arctica*), *Oxyria reniformis* Hook. (=*O. digyna*), *Papaver nudicaule* L. (misapplied name for *Oreomecon* sp.), *Poa arctica*, *Polygonum viviparum* (=*Bistorta vivipara*), *Pyrola rotundifolia*, *Ranunculus nivalis*, *Silene acaulis*, *Saxifraga cespitosa*, *Vaccinium uliginosum*). All of these common species are known from the flora area with the exception of *Carex rariflora*, which is known on Baffin Island but only south and west of the flora area; the three misapplied names most likely refer to similar species that do occur in our area. Including these 18 widespread species (excluding *C. rariflora*) and removing the six excluded species gives a maximum total of 55 species accepted here recorded by Taylor for the flora area.

Polunin (1940) in his Botany of the Canadian Eastern Arctic publication recorded 64 species from four specific localities in the flora area (Supplementary Files 7, 10). These were based on literature records and collections from Cape Adair and Scott's Inlet by Taylor (1863), collections by Malte from Clyde River in 1927, Polunin's own collections and observations made at Clyde River in 1934 and 1936, and several collections by S.C. Knapp of the Hudson Bay's Company and J.L. Hanham from Clyde River and Isabella Bay, respectively. Polunin (1940) considered an additional 21 species to be common and widespread in his Central Baffin region (Cape Adair to the Cumberland Peninsula) without indicating specific localities; all are currently known from the flora area, and most are common (except *Saxifraga hirculus*). Including these 21 common species and excluding 7 species (*Campanula rotundifolia*, *Cerastium regelii*, *Draba alpina*, *Gnaphalium norvegicum*, *Poa alpina*, *Salix cordifola*, *Saxifraga hieraciifolia*) discussed in the Excluded Species section gives a maximum total of 78 species accepted here recorded by Polunin for the flora area. Polunin cited specific collections for only 12 species; here we provide pre 1940 voucher collections (by Polunin, Malte, and Taylor) for 42 species in the flora area, including 12 of the common species not specifically recorded as in the flora area.

Hainault (1966) published the only vascular plant species list for localities in the flora area subsequent to Taylor (1863) and Polunin (1940), based on collections from his fieldwork in the Inugsuin Fiord area, June 6 to August 28, 1965 (Supplementary File 7). He listed 89 taxa (88 species, one additional subspecies) which correspond to 85 taxa (84 species, one subspecies) as

recognized here, from three localities: Inugsuin base camp (=Inugsuin Head) (83 species [82 currently recognized]), outer Inugsuin Fiord (=Inugsuin Mouth) (61 [59]), and McBeth Valley (40). As was common at the time, he treated *Stellaria longipes* subsp. *longipes* as multiple species with four in his list. Hainault cited no collections as vouchers for his species list. We located 326 collections from this fieldwork, which document 105 taxa (104 species, one subspecies) as treated here. The increase in species numbers is due to new identifications and updated taxonomies, especially in taxonomically complex genera such as *Draba*, *Papaver*, and *Potentilla*.

Subsequent to Polunin (1940), three Canadian Arctic floras provided dot maps of species occurrences across the Canadian Arctic but cited no voucher collections documenting these occurrences. Porsild (1957) mapped 139 taxa (138 species, one additional subspecies) as occurring in east-central Baffin Island in his Illustrated Flora of the Canadian Arctic Archipelago (Supplementary File 7). We exclude 11 species (*Astragalus alpinus*, *Campanula rotundifolia*, *Cerastium beeringinum*, *Cerastium regelii*, *Draba alpina*, *Hierochloe pauciflora* (=*Anthoxanthum arcticum*), *Hippuris vulgaris*, *Poa flexuosa*, *Poa nascopieana*, *Salix cordifolia* var. *callicarpea* (=*Salix glauca* var. *cordifolia*), *Taraxacum lapponicum*; see Excluded Species section) for a total number of 128 taxa (127 species, one subspecies). We were able to verify all but three species (*Arenaria sajanensis* (=*Cherleria biflora*), *Festuca baffinensis*, *Matricaria ambigua* [=*Tripleurospermum maritimum* subsp. *phaeocephalum*]) based on pre-1957 collections. Porsild and Cody's Vascular Plants of Continental Northwest Territories (1980), while focusing on the mainland Arctic, nevertheless provided updated maps for the entire Canadian Arctic. They mapped 150 taxa (149 species, one subspecies) in the flora area (including four species now included within *Stellaria longipes* subsp. *longipes*), which correspond to 147 species as currently recognized, and added 10 species to the flora: *Antennaria compacta* (=*A. media* subsp. *compacta*), *Draba cinerea*, *Draba fladnizensis*, *Epilobium arcticum*, *Eriophorum angustifolium*, *Ledum decumbens* (=*Rhododendron tomentosum* subsp. *decumbens*), *Sagina caespitosa*, *Salix arctophila*, *Saxifraga tenuis* (=*Micranthes tenuis*) (Supplementary File 7). We exclude 15 of these 147 taxa (*Antennaria angustata* [=*A. monocephala* subsp. *angustata*], *Antennaria canescens* [=*Antennaria alpina* subsp. *canescens*], *Poa alpina*, *Puccinellia vaginata*, *Woodsia ilvensis*, plus the 10 excluded species listed above [*Poa nascopieana* not in area covered in their flora]), add one species (*Poa hartzii*) mapped in 1957 but restricted to the Canadian Arctic Islands thus not treated in the 1980 flora, for a total of 133 taxa (132 species, one subspecies) in east-central Baffin Island. For some reason they omitted five species that were mapped in Porsild (1957) and vouchered here with collections from the 1950 expedition: *Carex maritima*, *Eutrema edwardsii*, *Potentilla vahliana*, *Ranunculus pedatifidus* var. *leiocarpus* (=*R. arcticus*), *Melandrium affine* (=*Silene involucrata* subsp. *involucrata*).

Aiken et al. (2007) in their updated Flora of the Canadian Arctic Archipelago mapped 166 taxa (162 species, 3 additional subspecies, one nothospecies) in east-central Baffin Island (Supplementary File 7). We exclude 13 taxa as not occurring in the flora area, 7 previously mapped in Porsild (1957) and Porsild and Cody (1980) and 6 not previously mapped or recorded in the flora area (*Braya glabella* subsp. *glabella*, *Draba arctogena*, *Draba crassifolia*, *Festuca* rubra subsp. *rubra*, *Phippsia concinna*, *Salix calcicola*; see Excluded Species). We exclude an additional 5 species from their list that were based on collections that have been reidentified or were mapped incorrectly, although the species are now known to occur in the flora area (*Festuca baffinensis*, *Hulteniella integrifolia*, *Poa abbreviata*, *Taraxacum holmenianum*, *Vaccinium vitis-idaea*). Taking into account these 18 excluded taxa, the total number in the flora area recorded in Aiken et al. (2007) is 148 taxa (145 species, 2 subspecies, one nothotaxon).
