# Supplementary material for: ﻿Vascular plants of east-central Baffin Island, Nunavut, Canada: an annotated checklist of a mid-Arctic flora
Source: PhytoKeys. 2025 Oct 13;264:1–176. doi: 10.3897/phytokeys.264.162520 (PMC12538218; doi:10.3897/phytokeys.264.162520)
Supplement: ﻿Supplementary material 3 — Maps of P. Dansereau and V.C. Wynne-Edwards’ collection sites at “Head of Clyde” (Kangiqtugaapik Head) made during the Baird 1950 Arctic expedition [file phytokeys-264-001_article-162520__-s003.pdf]

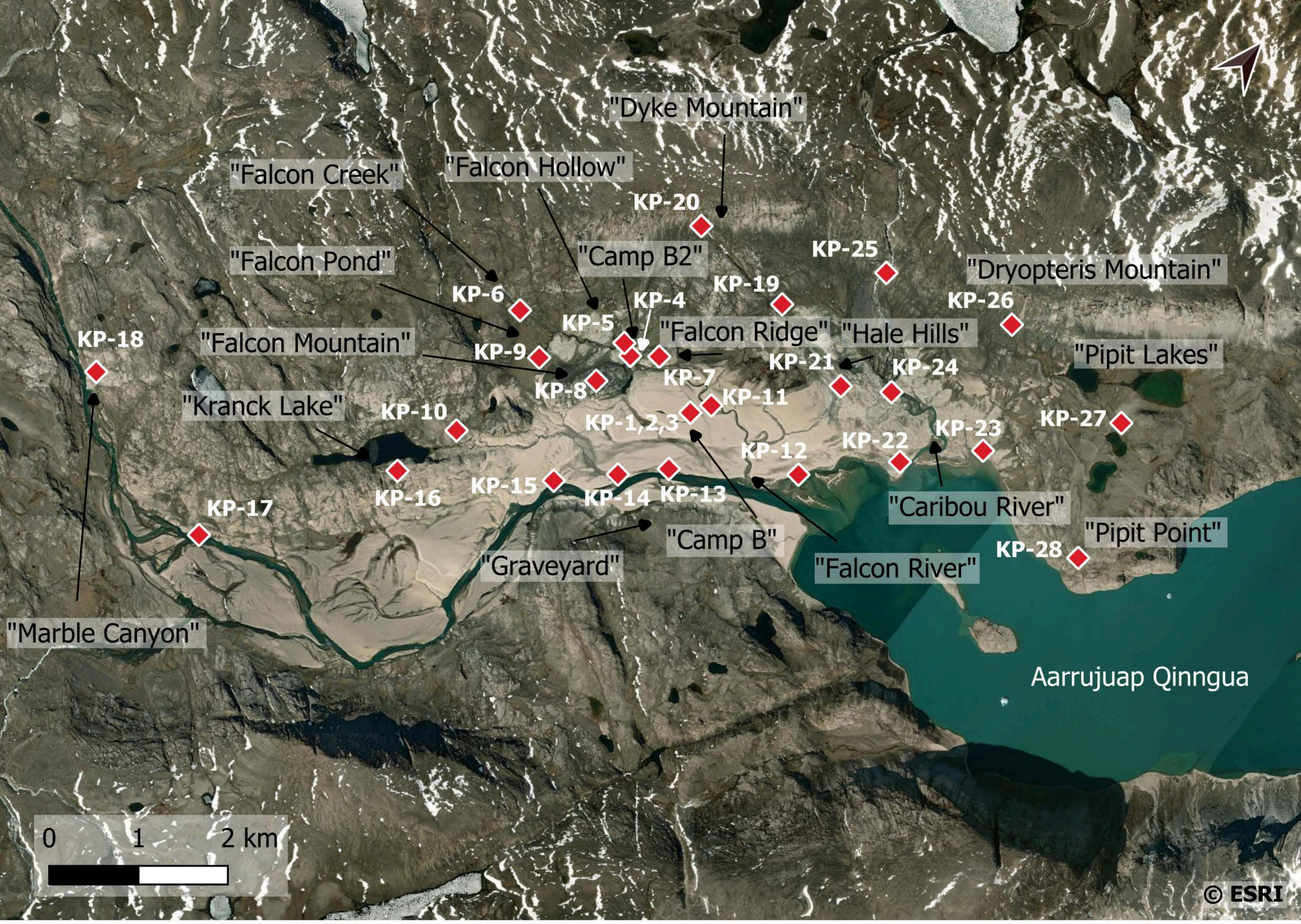
"Dyke Mountain"
"Falcon Hollow"
"Falcon Creek"
KP-20
"Camp B2"
KP-25
"Falcon Pond"
"Dryopteris Mountain"
KP-19
KP-6
KP-4
KP-26
KP-5
"Falcon Ridge"
"Hale Hills"
KP-18
"Falcon Mountain"
KP-9
KP-21
"Pipit Lakes"
KP-24
KP-7
KP-8
KP-11
"Kranck Lake"
KP-10
KP-1,2,3
KP-27
KP-23
KP-22
KP-12
KP-15
KP-16
KP-14
KP-13
"Caribou River"
KP-17
"Pipit Point"
"Camp B"
KP-28
"Graveyard"
"Falcon River"
"Marble Canyon"
Aarrujuap Qinngua
0
1
2 km
© ESRI
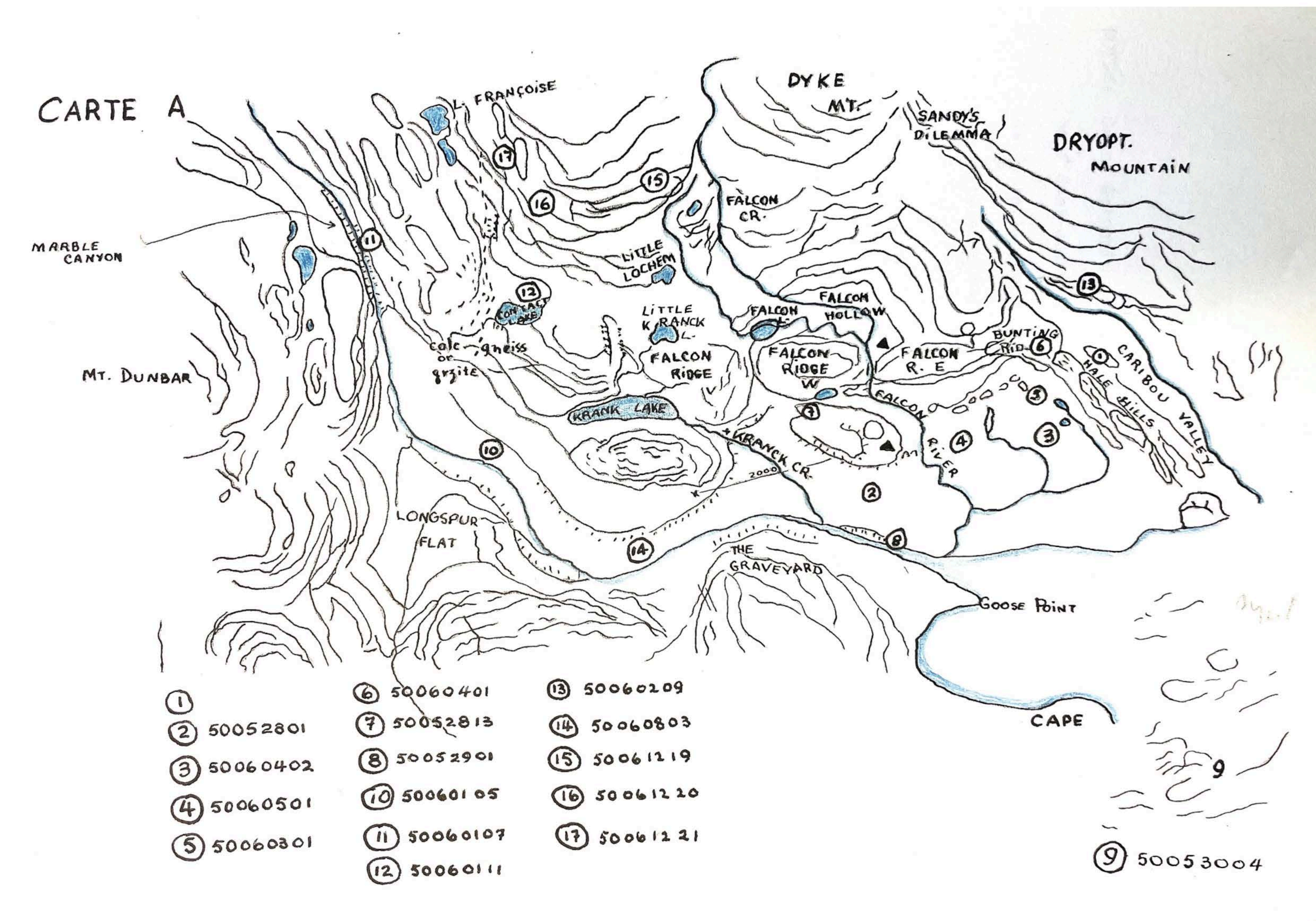
CARTE A
L. FRANÇOISE
DYKE MT.
SANDY'S DILEMMA
DRYOPT. MOUNTAIN
MARBLE CANYON
FALCON CR.
LITTLE LOCHEM
CONTACT LAKE
LITTLE KRANCK L.
FALCON HOLLOW
FALCON
BUNTING RID.
CARIBOU VALLEY
HALE HILLS
calc-gneiss or granite
MT. DUNBAR
FALCON RIDGE
FALCON RIDGE W
FALCON R. E
FALCON RIVER
KRANK LAKE
KRANCK CR.
2000
LONGSPUR FLAT
THE GRAVEYARD
GOOSE POINT
CAPE
1
2 50052801
3 50060402
4 50060501
5 50060301
6 50060401
7 50052813
8 50052901
10 50060105
11 50060107
12 50060111
13 50060209
14 50060803
15 50061219
16 50061220
17 50061221
9 50053004
